# Alkyl Radical Coupling with Phenoxy(imine)–Nickel(II)–Aryl Complexes: Evidence for a Multistep Process in C–C Bond Formation

**DOI:** 10.1021/jacs.6c02912

**Published:** 2026-04-27

**Authors:** Hao Liang, L. Reginald Mills, Marina Perez-Jimenez, Matthew V. Joannou, Heejun Lee, Steven R. Wisniewski, Eric M. Simmons, Karla Ravin, Paul J. Chirik

**Affiliations:** † Department of Chemistry, 6740 Princeton University, Princeton, New Jersey 08544, United States; ‡ Chemical Process Development, 3971 Bristol Myers Squibb Company, New Brunswick, New Jersey 08903, United States; § Department of Chemistry, University of Houston, Houston, Texas 77204-5003, United States; ∥ Department of Inorganic and Analytical Chemistry, University of Geneva, Geneva CH-1211, Switzerland

## Abstract

The reaction of well-defined phenoxy­(imine)–nickel­(II)–aryl complexes with alkyl radicals was investigated en route to C–C bond formation. A 4-dimethylaminopyridine–titanium tris­(anilide) complex was identified as a selective reagent for halogen-atom abstraction of unactivated alkyl halides, generating alkyl radicals that were subsequently captured by phenoxy­(imine)–nickel­(II)–aryl complexes. A combination of radical clock and competition experiments provided insight into ligand effects on both the phenoxy­(imine) and the nickel–aryl in radical capture and subsequent C–C bond formation. Differences between parallel and competition radical clock experiments support a multistep process for C–C bond formation. Results from ^12^C/^13^C kinetic isotope effect measurements and computational studies are also consistent with this pathway. Treatment of phenoxy­(imine)–nickel­(II)–aryl complexes with various oxidants generated transient nickel­(III) complexes that were detected by electron paramagnetic resonance (EPR) spectroscopy and intercepted by organozinc reagents to generate C–C bond formation products, supporting the intermediacy of this oxidation state in the overall process.

## Graphical abstract

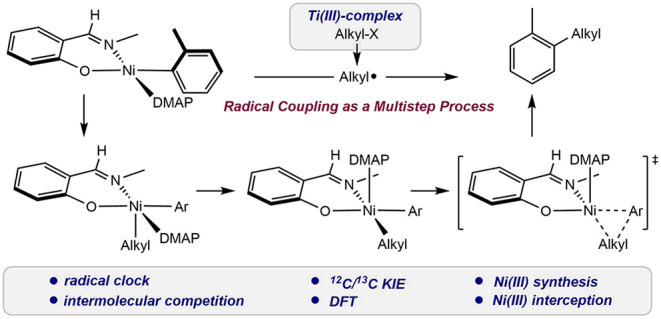

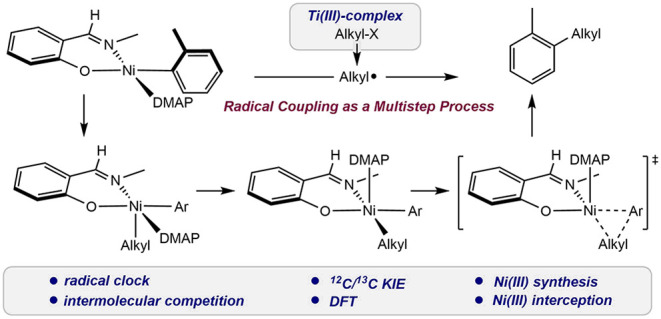

## Introduction

Catalytic cross-coupling reactions relying on C­(sp^3^)–alkyl partners are powerful and widely used strategies in organic synthesis.^1^ Such methods are increasingly attractive owing to the desire for increased C­(sp^3^) content in drug molecules.^2^ Nickel-catalyzed methods have been the most widely developed and studied for C­(sp^2^)–C­(sp^3^) bond formation. Examples include traditional thermal cross-couplings,^3^ decarboxylative methods,^4^ cross-electrophile couplings,^5^ electrochemical cross-coupling,^6^ and metallophotoredox catalysis.^7^ A unifying feature among these methods is the involvement of C­(sp^3^)-based radicals that are captured by the nickel catalyst—a key elementary sequence that has not been widely studied nor its catalyst design principles rationalized.

Recently, our laboratory has reported the development of planar nickel­(II)–aryl complexes bearing readily prepared and modified phenoxy­(imine) (FI) ligands for C­(sp^2^)–C­(sp^3^) Suzuki–Miyaura couplings between (hetero)­aryl boronic acids and alkyl bromide electrophiles (Scheme 1A).^8^ The nickel precatalysts are air stable, perform efficiently in alcohol solvents, and have been applied to C­(sp^2^)–C­(sp^3^) bond formation in the synthesis of the core of the TLR 7/8 antagonist afimetoran (Scheme 1B),^9^ a compound that is currently being investigated for the treatment of lupus.^10^ Mechanistic studies established that a square planar nickel­(II) aryl complex serves as the catalyst resting state and that alkyl radicals are formed from the alkyl halide electrophile. Notably, the planar nickel­(II) compounds were ineffective at generating the alkyl radical but were shown to capture it en route to formation of the C–C bond.

**Scheme 1:**
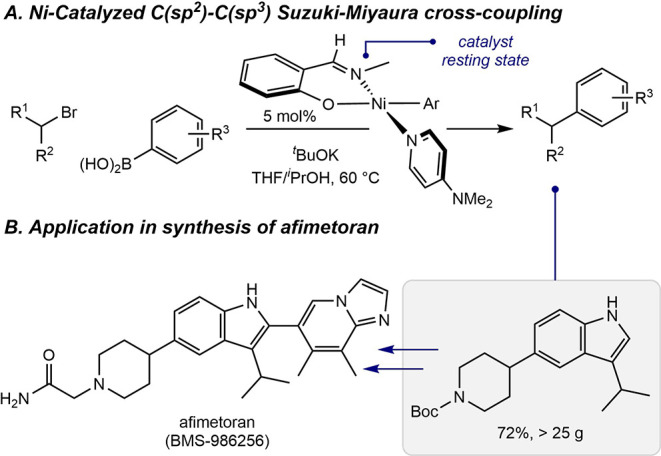
Application of Phenoxy­(Imine)–Nickel­(II)–Aryl Complexes as Precatalysts for C­(sp^2^)–C­(sp^3^) Suzuki–Miyaura Cross-Coupling under Thermal Conditions

Given the commonality of alkyl radical coupling among nickel-catalyzed cross-coupling methods, studies aimed at understanding this elementary step and catalyst features that optimize it are gaining attention. Molander and MacMillan independently demonstrated that irradiation of iridium-based photoredox catalysts promotes the reaction of potassium organotrifluoroborates or carboxylic acids with nickel­(II)–aryl complexes to furnish the products of C–C bond formation (Scheme 2A).^11^ Recently, Diao and coworkers have reported a systematic evaluation of the kinetics and thermodynamics associated with the capture of photochemically generated alkyl radicals by cationic nickel­(II)–aryl complexes supported by tridentate pyridine bis­(oxazoline) ligands (Scheme 2A).^12^ Based on radical clock experiments and data science analysis, a single-step, concerted inner-sphere pathway was proposed for direct C­(sp^2^)–C­(sp^3^) bond formation from a nickel­(II) complex that obviates the intermediacy of nickel­(III). Independent studies by Weix and Biswas^13^ and Sevov et al.^14^ have reported the evaluation of reactions between well-defined nickel­(II)–aryl complexes and alkyl halides in the presence of chemical or electrochemical reductants, implicating radical capture by the nickel­(II)–aryl as a key step leading to C–C bond formation in reductive cross-coupling methods (Scheme 2B).

**Scheme 2:**
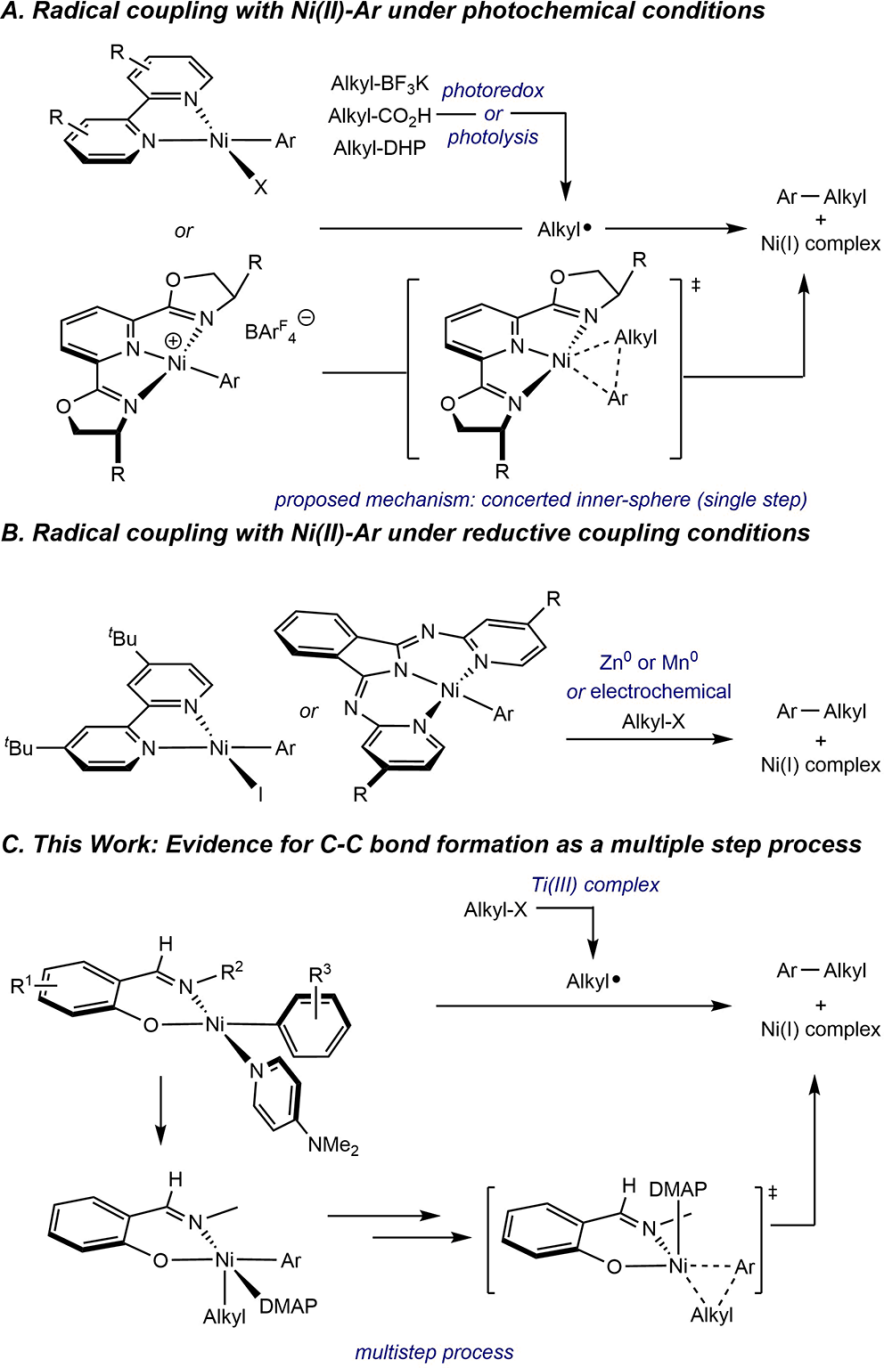
Radical Coupling Reactions Studied with Nickel­(II)–Aryl Complexes

Given the utility and versatility of phenoxy­(imine)-nickel catalysts in catalytic C­(sp^2^)–C­(sp^3^) Suzuki–Miyaura reactions, including applications to API synthesis, attention was devoted to understanding radical capture at neutral nickel­(II)-aryl complexes bearing bidentate L,X-type ligands and the subsequent pathway to C–C bond formation. In particular, the influence of N-imine substituents, the electronic properties of the phenoxy­(imine) ligand, and the nickel aryl group was examined with the goal of establishing the design principles for catalysts that promote this important product-forming step. Here, we describe the results of these studies, which include applications of radical clocks, competition experiments, natural abundance kinetic isotope effect measurements, spectroscopic data, and computational studies that all support a multistep pathway for C–C bond formation involving radical capture and subsequent reductive elimination from a nickel­(III)–aryl–alkyl intermediate (Scheme 2C).

## Results and Discussion

### Methods for the Generation of C­(sp^3^)–Alkyl Radicals

Previous studies from our laboratory have reported the generation of C­(sp^3^)-based radicals from the addition of alkyl bromides to Ni­(COD)_2_.^8^ Treatment of phenoxy­(imine)–nickel­(II)–aryl complexes with 1 equiv of Ni­(COD)_2_ along with 10 equiv of alkyl halide resulted in slow C–C bond formation at ambient temperature and furnished ∼10% yield of the arylated product after 30 min. Increasing the reaction temperature accelerated radical generation; however, even after full consumption of the nickel aryl complex, the yield of the C–C bond-formation product remained below 60%. Control experiments revealed that ligand exchange and loss of mass balance of the nickel complex occurred when heating the phenoxy­(imine)–nickel­(II)–aryl complexes (see Supporting Information for details). In addition, likely exchange between the coordinated 4-dimethylaminopyridine (DMAP) in the phenoxy­(imine)–nickel complexes and Ni­(COD)_2_ further complicated the reaction and its reproducibility.

These limitations motivated the search for more reliable and reproducible methods for alkyl radical generation. Thermal methods were preferred, given that the catalytic cross-coupling reactions proceed in the absence of external stimuli such as light or electrochemical potential. Unfortunately, most reported thermal methods rely on harsh reagents or strong oxidizing conditions^11,13,15^ that are incompatible with the phenoxy­(imine)–nickel­(II)–aryl complexes, as decomposition or deleterious side reactions were observed. However, titanium­(III) reagents^16^ are attractive as halogen atom acceptors, as previous work from Wolczanski and Covert^17^ and Cummins et al.^18^ has independently reported well-defined, three-coordinate alkoxide and anilide complexes for this purpose (Figure 1A). More recently, Peters, Fu et al. applied a tris­(anilido)–titanium complex to generate stabilized alkyl radicals that in the presence of a copper amide complex, promoted C–N bond formation.^19^

**Figure 1:**
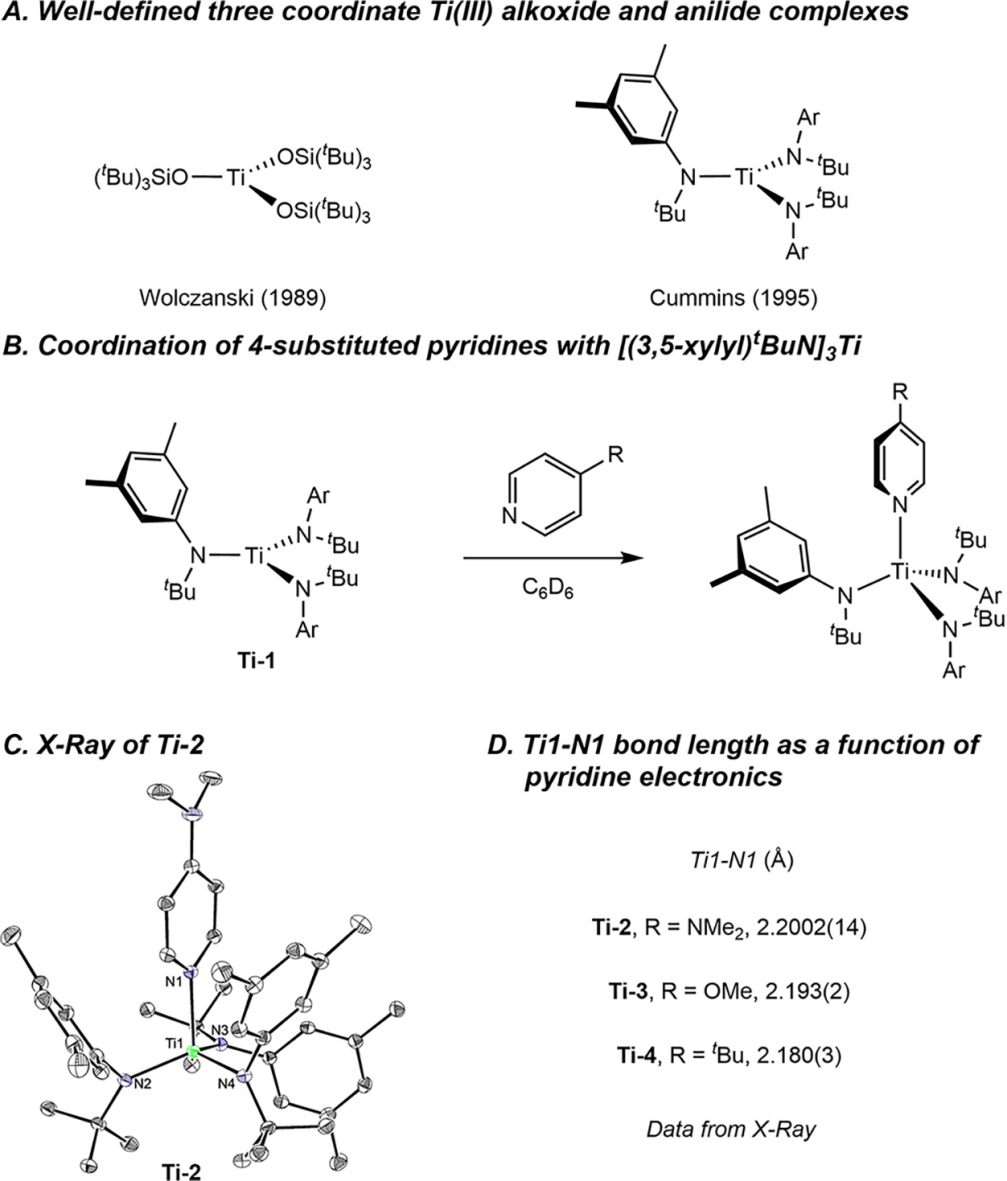
A. Three-coordinate Ti­(III) complexes. B. Reaction of 4-substituted pyridines with [(*Ar*)^t^BuN]_3_Ti. C. Representation of the solid-state structure of **Ti-2** at 30% probability ellisoids, with hydrogen atoms omitted for clarity. D. Analysis of structures of [(*Ar*)^t^BuN]_3_Ti–pyridine analogs.

In contrast to the prior studies with tris­(anilido) titanium complexes, the goal of this study was to generate nonstabilized alkyl radicals^20^ with short lifetimes that are relevant to nickel-catalyzed C­(sp^2^)–C­(sp^3^) Suzuki–Miyaura cross-coupling. These reactive fragments may then be captured by phenoxy­(imine)–nickel­(II)–aryl complexes, which have been identified as the catalytic resting states.^8^ In an initial experiment, the addition of [(*Ar*)^t^BuN]_3_Ti (**Ti-1**; *Ar* = 3,5-xylyl) to a thawing toluene solution containing phenoxy­(imine)–nickel­(II)–aryl (**Ni-1**) and 4-bromo-*N*-Boc piperidine (**1**) produced the desired C–C coupling product **2** in 51% yield, along with unidentified nickel products (Scheme 3). Subsequent analysis identified two key limitations. First, **Ti-1** was also effective for the capture of sterically accessible alkyl radicals;^21^ and second, **Ni-1** underwent deleterious side reactions with the titanium complex (see Supporting Information for details).

**Scheme 3:**
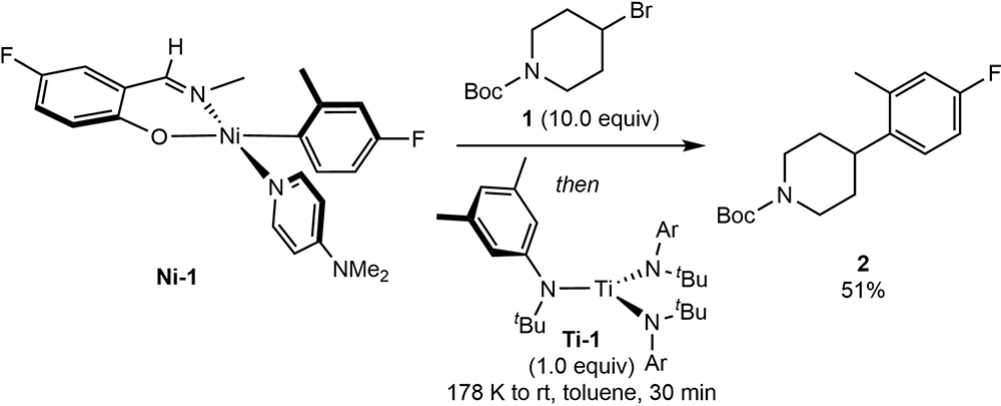
In Situ Radical Generation with **Ti-1** and Radical Coupling with **Ni-1**

To circumvent these issues, **Ti-1** was treated with a series of 4-substituted pyridines to reduce its reactivity toward the generated alkyl radical and obviate the transfer of the pyridine ligand from the nickel. Coordination of pyridine derivatives with **Ti-1** has been reported previously by Cummins and coworkers.^22^ The addition of excess DMAP to a benzene-*d*_6_ solution of **Ti-1** produced an immediate color change from forest green to dark purple and shifted resonances of both parent **Ti-1** and free DMAP, signaling formation of [(*Ar*)^t^BuN]_3_Ti­(DMAP) (**Ti-2**). This paramagnetic product was characterized by X-ray diffraction and confirmed the formation of **Ti-2**, which exhibited a distorted tetrahedral structure (Figure 1C, Ti(1)–N(1) = 2.2002(14) Å). Analogous titanium complexes were prepared from 4-OMe-pyridine (**Ti-3**; Ti(1)–N(1) = 2.193(2) Å) and 4-^*t*^Bu-pyridine (**Ti-4**; Ti(1)–N(1) = 2.180(3) Å) (Figure 1D). Progressively shorter Ti–N_pyr_ bond lengths were observed with less electron-donating pyridines, likely as a result of increased degree of reduction of the pyridine ring and increased covalency of the Ti–N bond. In the case of the DMAP example **Ti-2**, the Ti(1)–N(1) bond is relatively long and more consistent with a titanium-centered SOMO, which is supported by electron paramagnetic resonance (EPR) spectroscopy (see Supporting Information). Similar behavior has been reported by Wolczanski and coworkers with tris­(siloxide) titanium pyridine complexes.^23^ Given the straightforward and quantitative formation of **Ti-2**, which contains the same pyridine ligand (DMAP) as the phenoxy­(imine)–nickel­(II)–aryl complexes, this specific titanium derivative was used for subsequent C­(sp^3^)-radical generation studies.

### Reactivity of Phenoxy­(imine)–Nickel Complexes with In Situ Generated C­(sp^3^)-Derived Radicals

A control experiment was conducted whereby a benzene-*d*_6_ solution of **Ni-1** and **Ti-2** (generated in situ with 4 equiv of DMAP) was allowed to stand at ambient temperature (Scheme 4). No change in either complex was detected by NMR spectroscopy over the course of 12 h. Addition of the alkyl bromide **1** resulted in complete consumption of **Ni-1** and a quantitative yield of the C–C bond-formation product **2** in less than 5 min. The metal-containing products were identified as the bis­(chelate) nickel­(II) complex (FI)_2_Ni­(DMAP)_2_ (**Ni-2**) and [(*Ar*)^t^BuN]_3_TiBr (**Ti-5**), based on NMR spectroscopy and single crystal X-ray diffraction (see Supporting Information). These observations demonstrate that **Ti-2** is a selective reagent for the generation of alkyl radicals from unactivated alkyl bromides.

**Scheme 4:**
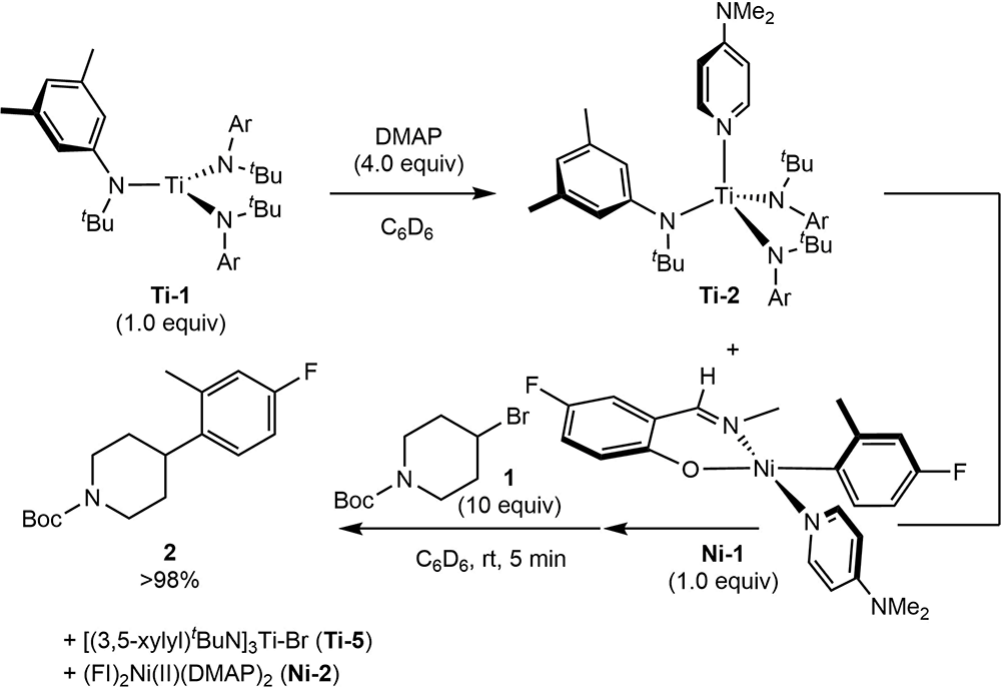
In Situ Radical Generation with Ti­(III) Reagents and Radical Coupling with **Ni-1**

The application of **Ti-2** for the generation of an array of alkyl radicals and subsequent C–C bond formation from **Ni-1** was assayed (Scheme 5). Addition of the piperidine alkyl bromide (**1**) or iodide (**3**) to a benzene-*d*_6_ solution containing 1.2 equiv of **Ti-2** and 1.0 equiv of **Ni-1** resulted in formation of the corresponding alkylated arene in high yields. The N-Boc piperidine alkyl chloride (**4**) and redox-active ester (**5**), known to be challenging substrates in nickel-catalyzed cross-coupling,^24^ performed effectively under these conditions. Methyl iodide (**6**) was used to generate methyl radicals and resulted in the rapid methylation of the nickel aryl. In contrast, acyclic secondary alkyl bromides (**7**) and benzylic alkyl bromides (**8**) furnished the corresponding arylation products in moderate yields, while tertiary alkyl halides (**10**) or activated α-carbonyl halides (**9**) were ineffective for C–C bond formation. In these cases, the alkyl halide was completely consumed with concomitant formation of the expected titanium­(IV) product **Ti-5**, but **Ni-1** remained intact. These observations mirror the nickel-catalyzed C­(sp^2^)–C­(sp^3^) Suzuki–Miyaura cross-coupling and imply that the reduced yields with acyclic alkyl bromides and the lack of product observed with tertiary alkyl electrophiles are the result of inefficient or ineffective radical capture by nickel­(II). Importantly, the titanium method of radical generation provides a reliable probe for the C–C bond-forming step from phenoxy­(imine)–nickel­(II)–aryl complexes.

**Scheme 5:**
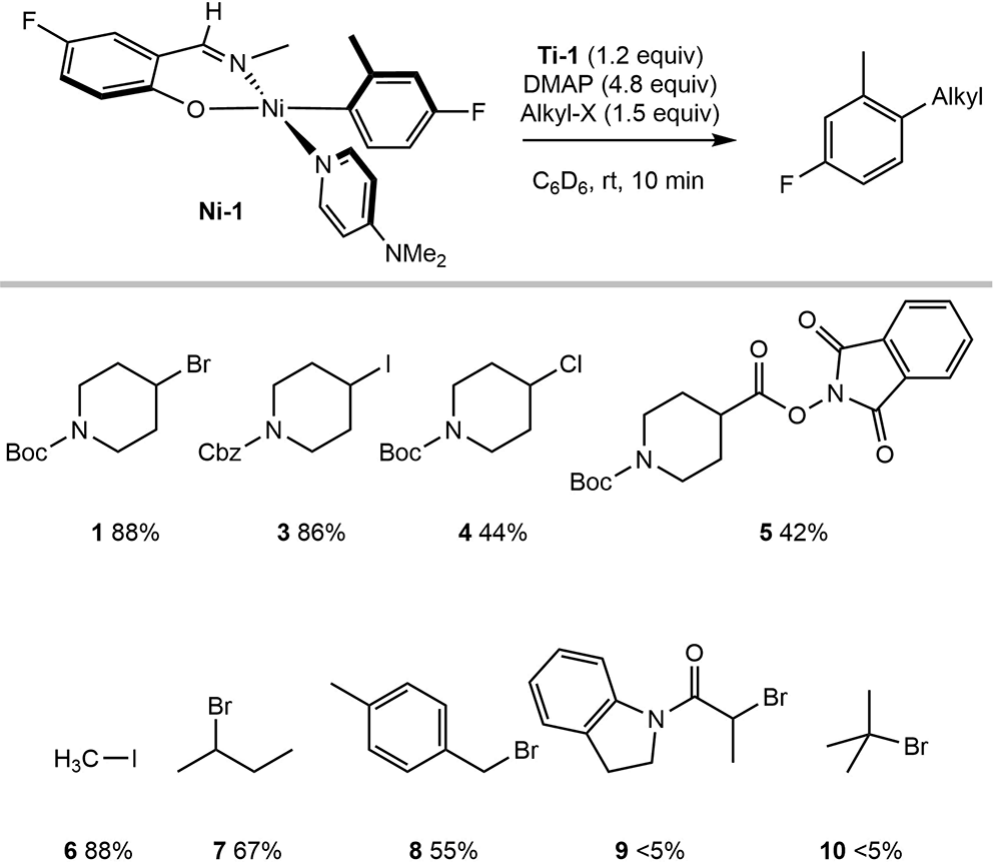
Scope of C–C Bond Formation from Addition of Alkyl Halides to a Mixture of **Ti-2** and **Ni-1** in Benzene-*d*_6_

To further explore the kinetics and thermodynamics of radical coupling with the phenoxy­(imine)–nickel­(II)–aryl complex, 5-hexenyl bromide was utilized as the alkyl halide precursor to generate a known radical clock. Ingold and coworkers have reported detailed kinetic data for the 5-exo-trig cyclization of the 5-hexenyl radical,^25^ enabling the use of the ratio of cyclized to acyclic products to report on the radical coupling time scale. Radical generation and capture experiments were conducted using 1 equiv of **Ni-1**, 0.1 equiv of **Ti-1**, 0.4 equiv of DMAP, and 0.1 equiv of hexenyl bromide, with in situ generation of **Ti-2**. The reaction was intentionally designed to produce low conversion of **Ni-1** such that radical coupling with the phenoxy­(imine)–nickel complex is under pseudo-first-order conditions. Quantitative analysis of the radical-clock outcome also relies on conditions under which only two competitive radical pathways operate: (i) direct coupling with the initially formed linear alkyl radical by **Ni-1** and (ii) radical rearrangement and subsequent capture of the cyclized alkyl radical by **Ni-1**. Control experiments were conducted to validate these assumptions and are presented below.

Treatment of mixtures of 0.1 equiv of **Ti-1** and 1.0 equiv of **Ni-1** in benzene-*d*_6_ with varying (0.4 – 1.6) equivalents of DMAP (Scheme 6A) resulted in no detectable change in the ratio of linear (**11**) to cyclized (**12**) radical coupling products (**11**/**12** = 4.0). These results establish that the dissociation or coordination of additional DMAP to the nickel is not significant during the radical capture and C–C bond-forming sequence. Experiments were also performed using 0.1 to 0.3 equiv of **Ti-2** relative to **Ni-1**, which produced an essentially invariant product ratio (**11**/**12**) from 3.8 to 4.3, suggesting that alkyl radical capture by the titanium­(III) complex is insignificant and that the observed product ratios are reflective of radical capture and C–C bond formation by the nickel complex, which is the resting state in the catalytic Suzuki–Miyaura coupling.

**Scheme 6:**
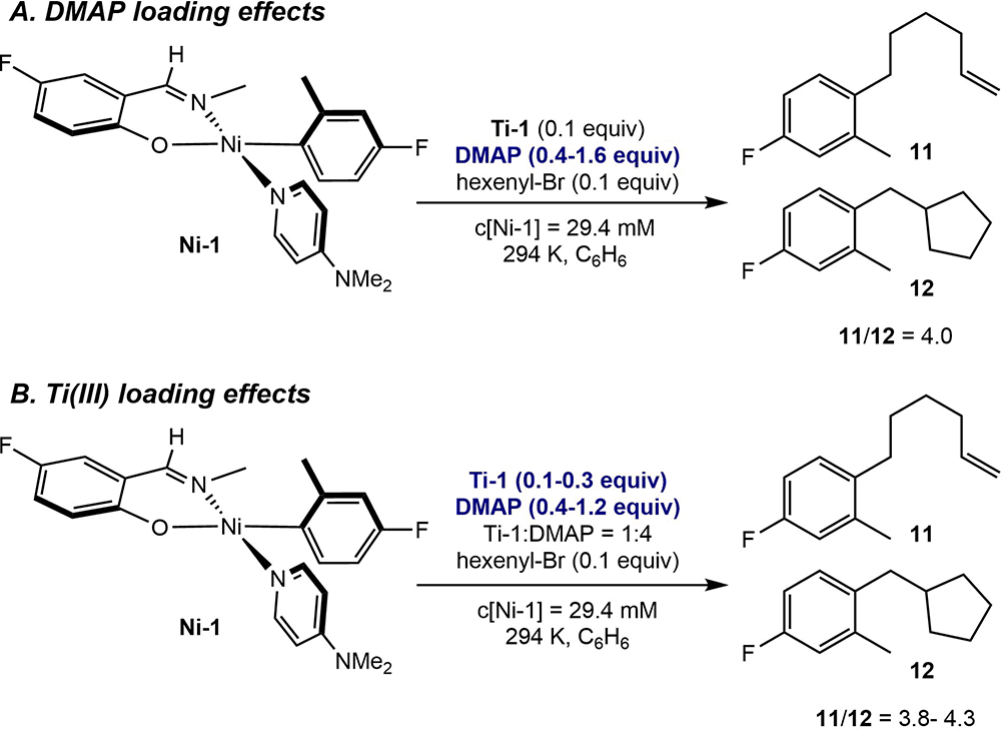
Radical Clock Experiments Exploring the Influence of the Equivalents of DMAP and **Ti-2** on the Product Distribution, **11**/**12**

The linear/cyclized product ratio (**11**/**12**) as a function of the concentration of **Ni-1** was also studied (Scheme 7). The ratio of **11** and **12** was proportional to the concentration of **Ni-1**, consistent with trends observed in catalytic reactions in our previous study.^8^ These results enabled estimation of a radical coupling rate constant of *k*_obs_ = 2.6 × 10^7^ M^–1^·s^–1^ at 294 K, corresponding to a free energy barrier of 7.2 kcal/mol.

**Scheme 7:**
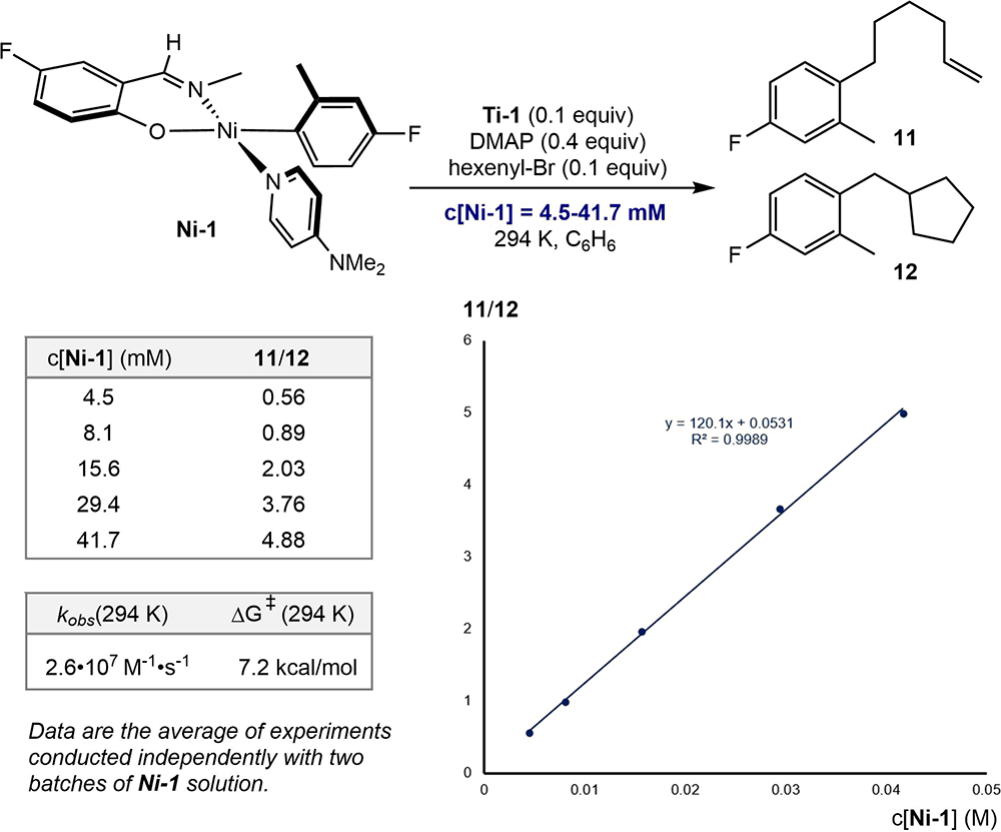
Influence of the Concentration of **Ni-1** on the Ratio of **11**/**12**

Conducting the experiments at different temperatures in toluene resulted in measurable changes in the product ratios (Scheme 8). Using the known kinetic parameters for 5-hexenyl-radical cyclization, temperature-dependent rate constants were extracted for radical coupling.^25^ These data provided a complete kinetic and thermodynamic description of the C–C bond-forming step from Eyring analysis, yielding a calculated enthalpy change (Δ*H* ^‡^) of 2.5(2) kcal/mol and an entropy of activation (Δ*S* ^‡^) of −16(1) cal·mol^–1^·K^–1^, consistent with a bimolecular reaction.

**Scheme 8:**
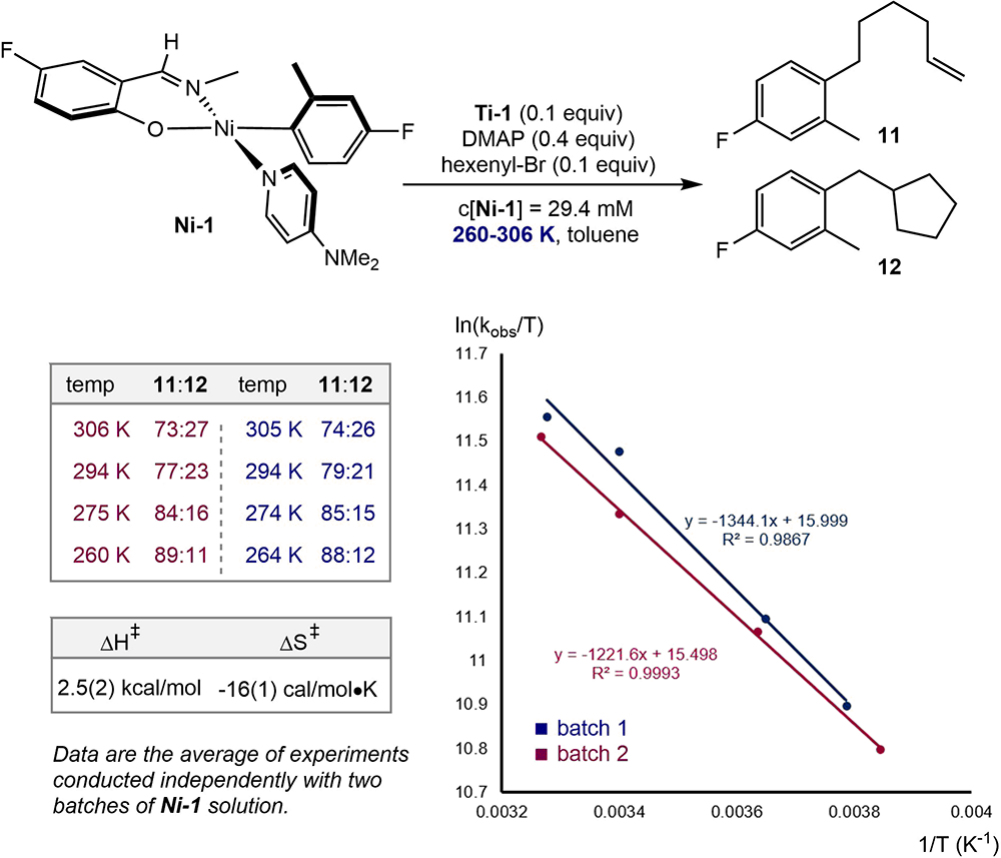
Temperature Effects on the Ratio of **11**/**12** from Radical Capture Experiments with **Ni-1**

To investigate the effects of the substitution on the phenoxy­(imine) ligand in the planar nickel aryl complex on radical capture, experiments were conducted with the 5-hexenyl radical clock with different nickel complexes in parallel (different flasks) and in competition (same flask). Because these two experiments probe not necessarily equivalent aspects of radical coupling, both were employed to avoid overinterpretation of a single mechanistic probe. In the first series of experiments (Scheme 9A), stock solutions of different nickel complexes were prepared at the same concentration, followed by the addition of 0.2 equiv of **Ti-1**, 0.8 equiv of DMAP to generate **Ti-2** in situ, and 0.1 equiv of hexenyl bromide. This procedure led to formation of a mixture of linear (**l**) and cyclic (**c**) arylation products under pseudo-first-order conditions. The linear-to-cyclic product ratio (**l**:**c**) provides the relative rate of radical coupling with different phenoxy­(imine)–nickel­(II)–aryl complexes. In the second series of experiments (Scheme 9B), two different phenoxy­(imine)–nickel­(II)–aryl complexes were combined in the same flask (competition), which was then treated with the same amount of **Ti-1**, DMAP, and hexenyl bromide. Analysis of the corresponding combined linear and cyclic product ratio with different nickel aryl complexes (Scheme 9B, product groups a and b) provided the relative rate of C–C bond formation between the two nickel complexes. Of note, independent control experiments confirmed that aryl exchange between nickel complexes was negligible under these conditions (see Supporting Information).

**Scheme 9:**
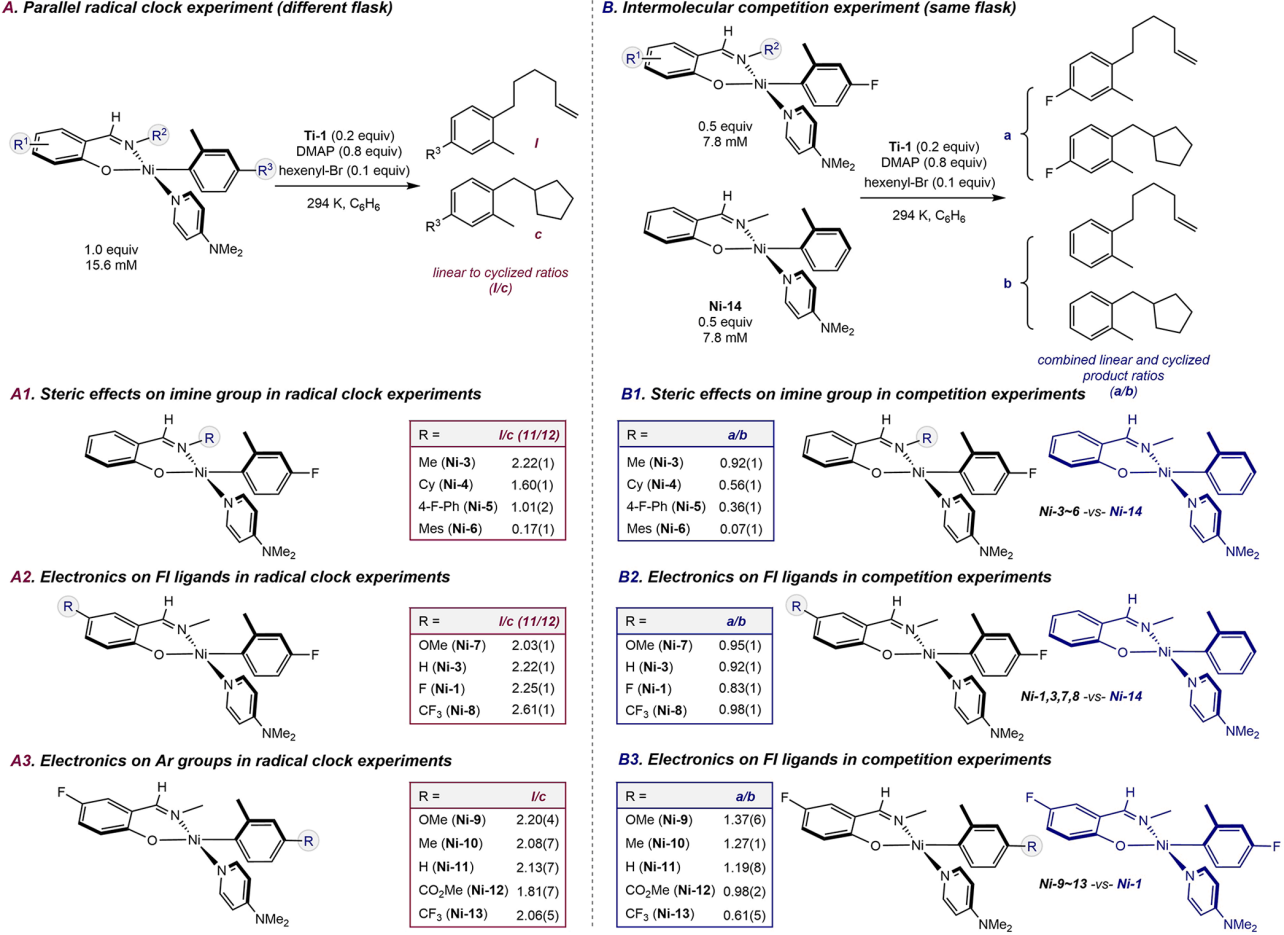
Radical Clock Experiments Conducted in Parallel (Different Flasks) and Intermolecular Competition Experiments Conducted in Single Flask with Different Phenoxy­(imine)–Nickel­(II)–Aryl Complexes

N-Imine substituent effects in the phenoxy­(imine)–nickel­(II)–aryl complexes were first examined. In parallel radical clock experiments (Scheme 9A1), the more sterically hindered groups resulted in a decreased ratio of **l**:**c**, ranging from 2.22(1) to 0.17(1) when the N-imine position was substituted with methyl (**Ni-3**), cyclohexyl (**Ni-4**), 4-fluorophenyl (**Ni-5**), and mesityl (**Ni-6**). Results from intermolecular competition experiments show a similar trend (Scheme 9B1). The ratio of the combined amount of linear and cyclic products derived from a given nickel aryl complex in reference to competition standard complex **Ni-14** was observed to decrease from 0.92(1) to 0.07(1) when the size of the N-imine group increased from methyl to mesityl.

Altering the electronic properties of the 4-aryl substituent on the phenoxy­(imine) ligands resulted in a slight variation in the linear-to-cyclic product ratio (**l**:**c**) (Scheme 9A2). Changing this group from methoxy (**Ni-7**) to hydrogen (**Ni-3**), fluorine (**Ni-1**), and trifluoromethyl (**Ni-8**) substituents resulted in a modest increase in the linear-to-cyclic product ratio from 2.03(1) to 2.61(1). Notably, this effect was not observed in the corresponding competition experiments, with no obvious trends in product distribution observed across the same series of electronically differentiated phenoxy­(imine) ligands (Scheme 9B2).

Electronic effects of the aryl groups in the phenoxy­(imine)–nickel­(II)–aryl complexes were also evaluated. In parallel radical clock experiments, the linear-to-cyclic product ratio produced no obvious trend (Scheme 9A3). Only a minor variance in the **l**:**c** ratio from 1.81(7) to 2.20(4) was observed, with no correlation to the electron-donating or -withdrawing nature of the substituent. In contrast, when the radical coupling was conducted in competition, a clear trend was observed, where aryl groups with electron-donating substituents converted preferentially versus those with electron-withdrawing groups, with the product distribution ratio (a:b) ranging from 1.37(6) to 0.61(5) (Scheme 9B3). The highest product formation ratio was observed when the aryl group was substituted with 4-methoxy (**Ni-9**) and the lowest ratio was observed with the 4-trifluoromethyl substituent (**Ni-13**). Taken together, the results of these experiments suggest that less sterically hindered and more electron-deficient ligands or more electron-rich aryl groups on nickel lead to acceleration of radical coupling. We note that the observed substituent effects may also be a result of variances in *trans* effects that would modulate the basicity of the nickel and alter the energy of the $d_{z^{2}}$ orbital. Notably, the results from separate flask radical clock experiments are not entirely consistent with the same-flask competition experiments.

Because single-step radical capture from nickel­(II) aryl complexes has been proposed based on experimental studies and data science analysis with radical clocks,^12,14^ a kinetic model based on the experimental data obtained in this study was developed and is presented in Figure 2. If such a pathway was operative, the quotient of the linear-to-cyclic product ratios for two nickel complexes would match the corresponding product-distribution ratio measured in an intermolecular competition experiment. Accordingly, both values should equal the quotient of radical coupling rate constants (*k*_1_/*k*_2_) under initial-rate conditions, and a linear relationship is expected (Figure 2B, red diagonal line). While an overall positive correlation was observed in the two (parallel versus intermolecular competition) data sets, the distribution of the data was generally scattered. Specifically, the data corresponding to the electronic variations in the phenoxy­(imine) ligands (Figure 2B, red diamonds) or aryl groups (Figure 2B, blue triangles) are not linear and therefore are inconsistent with a pathway involving a single step for the radical coupling responsible for C–C bond formation. Rather, the data are most consistent with a multistep process composed of different elementary steps that impact the reaction rate and product selectivity. Kinetic selectivities obtained from parallel reactions and intermolecular competition experiments are not necessarily equivalent under complex kinetic scenarios. This distinction has been thoroughly discussed in studies of kinetic isotope effects in transition-metal-catalyzed C–H functionalization.^26^ In analogy, trends in radical coupling inferred from parallel radical clock experiments versus intermolecular competition experiments likely report on different elementary steps within the overall multistep kinetic process (for alternative proposals on mechanistic possibilities, see Supporting Information).

**Figure 2:**
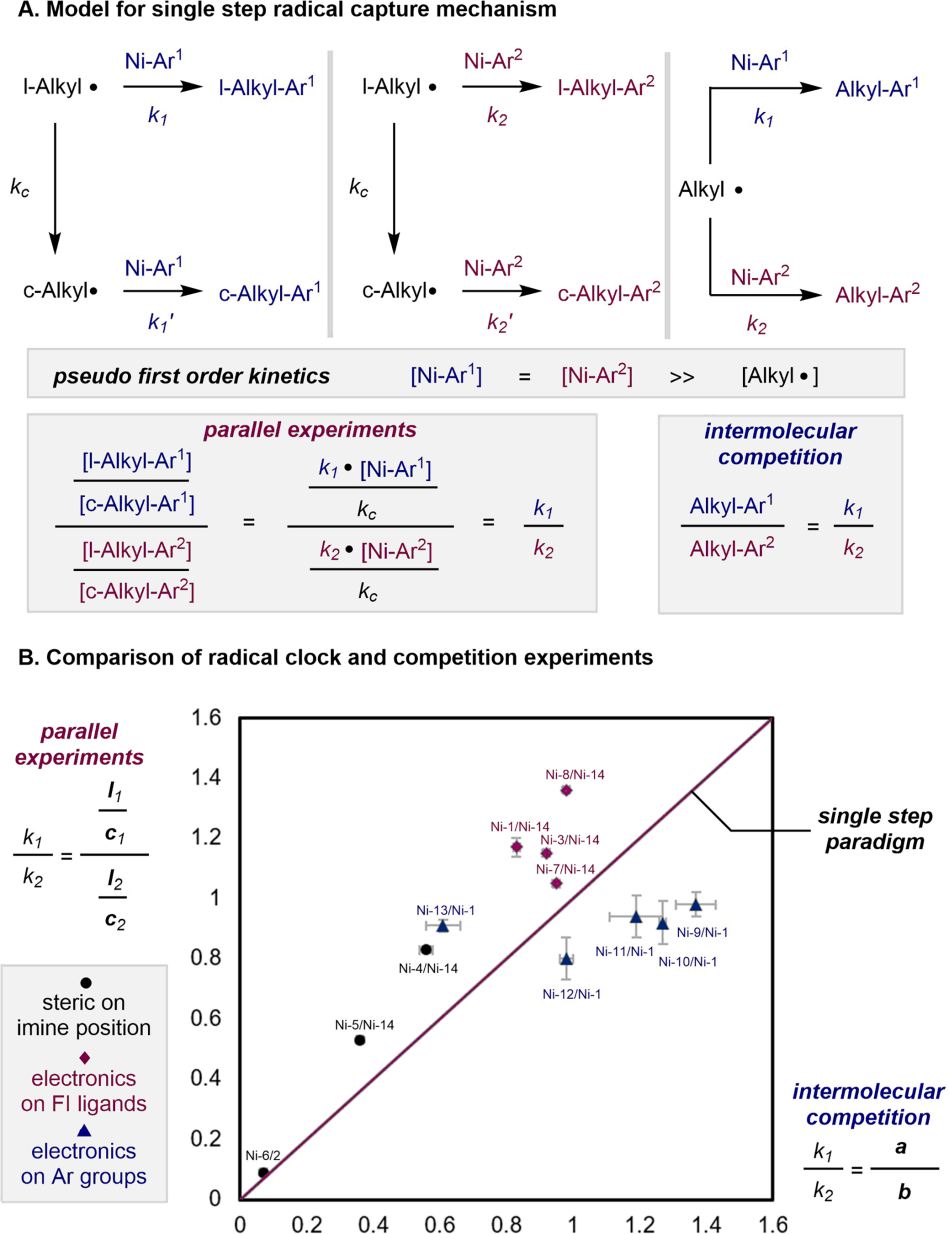
A. Kinetic model for a single-step radical capture pathway. B. Comparison of the relative radical coupling rate determined by radical clock experiments (*y*-axis) and by intermolecular competition experiments (*x*-axis).

Additional studies were conducted to interrogate a single-step versus multistep radical coupling pathway. Natural abundance ^12^C/^13^C kinetic isotope effects (KIE)^27^ were measured for the ipso carbon of the Ni–C_aryl_ bond. Quantitation was conducted by NMR spectroscopy by analyzing the arylated product generated from an experiment utilizing 0.15 equiv of **Ti-1**, 0.6 equiv of DMAP, and 0.15 equiv of alkyl halide to enable precise control of the conversion of the reaction. To minimize potential errors arising from a ^12^C/^13^C KIE during the synthesis of **Ni-1**, a separate reaction was carried out to full conversion as a reference (Scheme 10). A value of 1.004(7) was measured for the product derived from the Ni–C_aryl_ bond in **Ni-1**. If a single-step radical capture pathway was operative, direct C–C bond formation would be involved in the rate-determining step, and thus a measurable primary ^12^C/^13^C KIE at C1 would be expected. In a multistep pathway, a negligible KIE is consistent with a multiple-step pathway involving radical capture at the nickel­(II) center, followed by reductive elimination that does not dominate the overall reaction kinetics.

**Scheme 10:**
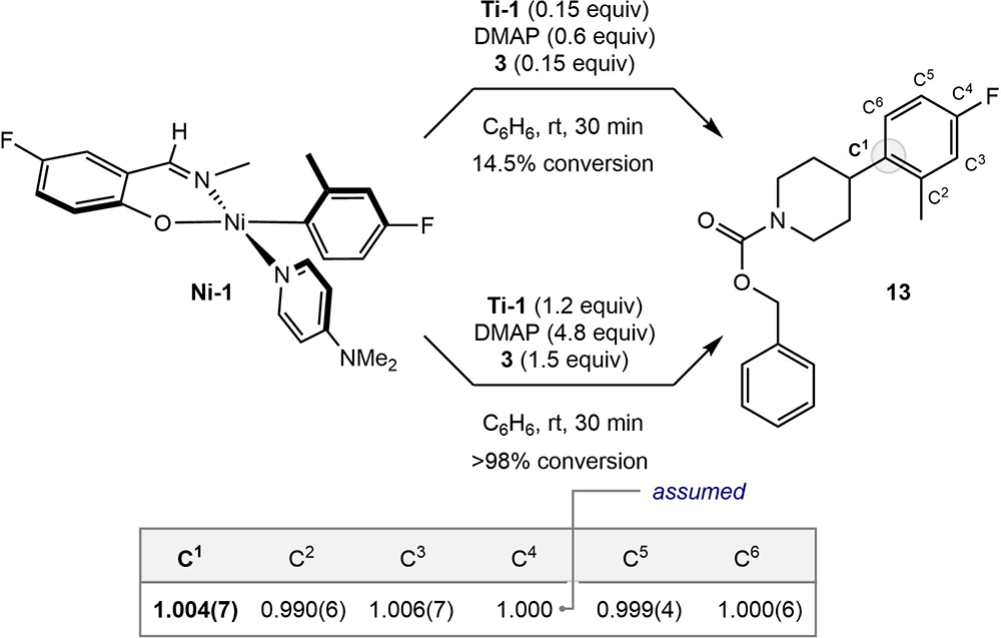
^12^C/^13^C Kinetic Isotope Effects (KIE) for Radical Coupling with **Ni-1**

A multistep pathway for radical coupling with phenoxy­(imine)–nickel­(II)–aryl complexes would likely involve the formation of a short-lived Ni­(III) intermediate. However, attempts to detect Ni­(III) intermediates during the radical coupling reaction by freeze-quench EPR spectroscopy have been unsuccessful (Scheme 11). In a typical experiment, the alkyl halide was added to a thawing toluene solution of **Ni-1**, **Ti-1**, and excess DMAP. The mixture was rapidly glassed at 77 K, and the EPR spectrum was recorded. The sample was then thawed for approximately 1 min and refrozen, and measurements were repeated until the signal for **Ti-2** disappeared. Although a clean decay of the **Ti-2** signal was observed as a function of time, attempts to identify new spectroscopic features corresponding to putative Ni­(III) or Ni­(I) intermediates were unsuccessful. Moreover, the decay of **Ti-2** signals on the minute time scale suggested that halide abstraction for radical generation is much slower than subsequent radical coupling with **Ni-1**. Such a scenario could plausibly contribute to the lack of observable nickel­(III) signals, as the downstream radical capture event occurs too rapidly to allow experimental observation.

**Scheme 11:**
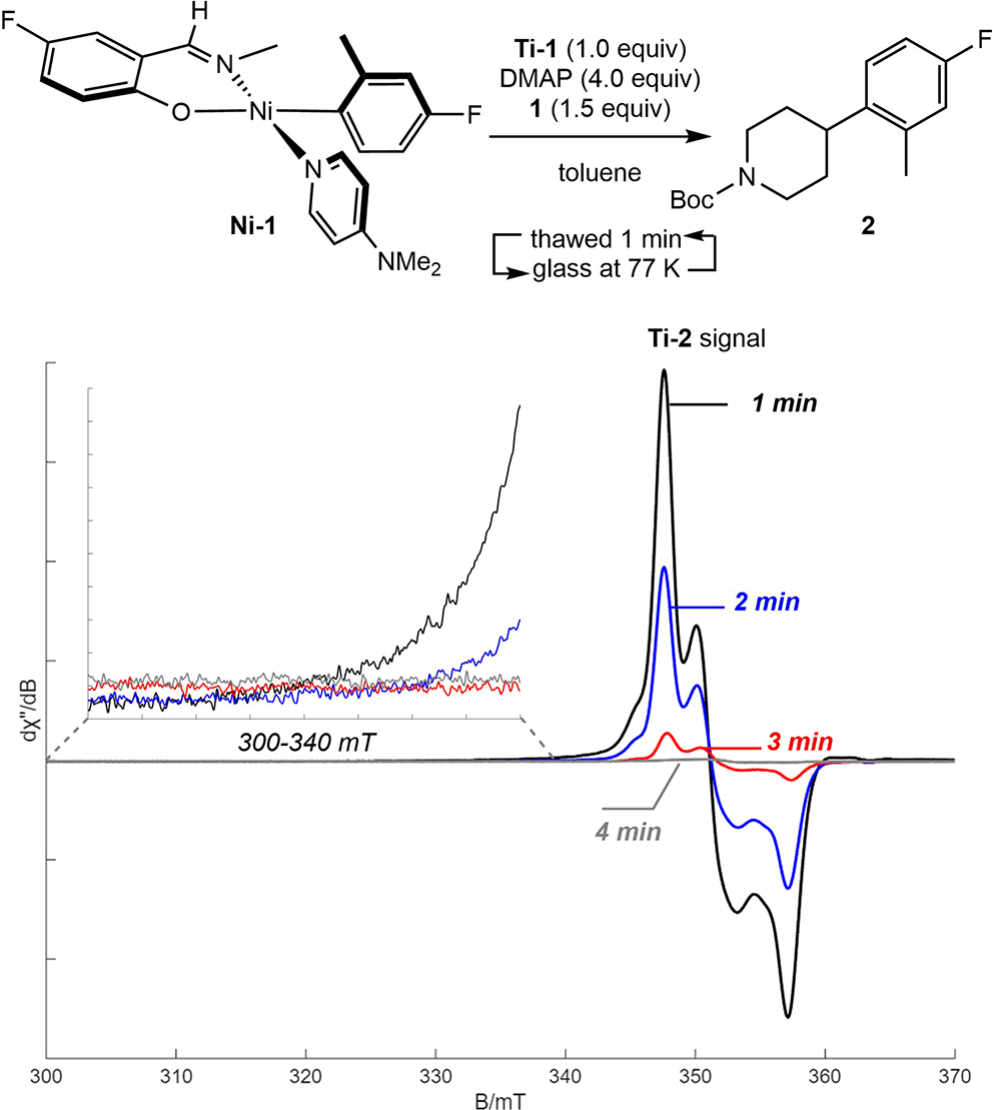
Radical Coupling Reaction Time Course Monitored by Freeze-Quench X-Band EPR Spectroscopy

To probe the viability of a nickel­(III) intermediate in C–C bond formation, independent syntheses were attempted that circumvented slow radical generation (Scheme 12A). Treatment of **Ni-1** with tris­(4-bromophenyl)­ammoniumyl hexachloroantimonate (“magic blue”, **14**) resulted in the rapid formation of a dark red solution, and analysis of the resulting glass by X-band EPR spectroscopy at 77 K produced an axial signal, tentatively assigned as the cationic nickel­(III) complex, **Ni-15**. Repeating the procedure using *N*-bromosuccinimide (NBS) as the oxidant produced a more complex EPR signal, assigned as a mixture of a butyronitrile-coordinated Ni­(III) ion pair (**Ni-16**) and phenoxy­(imine)–nickel­(III)–bromide derivative (**Ni-17**).^28^ Both of these nickel­(III) complexes were short-lived, persisting for <1 min at room temperature. In the case of oxidation with NBS, aryl bromide **15** was formed in 76% yield upon heating the reaction mixture to room temperature.

**Scheme 12:**
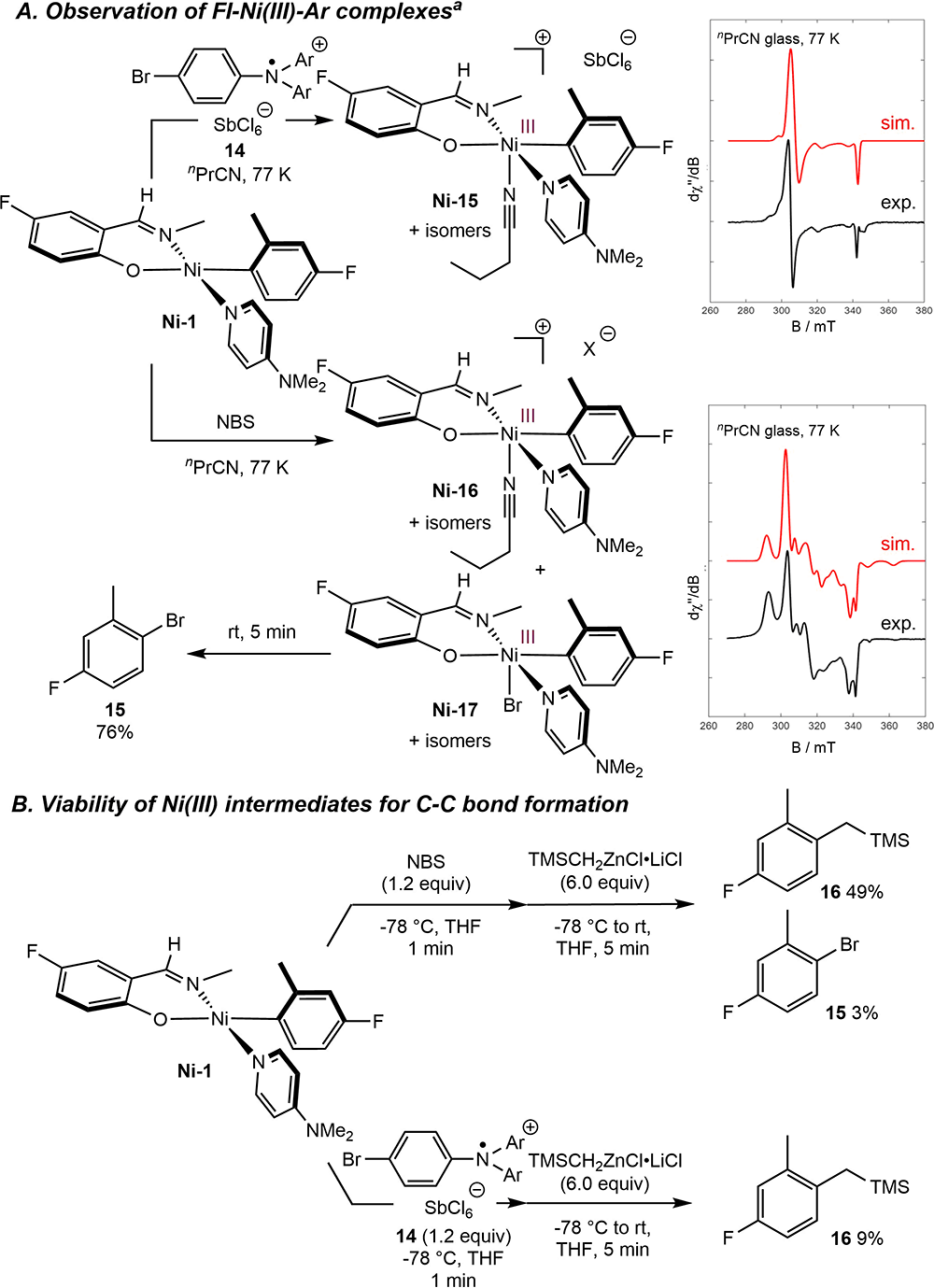
Spectroscopic Observation of Phenoxy­(imine)–Nickel­(III) Complexes and Viability of these Intermediates in C­(sp^2^)–C­(sp^3^) Bond Formation

a EPR spectra were collected at 77 K in butyronitrile glass. Experimental parameters: microwave frequency = 9.64 GHz, power = 0.3162 mW, and modulation amplitude = 4.000 G. Simulation parameters:*S*(1) **Ni-15** tentatively assigned as a mixture of two isomers: *S*(1) = 1/2, *g*_123_(1) = (2.26, 2.24, 2.01), *g*_strain_(1) = (0.03, 0.03, 0.01); *S*(2) = 1/2, *g*_12*3*_(2) = (2.31, 2.15, 2.04), *g*_strain_(2) = (0.03, 0.03, 0.03). **Ni-16** tentatively assigned as *S*(3)= 1/2, *g*_123_(3) = (2.28, 2.26, 2.02), *g*_strain_(3) = (0.03, 0.03, 0.01); **Ni-17** tentatively assigned as a mixture of two isomers: *S*(4)= 1/2, *g*_123_(4) = (2.35, 2.18, 2.02), *A*_123_(^79^Br) = (0, 0, 170 MHz), *g*_strain_(4) = (0.03, 0.03, 0.01); *S*(5)= 1/2, *g*_123_(5) = (2.24, 2.14, 2.04), *A*_123_(^79^Br) = (0, 0, 170 MHz), *g*_*strain*_(5) = (0.02, 0.03, 0.02) (see Supporting Information for additional details).

Formation of **15** likely occurs by C–Br reductive elimination from **Ni-17**.^28f,29^ To trap this intermediate, oxidation was performed at −78 °C in THF for 1 min, followed by the addition of excess (Me_3_SiCH_2_)­ZnCl·LiCl (Scheme 12B). An immediate color change from dark red to orange–yellow was observed upon the addition of the organozinc at −78 °C. After warming the reaction to ambient temperature, the C–C bond formation product **16** along with C–Br bond formation product **15** were observed in 49% and 3% yield, respectively. Replacing NBS with **14** and repeating the proposed transmetalation reaction with the alkyl zinc resulted in generation of **16** in 9% yield. Reaction between **Ni-1** and (Me_3_SiCH_2_)­ZnCl·LiCl in the absence of an oxidant furnished <2% of **16**. These results demonstrate that phenoxy­(imine)–nickel­(III) complexes can be generated in solution and intercepted as viable intermediates in C­(sp^2^)–C­(sp^3^) bond formation.^28e,28f^

DFT calculations were also performed to provide additional mechanistic insights into the radical coupling process using **Ni-3** and the ethyl radical as a representative C­(sp^3^) partner (Figure 3). The association of an ethyl radical with the square planar ground state of **Ni-3** (**GS-1**) was calculated to be endergonic (2.6 kcal/mol) and forms the putative Ni­(III)–alkyl–aryl intermediate, **GS-2**. Geometry optimization of this intermediate supports a square-pyramidal structure with an *S* = 1/2 ground state. Intermediates with additional DMAP coordination (**GS-3**) or a higher-spin state (**GS-4**, *S* = 3/2) were energetically less favorable. Attempts to locate a direct reductive elimination transition state from **GS-2** were unsuccessful due to conformational constraints, and isomerization of **GS-1** is thus required for productive C–C bond formation. This pathway is similar to the computational report from Molander, Gutierrez et al. on radical coupling with bipyridine nickel­(II)–aryl complexes.^30^ Several possible isomerization pathways were evaluated (see Supporting Information), and among them, a pseudo-rotation of the alkyl substituent with a barrier of 7.5 kcal/mol (**TS-1**) was found to be the most energetically favorable.^31^ This isomerization leads to a slightly more stable intermediate, **GS-5**, that is poised to undergo reductive elimination to form the C–C bond with a free energy barrier of 10.1 kcal/mol. Notably, the barriers for **GS-5** conversion to **GS-2** by pseudo-rotation (**TS-1**, 9.8 kcal/mol) and **GS-5** conversion to **GS-6** by reductive elimination (**TS-2**, 10.1 kcal·mol) are similar. The computed ^12^C/^13^C KIE for the ipso carbon of the Ni–C_aryl_ bond was calculated to be 1.003 and 1.019 for **TS-1** and **TS-2**. Due to the contribution of **TS-1** in product formation, the apparent KIE of the overall C–C bond formation process should be between 1.003 and 1.019. Finally, the single-step alternative C–C bond formation pathway through **TS-3** was also computed and found to be energetically disfavored, with a barrier of 29.4 kcal/mol and a normal primary KIE of 1.034, which is inconsistent with the experimental results. Identifying a transition state for a concerted inner-sphere mechanism was unsuccessful, as all transition states featuring alkyl radical interaction with both the Ni center and aryl carbon converge to the corresponding nickel­(III) intermediates after intrinsic reaction coordinate (IRC) calculations. The computed energy surface provides a rationale for the trends observed in the ligand effects studies. For example, increasing the size of the N-imine substituent from methyl to 2,6-xylyl on the FI ligand raises the barrier of the pseudo-rotation step by 1.2 kcal/mol, resulting in less efficient C–C bond formation. Computational analysis of a more electron-rich phenoxy­(imine) ligand bearing a 4-methoxy substituent revealed similar barriers for both pseudo-rotation and reductive elimination. In contrast, a more electron-rich aryl group with a 4-methoxy substituent exhibited lower barriers for pseudo-rotation (**TS-1′**, 7.3 kcal·mol^–1^) and reductive elimination (**TS-2′**, 7.6 kcal·mol^–1^) (see Supporting Information for details).

**Figure 3:**
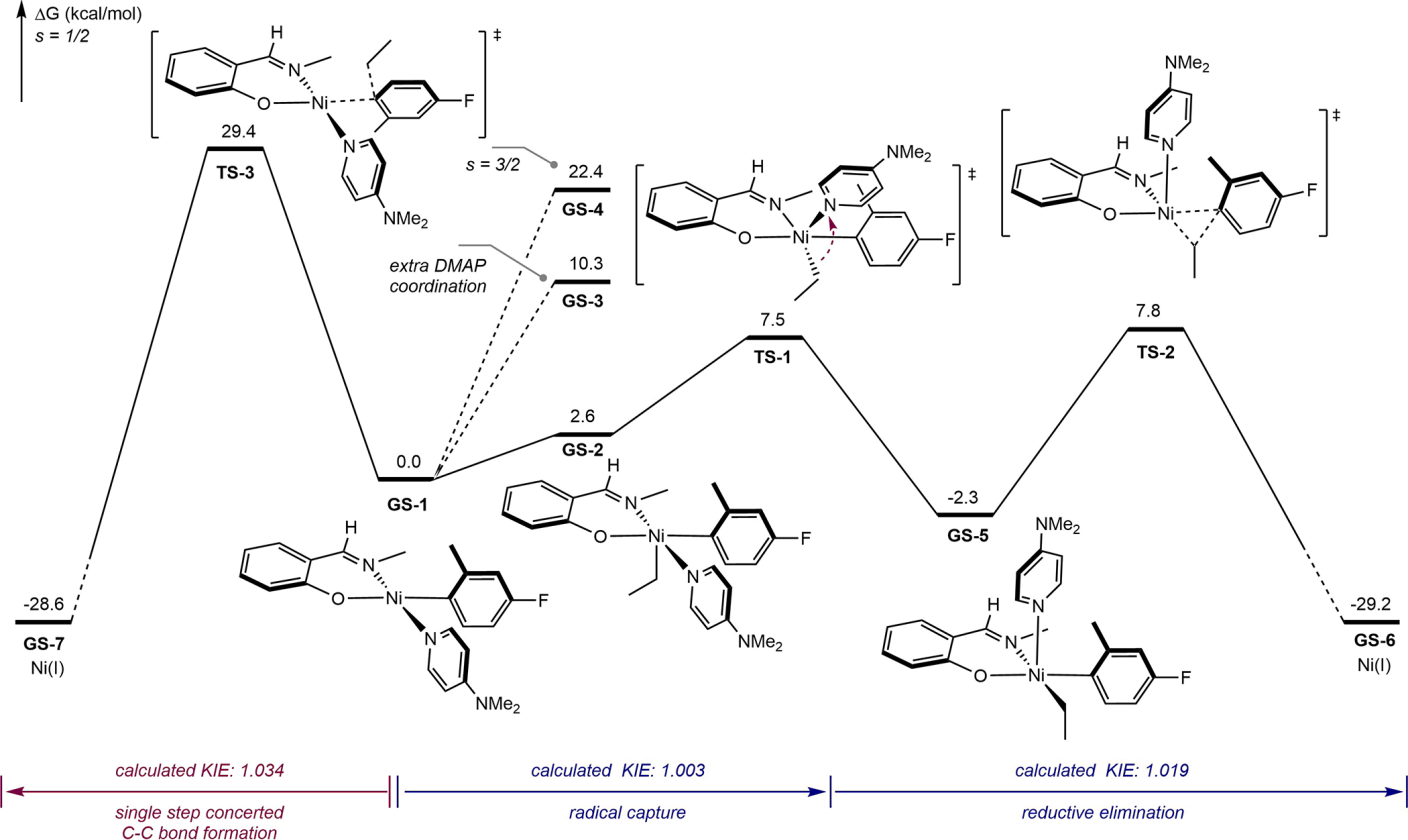
Investigation of radical capture with the FI–Ni–Ar complex through DFT calculations (TPSSh­(D4)/def2-TZVPP/SMD­(C_6_H_6_)//TPSS­(D3BJ)/def2-SVP).

Recently, Diao and coworkers proposed a concerted inner-sphere mechanism for alkyl radical capture by cationic nickel­(II)–aryl complexes supported by neutral tridentate ligands under photochemical conditions.^12b^ Systematic evaluation of trends and ligand effects established a volcano-type dependence for electronic effects on both the tridentate ligand and the aryl substituents, where sterically more demanding ligands resulted in slower radical capture. In work by Sevov and coworkers,^14^ electron-withdrawing ligands and electron-donating aryl groups were found to accelerate radical capture by neutral nickel­(II)–aryl complexes supported by monoanionic tridentate ligands. In our investigation of Ni­(II)–Ar complexes bearing monoanionic bidentate phenoxy­(imine) ligands, sterically less hindered, electron-withdrawing ligands and electron-donating aryl groups promote faster radical capture, as evidenced by radical clock and competition experiments. Additionally, the inconsistency of the radical clock and intermolecular competition experiments led us to recognize the limitations of radical clock experiments in probing complex kinetic scenarios, as interpretation of product ratios often relies on strong mechanistic assumptions.

## Conclusions

A combined experimental and computational study was conducted to investigate the mechanism of C–C bond formation from planar phenoxy­(imine)–nickel­(II)–aryl complexes and alkyl radicals. Such reactions constitute the key bond-forming step in nickel-catalyzed C­(sp^2^)–C­(sp^3^) Suzuki–Miyaura cross-coupling reactions between aryl boronic acids and alkyl bromides. Titanium­(III) reagents provided a reliable method for the generation of alkyl radicals from the corresponding alkyl halide, and stoichiometric studies identified radical capture as the origin of limitations in substrate scope with the electrophilic coupling partner. Substituent effects on both the supporting phenoxy­(imine) ligand and the nickel–aryl ligand were examined through competition experiments using the 5-hexenyl radical clock. Conducting these experiments in parallel or in competition furnished different linear-to-cyclic product ratios in certain instances, providing evidence for a multistep process for C­(sp^2^)–C­(sp^3^) bond formation. This conclusion was supported by ^12^C/^13^C kinetic isotope effects and computational studies. Eyring analysis and DFT results support a free energy of activation between 7 and 8 kcal/mol for the capture of primary alkyl radicals by the nickel­(II)–aryl complex at ambient temperature. The combined experimental and computational data are most consistent with a mechanism involving initial radical capture at the metal to form a nickel­(III) intermediate that undergoes isomerization and subsequent reductive elimination. These findings provide valuable insights for enhancing the reactivity in catalytic cross-coupling with challenging substrates through rational ligand design, an approach that is currently being actively pursued in our laboratory.

## Supplementary Material





## Notes

Additional experimental details and characterization data, including NMR, EPR spectra of new compounds; details on computational analysis (PDF)
XYX files of DMAP, **TS-1**–**TS-3**, **GS-1**–**GS-7** (ZIP)
