# Supplementary material for: Alkyl Radical Coupling with Phenoxy(imine)–Nickel(II)–Aryl Complexes: Evidence for a Multistep Process in C–C Bond Formation
Source: J Am Chem Soc. 2026 Apr 27;148(17):18231–42. doi: 10.1021/jacs.6c02912 (PMC13154193; doi:10.1021/jacs.6c02912)
Supplement: Supplementary file 1 [file ja6c02912_si_001.pdf]

Supporting Information

Alkyl Radical Coupling with Phenoxy(imine)-Nickel(II)-Aryl Complexes: Evidence for a Multistep Process for C–C Bond Formation

Hao Liang,[a] L. Reginald Mills,[a,c] Marina Perez-Jimenez,[a,d] Matthew V. Joannou,[b] Heejun Lee,[b] Steven R. Wisniewski,[b] Eric M. Simmons,[b] Karla Ravin,[a] and Paul J. Chirik[a]*

[a]Department of Chemistry, Frick Laboratory
Princeton University, Princeton, NJ 08544, U.S.A.
[b]Chemical Process Development, Bristol Myers Squibb Company
New Brunswick, NJ 08903, U.S.A.
[c]Department of Chemistry, University of Houston, Houston, TX 77204-5003, U.S.A.
[d]Department of Inorganic and Analytical Chemistry, University of Geneva, CH-1211 Geneva, Switzerland

*pchirik@princeton.edu

Table of Contents

I. General Considerations S2
II. Synthesis of (FI)Nickel Complexes........ S4
III. Experiments for Alkyl Radical Generation and Capture S31
IV Scope and Characterization of C-C Bond Formation Products S42
V Parallel Radical Clock Experiments S58
VI Intermolecular Competition Experiments S80
VII Comparison of Radical Clock and Competition Experiments S94
VIII $^{12}C/^{13}C$ Kinetic Isotope Effects S96
IV Synthesis and Interception of Ni(III) Complexes S103
X. Computational Data........ S115
XI. Kinetic Model for Multistep Radical Coupling S125
XII. Crystallographic Data S127
XIII. References S139

I. General Considerations

All air- and moisture-sensitive manipulations were carried out using vacuum line, Schlenk techniques or in an MBraun or Innovative Technology inert atmosphere (N_2) dry box unless otherwise noted. Glassware for air- and moisture-sensitive manipulations was oven-dried prior to use. The solvents used for air- and moisture-sensitive manipulations were dried and deoxygenated using literature procedures.[1] $Me^{R}FI$ ligands, $Na(Me^{R}FI)$ salts[2] and Ti(III)-tris-anilide complex[3] were prepared as previously described.

^{1}H NMR spectra were recorded on Bruker Avance 300, 400, or 500 spectrometers operating at 300 MHz, 400 MHz, and 500 MHz, respectively. ^{13}C NMR spectra were recorded on Bruker Avance 400 or 500 spectrometers operating at 101 MHz and 126 MHz, respectively. ^{19}F NMR were recorded on Bruker Avance 300 or 400 spectrometers operating at 282 MHz and 376 MHz, respectively. All ^{1}H and ^{13}C NMR chemical shifts are reported in ppm relative to $SiMe_4$, which was referenced using known chemical shifts of the solvent as internal standard. ^{1}H NMR data for diamagnetic compounds are reported as follows: chemical shift, multiplicity (s = singlet, d = doublet, t = triplet, q = quartet, pent = pentet, sept = septet, br = broad, m = multiplet, app = apparent, obsc = obscured), coupling constants (Hz), integration. ^{1}H NMR data for paramagnetic compounds are reported as follows: chemical shift, peak width at half height (Hz), integration. ^{13}C NMR data for diamagnetic compounds are reported as follows: chemical shift, multiplicity (if necessary).

Continuous wave EPR spectra were recorded at cryogenic temperatures on an X-band Bruker EMXPlus spectrometer equipped with an EMX standard resonator and a Bruker PremiumX microwave bridge, or on a Bruker EMXnano X-band spectrometer. The spectra were simulated using EasySpin for MATLAB.[4]

High-resolution mass spectra were obtained at Princeton University mass spectrometry facilities using an Agilent 6210 TOF LC/MS or Agilent 7250 GC QTOF with 8890 GC.

Single crystals suitable for X-ray diffraction were coated with polyisobutylene oil in the drybox, transferred to a nylon loop and then quickly transferred to either the goniometer head of a diffractometer equipped with a Bruker PHOTON III detector and Cu X-Ray tube (λ = 1.54178 Å) or a Rigaku XtaLAB Synergy-i equipped with a Mo X-ray tube (λ = 0.71073 Å) and a Cu X-ray tube (λ = 1.54178 Å). Preliminary data revealed the crystal system. The data collection strategy was optimized for completeness and redundancy using either the Bruker APEXII software suite or Rigaku CrysAlisPro software suite. The space group was identified, and the data were processed and corrected for absorption. The structures were solved using intrinsic phasing (SHELXT) and completed by subsequent Fourier synthesis and refined by full-matrix least-squares procedures in Olex2. Unless otherwise specified, hydrogen atoms were modelled as riding atoms. Constraints and restraints were utilized only to model disorder. For example, SADI commands were used to ensure relative bond lengths were similar, and EADP/SIMU/DELU commands were employed to constrain ADPs. In the case of structures where solvent molecules irrelevant to the organometallic compound of interest were co-crystallized, corresponding solvent electrons were masked with the SQUEEZE function.

II. Synthesis of (FI)Nickel Complexes

General Procedure A

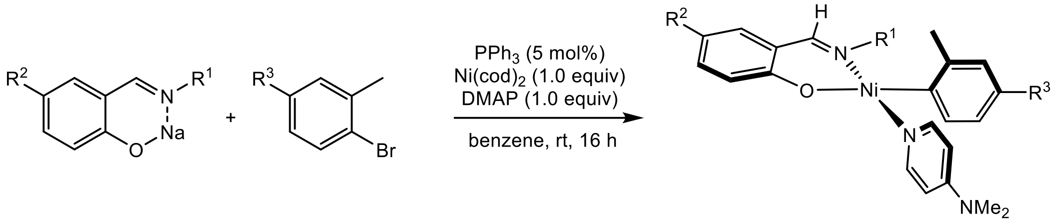

General Procedure B

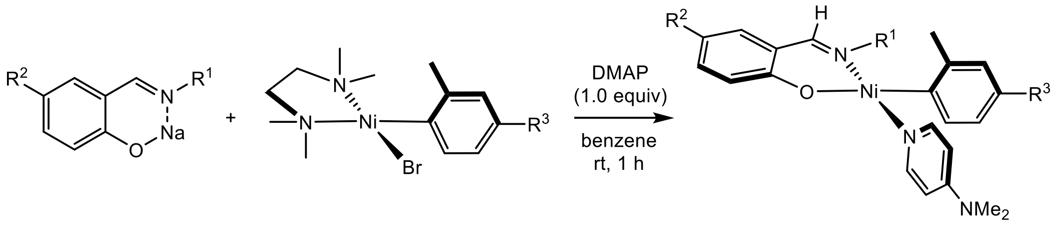

General Procedure A: In a N_2-filled glovebox, a 20-mL septum-capped vial with a stir bar was charged with Na(FI) (1.0 equiv), $Ni(COD)_2$ (1.0 equiv), DMAP (1.0 equiv), catalytic PPh_3 (5 mol%), and the appropriate aryl bromide (if solid) (1.0 equiv). Benzene was added (0.25 M), and the vial was capped, sealed with electrical tape, and removed from the glovebox. (If the aryl bromide was an oil, it was now added through the septum-cap by a syringe.) The solution was stirred at ambient temperature for 16 h. The vial was opened to air and filtered over Celite, and the Celite cake was washed with CH_2Cl_2 (2×10 mL) (note: most compounds were poorly soluble in benzene, so the CH_2Cl_2 wash was crucial for obtaining the isolated material in good yield). The organic filtrates were combined and concentrated under reduced pressure on a rotary evaporator with the water bath set to 45 ºC. After removal of the solvent, the concentrate was cooled to 0 ºC, at which time precipitation of the desired compound usually occurred. For compounds that did not immediately precipitate, the residue was triturated with hexanes to induce precipitation. The precipitate was collected in a fritted funnel and was washed with hexanes (1×20 mL) and Et_2O (1×10 mL), yielding the desired product.

General Procedure B: In a N_2-filled glovebox, a 20-mL septum-capped vial with a stir bar was charged with Na(FI) (1.0 equiv), (TMEDA)NiArBr (1.0 equiv), DMAP (1.0 equiv). Benzene was

added and the reaction was allowed to stir at room temperature for 1 hour. Upon completion, the mixture was filtered through a pad of celite and wash with benzene. The resulting solution was dried under vacuum to yield the desired product.

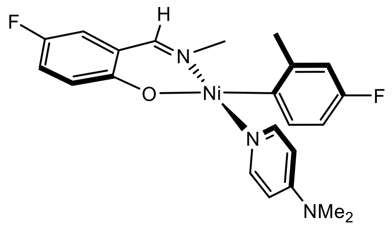

($Me^{F}FI$)Ni(2-Me-4-F-C_6H_3)(DMAP): Prepared according to General Procedure A in 2.5 mmol scale. The title compound was isolated as an orange-yellow solid (930 mg, 2.1 mmol, 84%). Purity of the title complex was determined to be 96% by ^{19}F NMR with C_6F_6 as internal standard. The analytical data were identical to literature.[2b] **^{1}H NMR** (500 MHz, C_6D_6, 25 ºC) δ_H 8.11 – 7.95 (m, 2H), 7.80 (t, *J* = 7.6 Hz, 1H), 6.96 – 6.90 (m, 2H), 6.89 – 6.81 (m, 2H), 6.75 (dd, *J* = 10.9, 2.8 Hz, 1H), 6.65 (dd, *J* = 9.1, 3.3 Hz, 1H), 5.57 – 5.33 (m, 2H), 3.27 (s, 3H), 2.29 (s, 3H), 1.89 (s, 6H).

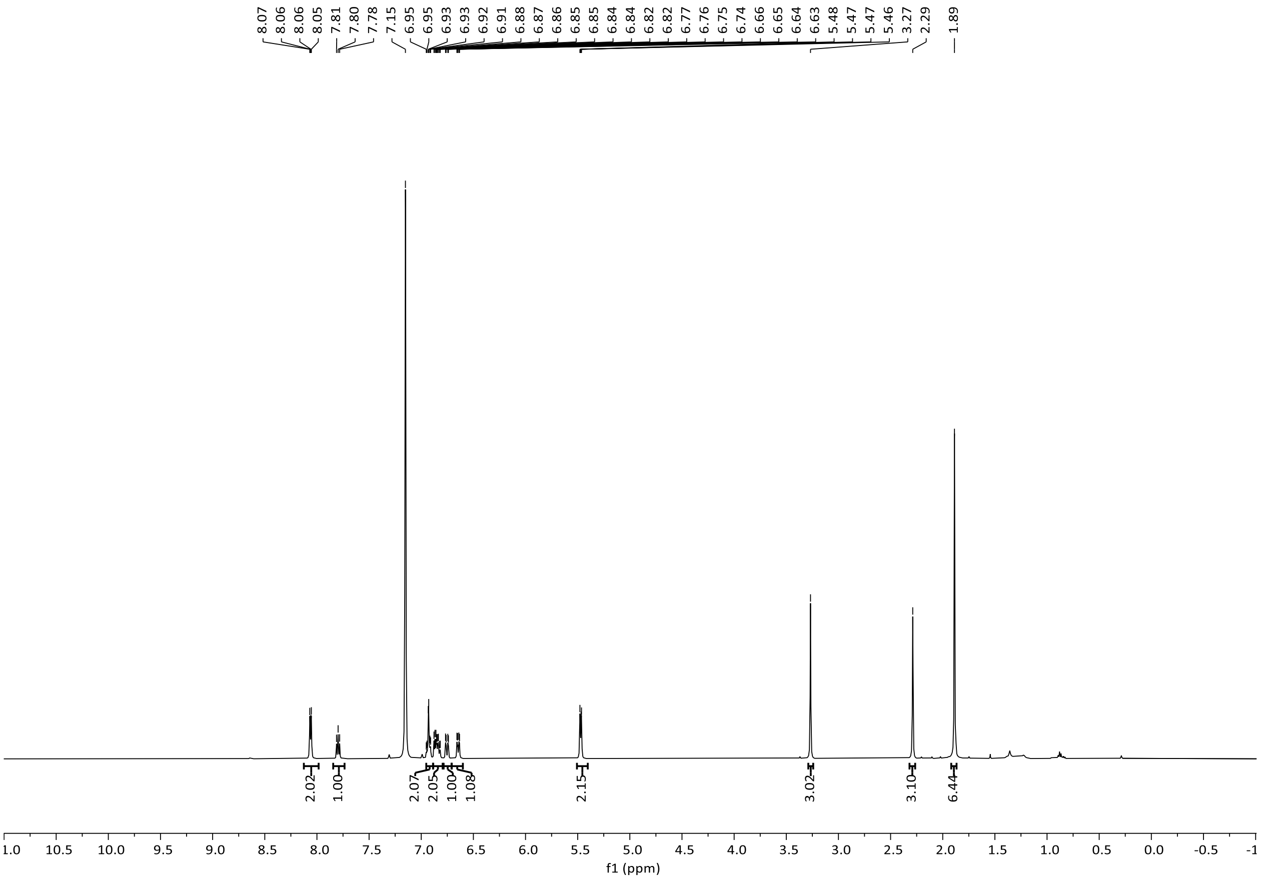

Figure S1. ^{1}H NMR spectrum (500 MHz, C_6D_6) of (Me^{F}FI)Ni(2-Me-4-F-C_6H_3)(DMAP).

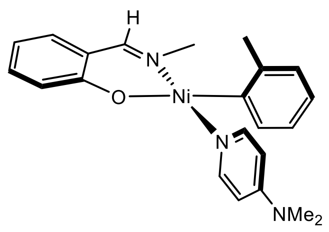

(MeFI)Ni(2-Me-C_6H_5)(DMAP): Prepared according to General Procedure A in 1.0 mmol scale. The title compound was isolated as a yellow solid (321 mg, 0.76 mmol, 76%). Purity of the title complex was determined to be 96% by ^{1}H NMR with 1,3,5-trimethoxybenzene as internal standard. The analytical data were identical to literature.[2b] **^{1}H NMR** (500 MHz, C_6D_6, 25 ºC) δ_H 8.22 – 8.16 (m, 2H), 8.15 – 8.06 (m, 1H), 7.26 – 7.20 (m, 2H), 7.13 (d, *J* = 8.7 Hz, 1H), 7.05 (ddd, *J* = 7.8, 5.7, 3.1 Hz, 1H), 6.99 (dd, *J* = 7.9, 1.9 Hz, 1H), 6.96 – 6.92 (m, 2H), 6.64 – 6.50 (m, 1H), 5.54 – 5.28 (m, 2H), 3.44 (s, 3H), 2.43 (d, *J* = 1.2 Hz, 3H), 1.88 (s, 6H).

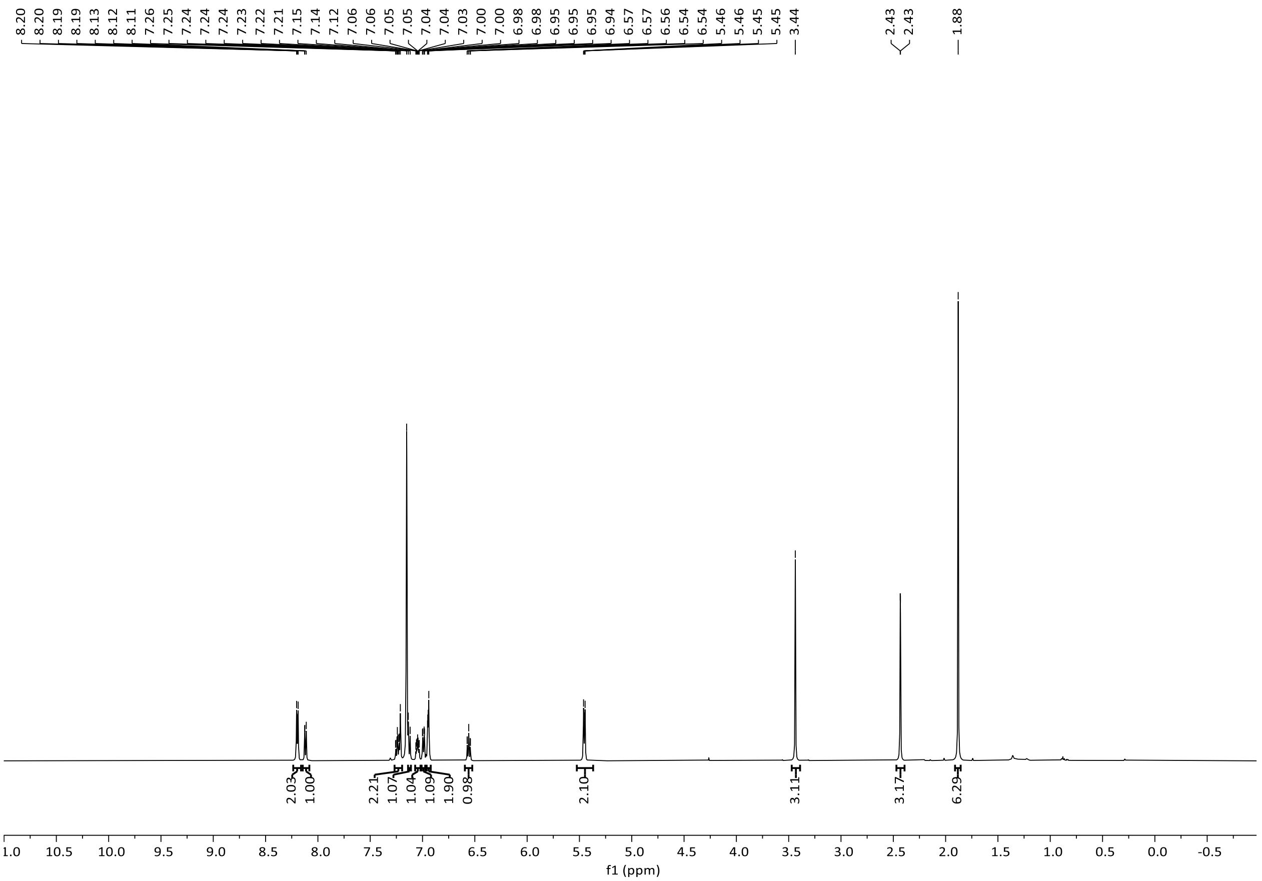

Figure S2. ^{1}H NMR spectrum (500 MHz, C_6D_6) of (MeFI)Ni(2-Me-C_6H_4)(DMAP).

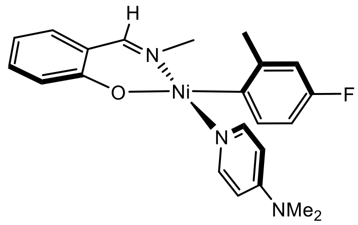

(MeFI)Ni(2-Me-4-F-C_6H_3)(DMAP): Prepared according to General Procedure A in 1.0 mmol scale. The title compound was isolated as a yellow solid (273 mg, 0.64 mmol, 64%). Purity of the title complex was determined to be 95% by ^{19}F NMR with C_6F_6 as internal standard. The analytical data were identical to literature.[2b] **^{1}H NMR** (500 MHz, C_6D_6, 25 ºC) δ_H 8.12 – 8.01 (m, 2H), 7.83 (t, *J* = 7.6 Hz, 1H), 7.23 (ddd, *J* = 8.6, 6.7, 1.9 Hz, 1H), 7.18 (s, 1H), 7.11 (d, *J* = 8.5 Hz, 1H), 6.99 (dd, *J* = 7.8, 1.9 Hz, 1H), 6.84 (td, *J* = 8.8, 2.8 Hz, 1H), 6.76 (dd, *J* = 11.0, 2.8 Hz, 1H), 6.56 (t, *J* = 7.3 Hz, 1H), 5.56 – 5.38 (m, 2H), 3.29 (s, 3H), 2.34 (s, 3H), 1.89 (s, 6H).

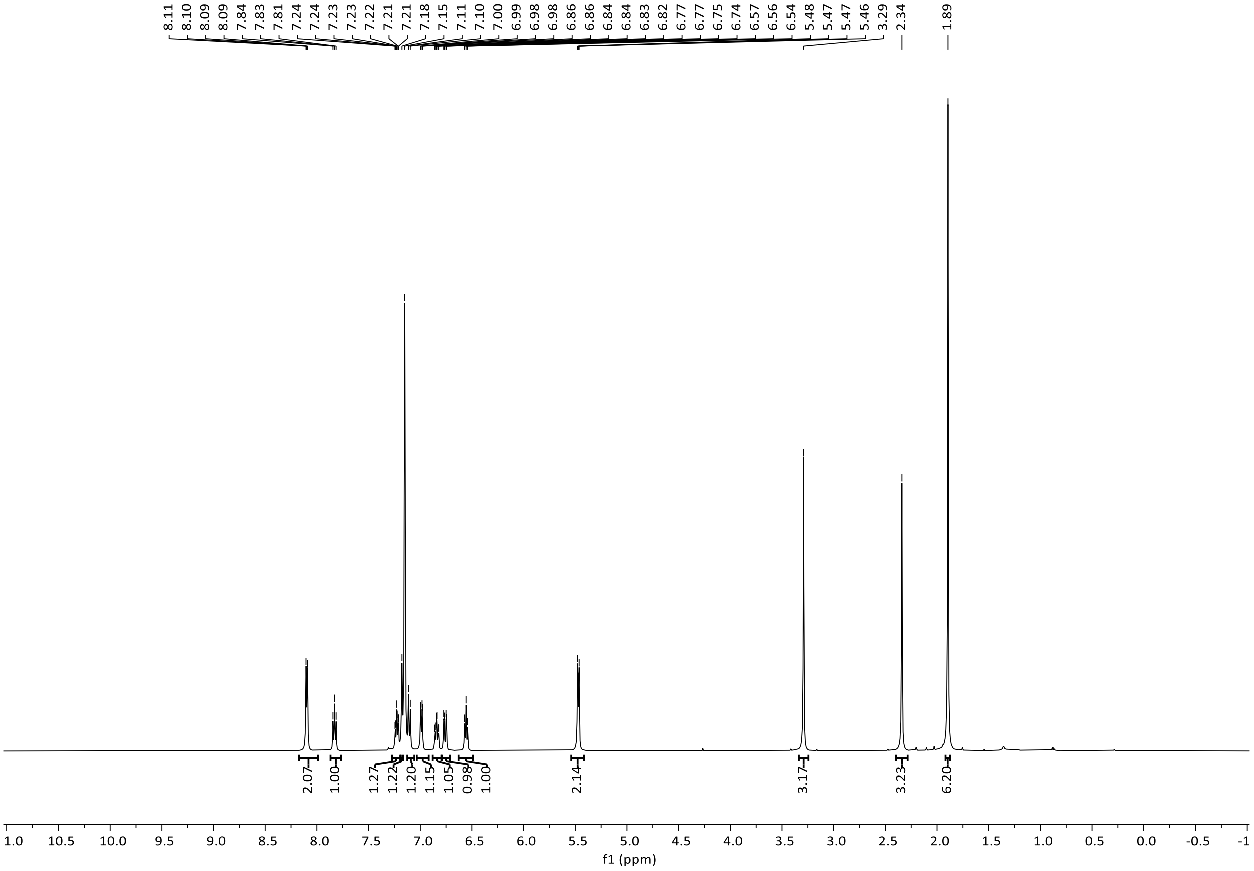

Figure S3. ^{1}H NMR spectrum (500 MHz, C_6D_6) of (MeFI)Ni(2-Me-4-F-C_6H_3)(DMAP).

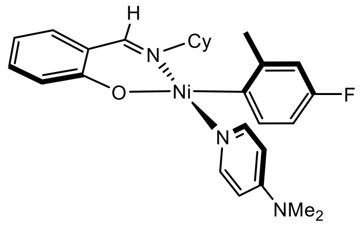

(CyFI)Ni(2-Me-4-F-C_6H_3)(DMAP): Prepared according to General Procedure A in 1.0 mmol scale. The title compound was isolated as a yellow solid (317 mg, 0.64 mmol, 64%). Purity of the title complex was determined to be 95% by ^{19}F NMR with C_6F_6 as internal standard. **^{1}H NMR** (500 MHz, C_6D_6, 25 ºC) δ_H 8.11 (d, *J* = 6.4 Hz, 2H), 7.89 (t, *J* = 7.5 Hz, 1H), 7.63 (s, 1H), 7.23 (dt, *J* = 6.9, 4.5 Hz, 1H), 7.14 – 7.08 (m, 2H), 6.90 – 6.71 (m, 2H), 6.59 (t, *J* = 7.3 Hz, 1H), 5.46 (d, *J* = 6.4 Hz, 2H), 3.37 (s, 3H), 2.57 – 2.48 (m, 1H), 1.99 (d, *J* = 12.2 Hz, 1H), δ 1.89 (s, 6H), 1.79 – 1.67 (m, 1H), 1.48 – 1.35 (m, 2H), 1.31 – 1.17 (m, 1H), 1.06 – 0.86 (m, 2H), 0.81 – 0.59 (m, 3H). **^{19}F NMR** (376 MHz, C_6D_6, 25 ºC): δ_F −124.6. **^{13}C NMR** (126 MHz, C_6D_6, 25 ºC) δ_C 166.7, 162.5, 162.0 (d, *J* = 260.8 Hz), 153.6, 151.0, 150.6, 143.9, 135.9 (d, *J* = 5.7 Hz), 133.8 (d, *J* = 3.1 Hz), 122.2, 121.9, 113.8 (d, *J* = 18.3 Hz), 113.5, 110.0 (d, *J* = 17.8 Hz), 106.1, 66.7, 38.1, 34.9, 34.3, 26.4, 26.2, 25.8, 25.6. **HRMS** *m/z* (ESI$^+$): calcd for $C_{27}H_{32}FN_3NiO$ (M^+): 491.1883; found: 491.1881.

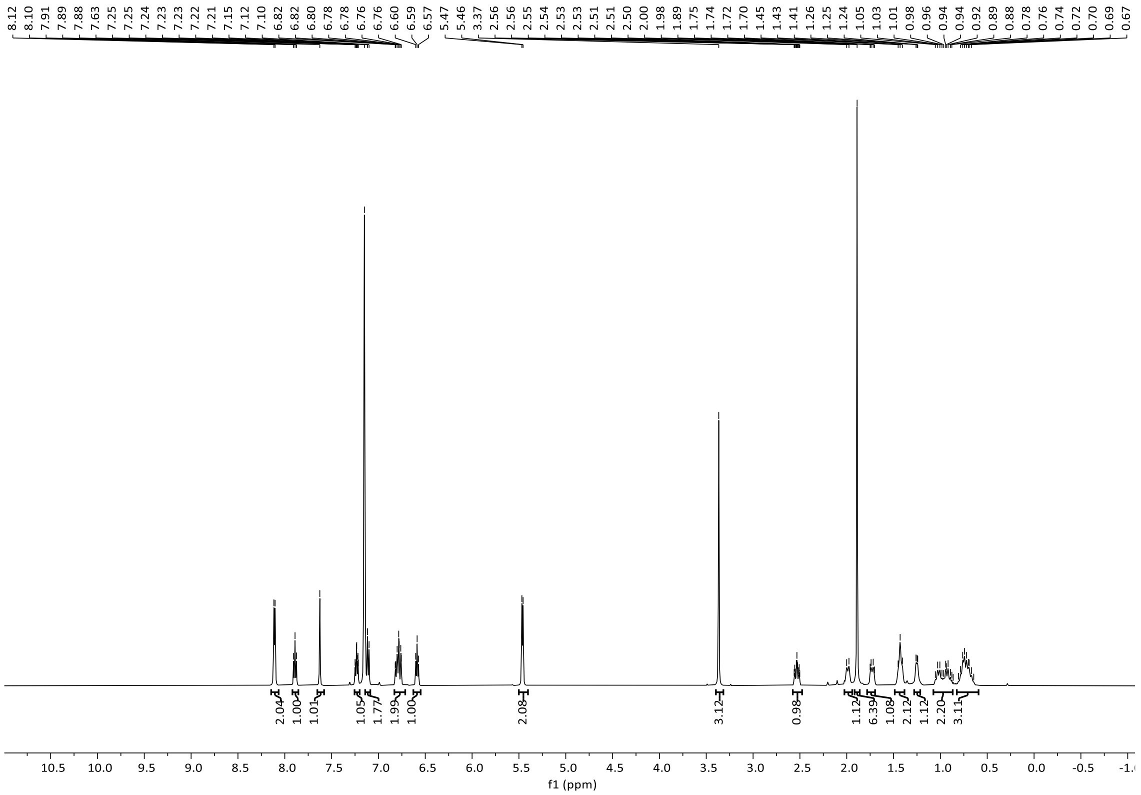

Figure S4. ^1H NMR spectrum (400 MHz, C_6D_6) of (CyFI)Ni(2-Me-4-F-C_6H_3)(DMAP).

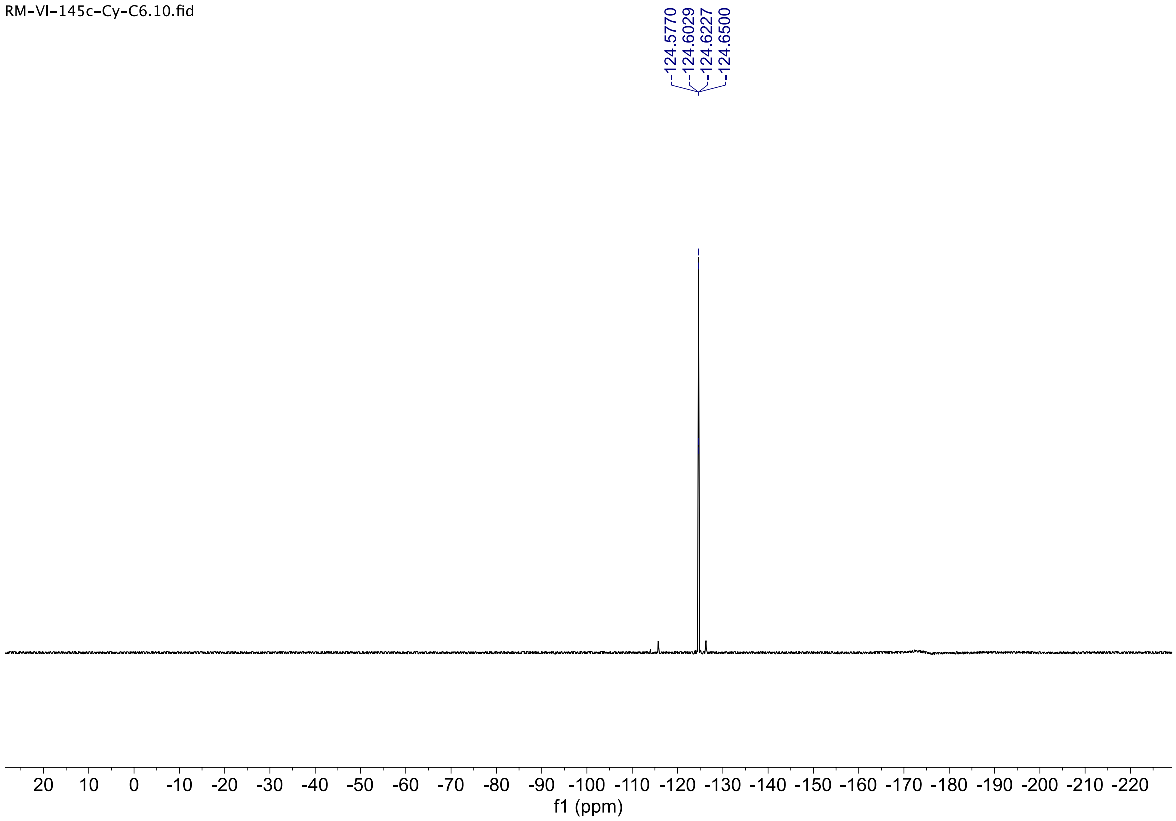

Figure S5. ^{19}F NMR spectrum of (376 MHz, C_6D_6) (CyFI)Ni(2-Me-4-F-C_6H_3)(DMAP).

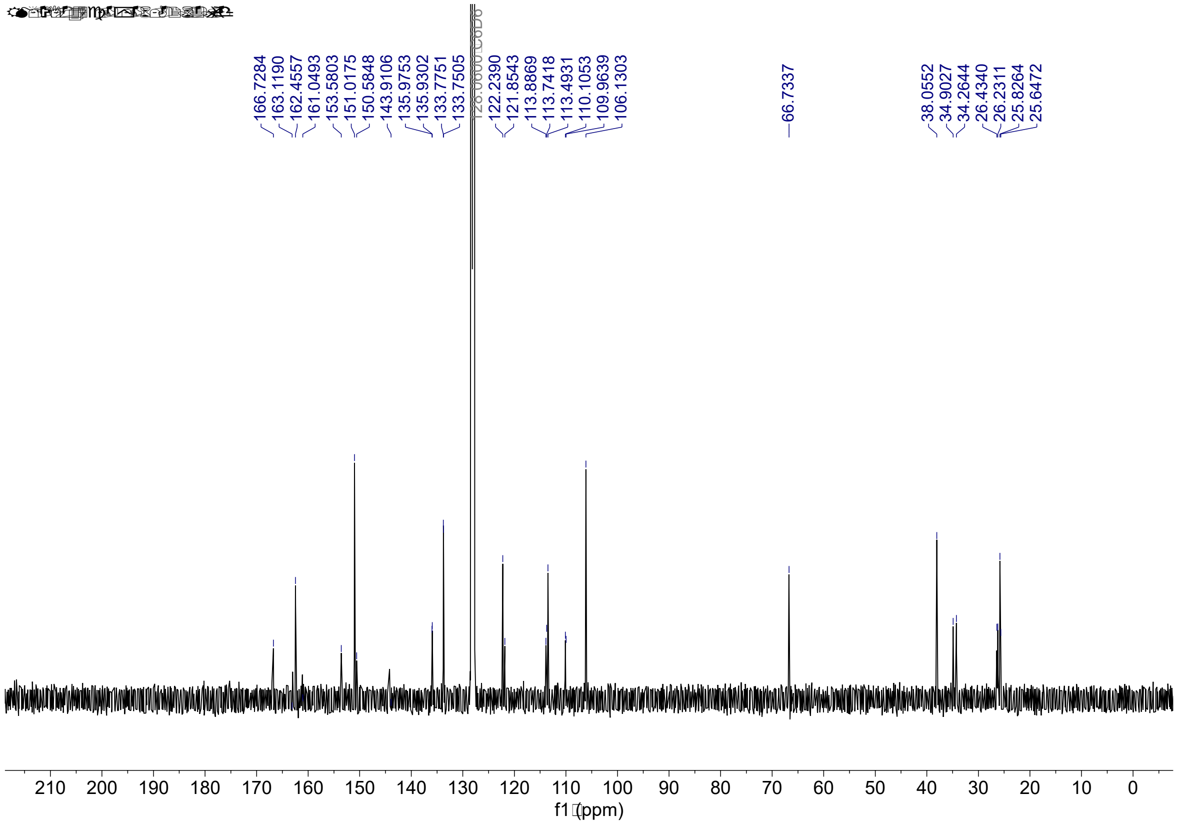

Figure S6. ^{13}C NMR spectrum (126 MHz, C_6D_6) of (CyFI)Ni(2-Me-4-F-C_6H_3)(DMAP).

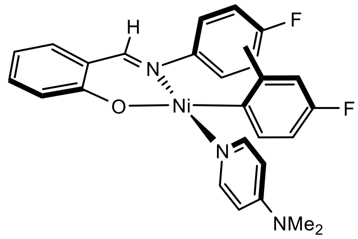

(4-F-C_6H_4-FI)Ni(2-Me-4-F-C_6H_3)(DMAP) Prepared according to General Procedure A in 1.0 mmol scale. The title compound was isolated as a yellow solid (307 mg, 0.61 mmol, 61%). Purity of the title complex was determined to be 94% by ^{19}F NMR with C_6F_6 as internal standard. The analytical data were identical to literature.[2b] **^{1}H NMR** (400 MHz, C_6D_6, 25 °C) δ_H 8.11 (d, *J* = 6.6 Hz, 2H), 7.50 (s, 1H), 7.40 (t, *J* = 7.6 Hz, 1H), 7.24 (ddd, *J* = 8.7, 6.7, 1.9 Hz, 1H), 7.12 (s, 1H), 6.97 (dd, *J* = 7.9, 1.9 Hz, 1H), 6.61 – 6.49 (m, 2H), 6.49 – 6.36 (m, 5H), 5.48 (d, *J* = 6.6 Hz, 2H), 3.15 (s, 3H), 1.89 (s, 6H).

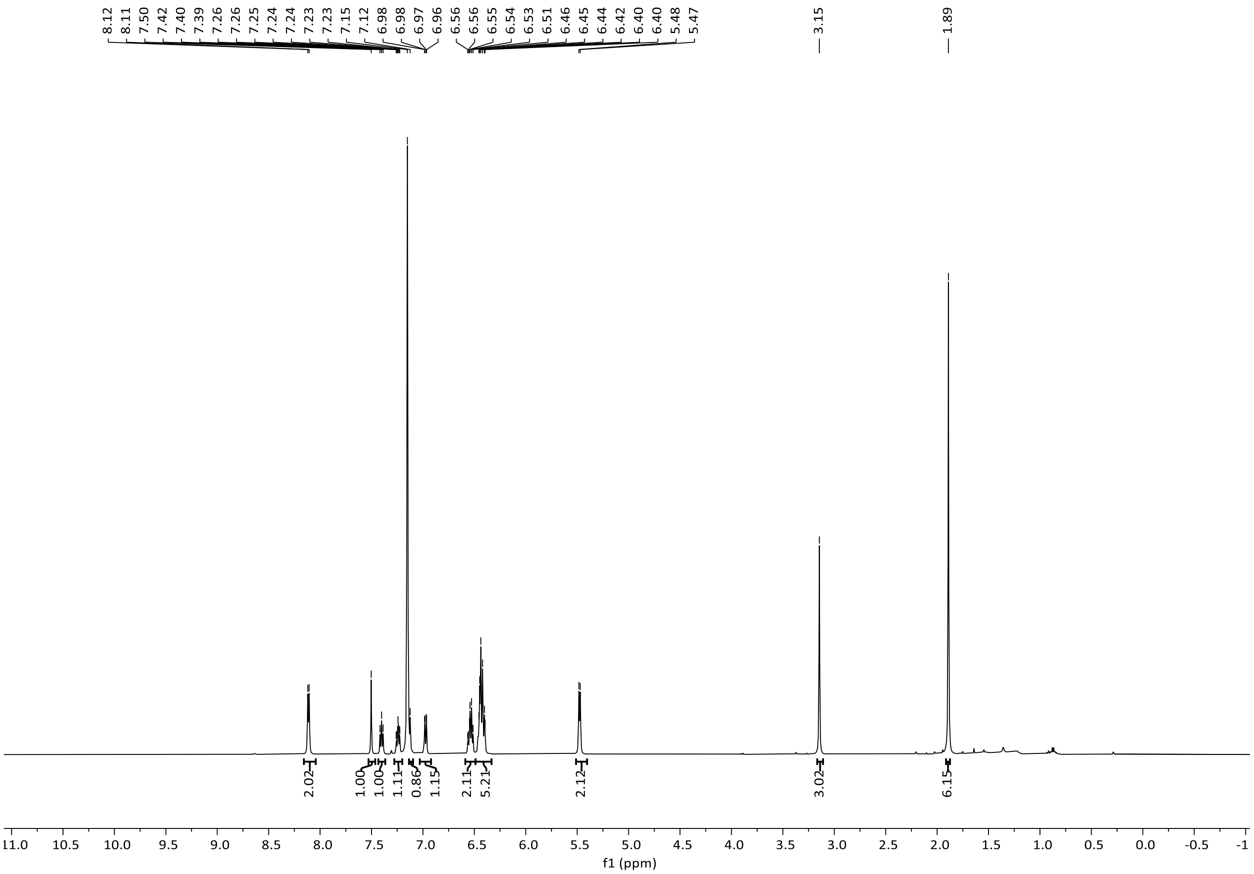

Figure S7. ^{1}H NMR spectrum (500 MHz, C_6D_6) of (4-F-Ph-FI)Ni(2-Me-4-F-C_6H_3)(DMAP).

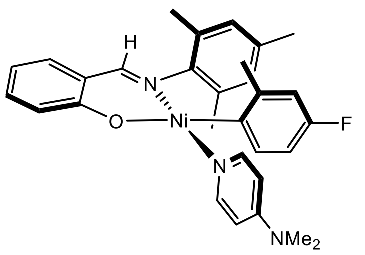

(4-F-C_6H_4-FI)Ni(2-Me-4-F-C_6H_3)(DMAP) Prepared according to General Procedure B in 0.5 mmol scale. The title compound was isolated as a orange-yellow solid (178 mg, 0.34 mmol, 67%). Purity of the title complex was determined to be >98% by ^{19}F NMR with C_6F_6 as internal standard. **^{1}H NMR** (500 MHz, C_6D_6, 25 ºC) δ_H 8.28 – 8.14 (m, 2H), 7.30 (s, 1H), 7.26 (ddd, *J* = 8.6, 6.7, 1.9 Hz, 1H), 7.21 – 7.18 (m, 1H), 7.06 (dd, *J* = 7.9, 7.0 Hz, 1H), 6.95 (dd, *J* = 7.9, 1.8 Hz, 1H), 6.70 (d, *J* = 2.2 Hz, 1H), 6.55 – 6.50 (m, 1H), 6.50 – 6.43 (m, 2H), 6.41 – 6.38 (m, 1H), 5.54 – 5.46 (m, 2H), 3.41 (s, 3H), 2.85 (s, 3H), 2.04 (s, 3H), 2.01 (s, 3H), 1.90 (s, 6H). **^{19}F NMR** (471 MHz, C_6D_6, 25 ºC) δ_F -125.50 (td, *J* = 10.2, 6.9 Hz). **^{13}C{^{1}H} NMR** (126 MHz, C_6D_6) δ_C 167.9, 166.9, 161.9 (d, *J* = 237.1 Hz), 153.9, 151.6, 151.3, 144.6 (d, *J* = 5.1 Hz), 144.1 (d, *J* = 2.6 Hz), 136.8 (d, *J* = 5.9 Hz), 134.8, 134.6, 134.4, 131.1, 129.9, 129.0, 128.9, 123.1, 121.8, 114.2, 112.6 (d, *J* = 18.2 Hz), 109.8 (d, *J* = 18.0 Hz), 106.3, 38.3, 26.24, 26.22, 21.1, 20.2, 18.6. **HRMS** *m/z* (ESI$^+$): calcd for $C_{23}H_{22}FNNiO^+$ (M-DMAP$^+$): 405.1033; found: 405.1967. calcd for $C_7H_{11}N_2^+$:123.0917 (DMAP+H$^+$); found 123.0922; calc for $C_{16}H_{17}NO$ (FI+H$^+$): 204.1383; found 240.1389.

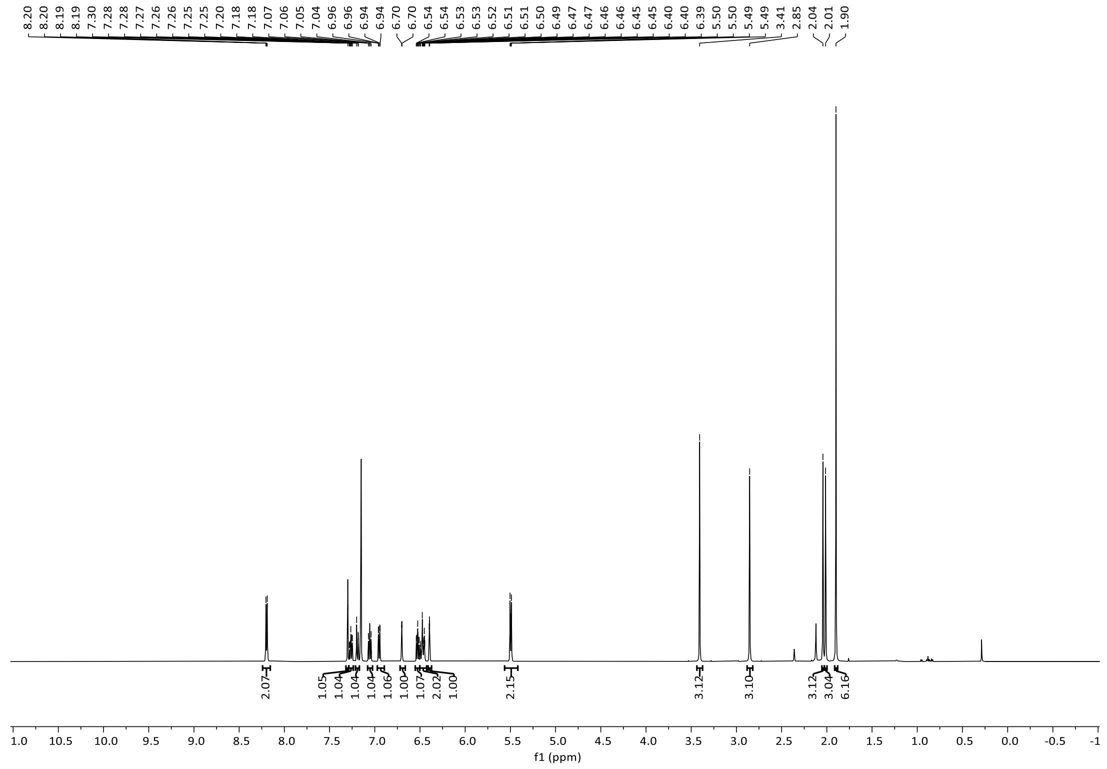

Figure S8. ^{1}H NMR spectrum (500 MHz, C_6D_6) of (Mes-FI)Ni(2-Me-4-F-C_6H_3)(DMAP).

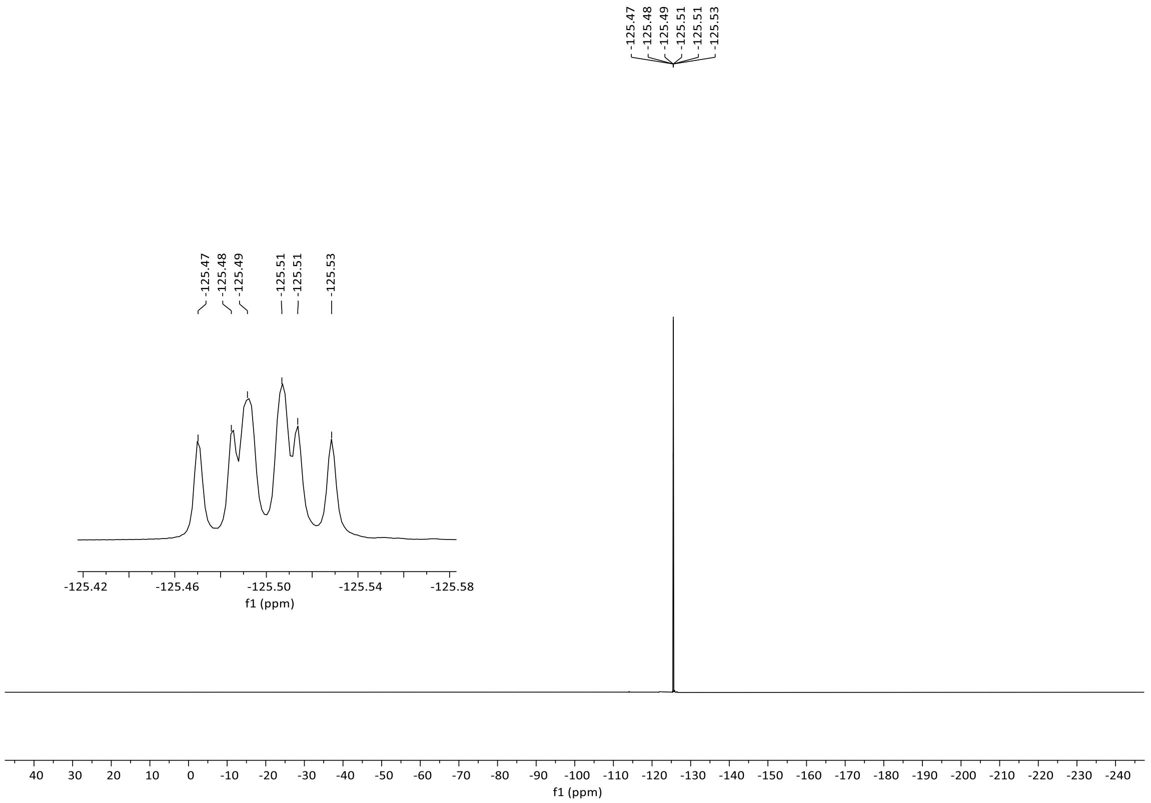

Figure S9. ^{19}F NMR spectrum (471 MHz, C_6D_6) of (Mes-FI)Ni(2-Me-4-F-C_6H_3)(DMAP).

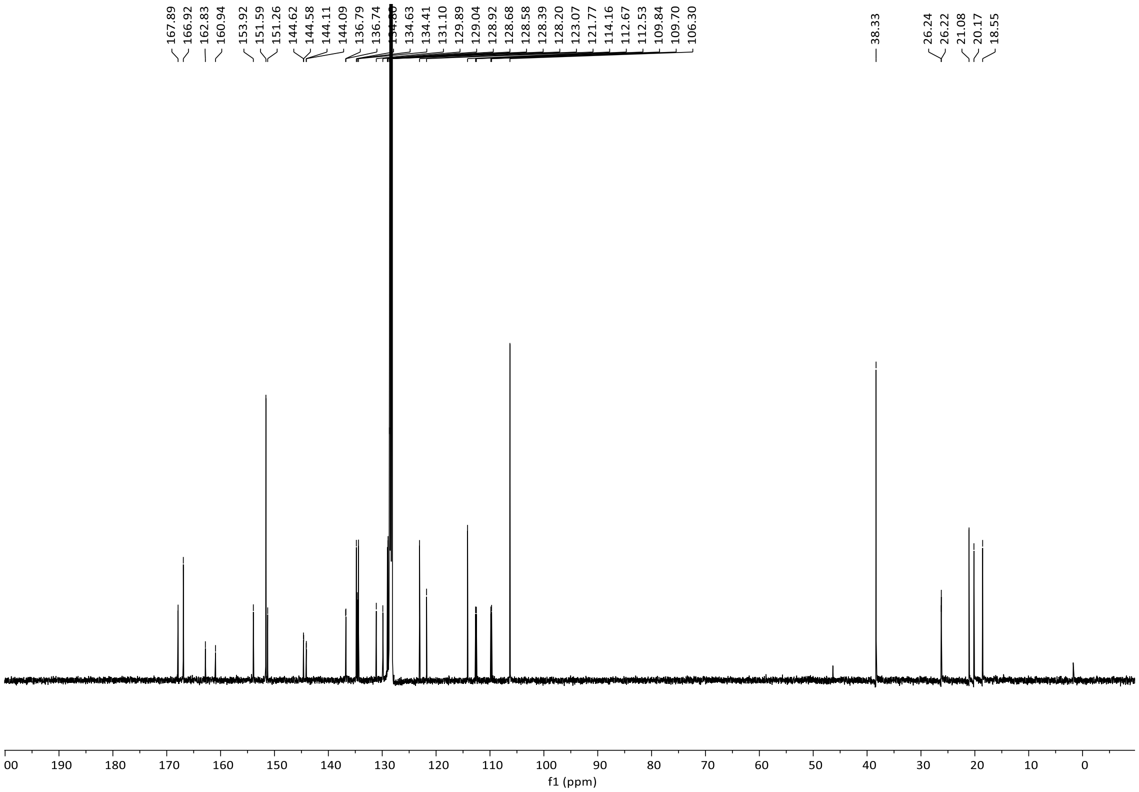

Figure S10. ^{13}C NMR spectrum (126 MHz, C_6D_6) of (Mes-FI)Ni(2-Me-4-F-C_6H_3)(DMAP).

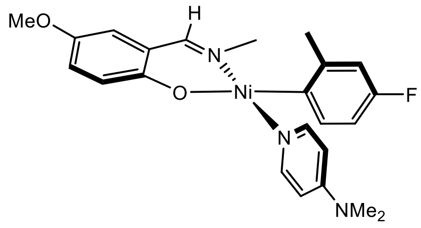

(Me^{OMe}FI)Ni(2-Me-4-F-C_6H_3)(DMAP): Prepared according to General Procedure A in 1.0 mmol scale. The title compound was isolated as a yellow solid (353 mg, 0.78 mmol, 78%). Purity of the title complex was determined to be 98% by ^{19}F NMR with C_6F_6 as internal standard. The analytical data were identical to literature.[2b] **^{1}H NMR** (500 MHz, C_6D_6, 25 °C) δ_H 8.12 (d, *J* = 6.7 Hz, 2H), 7.86 (t, *J* = 7.6 Hz, 1H), 7.14 (s, 1H), 7.08 – 6.99 (m, 2H), 6.86 (td, *J* = 8.8, 2.8 Hz, 1H), 6.78 (dd, *J* = 11.0, 2.8 Hz, 1H), 6.47 (d, *J* = 3.0 Hz, 1H), 5.48 (d, *J* = 6.5 Hz, 2H), 3.45 (s, 3H), 3.32 (s, 3H), 2.40 (s, 3H), 1.89 (s, 6H).

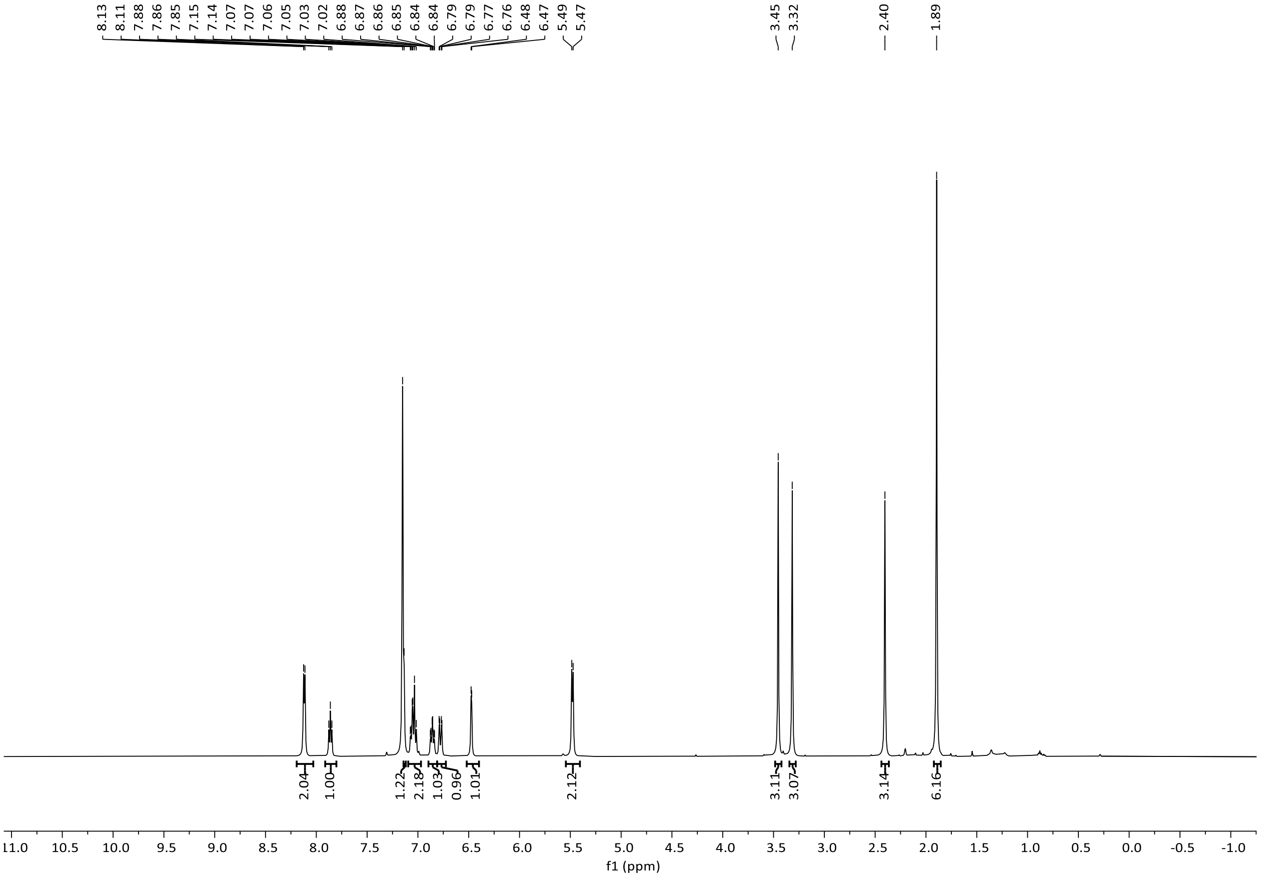

Figure S11. ^{1}H NMR spectrum (500 MHz, C_6D_6) of (Me^{OMe}FI)Ni(2-Me-4-F-C_6H_3)(DMAP).

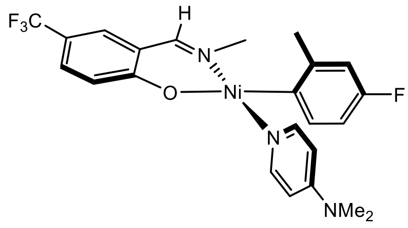

(Me^{CF3}FI)Ni(2-Me-4-F-C_6H_3)(DMAP): Prepared according to General Procedure A in 1.0 mmol scale. The title compound was isolated as a yellow solid (265 mg, 0.54 mmol, 54%). Purity of the title complex was determined to be 92% by ^{19}F NMR with C_6F_6 as internal standard. The analytical data were identical to literature.[2b] **^{1}H NMR** (500 MHz, C_6D_6, 25 ºC) δ_H 8.06 – 7.94 (m, 2H), 7.74 (dd, *J* = 8.1, 6.9 Hz, 1H), 7.33 (dd, *J* = 8.9, 2.5 Hz, 1H), 7.20 (d, *J* = 2.4 Hz, 1H), 6.90 – 6.78 (m, 3H), 6.74 (dd, *J* = 10.9, 2.7 Hz, 1H), 5.58 – 5.43 (m, 2H), 3.25 (s, 3H), 2.26 (s, 3H), 1.89 (s, 6H).

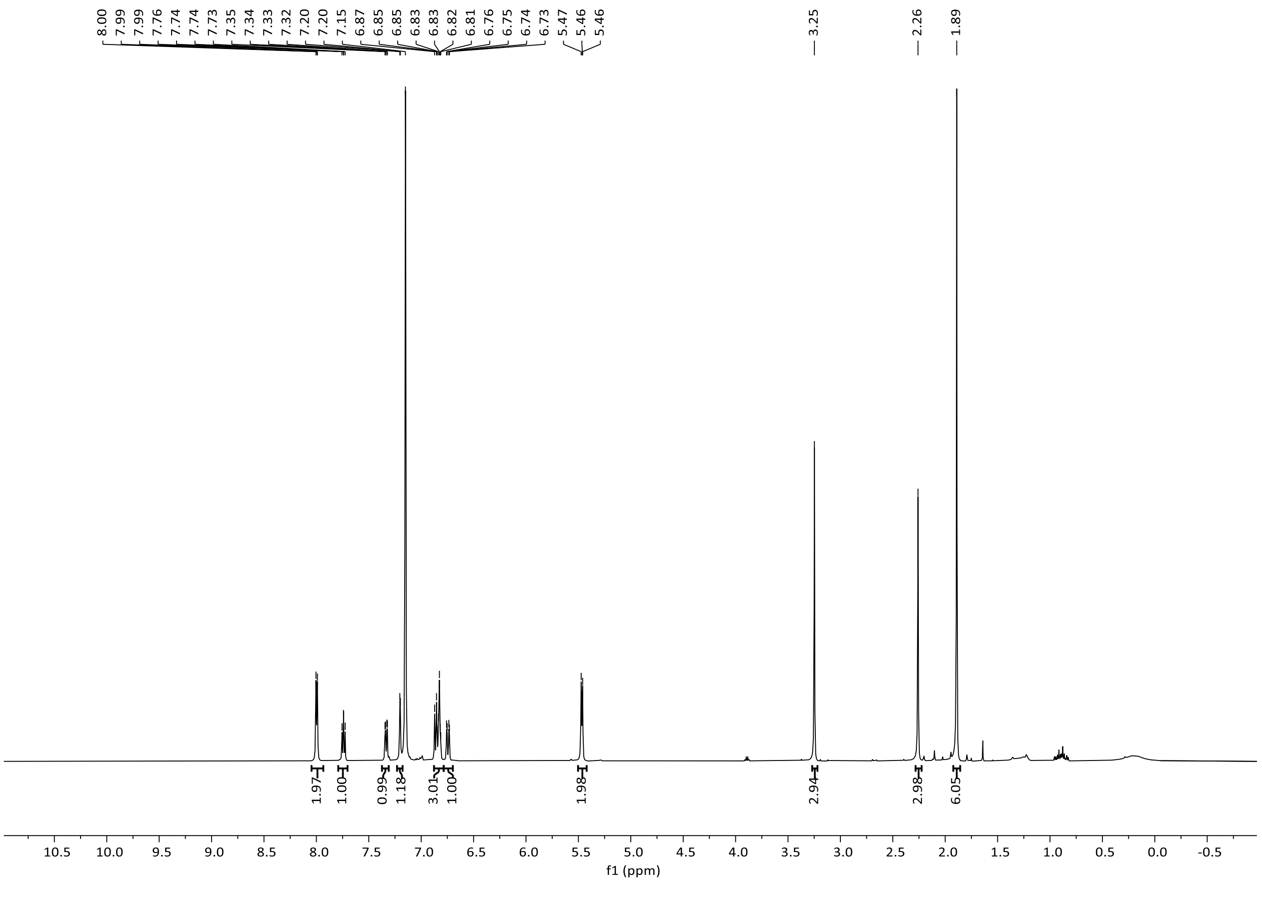

Figure S12. ^{1}H NMR spectrum (500 MHz, C_6D_6) of (Me^{CF3}FI)Ni(2-Me-4-F-C_6H_3)(DMAP).

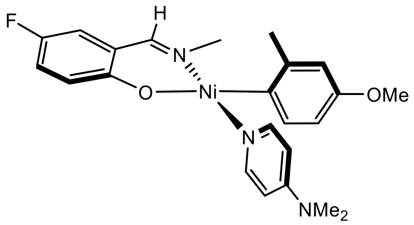

(Me^{F}FI)Ni(2-Me-4-OMe-C_6H_3)(DMAP): Prepared according to General Procedure A in 1 mmol scale. The title compound was isolated as a orange-yellow solid (228 mg, 0.50 mmol, 50%). Purity of the title complex was determined to be 95% by ^{19}F NMR with C_6F_6 as internal standard. **^{1}H NMR** (500 MHz, C_6D_6, 25 ºC): δ_H 8.25 – 8.07 (m, 2H), 7.87 (d, *J* = 8.0 Hz, 1H), 6.98 (s, 1H), 6.95 (td, *J* = 8.7, 3.3 Hz, 1H), 6.89 (dd, *J* = 9.2, 4.8 Hz, 1H), 6.79 (dd, *J* = 8.0, 2.8 Hz, 1H), 6.76 (d, *J* = 2.8 Hz, 1H), 6.66 (dd, *J* = 9.2, 3.3 Hz, 1H), 5.54 – 5.45 (m, 2H), 3.45 (s, 3H), 3.36 (s, 3H), 2.44 – 2.37 (m, 3H), 1.89 (s, 6H). **^{19}F NMR** (376 MHz, C_6D_6, 25 ºC): δ_F −133.4. **^{13}C NMR** (126 MHz, C_6D_6, 25 ºC): δ_C 164.8 (d, *J* = 3.0 Hz), 163.7, 157.8, 153.6, 152.6 (d, *J* = 228.9 Hz), 151.1, 143.3 (d, *J* = 18.0 Hz), 136.1, 122.9 (d, *J* = 7.3 Hz), 121.7 (d, *J* = 23.8 Hz), 119.2 (d, *J* = 7.9 Hz), 116.2 (s, *J* = 21.5 Hz), 114.1, 110.3, 106.1, 55.4, 54.7, 38.1, 25.5. **HRMS** *m/z* (ESI$^+$): calcd for $C_{23}H_{26}FN_3NiO_2$ (M^+): 453.1363; found: 453.1308.

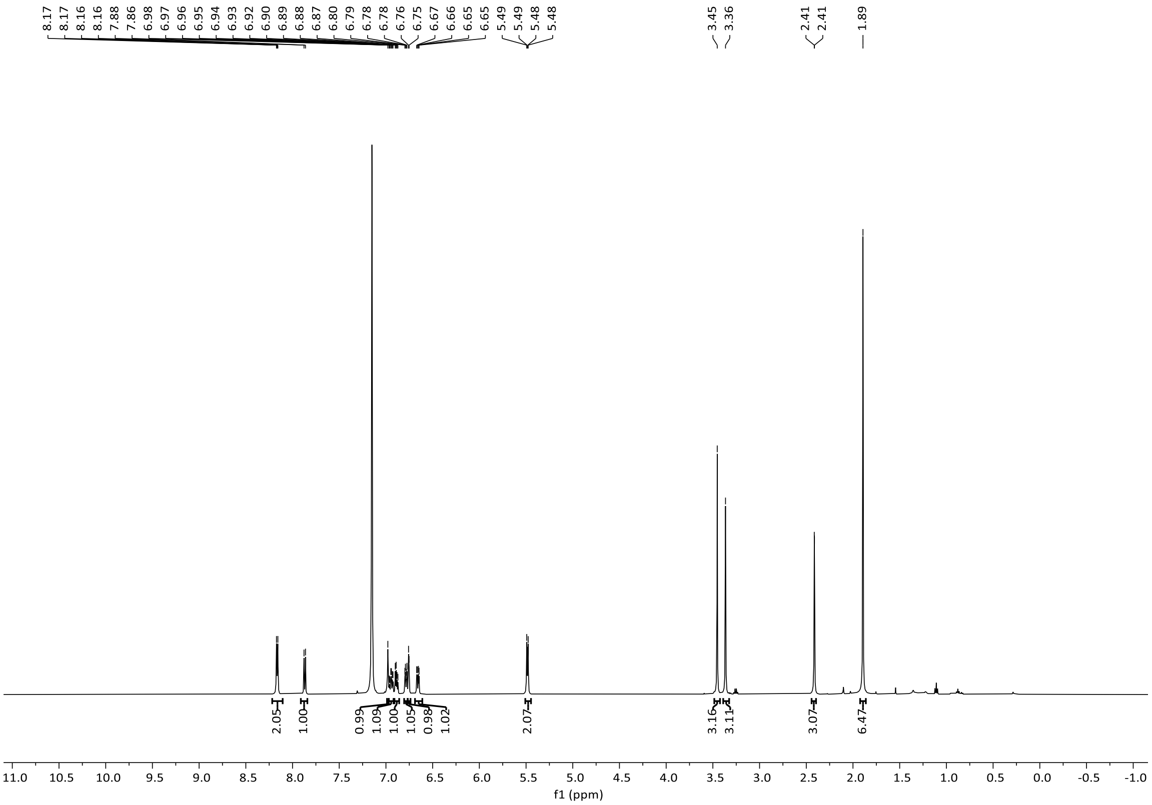

Figure S13. ^{1}H NMR spectrum (500 MHz, C_6D_6) of (Me^FFI)Ni(2-Me-4-OMe-C_6H_3)(DMAP).

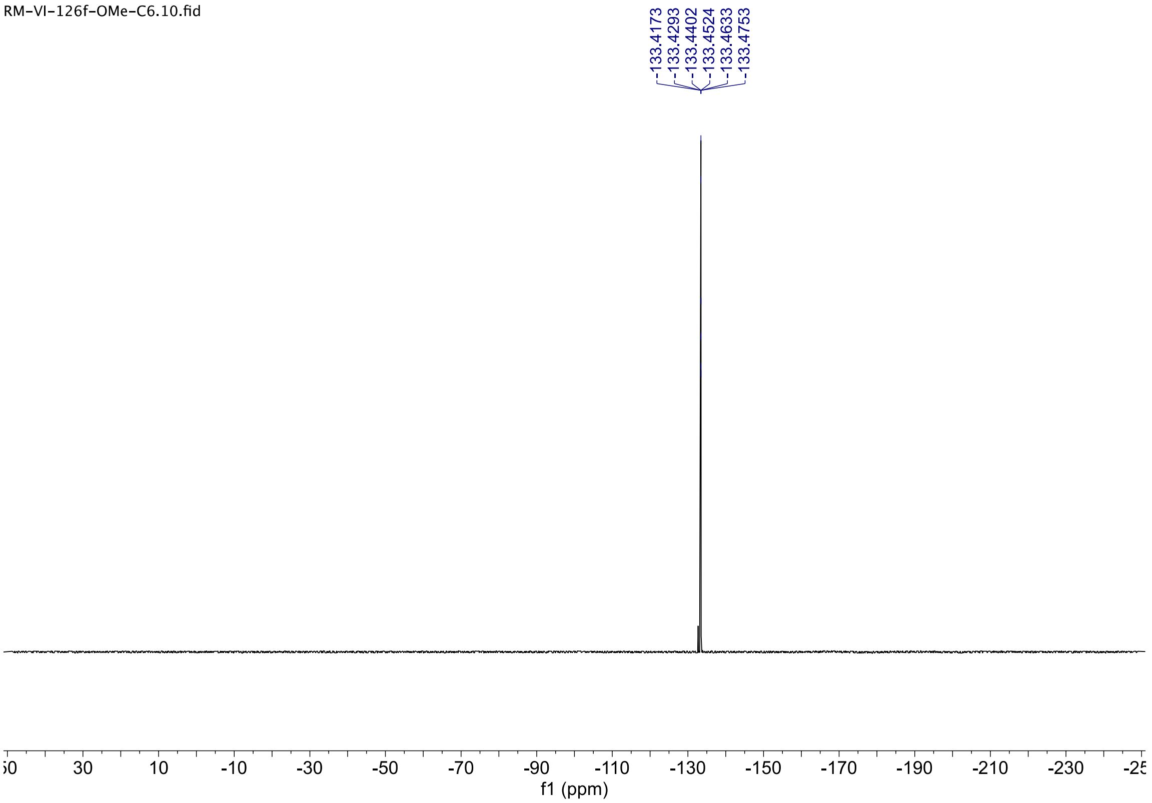

Figure S14. ^{19}F NMR spectrum (376 MHz, C_6D_6) of (Me^FFI)Ni(2-Me-4-OMe-C_6H_3)(DMAP).

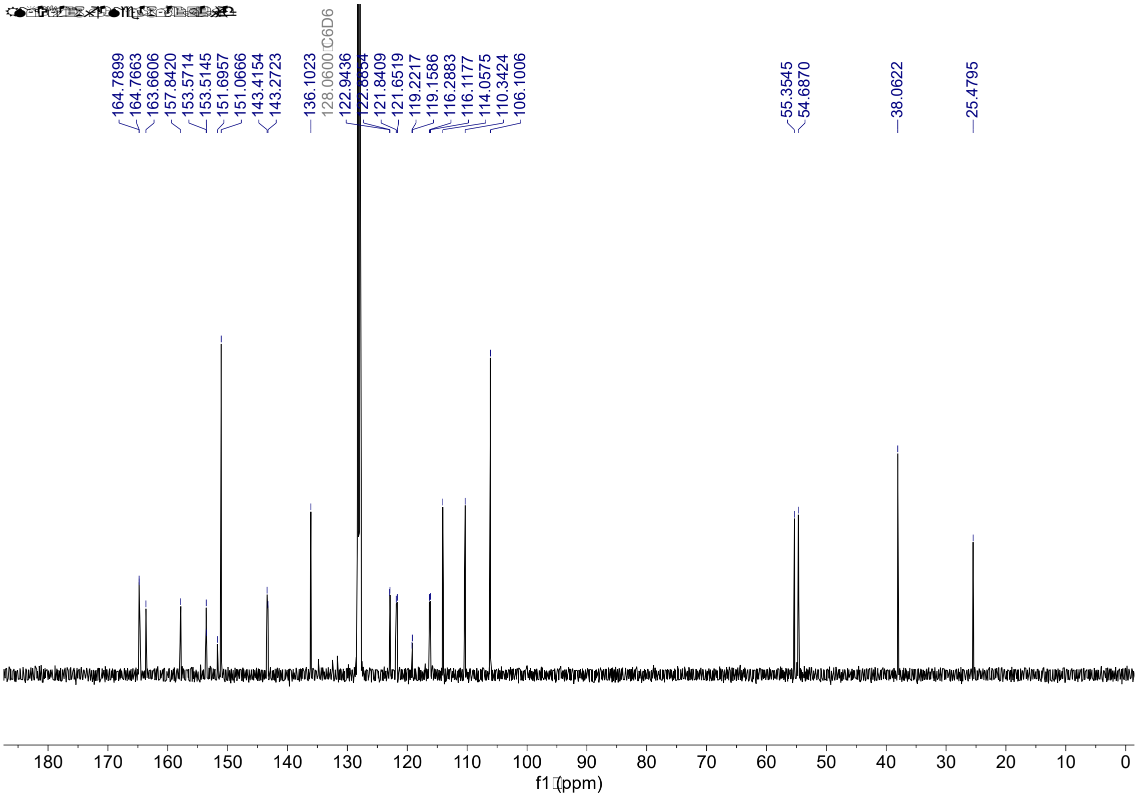

Figure S15. ^{13}C NMR spectrum (126 MHz, C_6D_6) of (Me^{F}FI)Ni(2-Me-4-OMe-C_6H_3)(DMAP).

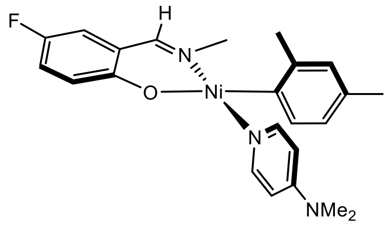

(Me^{F}FI)Ni(2,4-di-Me-C_6H_3)(DMAP): Prepared according to General Procedure A in 1 mmol scale. The title compound was isolated as an orange-yellow solid (240 mg, 0.55 mmol, 55%). Purity of the title complex was determined to be 96% by ^{19}F NMR with C_6F_6 as internal standard. **^{1}H NMR** (500 MHz, C_6D_6, 25 ºC): δ_H 8.24 – 8.13 (m, 2H), 7.96 (d, *J* = 7.5 Hz, 1H), 6.98 (s, 1H), 6.94 (td, *J* = 8.8, 3.2 Hz, 1H), 6.92 – 6.86 (m, 2H), 6.80 – 6.77 (m, 1H), 6.65 (dd, *J* = 9.2, 3.3 Hz, 1H), 5.53 – 5.39 (m, 2H), 3.40 (s, 3H), 2.47 – 2.36 (m, 3H), 2.27 (s, 3H), 1.88 (s, 6H). **^{19}F NMR** (376 MHz, C_6D_6, 25 ºC): δ_F −133.5. **^{13}C NMR** (126 MHz, C_6D_6): δ_C 164.8, 163.9, 153.5, 152.5 (d, *J* = 248.9 Hz), 152.1, 151.1, 143.0, 136.3, 131.5, 128.6, 125.0, 123.0 (d, *J* = 7.4 Hz), 121.7 (d, *J* = 23.8 Hz), 119.0, 116.2 (d, *J* = 22.3 Hz), 106.1, 55.4, 38.0, 25.4, 21.0. **HRMS** *m/z* (ESI$^+$): calcd for $C_{23}H_{26}FN_3NiO$ (M^+): 437.1413; found: 437.2311.

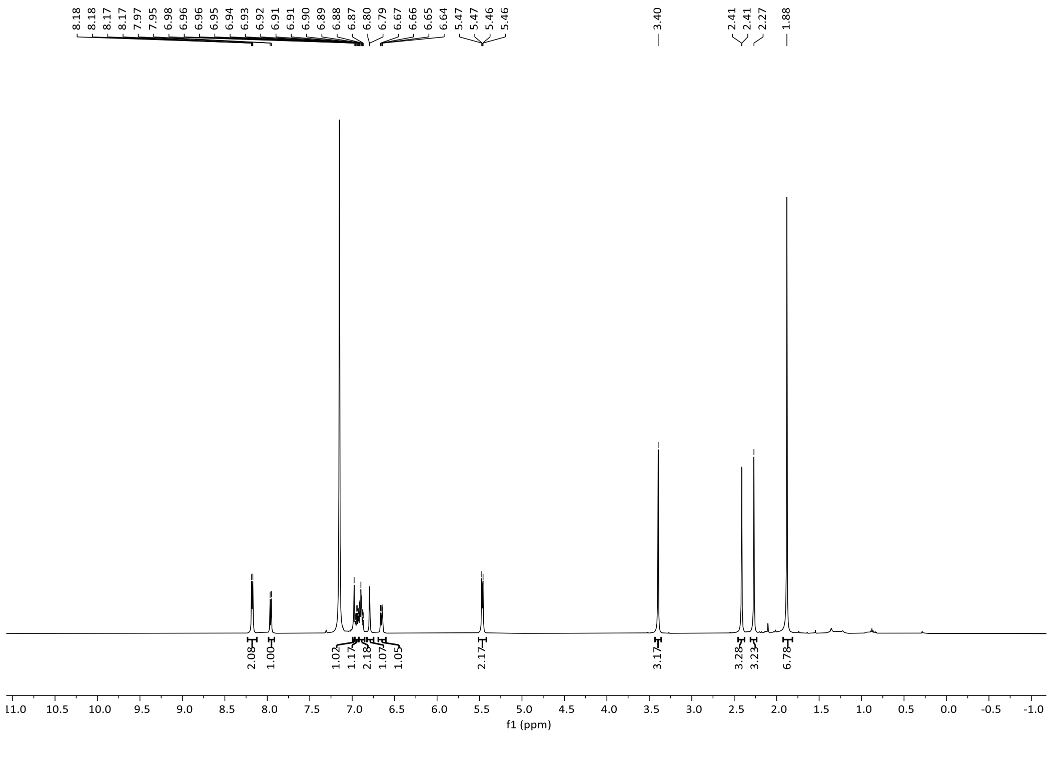

Figure S16. ^{1}H NMR spectrum (500 MHz, C_6D_6) of (Me^{F}FI)Ni(2,4-di-Me-C_6H_3)(DMAP).

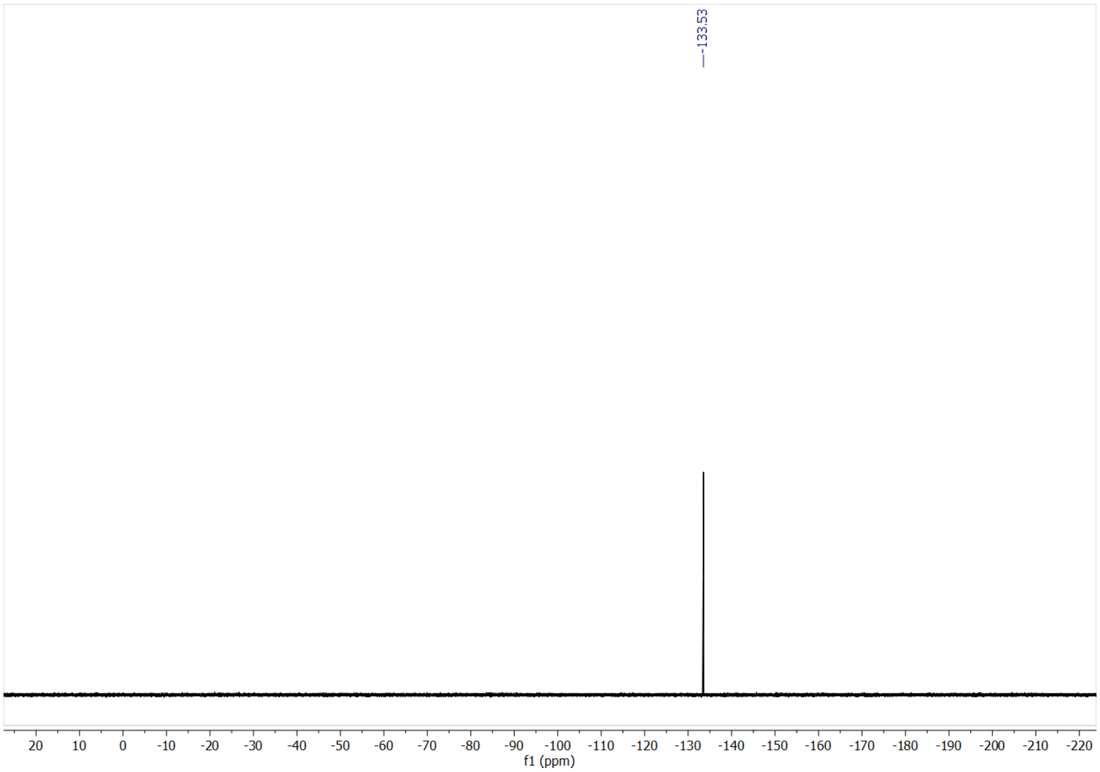

Figure S17. ^{19}F NMR spectrum (376 MHz, C_6D_6) of (Me^{F}FI)Ni(2,4-di-Me-C_6H_3)(DMAP).

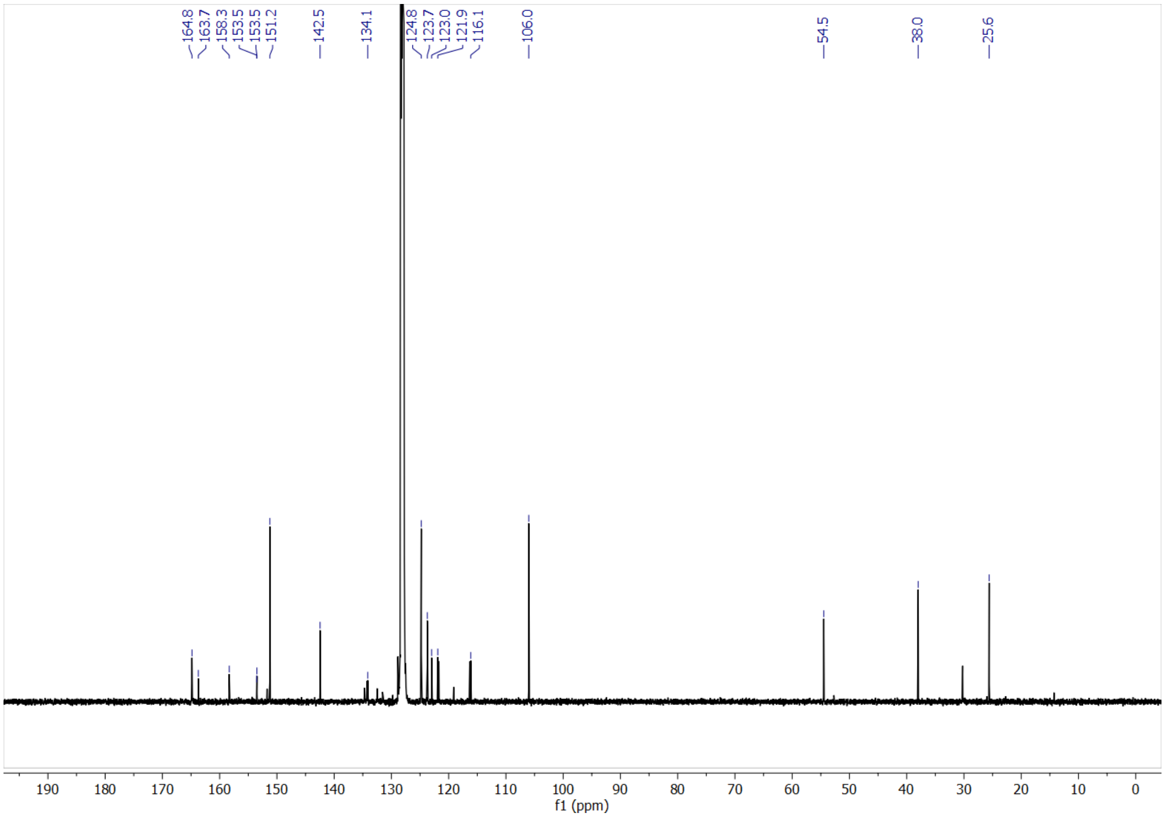

Figure S18. ^{13}C NMR spectrum (126 MHz, C_6D_6) of (Me^{F}FI)Ni(2,4-di-Me-C_6H_3)(DMAP).

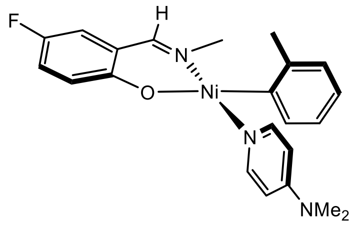

(Me^{F}FI)Ni(2-Tol)(DMAP): Prepared according to General Procedure A in 1 mmol scale. The title compound was isolated as a orange-yellow solid (286 mg, 0.67 mmol, 67%). Purity of the title complex was determined to be >98% by ^{19}F NMR with C_6F_6 as internal standard. **^{1}H NMR** (500 MHz, C_6D_6, 25 ºC): δ_H 8.17 – 8.11 (m, 2H), 8.08 (d, *J* = 7.3 Hz, 1H), 7.04 (td, *J* = 7.7, 7.1, 2.5 Hz, 1H), 6.99 – 6.91 (m, 4H), 6.88 (dd, *J* = 9.3, 4.8 Hz, 1H), 6.64 (dd, *J* = 9.2, 3.3 Hz, 1H), 5.51 – 5.39 (m, 2H), 3.41 (s, 3H), 2.38 (d, *J* = 1.1 Hz, 3H), 1.88 (s, 6H). **^{19}F NMR** (376 MHz, C_6D_6, 25 ºC): δ_F −133.3. **^{13}C NMR** (126 MHz, C_6D_6, 25 ºC): δ_C 164.9 (d, *J* = 2.9 Hz), 163.7, 157.8, 153.3, 151.7, 151.1, 143.3, 136.6, 127.0, 123.9, 123.0, 122.9, 121.8 (d, *J* = 23.8 Hz), 119.1 (d, *J* = 7.7 Hz), 116.2 (d, *J* = 21.5 Hz), 106.1, 55.3, 38.0, 25.5. **HRMS** *m/z* calcd for $C_{22}H_{24}FN_3NiO$ (M^+): 423.1257; found: 423.1263.

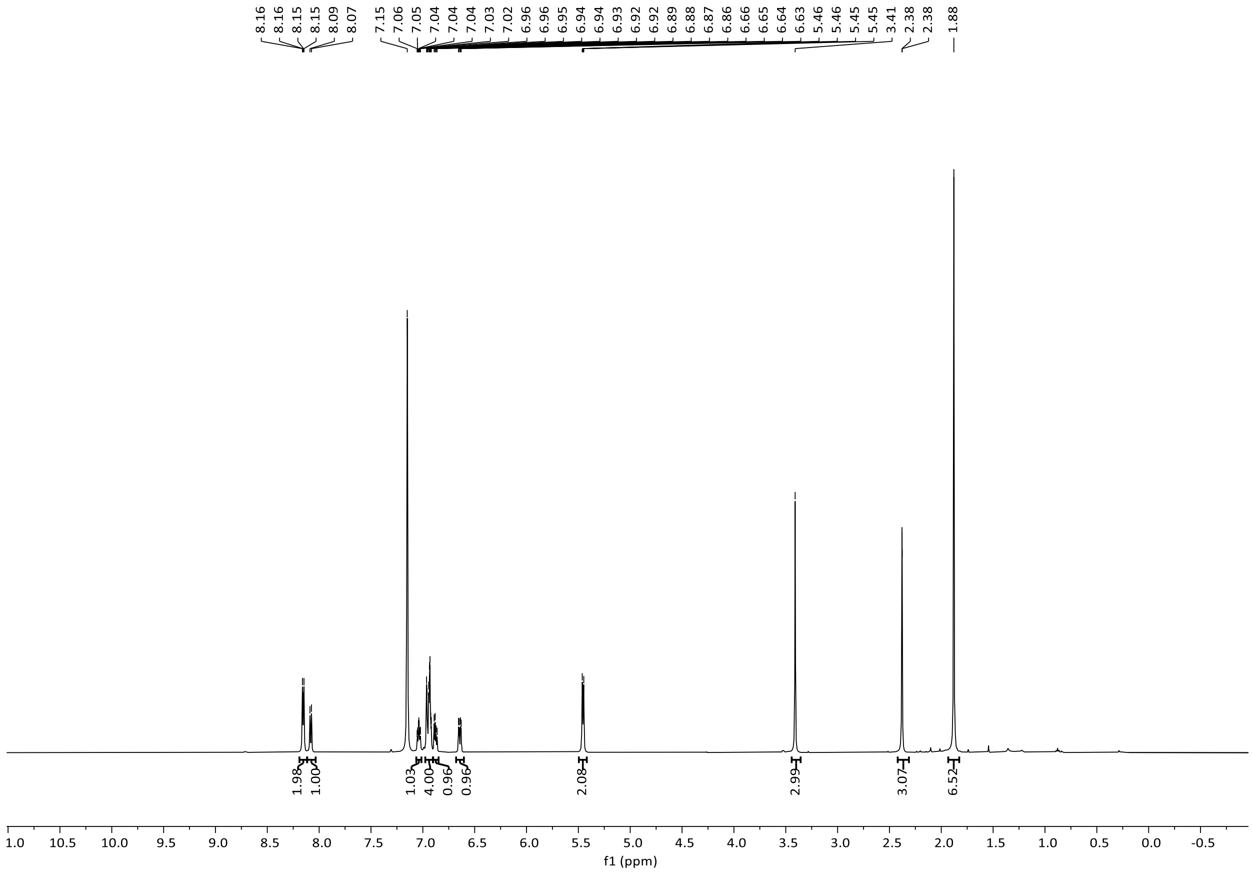

Figure S19. ^{1}H NMR spectrum (500 MHz, C_6D_6) of ($Me^{F}FI$)Ni(2-Tol)(DMAP).

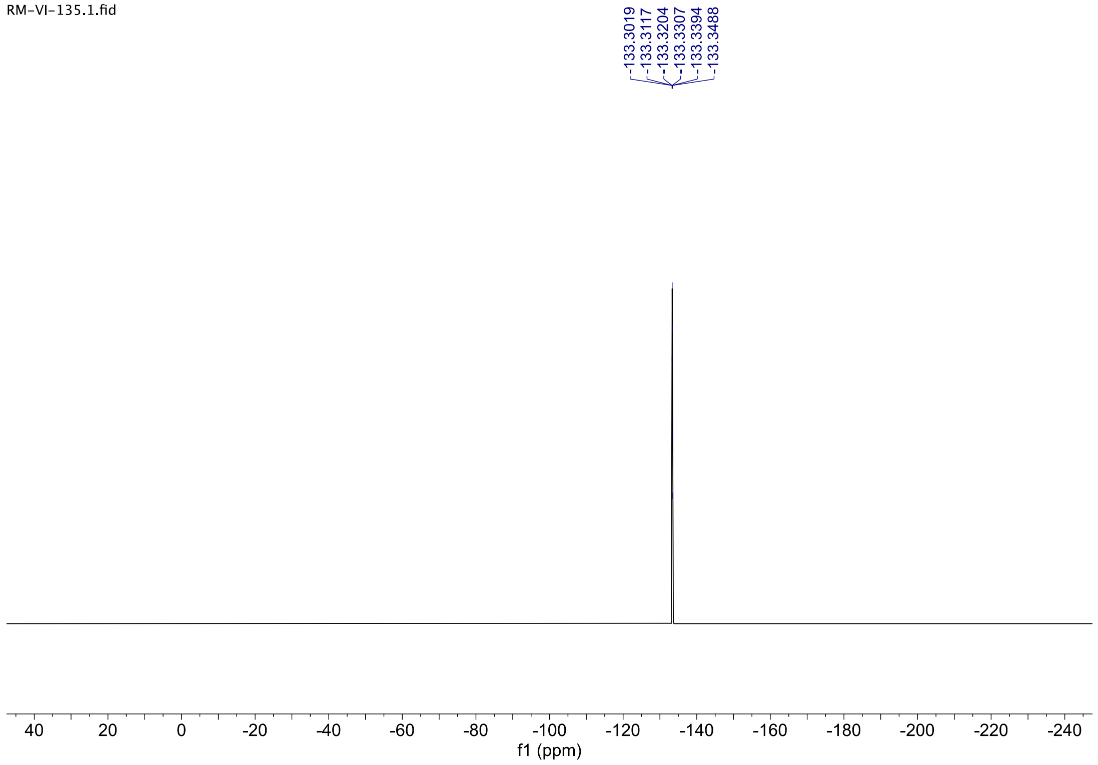

Figure S20. ^{19}F NMR spectrum (470 MHz, C_6D_6, 25 ºC) of ($Me^{F}FI$)Ni(2-Tol)(DMAP).

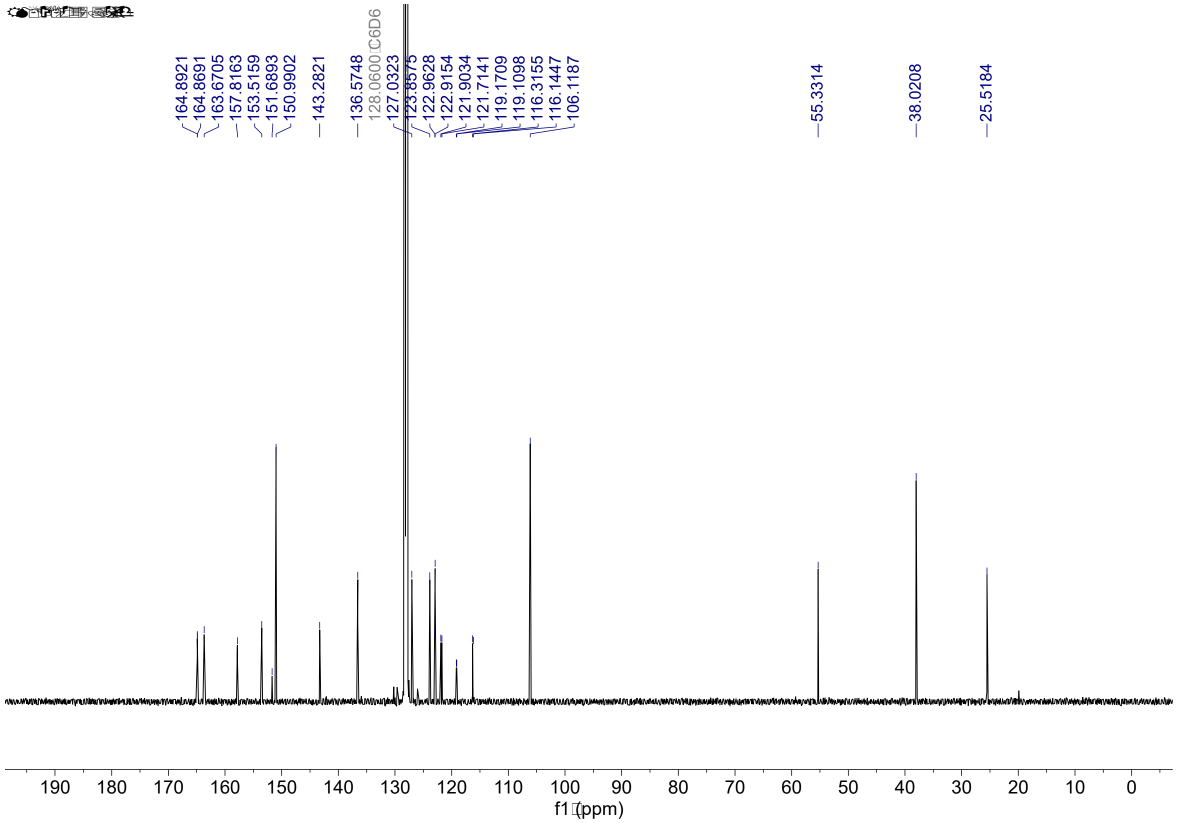

Figure S21. ^{13}C NMR spectrum (126 MHz, C_6D_6, 25 ºC) of (Me^{F}FI)Ni(2-Tol)(DMAP).

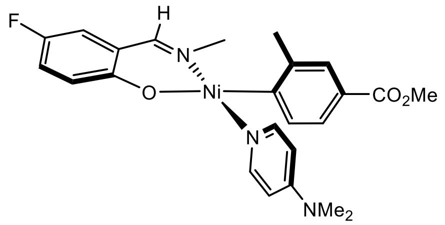

(Me^F FI)Ni(2-Me-4-CO$_2$Me-C$_6$H$_3$)(DMAP): Prepared according to General Procedure A in 1 mmol scale. The title compound was isolated as a orange-yellow solid (404 mg, 0.84 mmol, 84%). Purity of the title complex was determined to be 95% by ^{19}F NMR with C_6F_6 as internal standard. **^{1}H NMR** (400 MHz, C_6D_6, 25 ºC): δ_H 8.14 (d, *J* = 7.7 Hz, 1H), 8.08 – 7.99 (m, 2H), 7.95 – 7.91 (m, 1H), 7.85 (d, *J* = 2.0 Hz, 1H), 6.96 – 6.89 (m, 2H), 6.86 (dd, *J* = 9.2, 4.7 Hz, 1H), 6.64 (dd, *J* = 9.1, 3.2 Hz, 1H), 5.62 – 4.89 (m, 2H), 3.56 (s, 3H), 3.35 (s, 3H), 2.25 (s, 3H), 1.88 (s, 6H). **^{19}F NMR** (376 MHz, C_6D_6, 25 ºC): δ_F −133.0. **^{13}C NMR** (126 MHz, C_6D_6, 25 ºC): δ_C 171.5, 168.4, 165.1, 163.4, 153.6, 151.8, 150.7, 143.7, 136.6, 127.0, 125.8, 124.1, 122.8 (d, *J* = 7.3 Hz), 122.1 (d, *J* = 23.9 Hz), 119.0 (d, *J* = 7.9 Hz), 116.2 (d, *J* = 21.5 Hz), 106.2, 55.2, 51.1, 38.0, 25.3. **HRMS** *m/z* (ESI$^+$): calcd for $C_{24}H_{26}FN_3NiO_3$ (M$^+$): 481.1306; found: 481.1453.

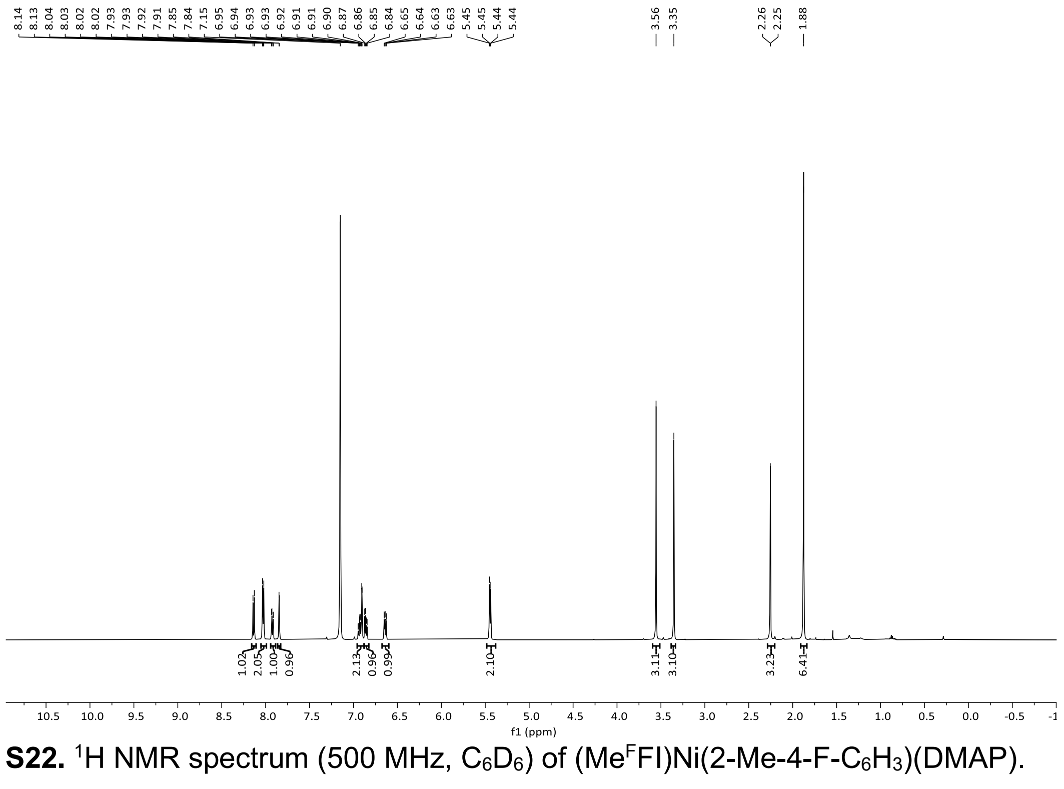

Figure S22. ^{1}H NMR spectrum (500 MHz, C_6D_6) of (Me^F FI)Ni(2-Me-4-F-C$_6$H$_3$)(DMAP).

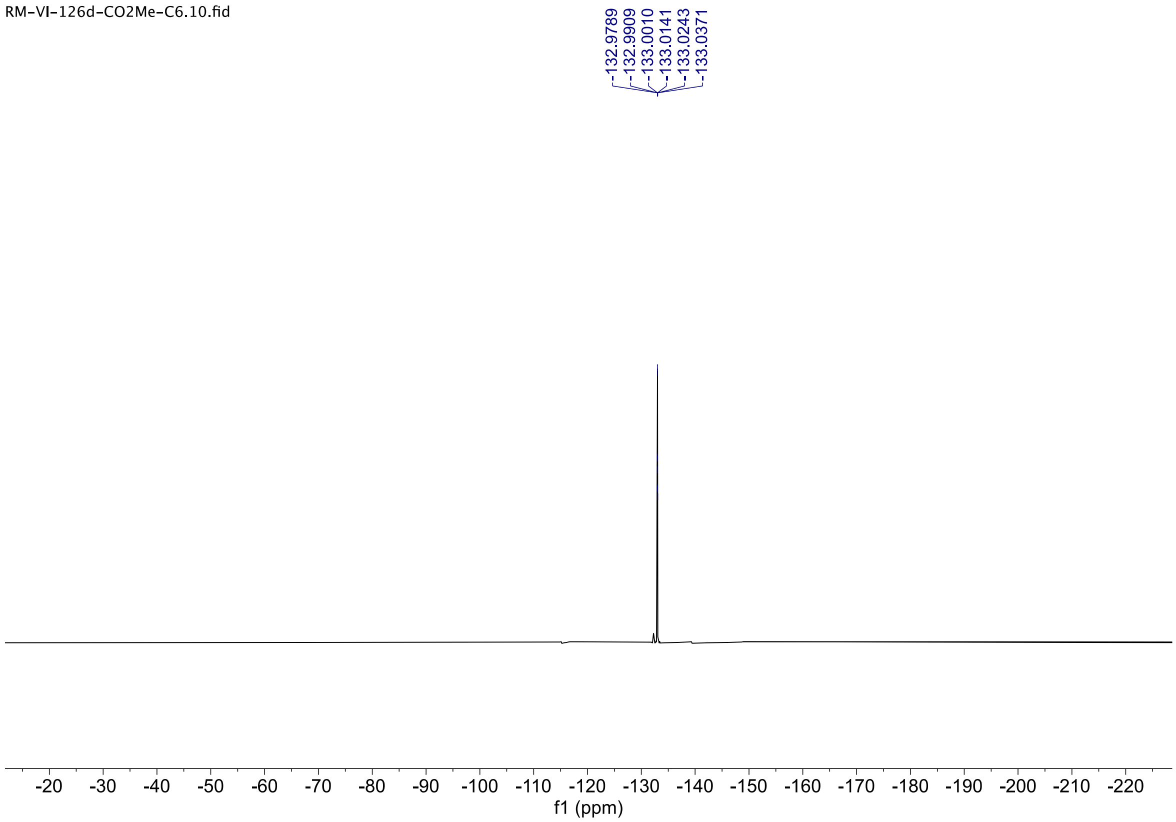

Figure S23. ^{19}F NMR spectrum (376 MHz, C_6D_6) of (Me^{F}FI)Ni(2-Me-4-F-C_6H_3)(DMAP).

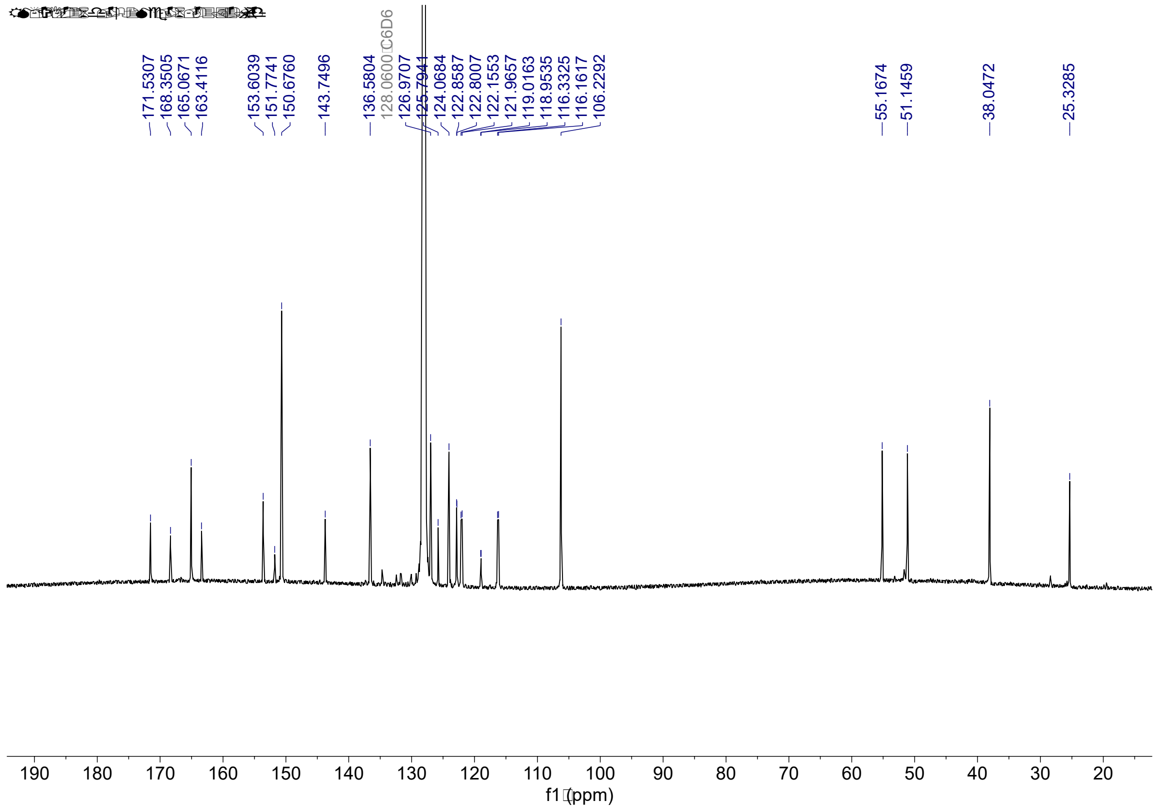

Figure S24. ^{13}C NMR spectrum (126 MHz, C_6D_6) of (Me^{F}FI)Ni(2-Me-4-F-C_6H_3)(DMAP).

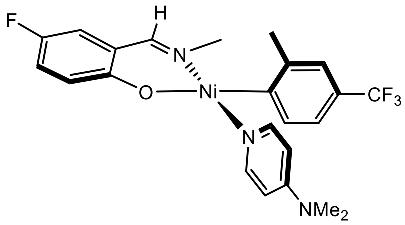

(Me^{F}FI)Ni(2-Me-4-CF$_3$-C$_6$H$_3$)(DMAP): Prepared according to General Procedure A employing K(Me^{F}FI) (0.19 g, 1.0 mmol, 1.0 equiv), Ni(COD)$_2$ (0.28 g, 1.0 mmol, 1.0 equiv), DMAP (0.12 g, 1.0 mmol, 1.0 equiv), catalytic PPh$_3$ (13 mg, 0.050 mmol, 0.050 equiv), PhH (10 mL, 0.10 M), and 1-bromo-2-methyl-4-(trifluoromethyl)benzene (0.29 g, 1.2 mmol, 1.2 equiv), yielding the desired compound as a yellow solid (0.45 g, 91%). Purity of the title complex was determined to be 94% by ^{19}F NMR with C$_6$F$_6$ as internal standard. **^{1}H NMR** (400 MHz, C$_6$D$_6$, 25 ºC): δ_H 8.01 (d, *J* = 7.7 Hz, 1H), 7.98 (d, *J* = 7.4 Hz, 2H), 7.23 (dd, *J* = 7.8, 2.0 Hz, 1H), 6.92 (td, *J* = 8.7, 8.1, 3.2 Hz, 1H), 6.88 (s, 1H), 6.85 (dd, *J* = 9.2, 4.7 Hz, 1H), 6.63 (dd, *J* = 9.1, 3.2 Hz, 1H), 5.43 (d, *J* = 7.4 Hz, 2H), 3.27 (s, 3H), 2.18 (s, 3H), 1.87 (s, 6H). **^{19}F NMR** (376 MHz, C$_6$D$_6$, 25 ºC): δ_F −61.0, −132.9. **^{13}C NMR** (126 MHz, C$_6$D$_6$, 25 ºC): δ_C 166.7, 164.7, 163.1, 153.3, 151.4, 150.3, 143.7, 136.3, 125.2 (d, *J* = 30.8 Hz), 122.5 (d, *J* = 7.3 Hz), 122.0, 121.8 (d, *J* = 24.0 Hz), 119.1, 118.6 (d, *J* = 7.7 Hz), 115.9 (d, *J* = 21.5 Hz), 105.9, 54.8, 37.7, 25.0. **HRMS** *m/z* (ESI$^+$): calcd for C$_{23}$H$_{23}$F$_4$N$_3$NiO (M$^+$): 491.1131; found: 491.1683.

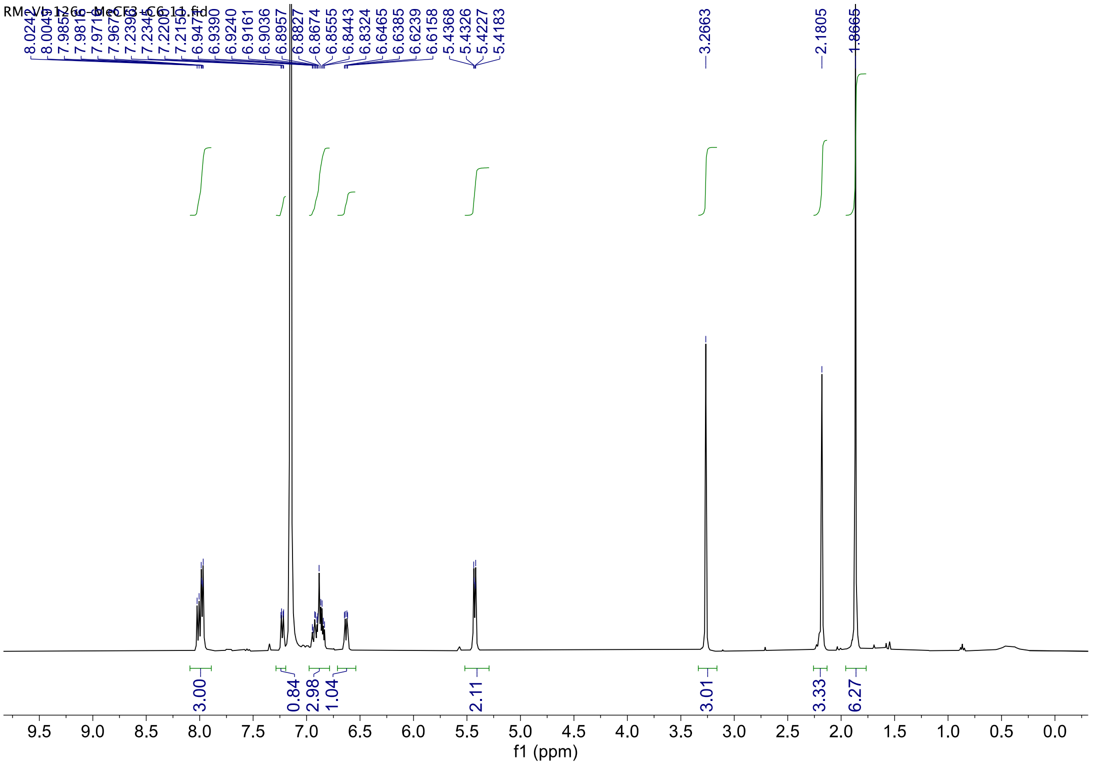

Figure S25. ^{1}H NMR spectrum (400 MHz, C_6D_6) of ($Me^{F}FI$)Ni(2-Me-4-CF_3-C_6H_3)(DMAP).

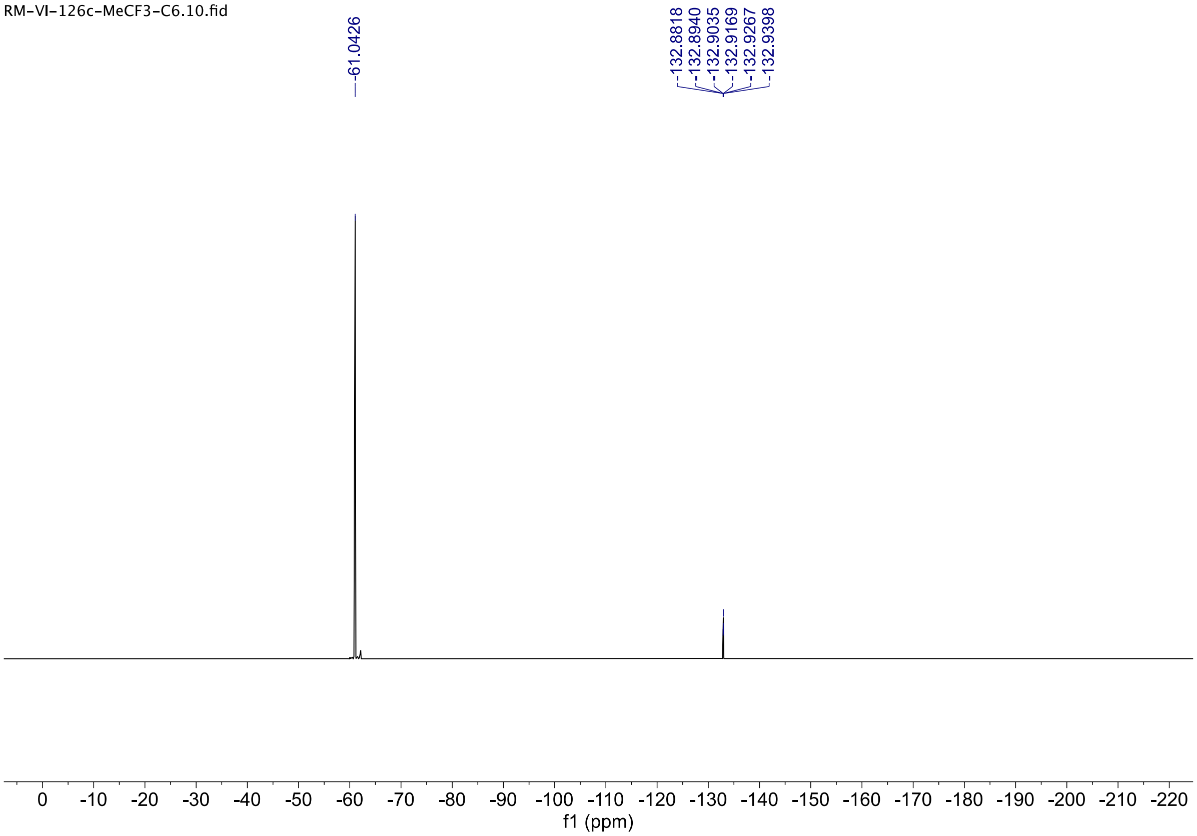

Figure S26. ^{19}F NMR spectrum (376 MHz, C_6D_6) of ($Me^{F}FI$)Ni(2-Me-4-CF_3-C_6H_3)(DMAP).

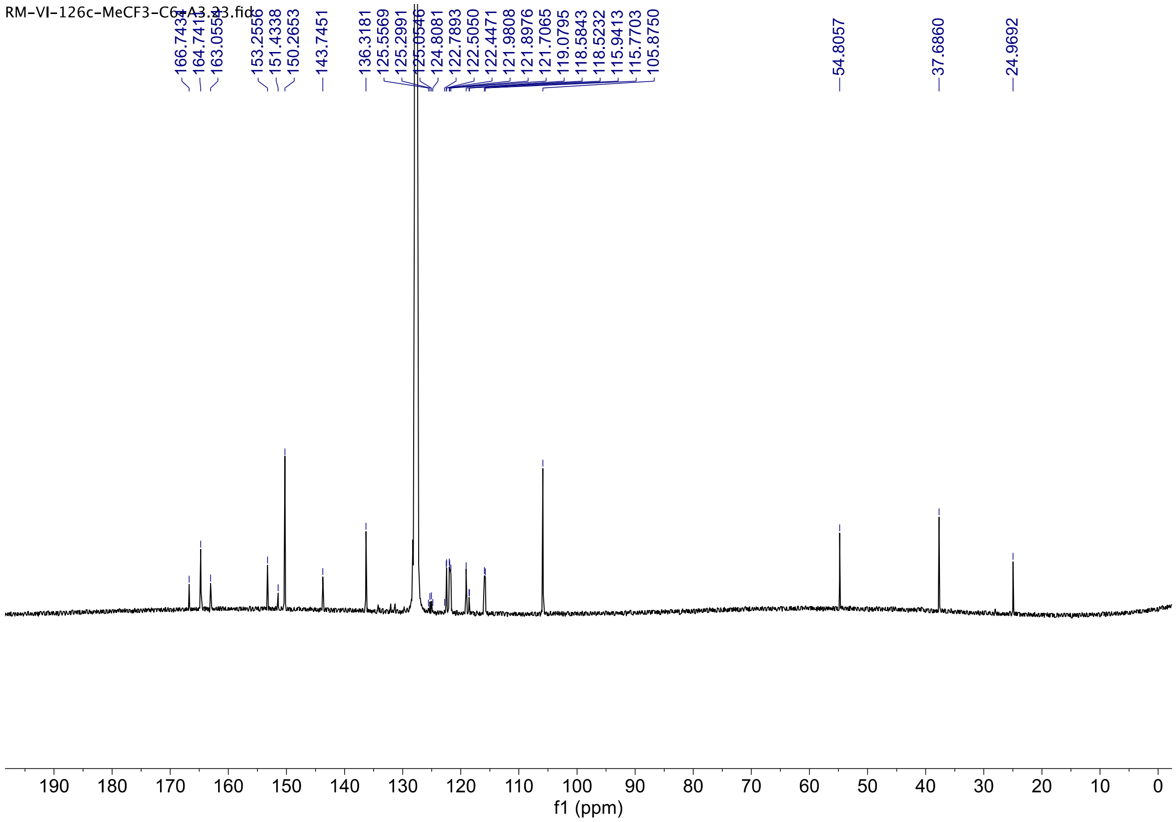

Figure S27. ^{13}C NMR spectrum (126 MHz, C_6D_6, 25 ºC) of $(Me^{F}FI)Ni(2\text{-}Me\text{-}4\text{-}CF_3\text{-}C_6H_3)(DMAP)$.

III. Experiments for Alkyl Radical Generation and Capture

Stability of Nickel Complexes and Aryl Exchange

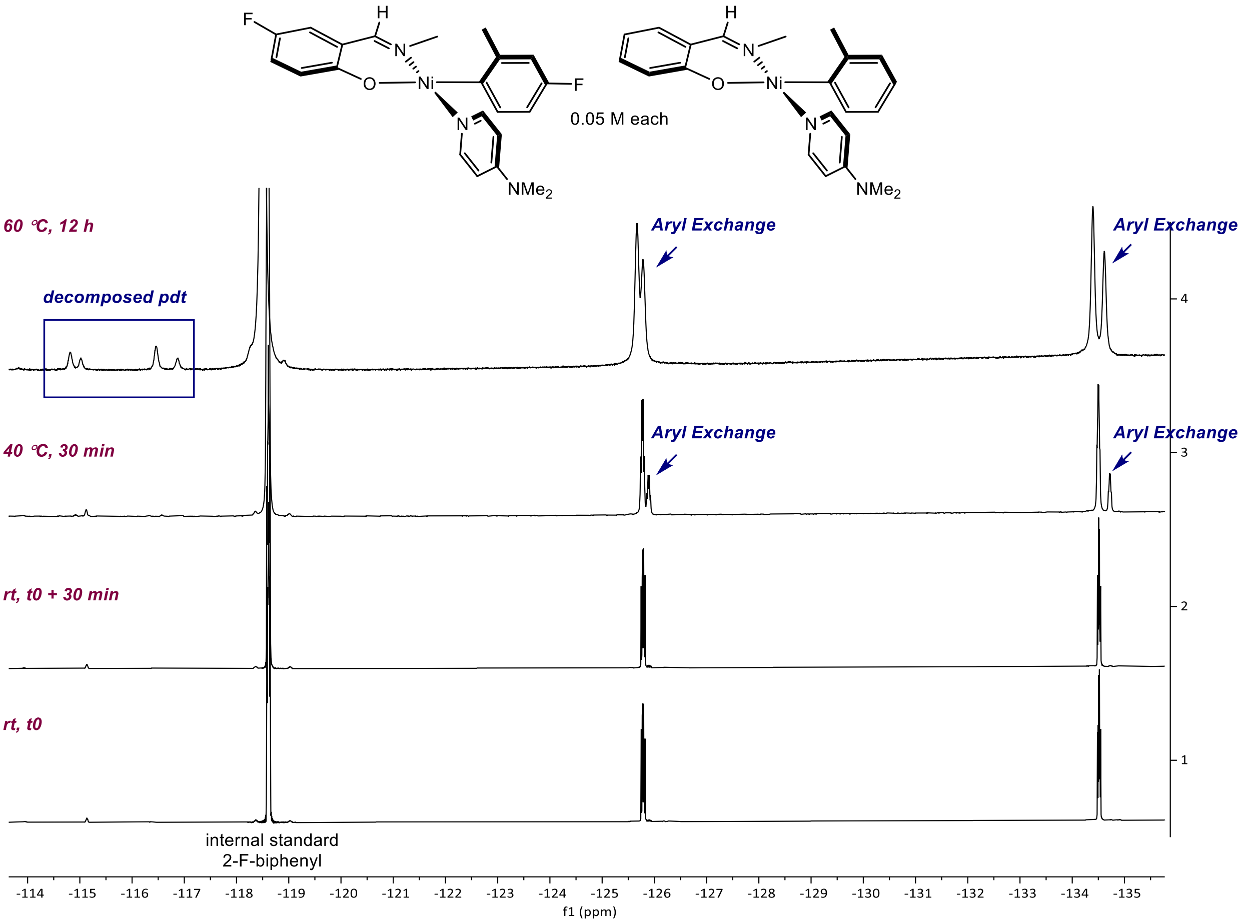

Figure S28. Stability and aryl exchange of FI-Nickel complexes in C_6D_6 monitored by ^{19}F NMR.

Nickel complexes are stable in C_6D_6 solution at room temperature with trace amount of ligand exchange at room temperature over 1-2 hours. Heating the mixture to 40 ˚C (aluminum block) for 30 minutes led to aryl exchange with 16% loss of mass balance. Heating the mixture to 60 ˚C (aluminum block) for 12 hours lead to further aryl exchange and loss of mass balance with formation of unidentified decomposition product.

Alkyl Radical Generation with $Ni(cod)_2$ and Alkyl Bromide

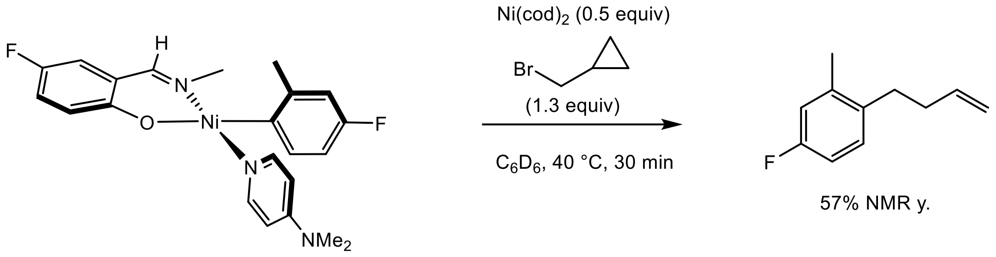

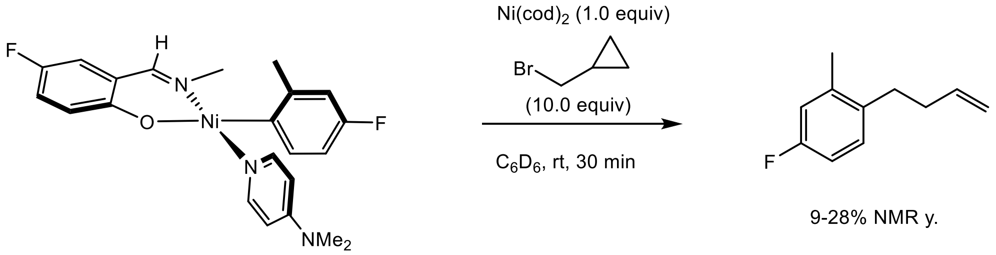

Reaction between cyclopropylmethyl bromide and $Ni(cod)_2$ was attempted for radical generation. Reaction at 40 ˚C (aluminum block) lead to 57% NMR yield of C-C bond formation product. Reaction at room temperature was much more sluggish at room temperature, and increasing $Ni(cod)_2$ loading to 1.0 equivalent as well as alkyl halide loading to 10.0 equivalents led to a 9-28% yield of C-C bond formation product at room temperature for 30 minutes.

Alkyl Radical Generation with Ti(III) Reagents

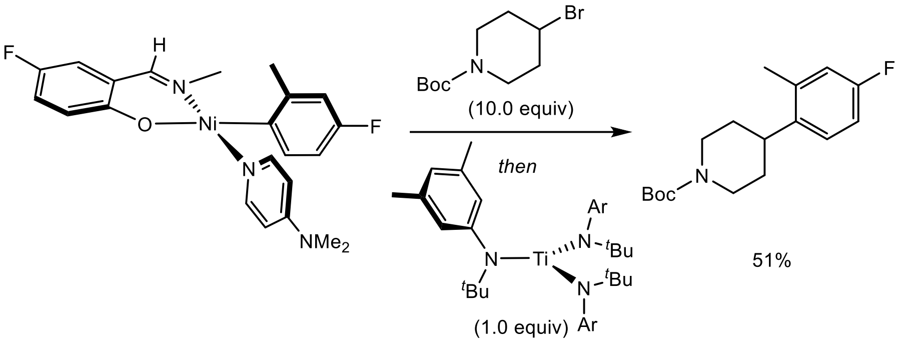

In a N_2-filled glovebox, a 20-mL vial was charged with nickel complex (Me^{F}FI)Ni(2-Me-4-F-C_6H_3)(DMAP) (22 mg, 0.05 mmol, 1.0 equiv), *tert*-butyl 4-bromopiperidine-1-carboxylate (132 mg, 0.5 mmol, 10.0 equiv) and 1 mL toluene. The solution was frozen in a cold well with liquid N_2 bath, and Ti(III)-tris-anilide complex (28 mg, 0.05 mmol, 1.0 equiv) was added to the thawing solution. The reaction was allowed to warm to room temperature and further stirred for 30 min. Upon completion, internal standard C_6F_6 (5 μL) was added and a portion of the reaction mixture was diluted with C_6D_6 for ^{19}F NMR analysis.

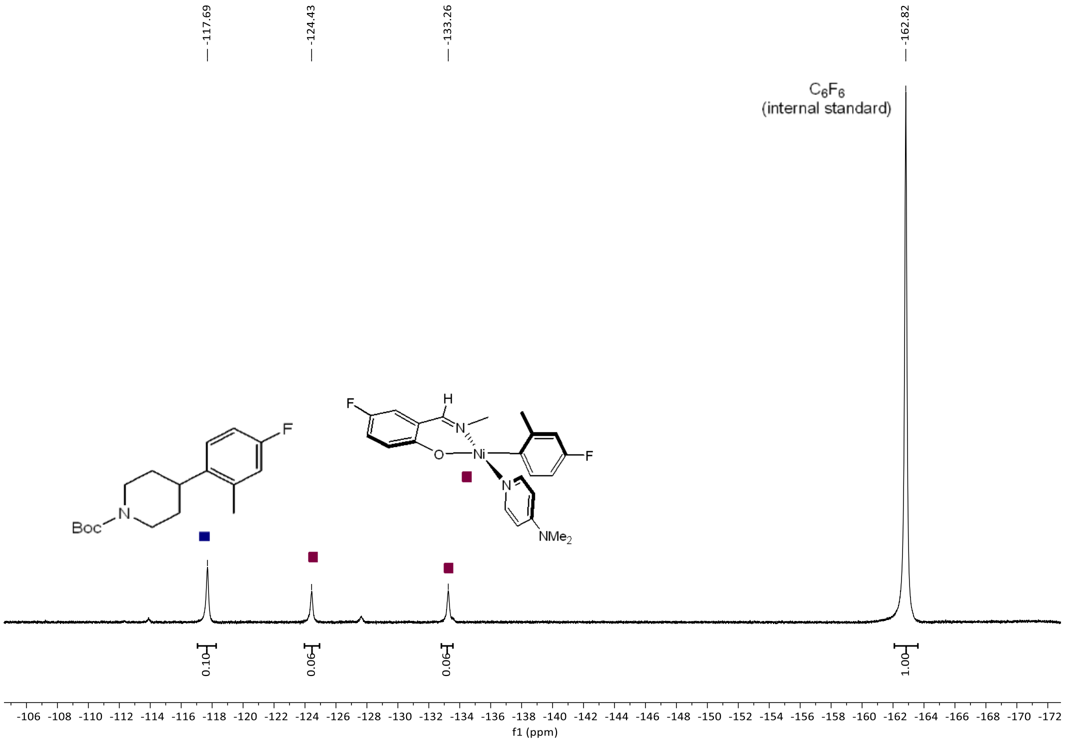

Figure S29. Crude reaction mixture with direct addition of **Ti-1** by ^{19}F NMR.

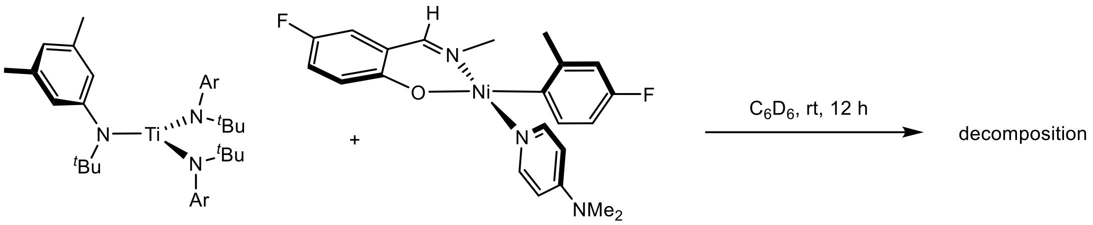

In a N_2-filled glovebox, a J. Young NMR tube was charged with (Me^{F}FI)Ni(2-Me-4-F-C_6H_3)(DMAP) (4 mg, 0.01 mmol, 1.0 equiv), Ti(III)-tris-anilide (6 mg, 0.01 mmol, 1.0 equiv) and 0.5 mL C_6D_6 to give a brown solution. The sample was monitored by ^{19F} NMR and decomposition of (Me^{F}FI)Ni(2-Me-4-F-C_6H_3)(DMAP) complex was observed.

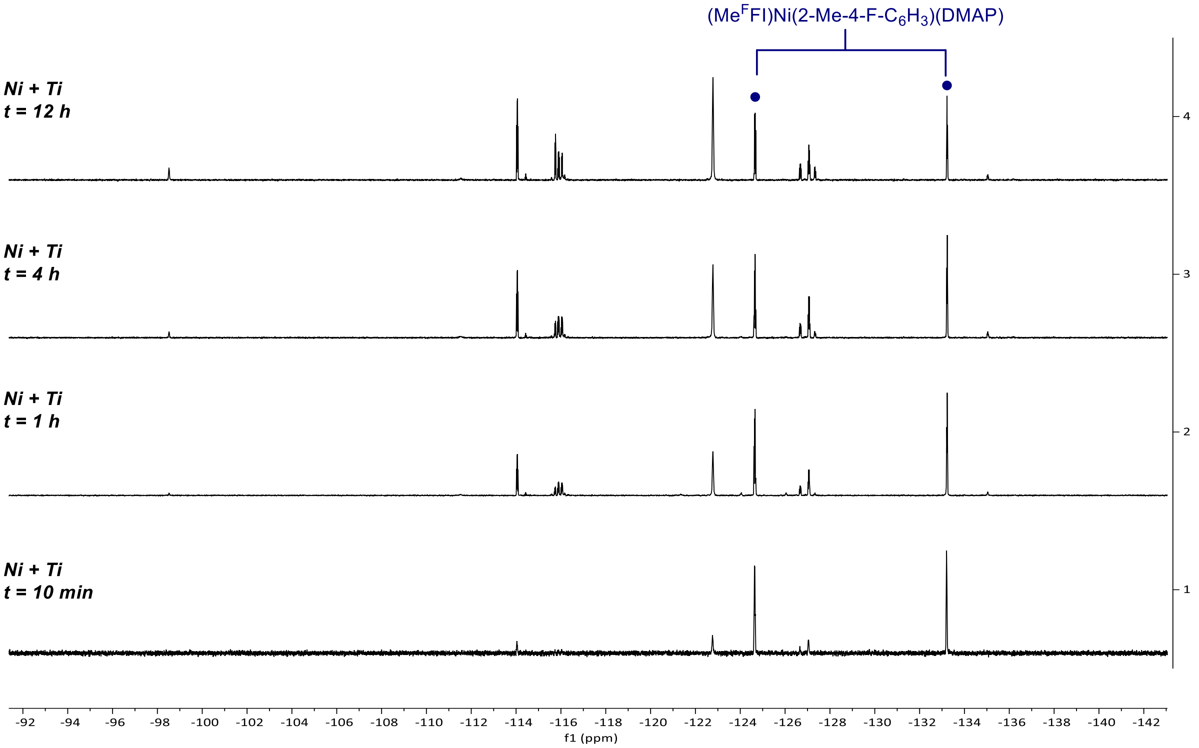

Figure S30. Side reaction between **Ti-1** and **Ni-1** monitored by ^{19}F NMR.

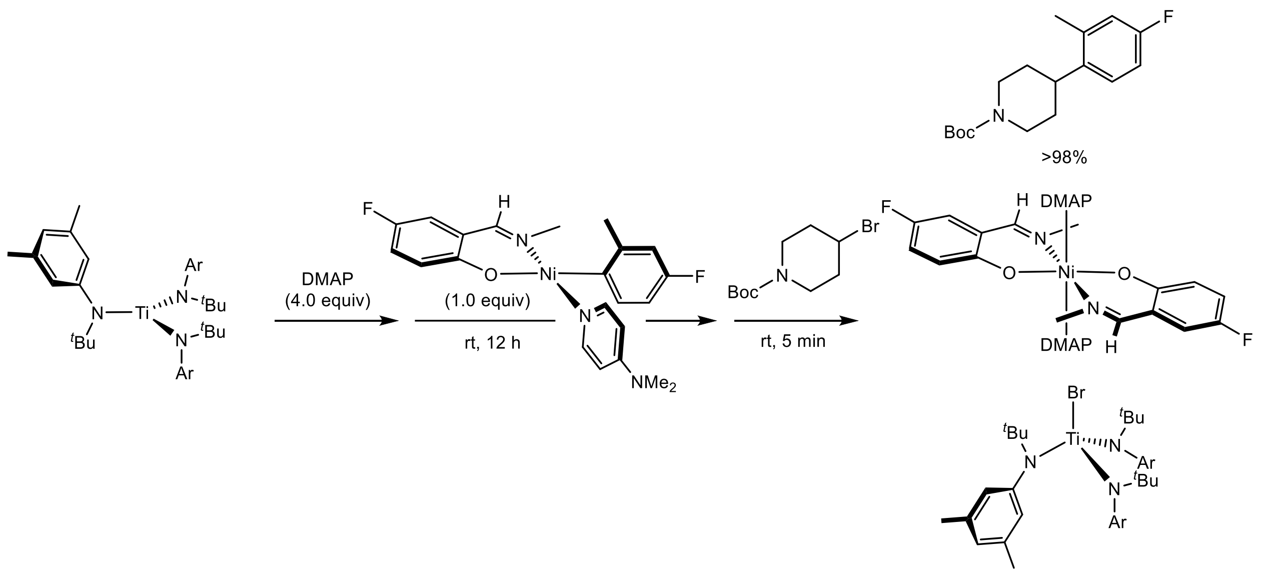

In a N_2-filled glovebox, a J. Young NMR tube was charged with Ti(III)-tris-anilide (6 mg, 0.01 mmol, 1.0 equiv) and 0.5 mL C_6D_6 to give a forest green solution. DMAP (5 mg, 0.04 mmol, 4.0 equiv) was added and the solution quickly turned into dark purple. (Me^FFI)Ni(2-Me-4-F-C_6H_3)(DMAP) (4 mg, 0.01 mmol, 1.0 equiv) was added and the sample was monitored by ^{19}F NMR. No decomposition of (Me^FFI)Ni(2-Me-4-F-C_6H_3)(DMAP) complex was observed after 12 h. The J. Young NMR tube was then transferred back to the glovebox and *tert*-butyl 4-bromopiperidine-1-carboxylate (~ 10 mg, ~0.4 mmol, ~ 10.0 equiv) was added. The dark purple solution quickly turned into dark orange and yellow solid precipitated in 5 min, ^{19}F NMR indicate full conversion of (Me^FFI)Ni(2-Me-4-F-C_6H_3)(DMAP) and clean formation of *tert*-butyl 4-(4-fluoro-2-methylphenyl)piperidine-1-carboxylate. The sample was transferred back to the glovebox and was filtered through filter paper. The filtrated was allowed to slowly evaporate to give orange-red crystal of Ti(III)-Br-tris-anilide suitable for X-ray diffraction. The filter residue was redissolved in benzene, and was allowed to slowly evaporate to give yellow crystal of (Me^FFI)$_2$Ni(DMAP)$_2$ suitable for X-ray diffraction.

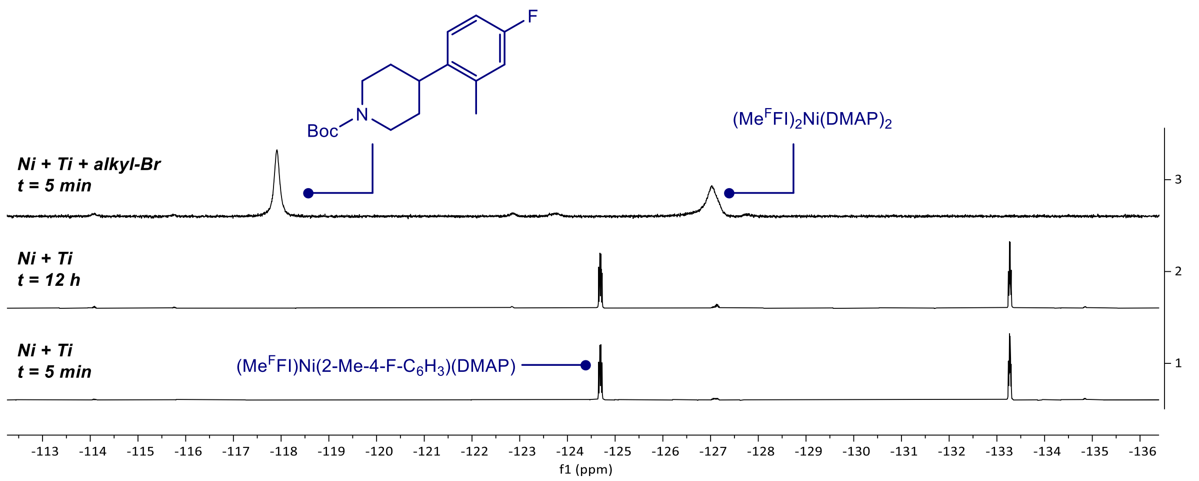

Figure S31. Radical generation with **Ti-2** and radical capture by **Ni-1** monitored by ^{19}F NMR.

Reaction between **Ti-2** and alkyl bromide was monitored by EPR, in which only a decay signal attributed to **Ti-2** was observed and no evidence of a signal from free radical EPR signal. This is likely due to the short life time of an alkyl radical ($T_{1/2}$ ~ 10^{-4}~10^{-5} seconds), which is much shorter than halide abstraction with alkyl bromide (~minutes) and therefore direction observation of a free radical before decomposition can be challenging.

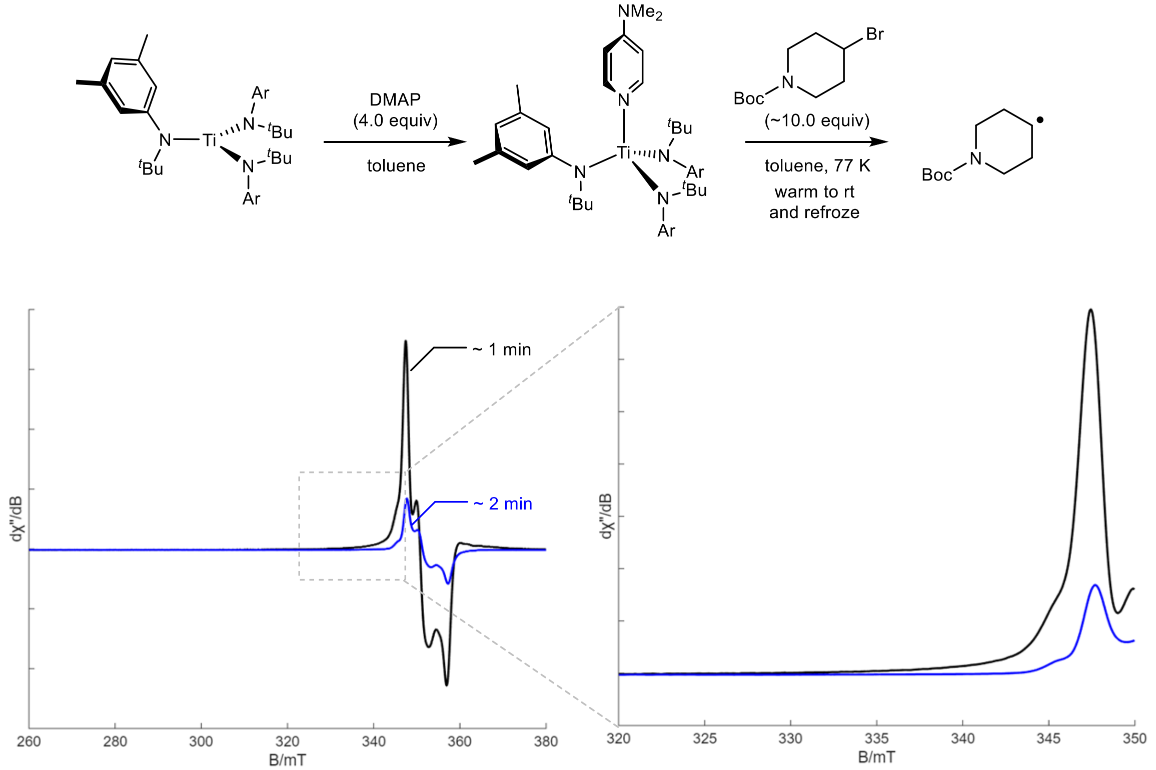

Figure S32. Attempts for directed observation of alkyl radical

However, addition of excess amounts of an alkyl halide as well as one equivalence of a spin trap reagent in one pot at room temperature allow direct detection of trapped, persistent radicals characterized by EPR, which provide support for alkyl radical generation. This also suggest that alkyl radical generation with **Ti-2** is independent from nickel complexes.

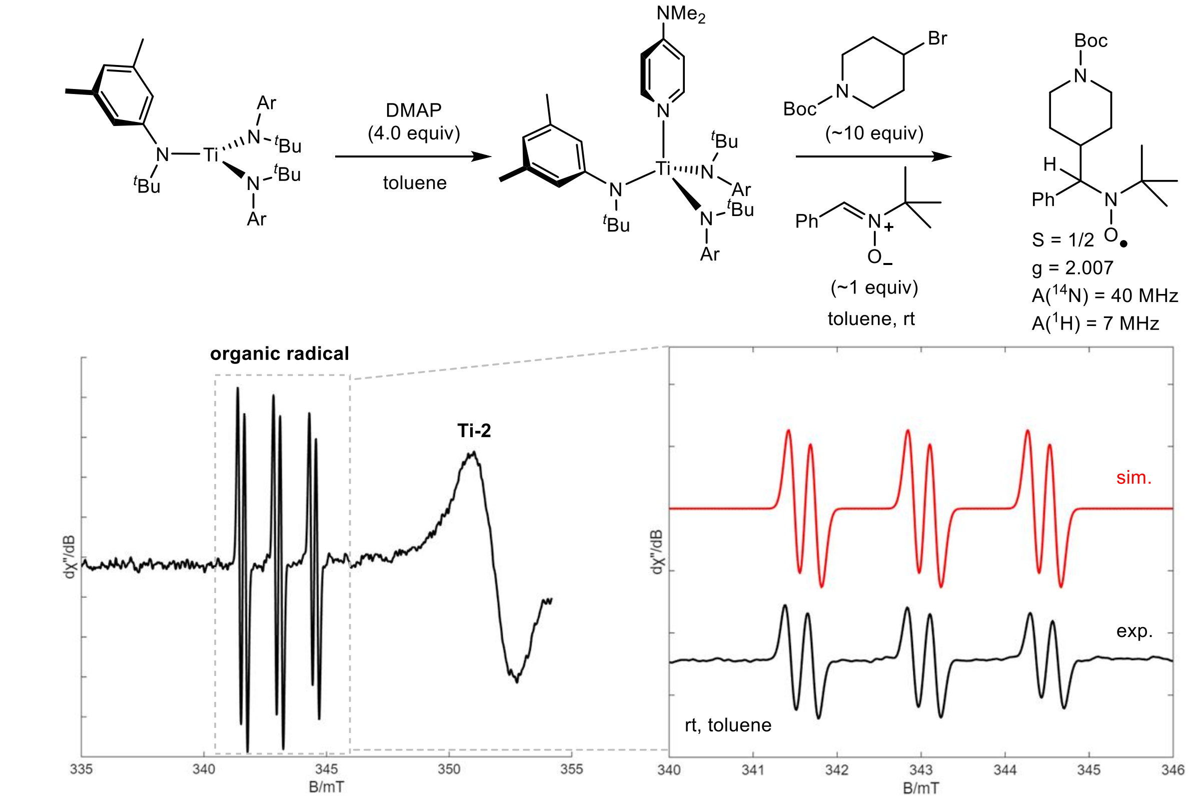

Figure S33. Evidence of alkyl radical generation by spin trap reagent PBN

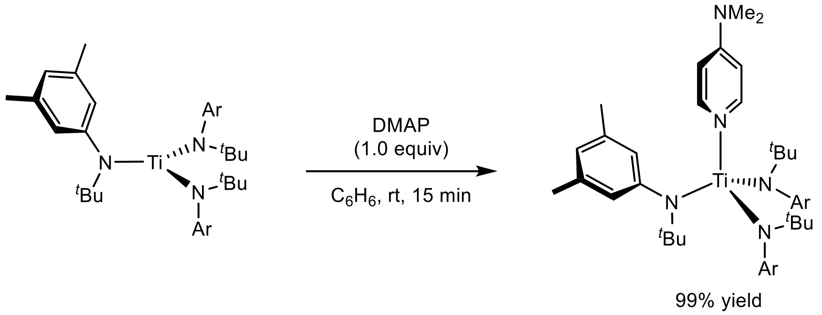

DMAP-Ti(III)-tris-anilide: In a N_2-filled glovebox, a 20-mL septum-capped vial with a stir bar was charged with Ti(III)-tris-anilide (115 mg, 0.2 mmol, 1.0 equiv), DMAP (24 mg, 0.2 mmol, 1.0 equiv) and benzene (1.0 mL). The reaction mixture was allowed to stir at room temperature for 15 min, and solvent was removed to afford product as a dark purple solid (140 mg, 0.2 mmol, 99% yield). The compound was redissolved in Et_2O, and was allowed to slowly evaporate to give dark-purple crystal of suitable for X-ray diffraction.

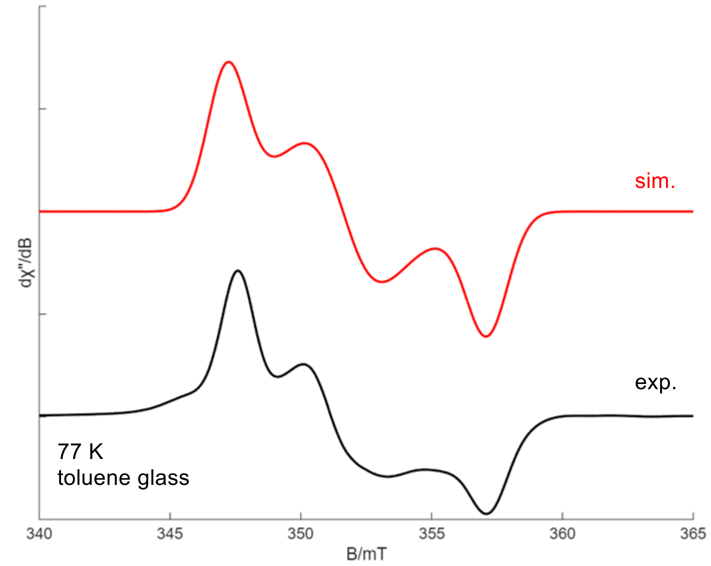

Figure S34. EPR spectrum of [(3,5-xylyl)^{t}BuN]$_3$Ti(DMAP) (**Ti-2**) at 77K in toluene glass

EPR spectrum were collected at 77 K in toluene glass. Experimental parameters: microwave frequency = 9.646 GHz, power = 0.3162 mW, and modulation amplitude = 4.000 G.

Simulation parameters:

S = 1/2, g_1 = 1.99, g_2 = 1.96, g_3 = 1.93, and g_{strain} = (0.01, 0.02, 0.01);

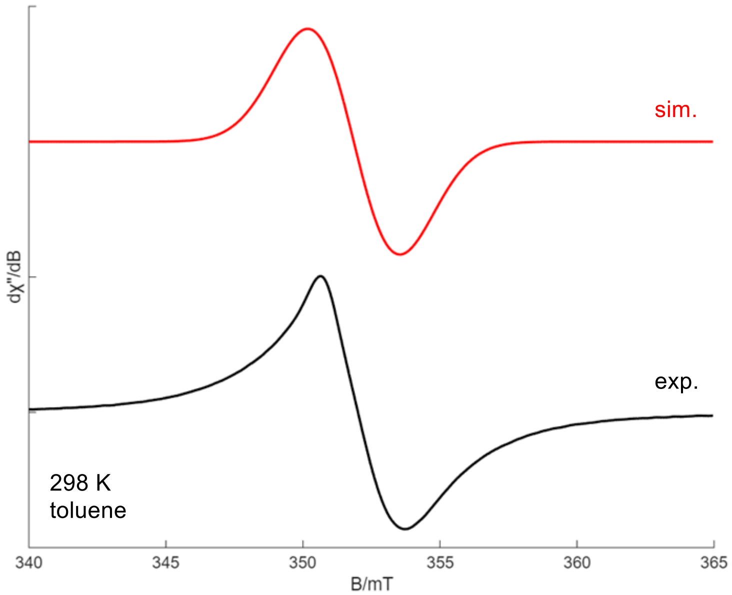

Figure S35. EPR spectrum of [(3,5-xylyl)^{t}BuN]$_3$Ti(DMAP) (**Ti-2**) at 298K in toluene

EPR spectrum were collected at 298 K in toluene. Experimental parameters: microwave frequency = 9.633 GHz, power = 0.3162 mW, and modulation amplitude = 4.000 G.

Simulation parameters:

S = 1/2, g_1 = 1.96, g_2 = 1.96, g_3 = 1.96, and g_{strain} = (0.02, 0.02, 0.02);

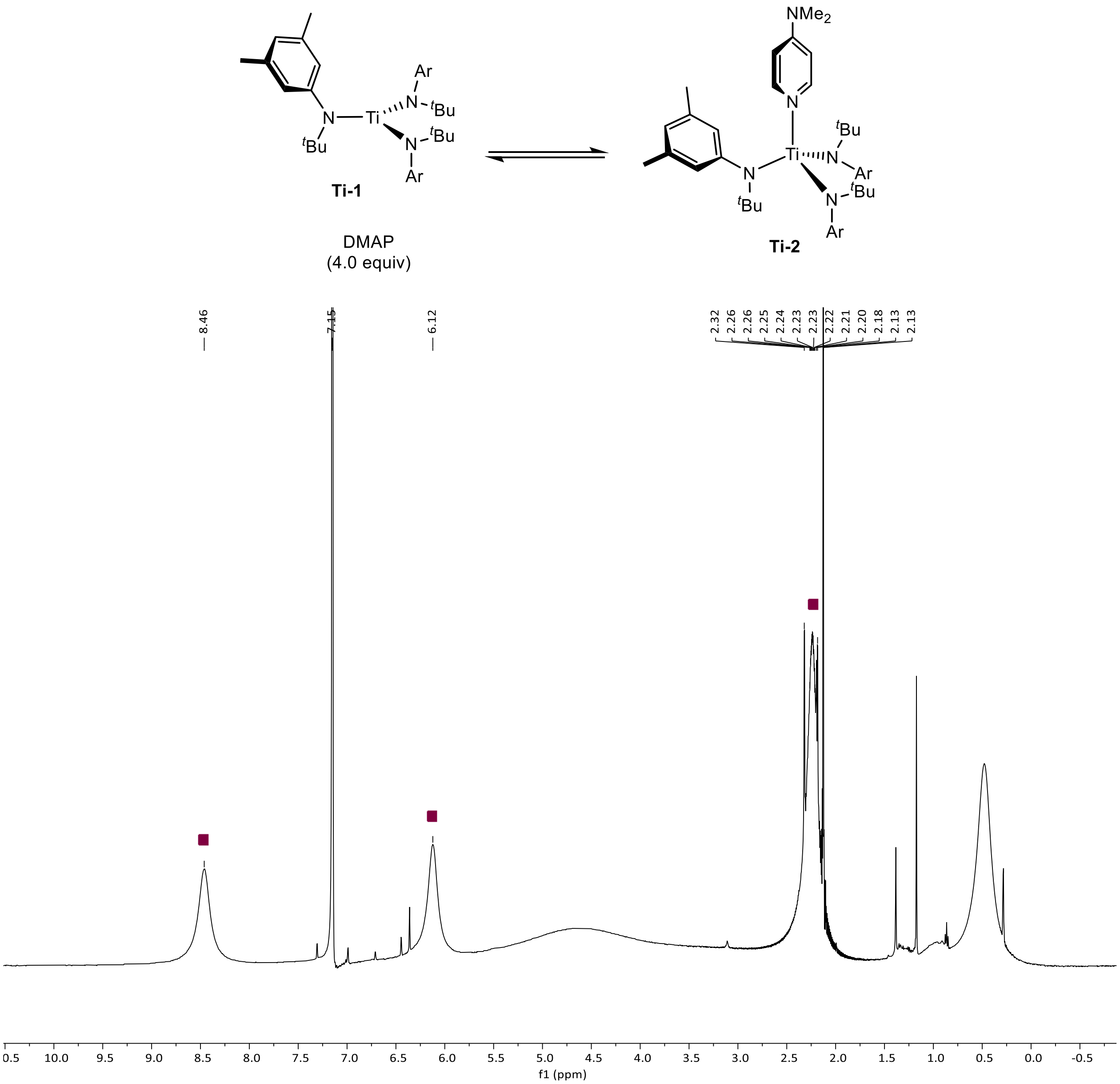

Figure S36. ^{1}H NMR spectrum of [(3,5-xylyl)^{t}BuN]$_3$Ti(DMAP) (**Ti-2**) and excess DMAP at room temperature in C_6D_6.

Broadening of ^{1}H NMR singles for DMAP motif was observed in ^{1}H NMR. These observations are consistent with reversible DMAP coordination with **Ti-1**, consistent with observations by Cummins and co-workers.[5]

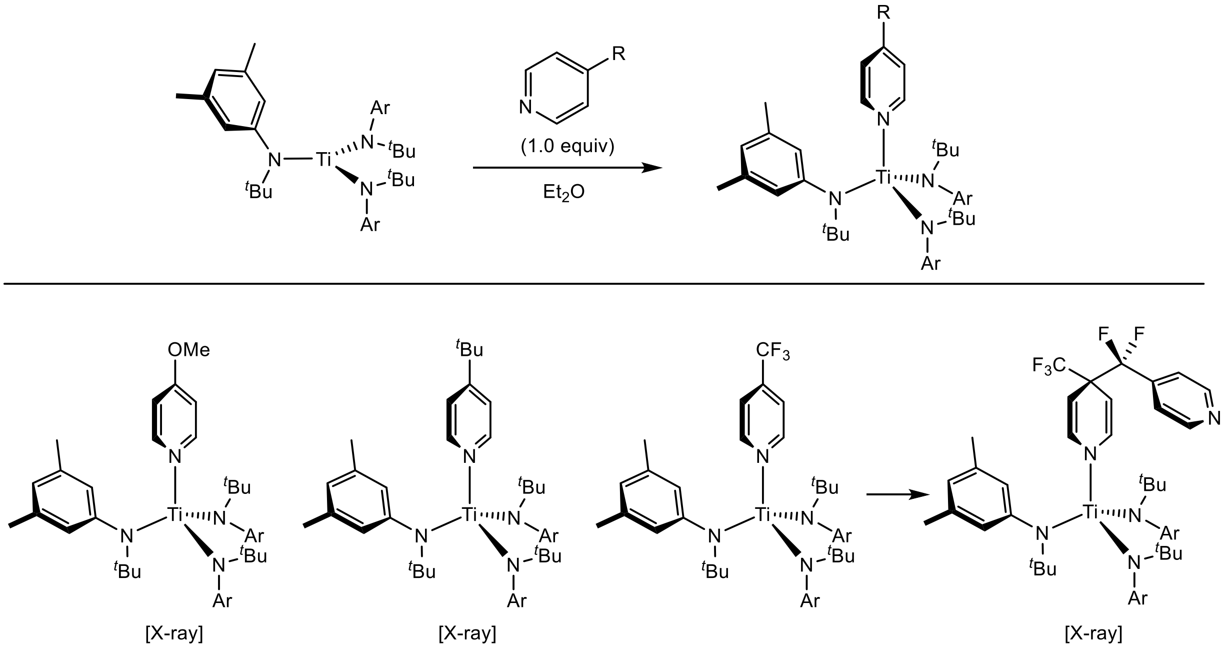

Isolation of 4-R-pyridine-Ti(III)-tris-anilide: In a N_2-filled glovebox, a 20-mL septum-capped vial with a stir bar was charged with Ti(III)-tris-anilide (1.0 equiv), pyridine derivatives (1.0 equiv) and Et_2O (1.0 mL). The solution was allowed to slowly evaporate to give crystal of suitable for X-ray diffraction.

IV Scope and Characterization of C-C Bond Formation Products

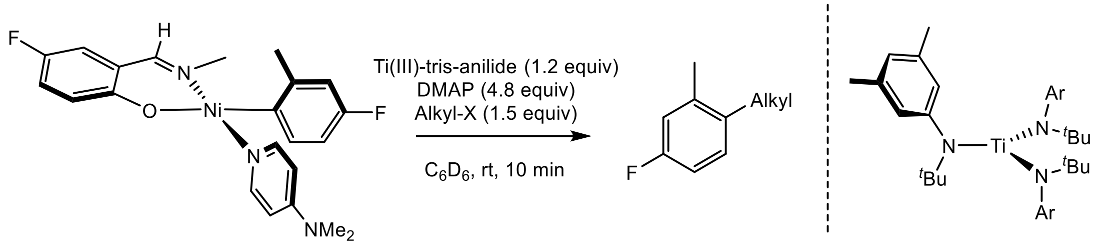

General Procedure C: In a N_2-filled glovebox, a 20-mL vial with a stir bar was charged with Ti(III)-tris-anilide (0.12 mmol 1.0 equiv), DMAP (0.48 mmol, 1.0 equiv) and C_6D_6 (1.0 mL). (Me^{F}FI)Ni(2-Me-4-F-C_6H_3)(DMAP) (44 mg, 0.1 mmol, 1.0 equiv) followed by addition of alkyl-halide (0.15 mmol, 1.5 equiv). The reaction mixture was allowed to stir at room temperature for 10 min. Upon completion, C_6F_6 (5 μL) was added and the reaction mixture was analyzed by ^{19}F NMR. The mixture was further quenched with HCl (2.0 M, aq.) and extracted with Et_2O. The organic layers were combined and concentrated under reduced pressure. The desired product was isolated by silica gel column chromatography.

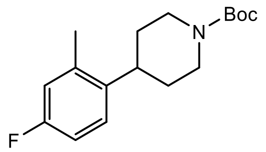

***tert*-butyl 4-(4-fluoro-2-methylphenyl)piperidine-1-carboxylate**

With alkyl-Br as electrophile: The title compound was form in 88% yield by ^{19}F NMR analysis with >98% conversion of (Me^{F}FI)Ni(2-Me-4-F-C_6H_3)(DMAP), and was isolated as a colorless oil (29 mg, 0.1 mmol, 99% yield).

With alkyl-Cl as electrophile: The reaction was allowed to stir at room temperature for 36 h, and the title compound was form in 44% yield with >98% conversion of (Me^{F}FI)Ni(2-Me-4-F-C_6H_3)(DMAP) by ^{19}F NMR analysis.

With alkyl-RAE as electrophile: The title compound was form in 42% yield with 58% conversion of (Me^{F}FI)Ni(2-Me-4-F-C_6H_3)(DMAP) by ^{19}F NMR analysis,

^{1}H NMR (500 MHz, $CDCl_3$, 25 °C): δ 7.25 – 7.06 (m, 1H), 7.01 – 6.81 (m, 2H), 4.55 – 4.13 (m, 2H), 3.14 – 2.74 (m, 3H), 2.59 – 2.25 (m, 3H), 1.90 – 1.75 (m, 4H), 1.56 (s, 9H). **^{19}F NMR** (471 MHz, $CDCl_3$, 25 °C): -117.87 (t, *J* = 8.5 Hz).**^{13}C NMR** (126 MHz, $CDCl_3$, 25 °C): δ 161.0 (d, *J* = 243.6 Hz), 155.0, 139.5, 137.4 (d, *J* = 7.2 Hz), 126.9 (d, *J* = 8.1 Hz), 117.0 (d, *J* = 20.6 Hz), 112.9 (d, *J* = 20.4 Hz), 79.6, 44.7 (br), 38.0, 32.7, 28.6, 19.5. **HRMS** *m/z* (ESI^+): calcd for $C_{13}H_{17}FNO_2$ ($M+H^+-C_4H_8$): 238.1238; found: 238.1241.

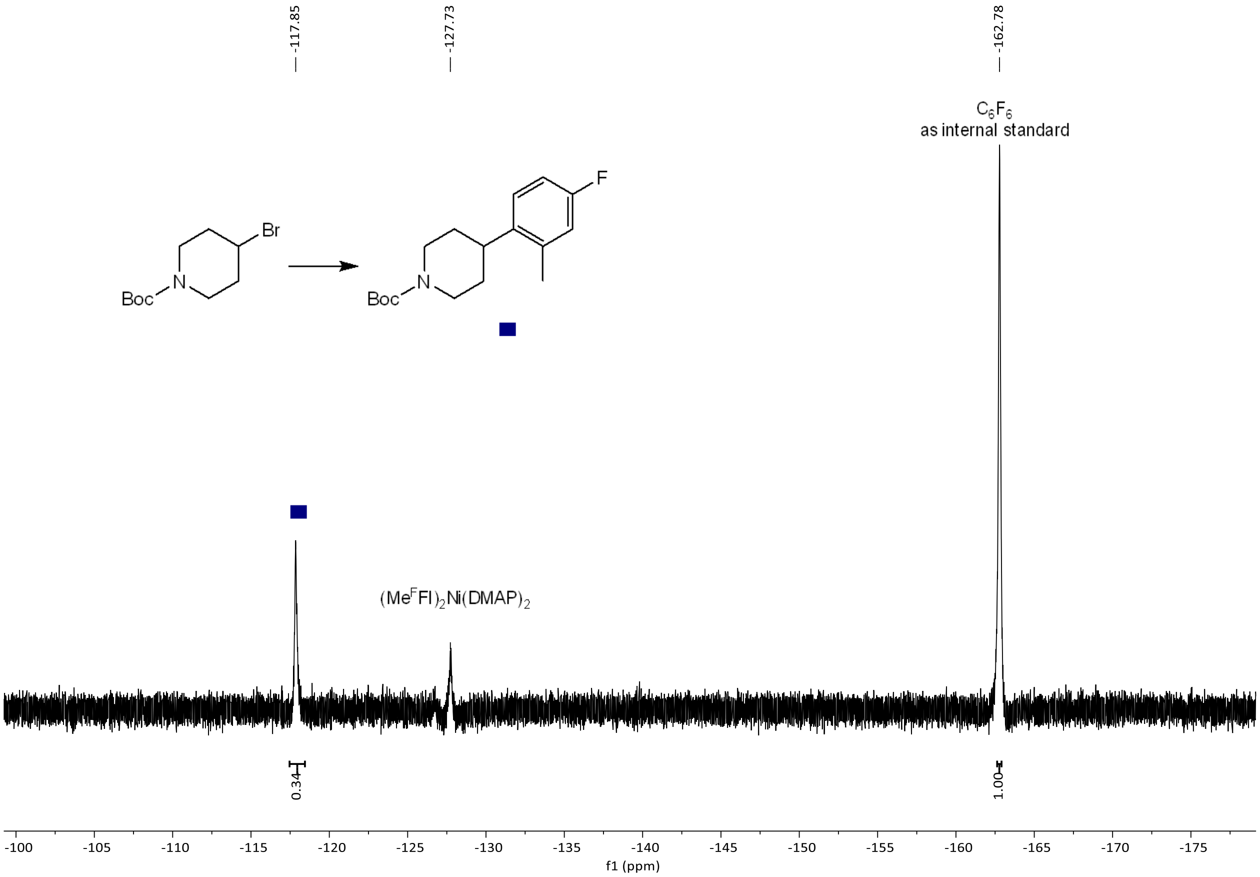

Figure S37. Crude reaction mixture with *tert*-butyl 4-bromopiperidine-1-carboxylate as electrophile monitored by ^{19}F NMR.

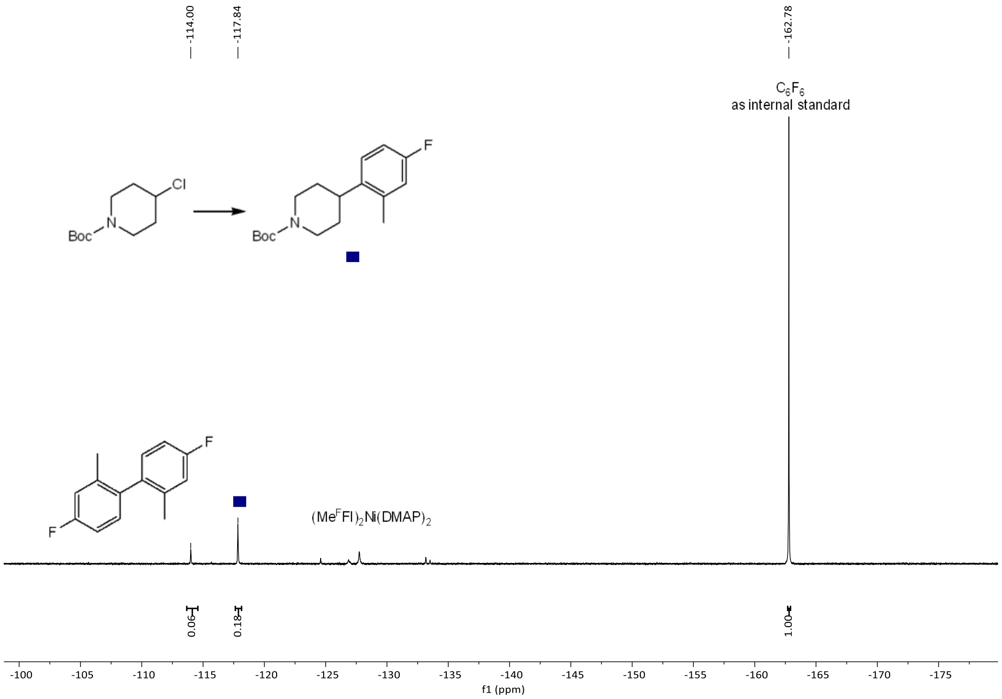

Figure S38. Crude reaction mixture with *tert*-butyl 4-chloropiperidine-1-carboxylate as electrophile monitored by ^{19}F NMR.

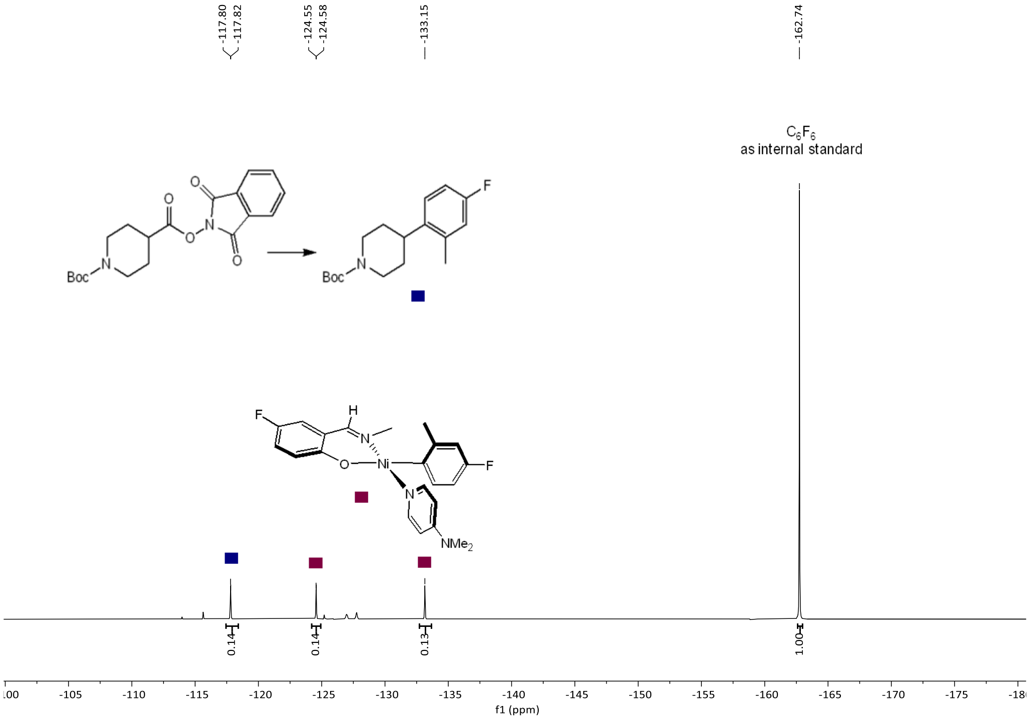

Figure S39. Crude reaction mixture with *tert*-butyl 4-(DHP)piperidine-1-carboxylate as electrophile monitored by ^{19}F NMR.

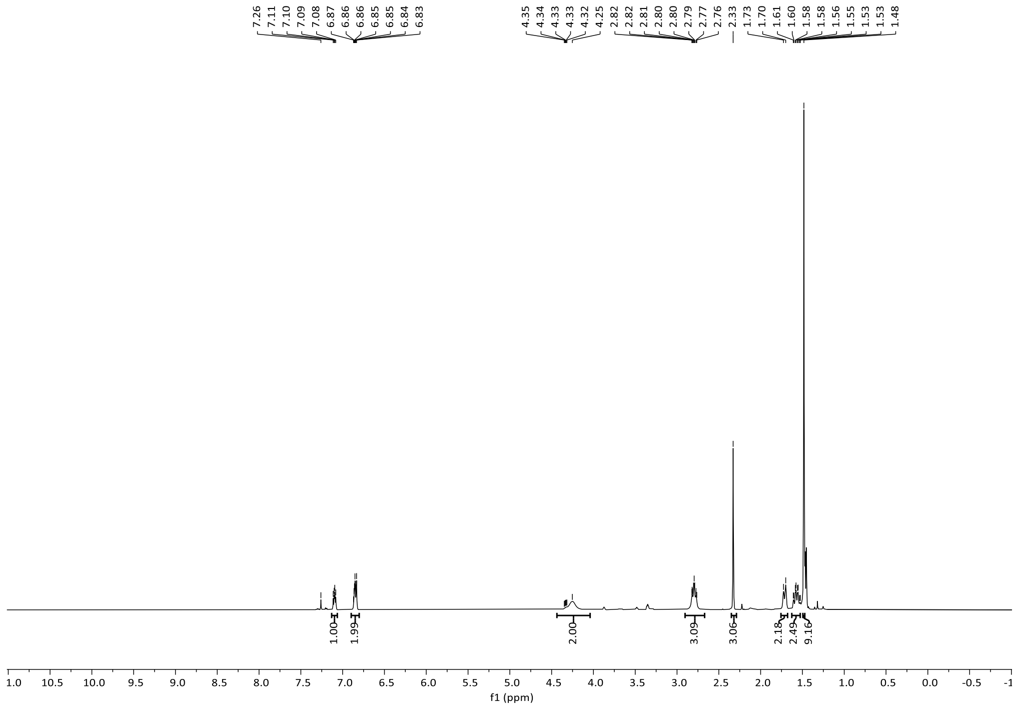

Figure S40. ^{1}H NMR spectrum (500 MHz, $CDCl_3$) of *tert*-butyl 4-(4-fluoro-2-methylphenyl)piperidine-1-carboxylate

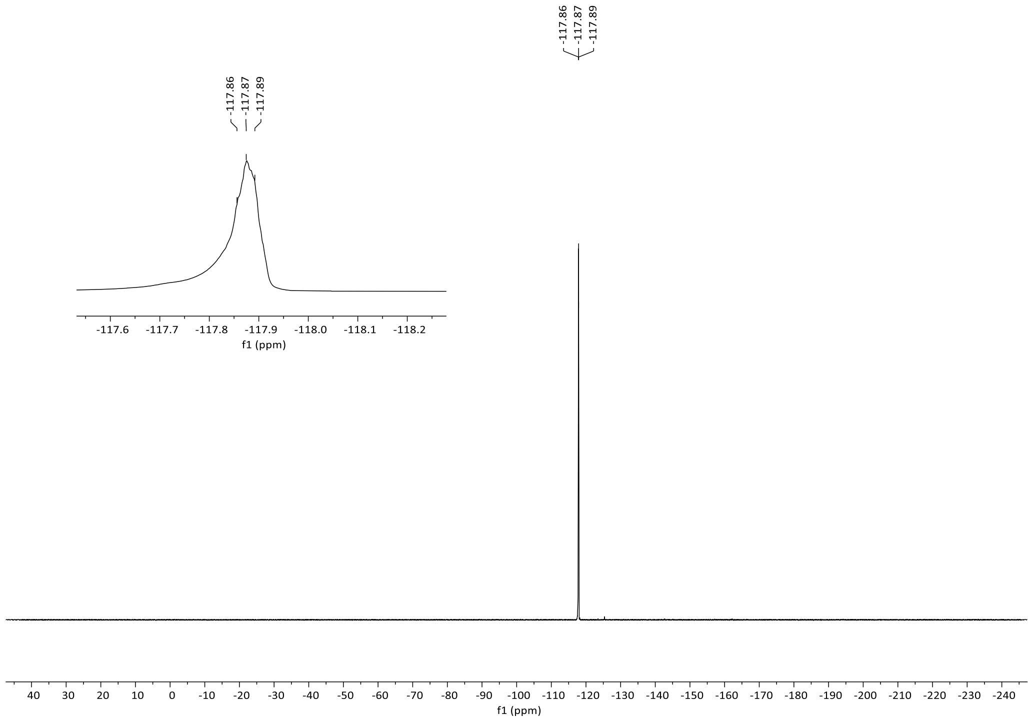

Figure S41. ^{19}F NMR spectrum (471 MHz, $CDCl_3$) of *tert*-butyl 4-(4-fluoro-2-methylphenyl)piperidine-1-carboxylate

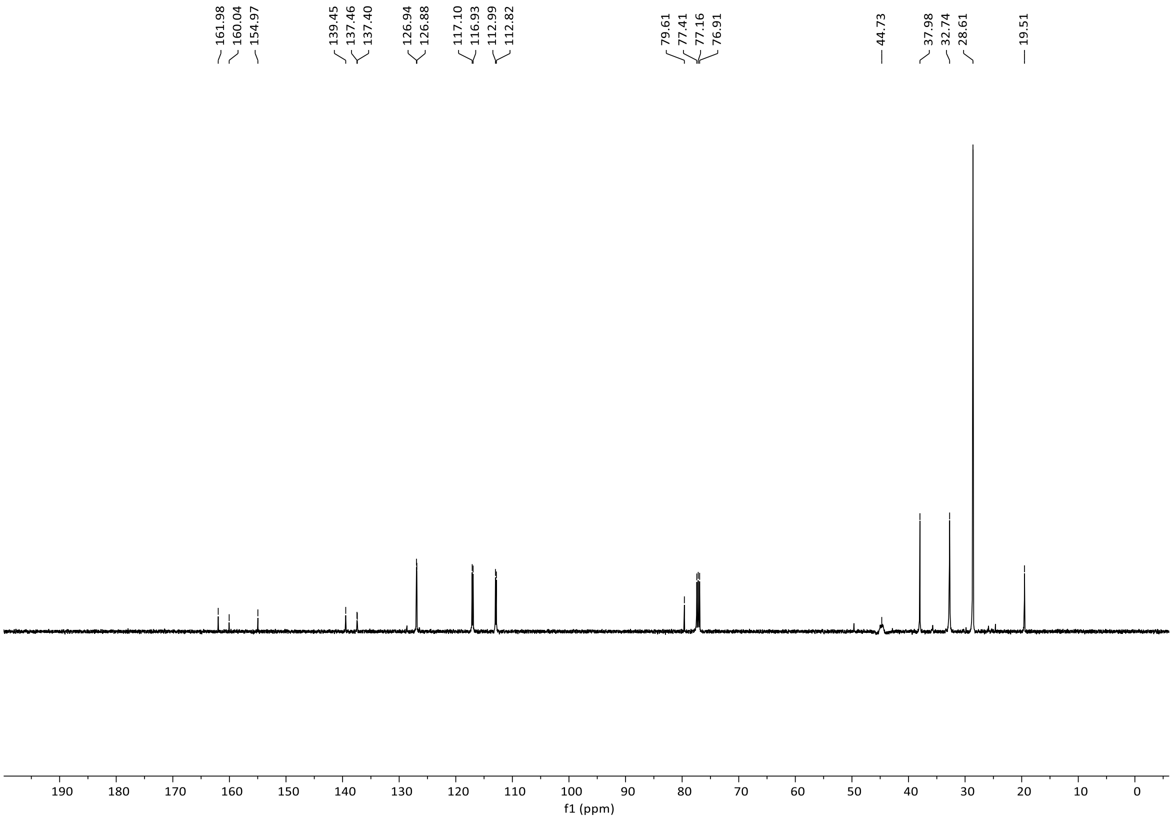

Figure S42. ^{13}C NMR spectrum (126 MHz, $CDCl_3$) of *tert*-butyl 4-(4-fluoro-2-methylphenyl)piperidine-1-carboxylate

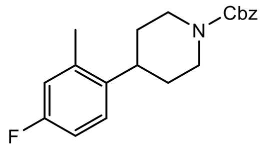

benzyl 4-(4-fluoro-2-methylphenyl)piperidine-1-carboxylate

With alkyl-I as electrophile: The title compound was form in 86% yield with >98% conversion of (Me^FFI)Ni(2-Me-4-F-C_6H_3)(DMAP) by ^{19}F NMR analysis, and was isolated as a colorless oil (52 mg, 0.086 mmol, 86% yield). **^1H NMR** (500 MHz, $CDCl_3$, 25 °C): δ 7.43 – 7.38 (m, 5H), 7.37 – 7.33 (m, 1H), 7.17 – 7.06 (m, 1H), 6.96 – 6.82 (m, 2H), 5.22 – 5.16 (m, 2H), 4.54 – 4.20 (m, 2H), 3.09 – 2.67 (m, 3H), 2.36 (d, *J* = 2.0 Hz, 3H), 1.92 – 1.70 (m, 2H), 1.71 – 1.55 (m, 2H). **^9F NMR** (471 MHz, CD6Cl_3, 25 °C): δ -117.70 (td, *J* = 9.0, 5.8 Hz). **^{13}C NMR** (126 MHz, $CDCl_3$, 25 °C): δ 161.0 (d, *J* = 243.9 Hz), 155.4, 139.2 (d, *J* = 3.1 Hz), 137.4 (d, *J* = 7.4 Hz), 137.0, 128.6, 128.1, 128.0, 126.9 (d, *J* = 8.2 Hz), 117.1 (d, *J* = 20.7 Hz), 113.0 (d, *J* = 20.7 Hz), 67.2, 44.9, 37.8, 32.6, 19.5 (d, *J* = 1.5 Hz). **HRMS** *m/z* (ESI^+): calcd for $C_{20}H_{23}FNO_2$ ($M+H^+$): 328.1707; found: 328.1711.

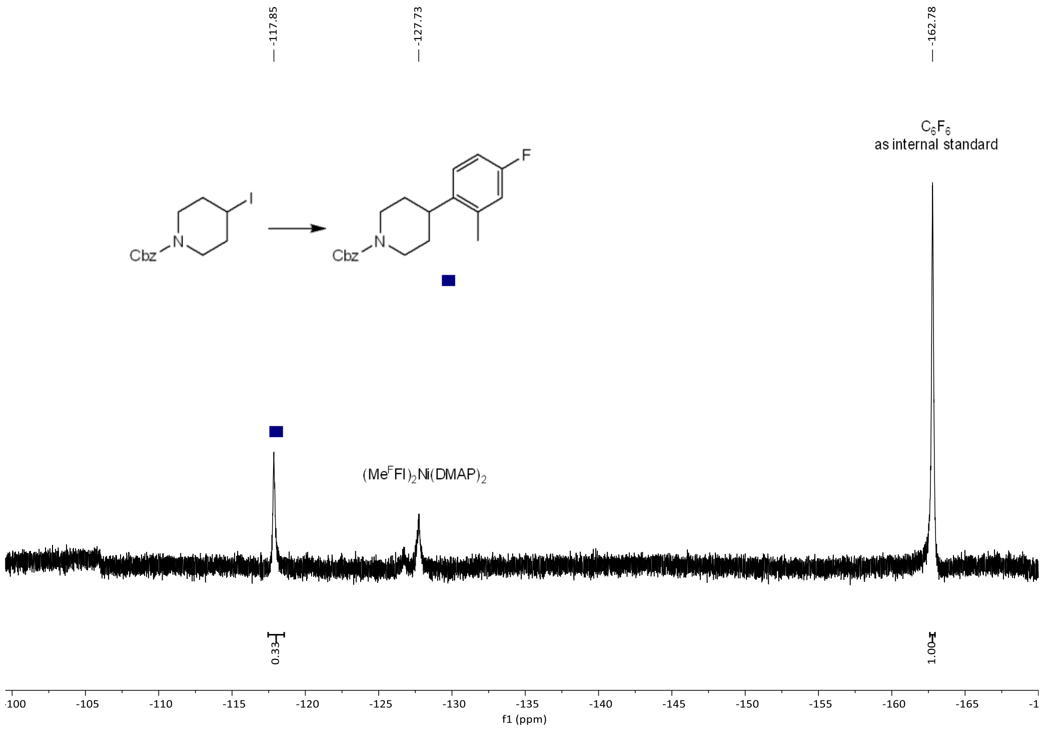

Figure S43. Crude reaction mixture with benzyl 4-iodopiperidine-1-carboxylate as electrophile monitored by ^{19}F NMR.

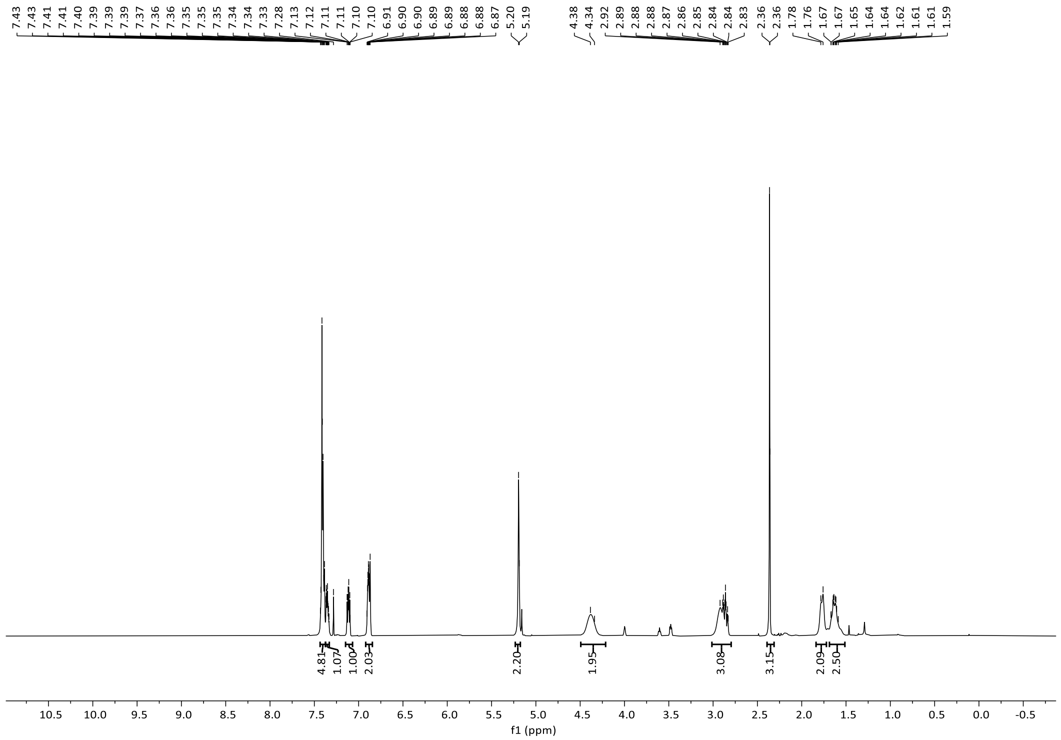

Figure S44. ^{1}H NMR spectrum (500 MHz, $CDCl_3$) of benzyl 4-(4-fluoro-2-methylphenyl)piperidine-1-carboxylate.

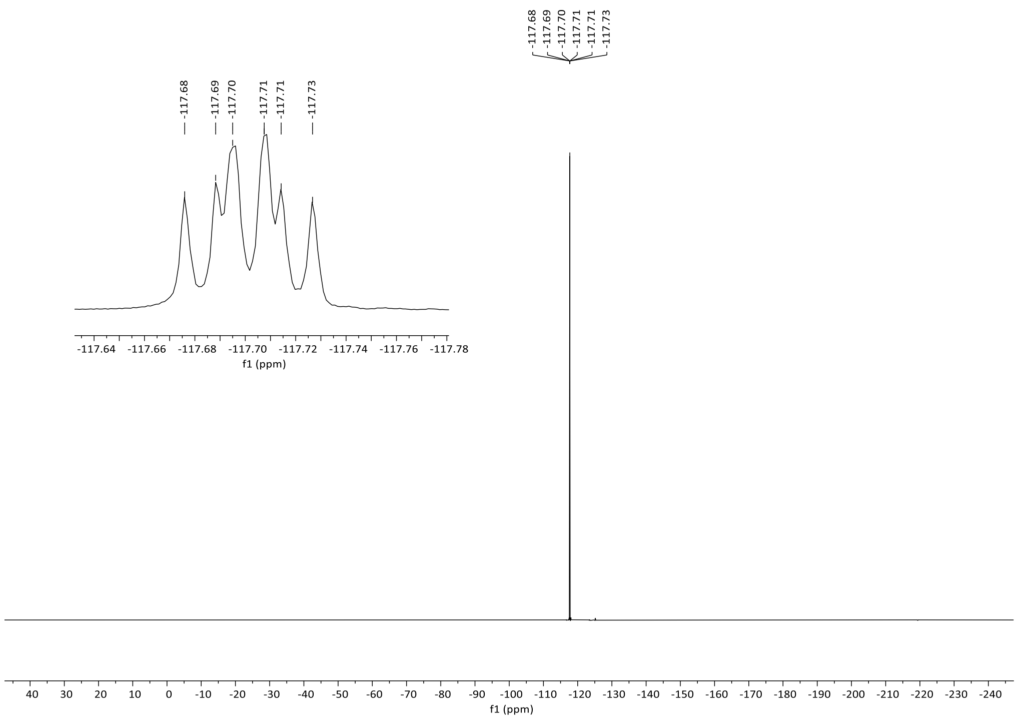

Figure S45. ^{19}F NMR spectrum (471 MHz, $CDCl_3$) of benzyl 4-(4-fluoro-2-methylphenyl)piperidine-1-carboxylate.

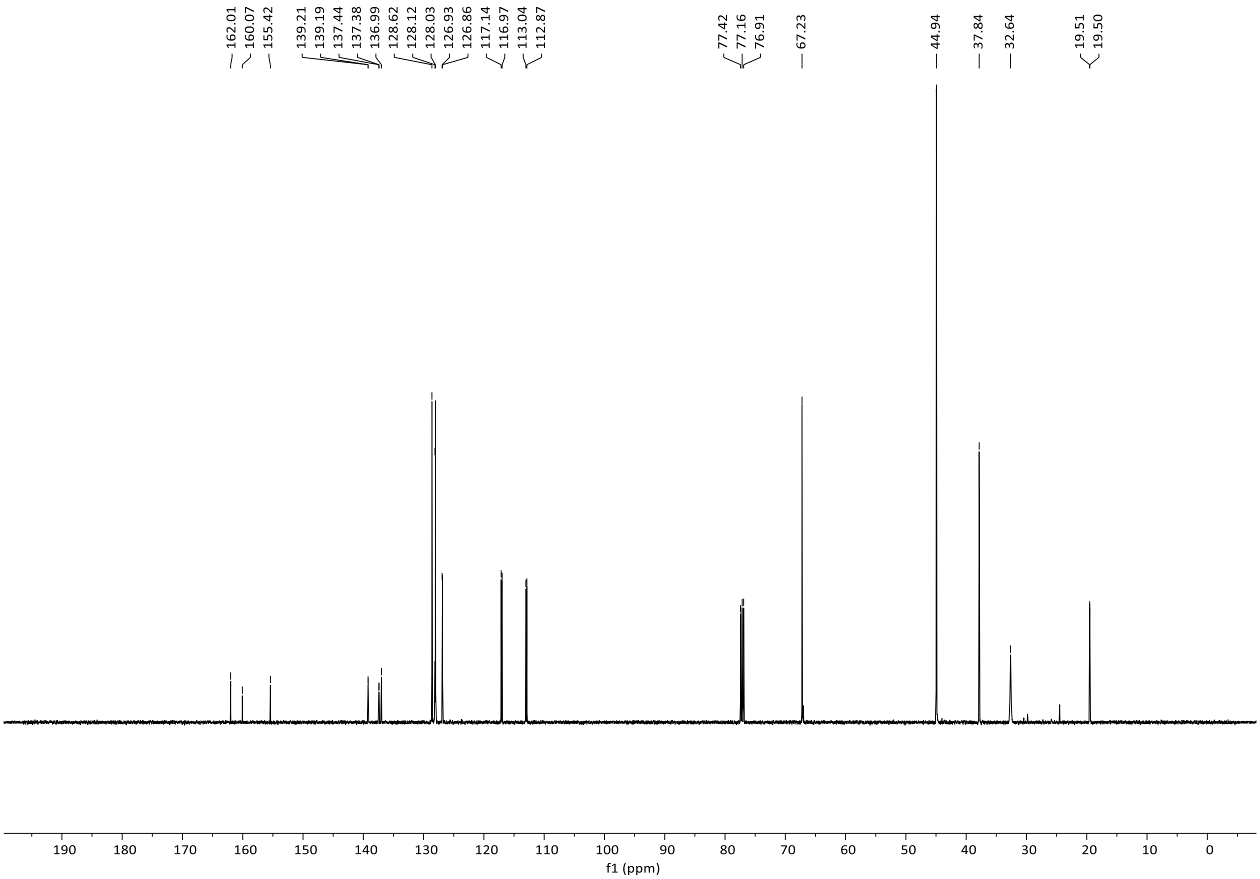

Figure S46. ^{13}C NMR spectrum (126 MHz, $CDCl_3$) of benzyl 4-(4-fluoro-2-methylphenyl)piperidine-1-carboxylate.

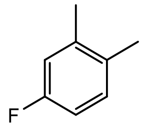

4-fluoro-1,2-dimethylbenzene

With Me-I as electrophile: The title compound was form in 88% yield with >98% conversion of $(Me^{F}FI)Ni(2\text{-}Me\text{-}4\text{-}F\text{-}C_6H_3)(DMAP)$ by ^{19}F NMR analysis, product was not isolated due to its low boiling point. The chemical shift was consistent with reported values.[6]

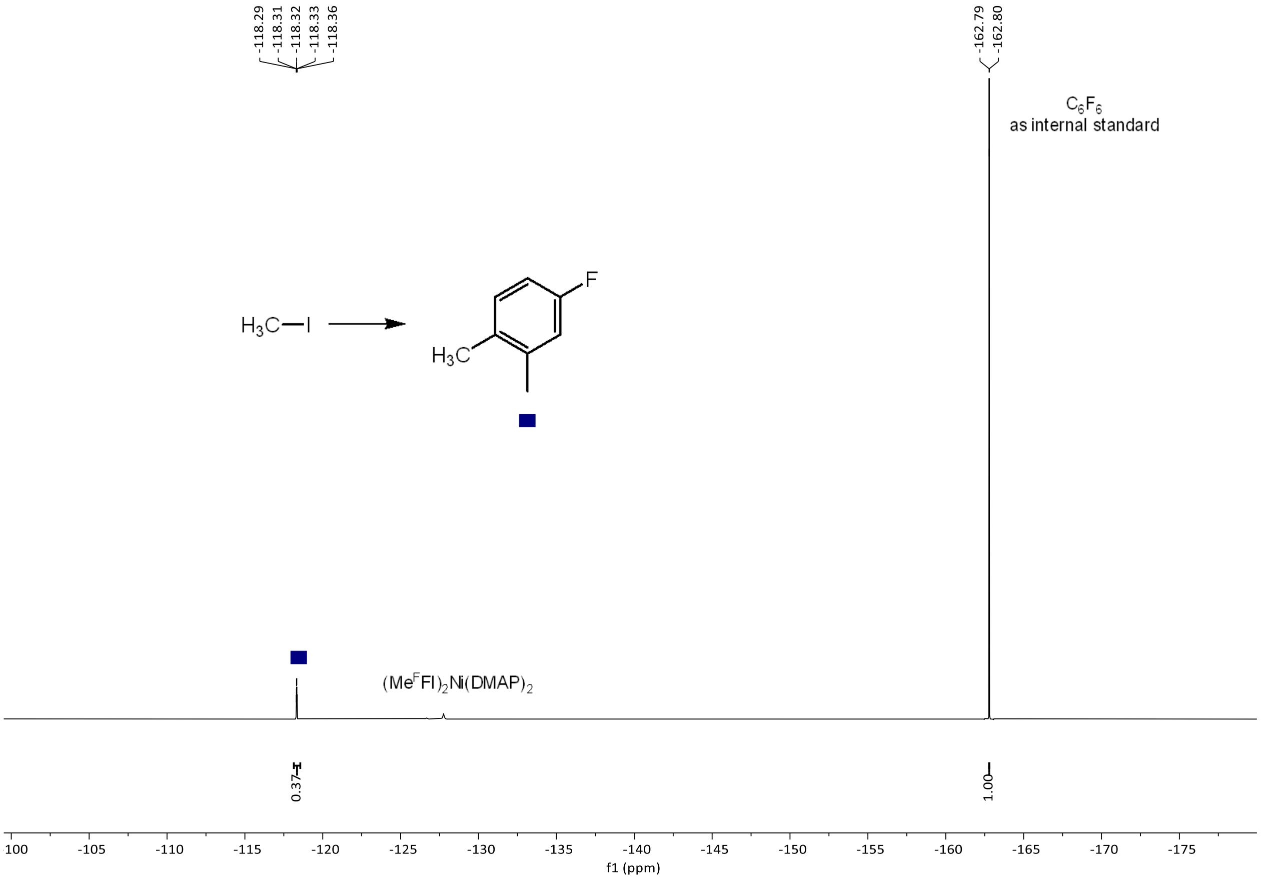

Figure S47. Crude reaction mixture with methyl iodide as electrophile monitored by ^{19}F NMR.

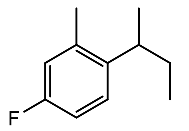

1-(*sec*-butyl)-4-fluoro-2-methylbenzene

With 2-bromobutane as electrophile: The title compound was form in 67% yield with 85% conversion of (Me^{F}FI)Ni(2-Me-4-F-C_6H_3)(DMAP) by ^{19}F NMR analysis, product was isolated as a colorless oil (10 mg, 0.060 mmol, 60%). **^{1}H NMR** (500 MHz, $CDCl_3$, 25 ºC): δ 7.11 (dd, *J* = 8.5, 6.0 Hz, 1H), 6.88 – 6.80 (m, 2H), 2.83 (h, *J* = 7.0 Hz, 1H), 2.30 (s, 3H), 1.62 – 1.55 (m, 2H), 1.17 (d, *J* = 6.9 Hz, 3H), 0.84 (t, *J* = 7.4 Hz, 3H). **^{19}F NMR** (471 MHz, $CDCl_3$, 25 ºC): δ -119.01 (ddd, *J* = 10.0, 8.5, 5.9 Hz). **^{13}C NMR** (126 MHz, $CDCl_3$, 25 ºC): δ 160.7 (d, *J* = 242.5 Hz), 141.5 (d, *J* = 3.2 Hz), 137.7 (d, *J* = 7.2 Hz), 126.7 (d, *J* = 8.2 Hz), 116.6 (d, *J* = 20.6 Hz), 112.8 (d, *J* = 20.6 Hz), 35.8, 30.8, 21.5, 19.8 (d, *J* = 1.6 Hz), 12.3. **HRMS** m/z (EI^{+}) calcd for $C_{11}H_{15}F^{+}$ (M^{+}) 166.1152, found: 166.1159

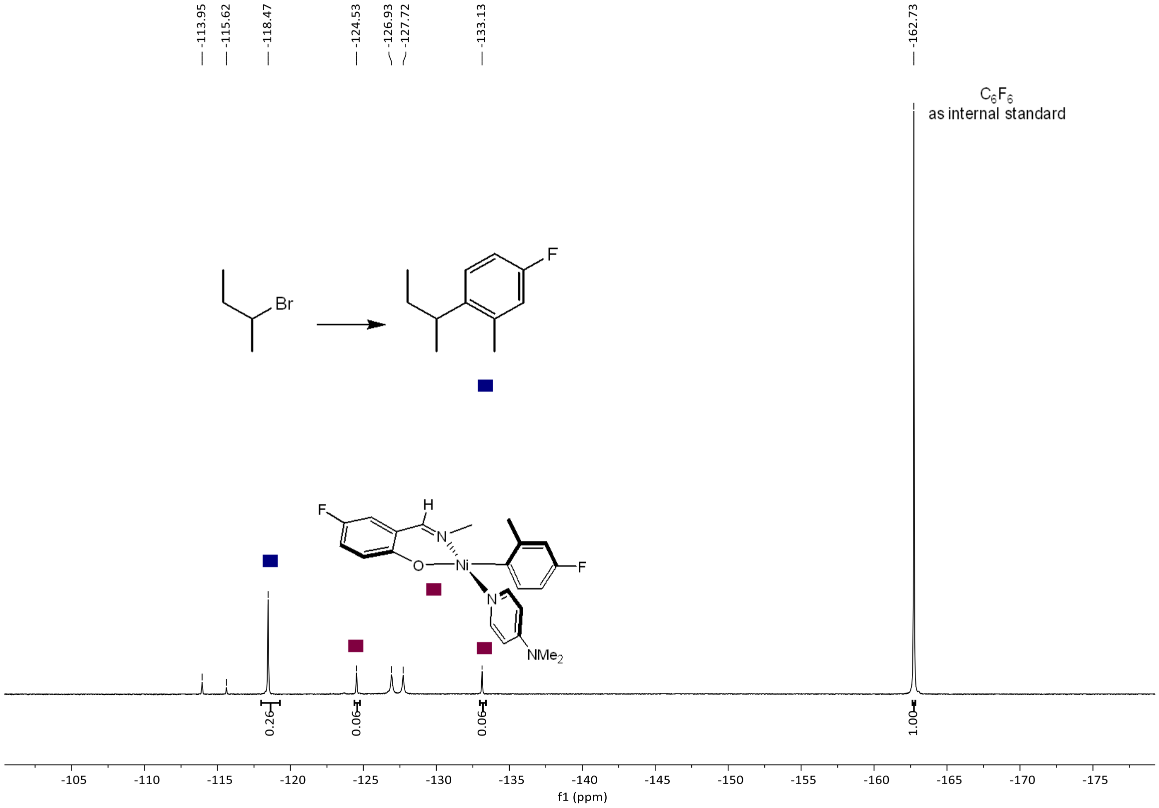

Figure S48. Crude reaction mixture with *sec*-butyl bromide as electrophile monitored by ^{19}F NMR.

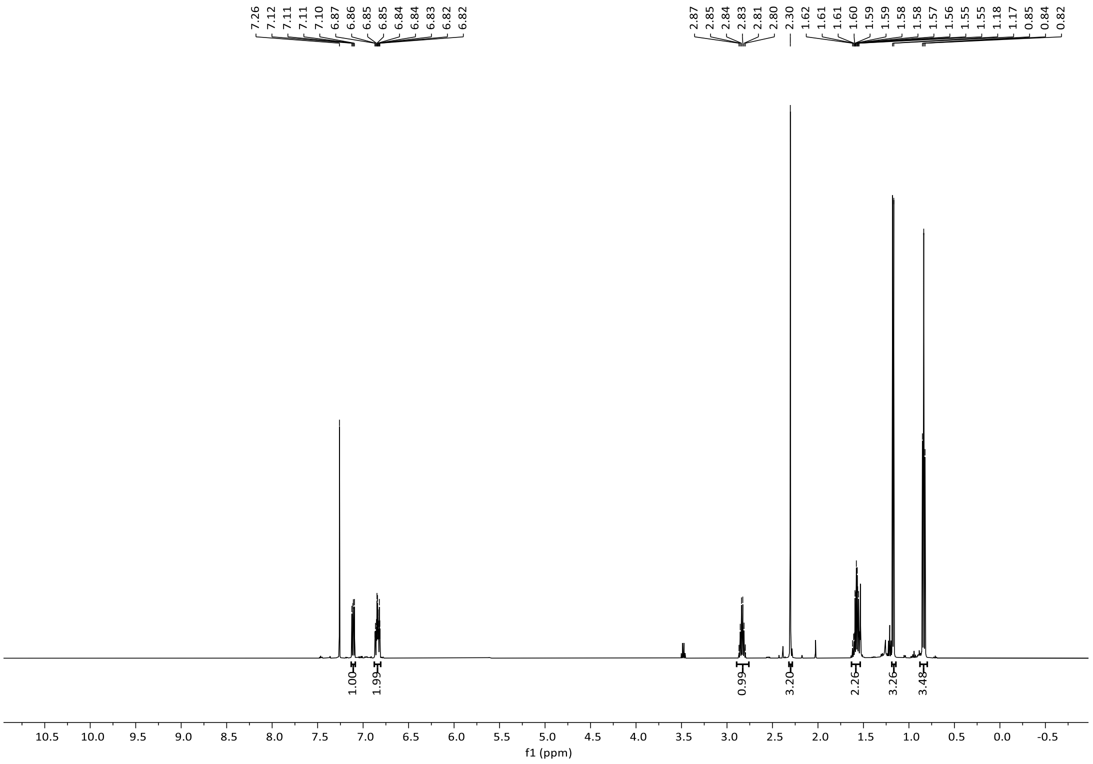

Figure S49. ^{1}H NMR spectrum (500 MHz, $CDCl_3$) of 1-(*sec*-butyl)-4-fluoro-2-methylbenzene

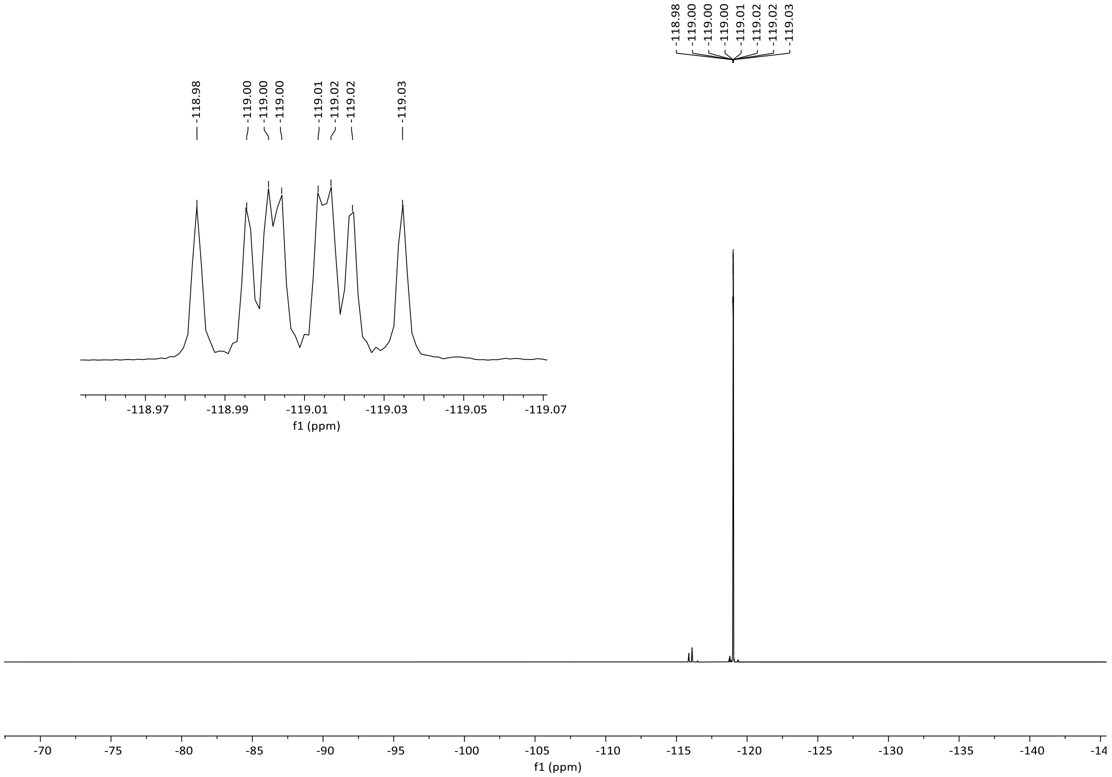

Figure S50. ^{19}F NMR spectrum (471 MHz, $CDCl_3$) of 1-(*sec*-butyl)-4-fluoro-2-methylbenzene

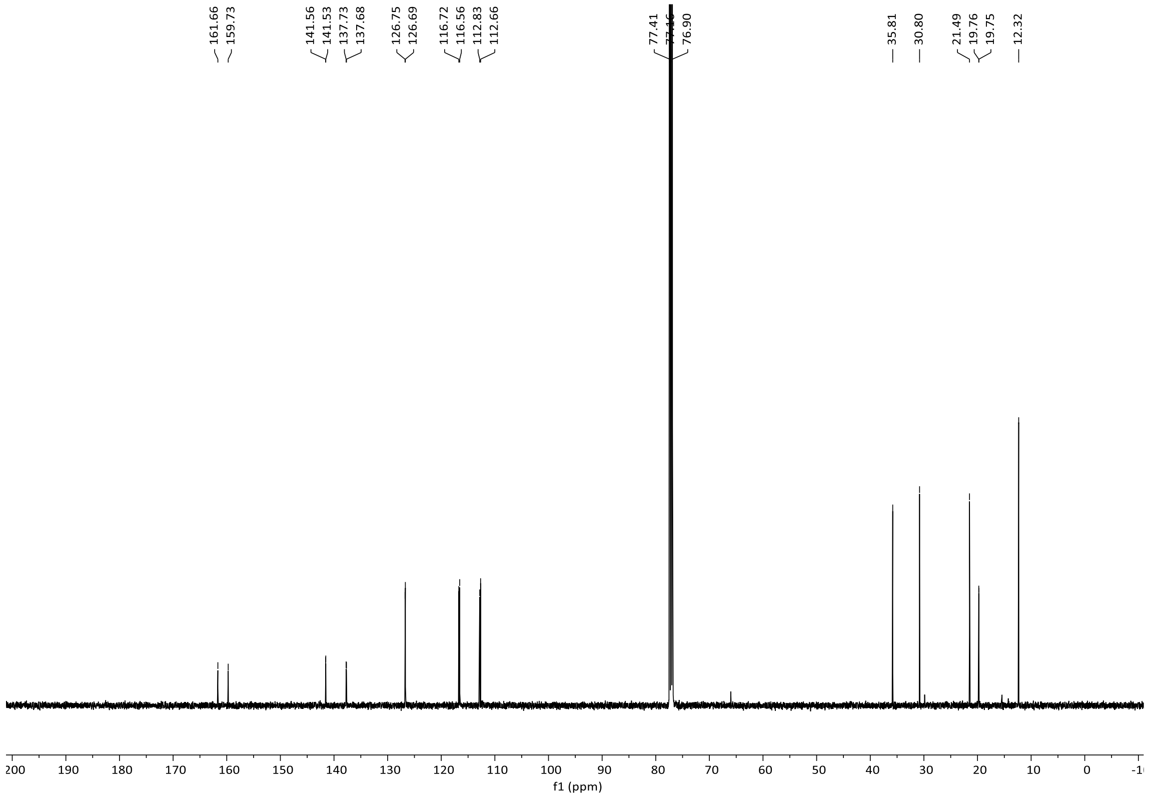

Figure S51. ^{13}C NMR spectrum (126 MHz, $CDCl_3$) of 1-(*sec*-butyl)-4-fluoro-2-methylbenzene

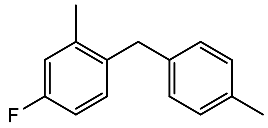

4-fluoro-2-methyl-1-(4-methylbenzyl)benzene

With 1-(bromomethyl)-4-methylbenzene as electrophile: The title compound was form in 55% yield with 64% conversion of (Me^{F}FI)Ni(2-Me-4-F-C_6H_3)(DMAP) by ^{19}F NMR analysis, product was isolated as a colorless oil (12 mg, 0.056 mmol, 56%).

^{1}H NMR (500 MHz, $CDCl_3$, 25 ºC): δ 7.09 (d, *J* = 7.8 Hz, 2H), 7.04 (dd, *J* = 8.4, 6.0 Hz, 1H), 6.99 (d, *J* = 7.8 Hz, 2H), 6.87 (dd, *J* = 9.7, 2.8 Hz, 1H), 6.83 (td, *J* = 8.4, 2.8 Hz, 1H), 3.90 (s, 1H), 2.32 (s, 2H), 2.23 (s, 2H). **^{19}F NMR** (471 MHz, $CDCl_3$, 25 ºC): δ -117.81 (td, *J* = 9.1, 6.0 Hz). **^{13}C NMR** (126 MHz, $CDCl_3$, 25 ºC): δ 161.6 (d, *J* = 243.6 Hz), 138.9 (d, *J* = 7.4 Hz), 137.3, 135.7, 135.0 (d, *J* = 3.1 Hz), 131.2 (d, *J* = 8.2 Hz), 129.3, 128.6, 117.0 (d, *J* = 20.8 Hz), 112.6 (d, *J* = 20.6 Hz), 38.4, 21.1, 19.9 (d, *J* = 1.7 Hz). **HRMS** m/z (EI) calcd for $C_{15}H_{15}F^+$ (M^+) 214.1152, found: 214.1157

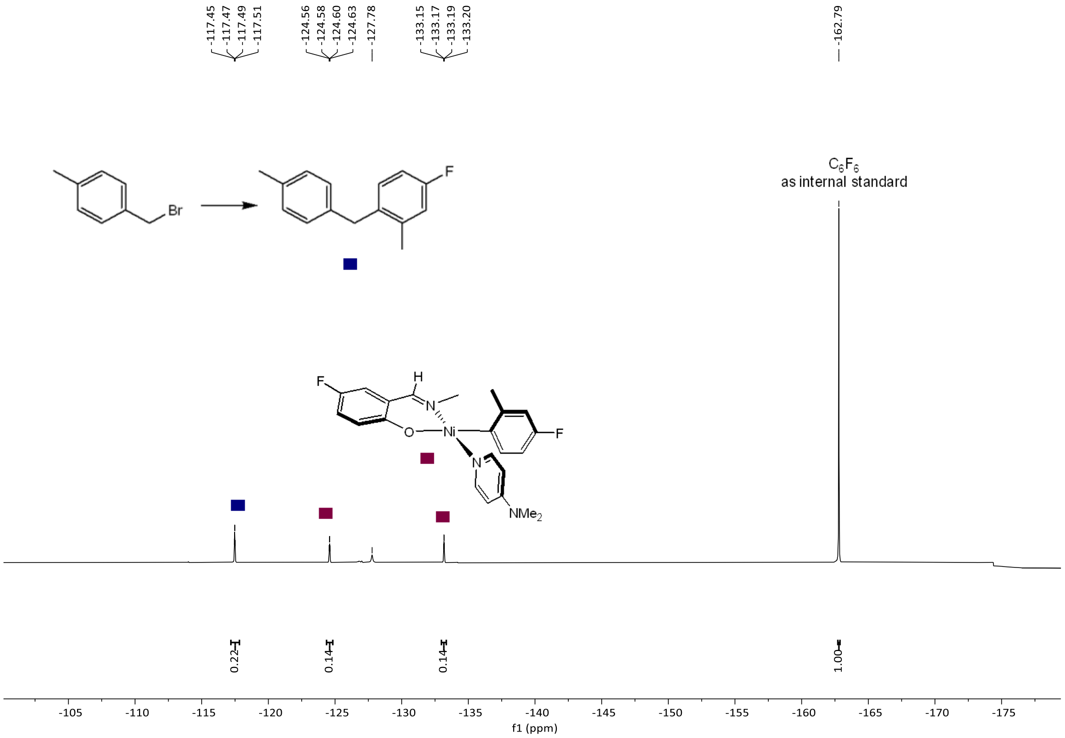

Figure S52. Crude reaction mixture with 1-(bromomethyl)-4-methylbenzene as electrophile monitored by ^{19}F NMR.

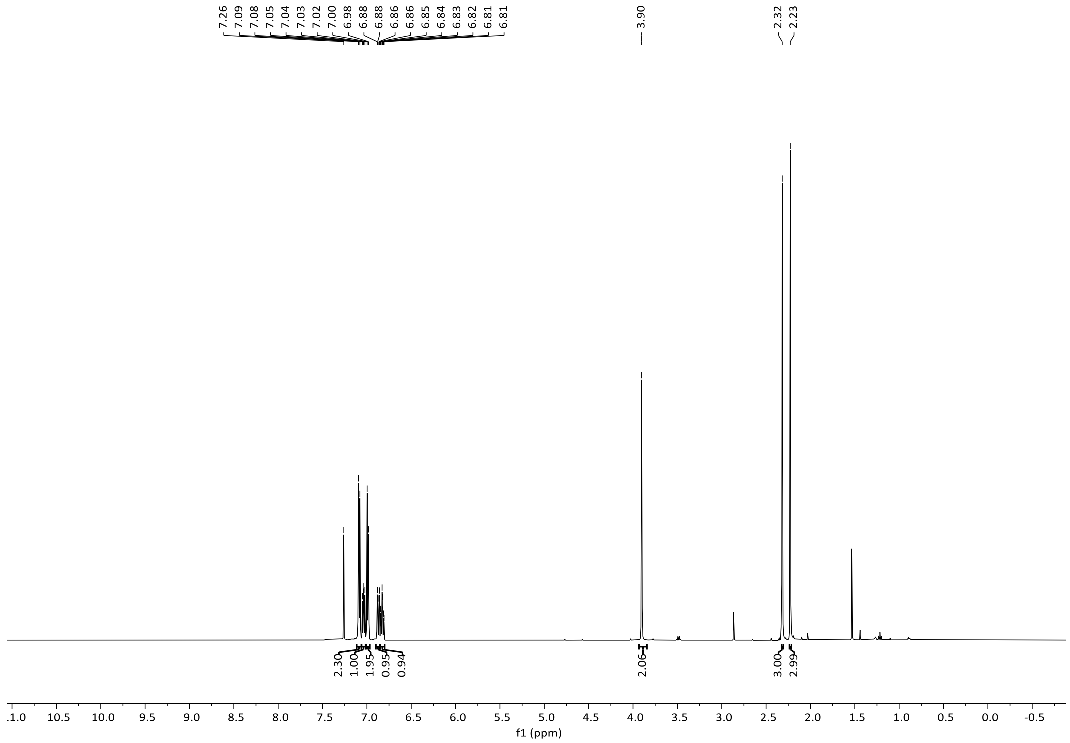

Figure S53. ^{1}H NMR spectrum (500 MHz, $CDCl_3$) of 4-fluoro-2-methyl-1-(4-methylbenzyl)benzene

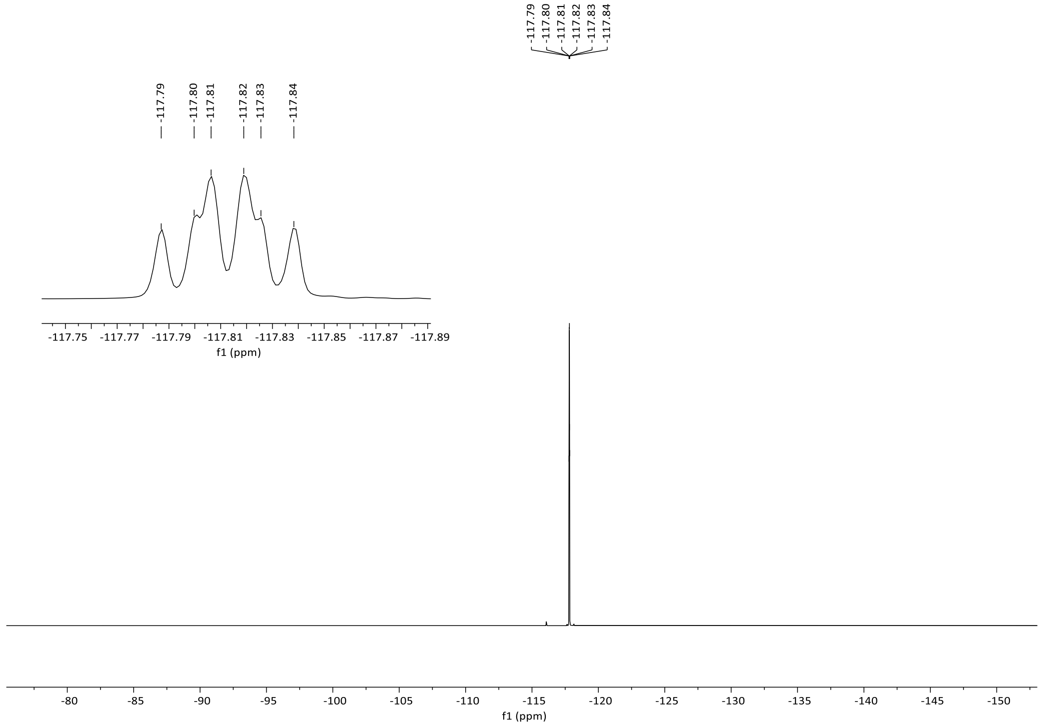

Figure S54. ^{19}F NMR spectrum (471 MHz, $CDCl_3$) of 4-fluoro-2-methyl-1-(4-methylbenzyl)benzene

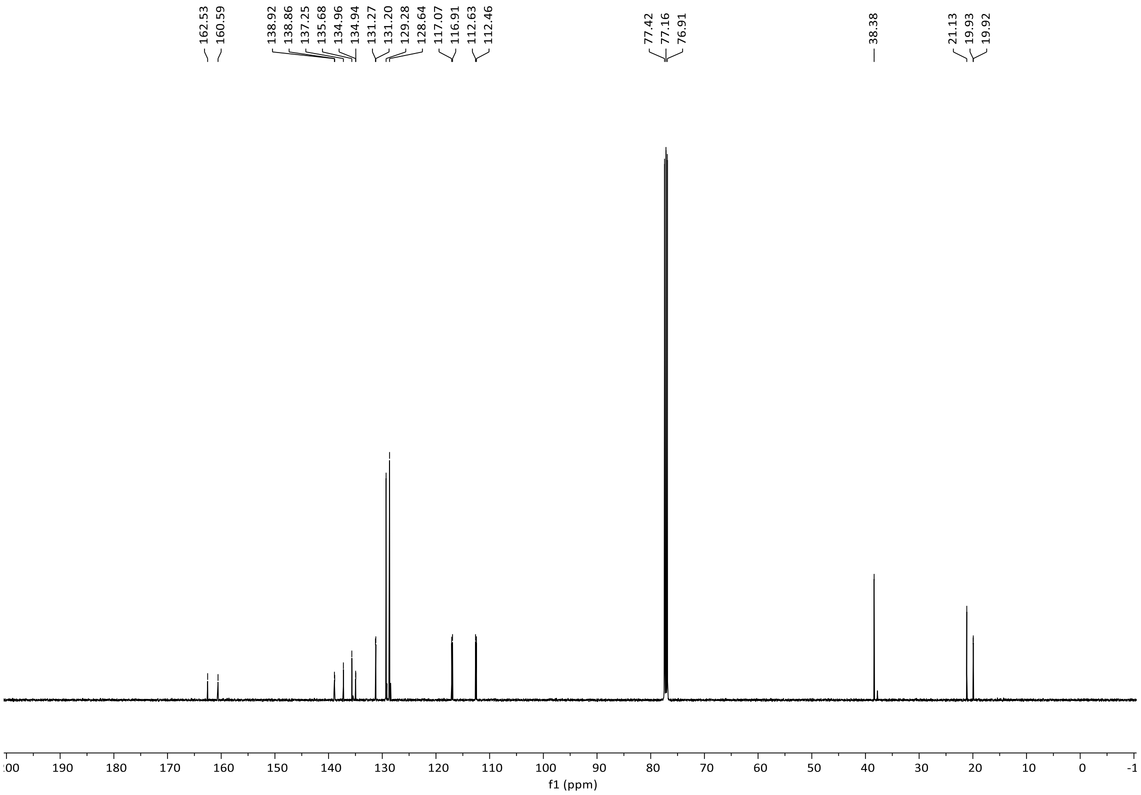

Figure S55. ^{13}C NMR spectrum (126 MHz, $CDCl_3$) of 4-fluoro-2-methyl-1-(4-methylbenzyl)benzene

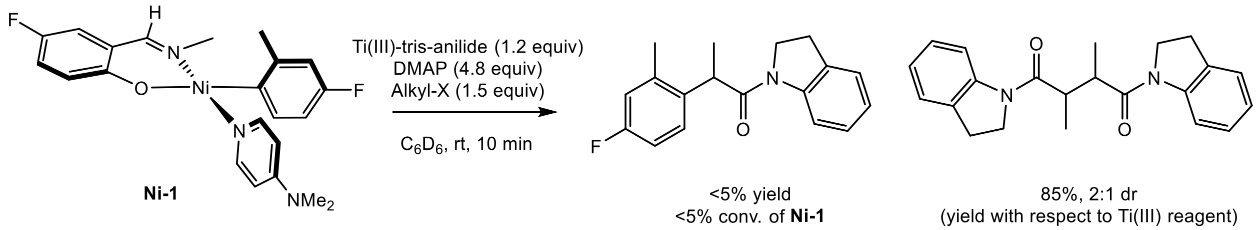

For alkyl electrophile 2-bromo-1-(indolin-1-yl)propan-1-one (**9**), no conversion of nickel(II)-aryl complex was observed. (Similar results were also observed when ^{t}Bu-Br was used.) Analysis of crude reaction mixture with ^{1}H NMR and HRMS after aqueous work up indicated radical dimerization as the major organic product.

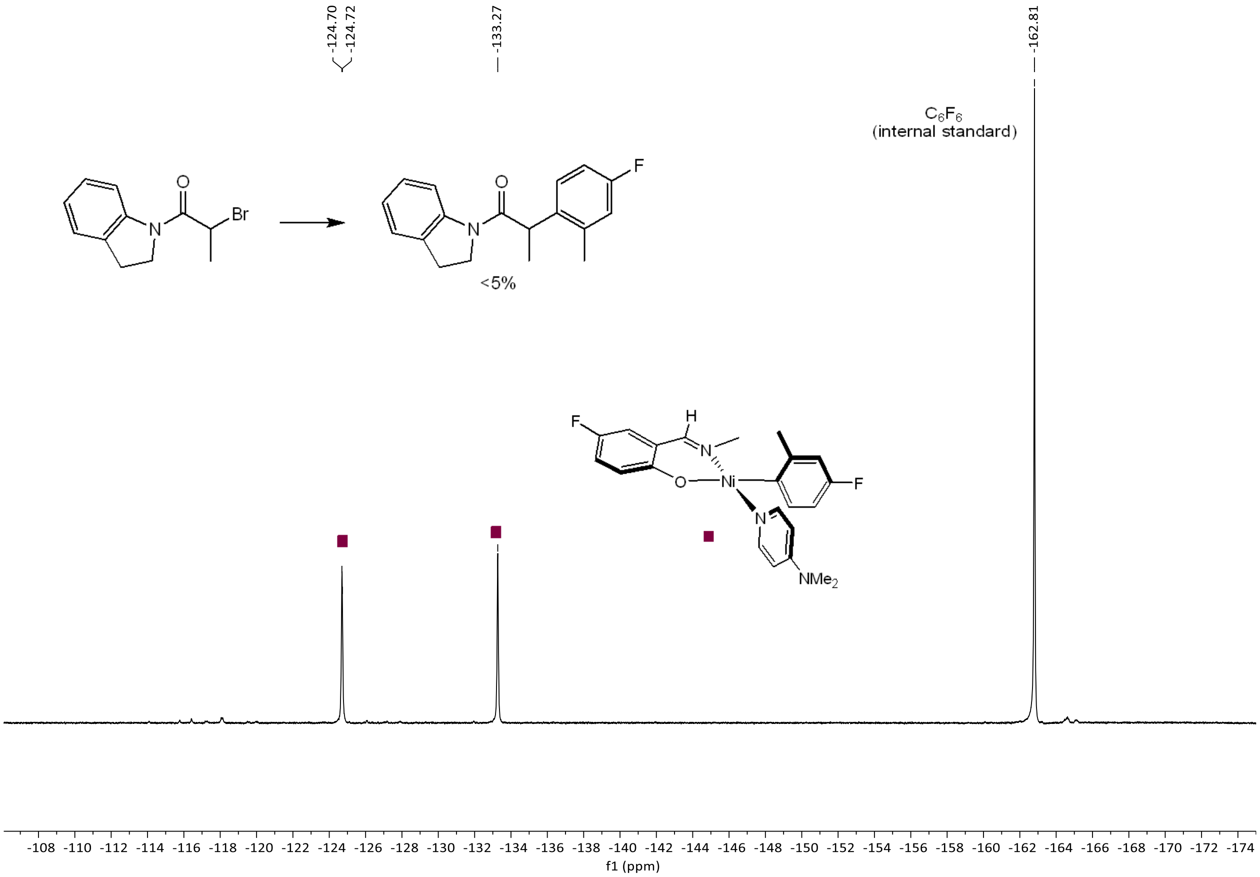

Figure S56. Crude reaction mixture with 2-bromo-1-(indolin-1-yl)propan-1-one as electrophile monitored by ^{19}F NMR.

V Parallel Radical Clock Experiments

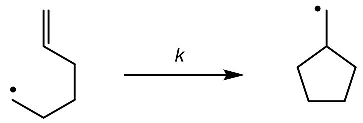

Kinetic data of 5-exo-trig cyclization of a hexenyl radical was reported.[7] Rate constants at given temperature were calculated based on the following equation:

$$\log k = (10.42 \pm 0.32) - \frac{(6.85 \pm 0.42)}{2.3RT}$$

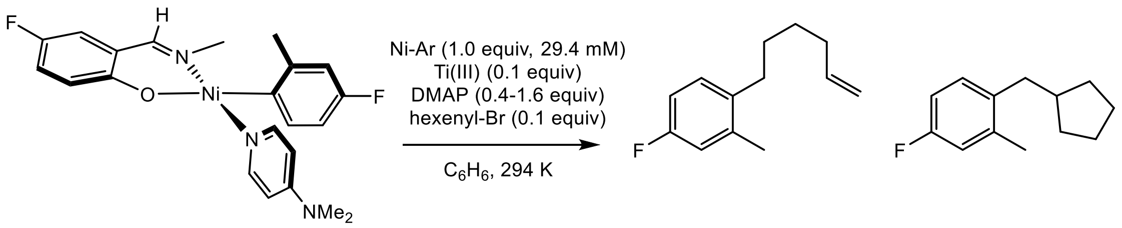

DMAP Effects: In a N_2-filled glovebox, a 20-mL vial with a stir bar was added a benzene solution of Ti(III)-tris-anilide (0.1 mL, 0.05 M, 0.1 equiv) and DMAP (0.2 equiv to 1.6 equiv). A benzene solution of $(Me^{F}FI)Ni(2\text{-}Me\text{-}4\text{-}F\text{-}C_6H_3)(DMAP)$ (1 mL, 0.05 M, 1.0 equiv) and 0.55 mL of benzene was then added to normalize concentration, followed by adding a solution of hexenyl bromide (0.05 mL, 0.1 M, 0.1 equiv). The reaction mixture was allowed to stir at room temperature for 1 h. Upon completion, the mixture was quenched with HCl (2M, aq.) and extracted with Et_2O. The organic layers were combined and concentrated under reduced pressure. Linear to cyclized product ratio was analyzed by ^{19}F NMR.

DMAP (0.4 equiv)

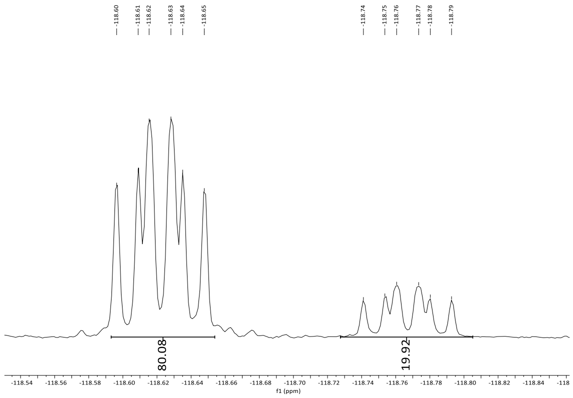

DMAP (0.8 equiv)

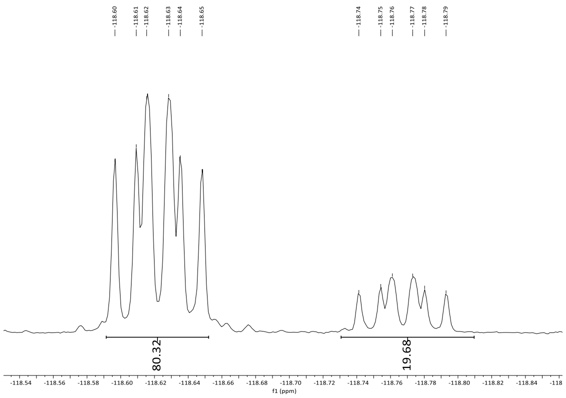

DMAP (1.6 equiv)

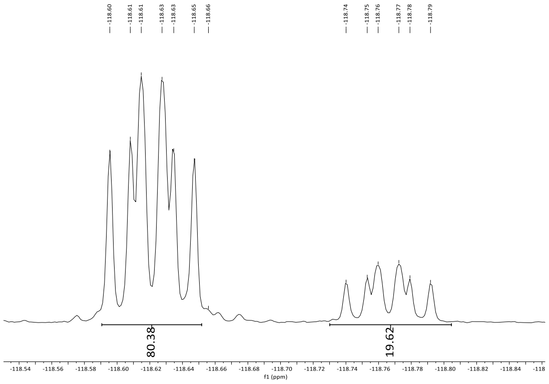

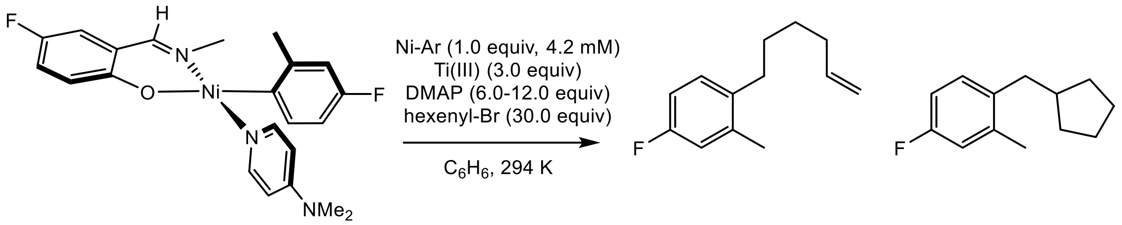

DMAP Effects (at higher DMAP concentration): In a N_2-filled glovebox, a 20-mL vial with a stir bar was added a benzene solution of Ti(III)-tris-anilide (0.1 mL, 0.1 M, 3.0 equiv) and a benzene solution of DMAP (0.1-0.5 mL, 0.2 M mmol, 6.0-30.0 equiv), 0.4-0.1 mL benzene was then added to normalize concentration. A benzene solution of ($Me^{F}FI$)Ni(2-Me-4-F-C_6H_3)(DMAP) (0.1 mL, 0.033 M, 1.0 equiv) was added, followed by adding a solution of hexenyl bromide (0.1 m, 1.0 M, 30.0 euqiv). The reaction mixture was allowed to stir at room temperature for 10 min, and all proceed to full conversion as monitored by ^{19}F NMR. Upon completion, the reaction mixture was quenched with HCl (2M, aq.) and extracted with Et_2O. The organic layers were combined and concentrated under reduced pressure. Linear to cyclized product ratio was analyzed by ^{19}F NMR.

[DMAP] = 25 mM

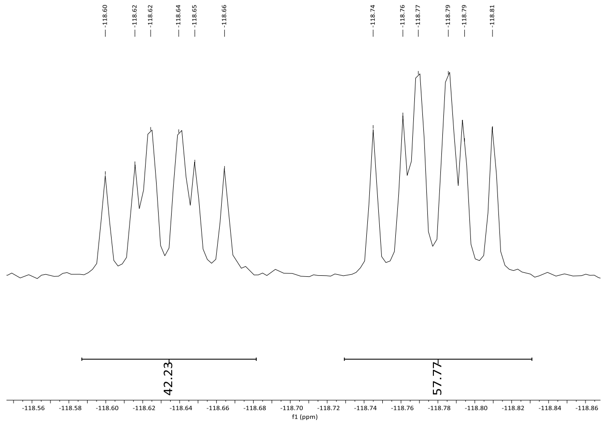

[DMAP] = 50 mM

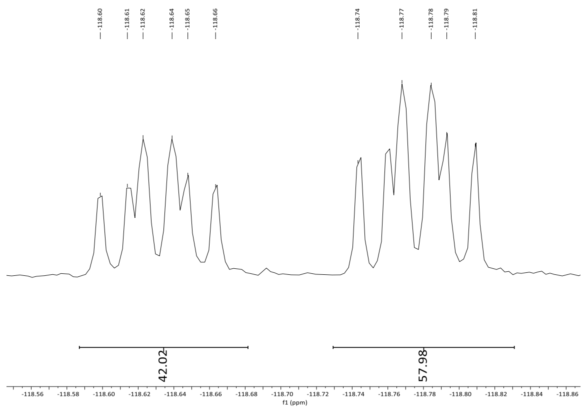

[DMAP] = 75 mM

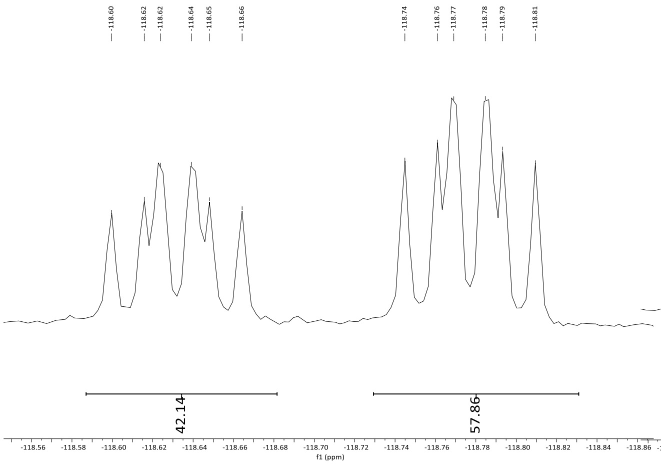

[DMAP] = 100 mM

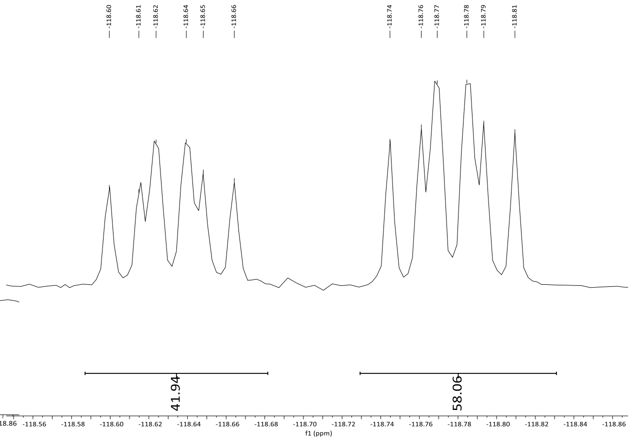

[DMAP] = 125 mM

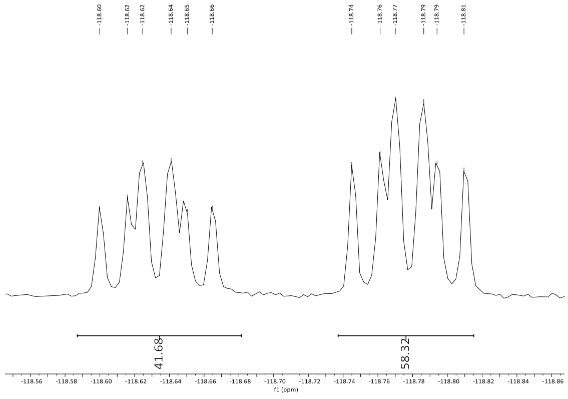

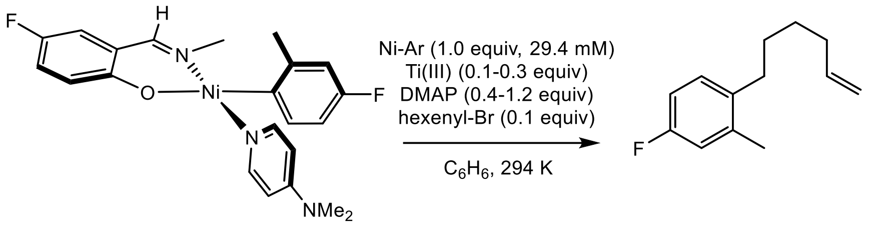

Ti(III)-tris-anilide Effects: In a N_2-filled glovebox, a 20-mL vial with a stir bar was added a benzene solution of DMAP and Ti(III)-tris-anilide (0.15-0.45 mL, 0.033 M (Ti), 0.132 M (DMAP)) and a benzene solution of (Me^{F}FI)Ni(2-Me-4-F-C_6H_3)(DMAP) (1 mL, 0.05 M, 1.0 equiv). 0.5-0.2 mL benzene was then added to normalize concentration, followed by adding a solution of hexenyl bromide (0.05 mL, 0.1 M, 0.1 equiv). The reaction mixture was allowed to stir at room temperature for 1 h. Upon completion, the mixture was quenched with HCl (2M, aq.) and extracted with Et_2O. The organic layers were combined and concentrated under reduced pressure. Linear to cyclized product ratio was analyzed by ^{19}F NMR.

Ti(III) and DMAP (0.1 and 0.4 equiv)

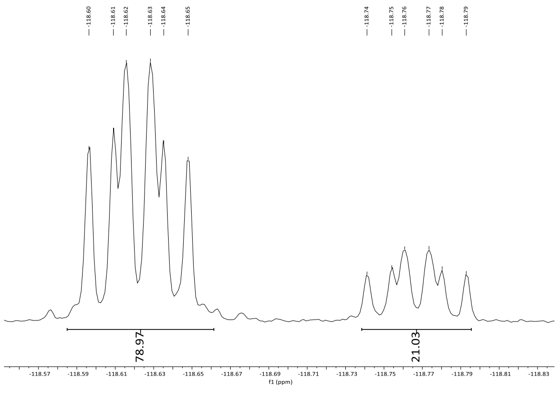

Ti(III) and DMAP (0.2 and 0.8 equiv)

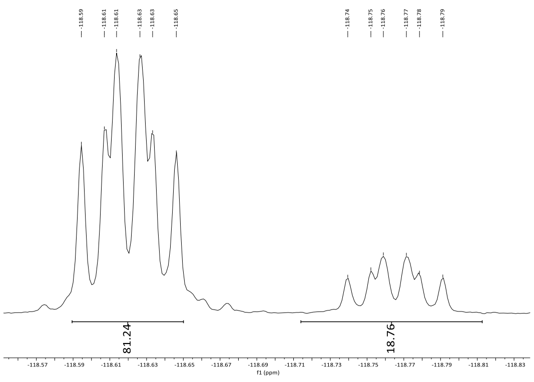

Ti(III) and DMAP (0.3 and 1.2 equiv)

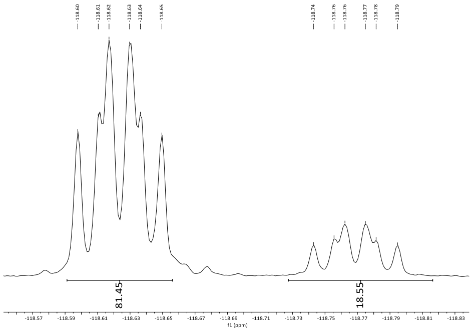

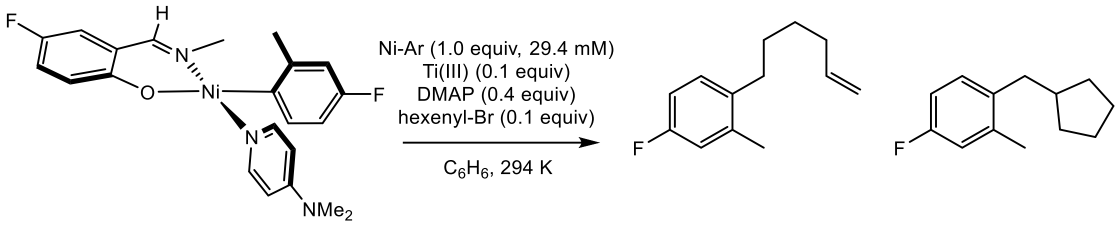

Concentration Effects: In a N_2-filled glovebox, a 20-mL vial with a stir bar was added a benzene solution of DMAP and Ti(III)-tris-anilide (0.15 mL, 0.033 M (Ti), 0.132 M (DMAP), 0.1 equiv) and a benzene solution of $(Me^{F}FI)Ni(2\text{-}Me\text{-}4\text{-}F\text{-}C_6H_3)(DMAP)$ (1 mL, 0.05 M, 1.0 equiv). 0-10.0 mL benzene was then added to adjust concentration, followed by adding a solution of hexenyl bromide (0.05 mL, 0.1 M, 0.1 equiv). The reaction mixture was allowed to stir at room temperature for 1 h. Upon completion, the mixture was quenched with HCl (2M, aq.) and extracted with Et_2O. The organic layers were combined and concentrated under reduced pressure. Linear to cyclized product ratio was analyzed by ^{19}F NMR. The reaction was replicated with two batches of nickel complex solution, which was separately plotted to minimize error from each stock solution preparation.

Batch 1:

[Ni] = 41.7 mM

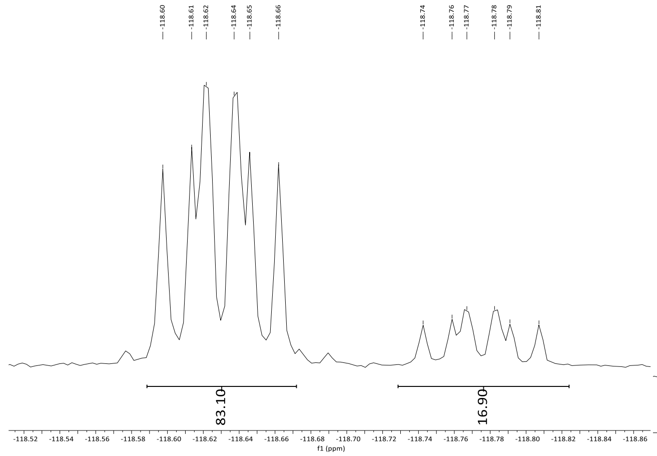

[Ni] = 29.4 mM

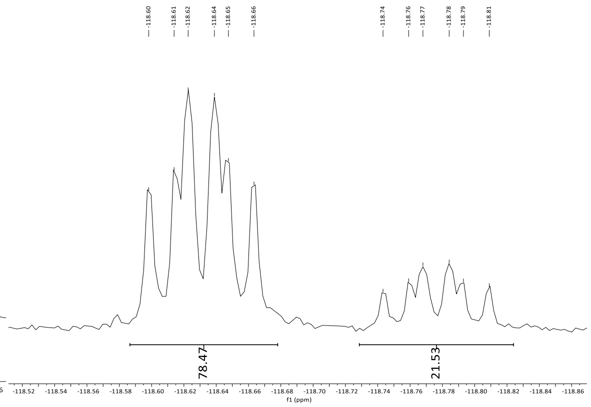

[Ni] = 15.6 mM

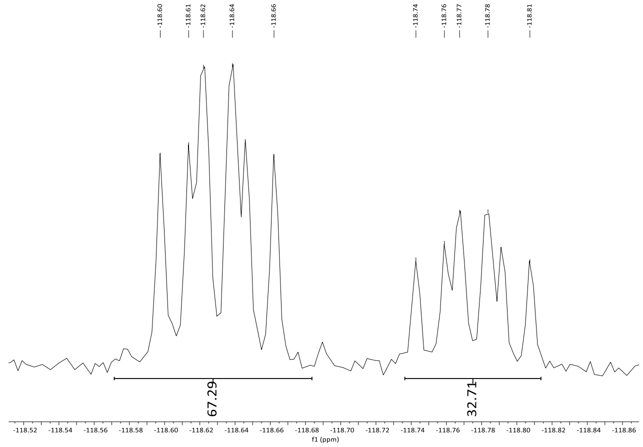

[Ni] = 8.1 mM

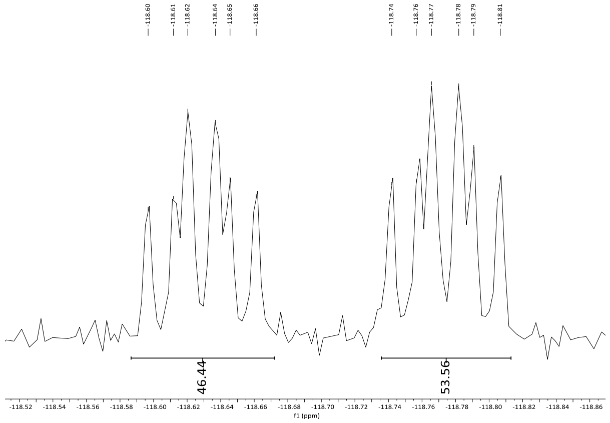

[Ni] = 4.5 mM

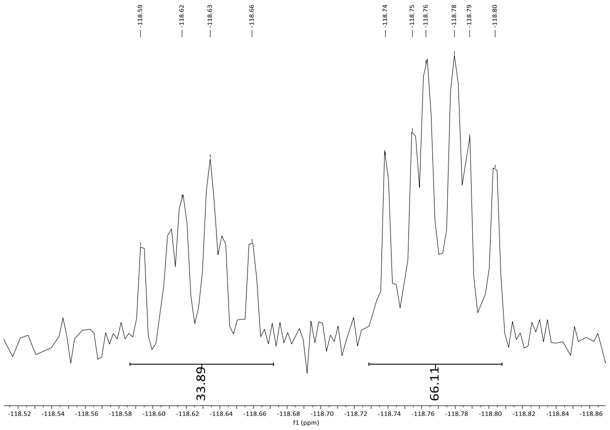

Batch 2:

[Ni] = 41.7 mM

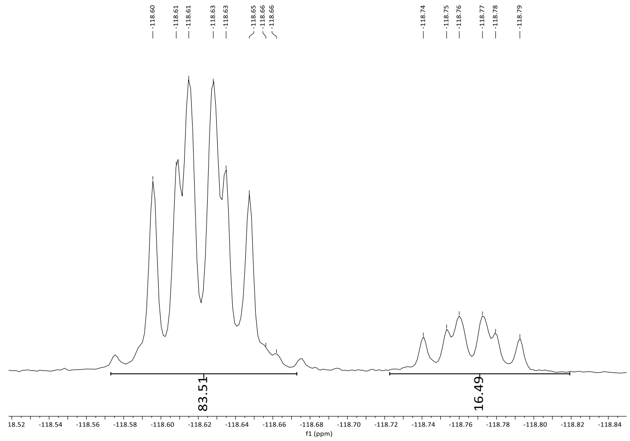

[Ni] = 29.4 mM

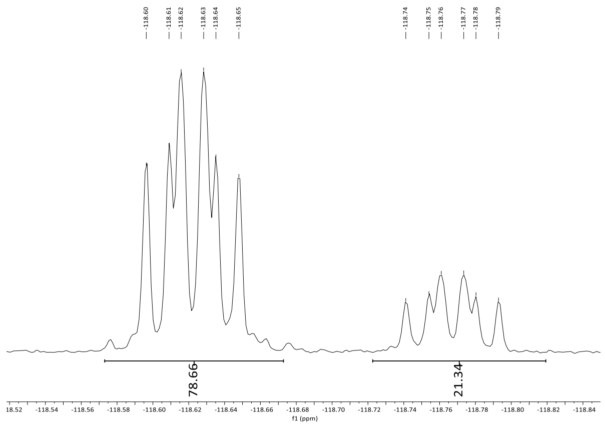

[Ni] = 15.6 mM

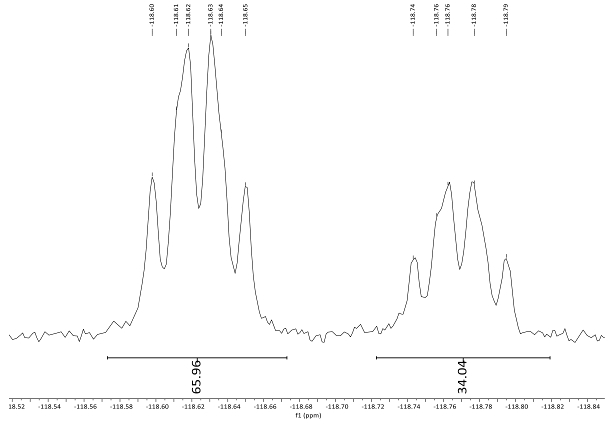

[Ni] = 8.1 mM

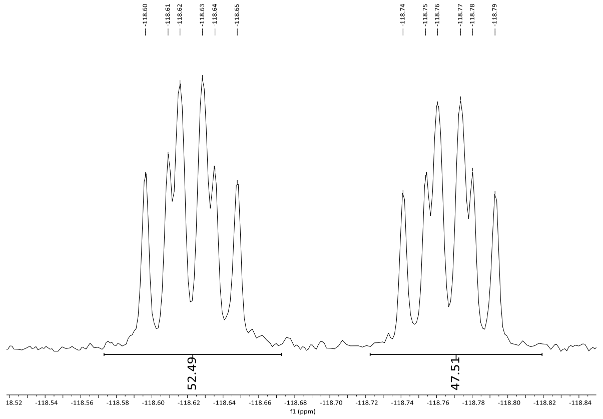

[Ni] = 4.5 mM

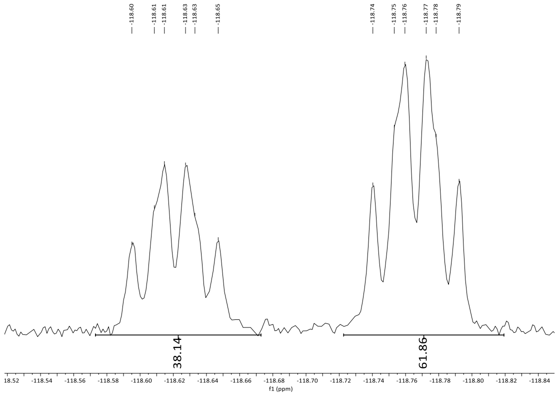

Table S1. Linear to cyclic product distribution as a function of concentration of nickel complex

concentration	ratio(l:c) (batch 1)	ratio(l:c) (batch 2)
0.004464	0.51	0.62
0.008065	0.87	1.10
0.015625	2.01	1.94
0.029412	3.65	3.69
0.041667	4.92	5.06
	**batch 1**	**batch 2**
slope	120.4	119.8
rate constant (mol·L^{-1}·S^{-1})	25809836.13	25666821.45
ΔG$^{\neq}$	7.235	7.238
ΔG$^{\neq}$(average)	7.237(0.002)	

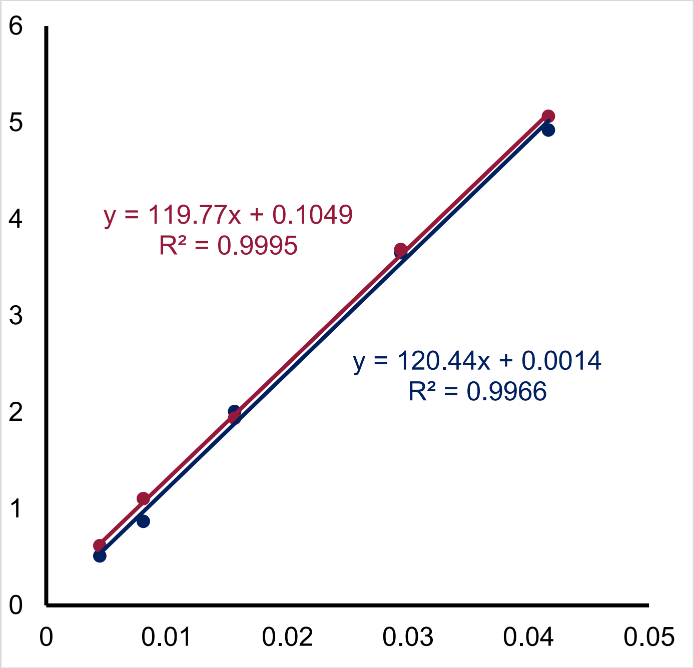

Figure S57. Linear to cyclic product distribution as a function of concentration of nickel complex

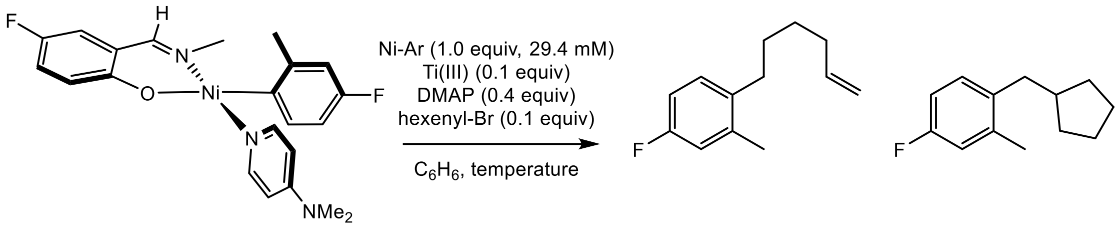

Temperature Effects: In a N_2-filled glovebox, a 20-mL vial with a stir bar was added a benzene solution of DMAP and Ti(III)-tris-anilide (0.15 mL, 0.033 M (Ti), 0.132 M (DMAP), 0.1 equiv) and a benzene solution of (Me^{F}FI)Ni(2-Me-4-F-C_6H_3)(DMAP) (1 mL, 0.05 M, 1.0 equiv). 0.5 mL benzene was then added to adjust concentration. The vial was then equilibrate to different temperatures (ice-acetone, ice-water, room temperature, hot plate), with temperature measured by a thermometer. A solution of hexenyl bromide was then added (0.05 mL, 0.1 M, 0.1 equiv). The reaction mixture was allowed to stir at room temperature for 1 h. Upon completion, the mixture was quenched with HCl (2M, aq.) and extracted with Et_2O. The organic layers were combined and concentrated under reduced pressure. Linear to cyclized product ratio was analyzed by ^{19}F NMR. The reaction was replicated with two batches of nickel complex solution, which was separately plotted to minimize error from each stock solution preparation.

Batch 1:

305 K (oil bath)

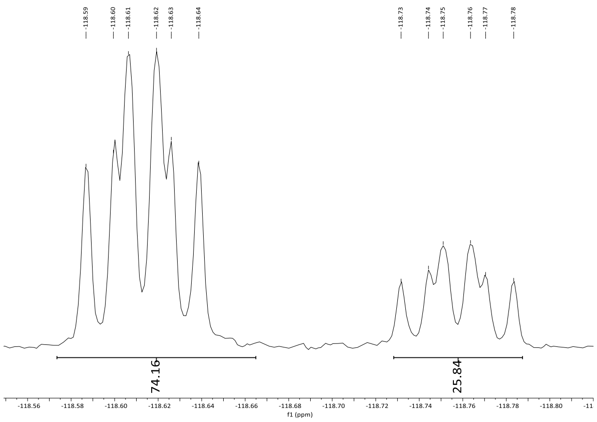

294 K (room temperature)

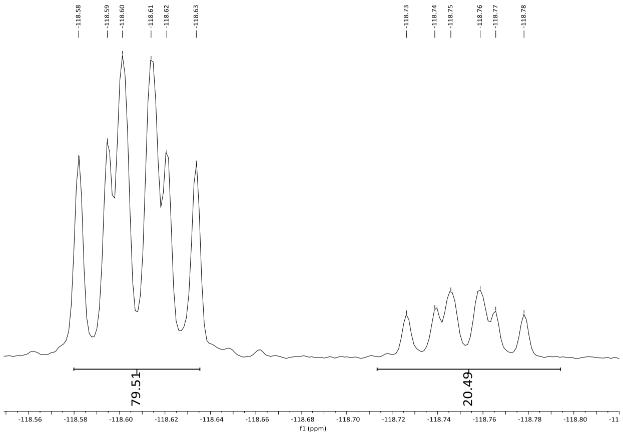

274 K (ice-water)

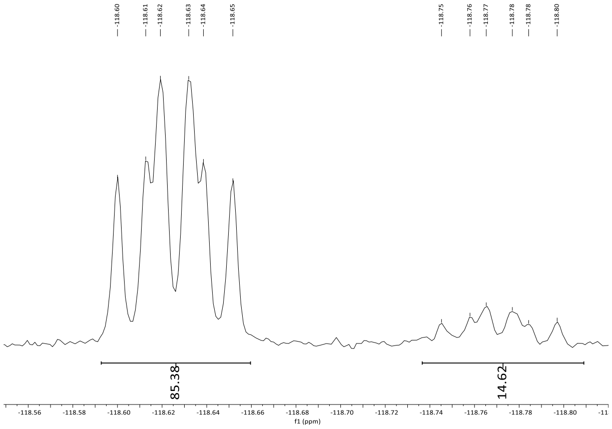

264 K (ice-acetone)

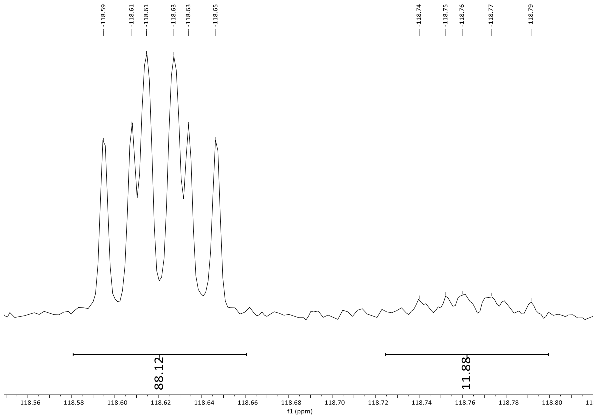

Batch 2:

306 K (hot plate)

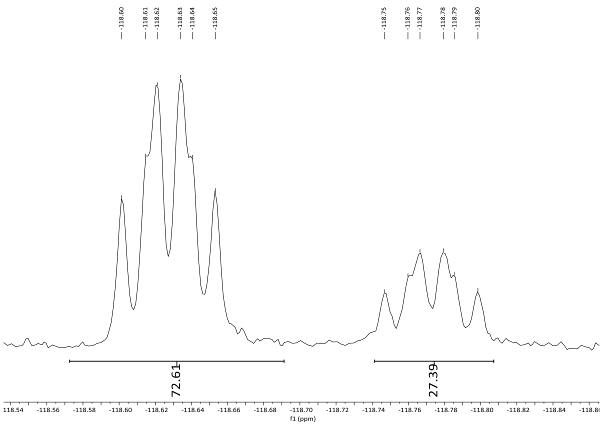

294 K (room temperature)

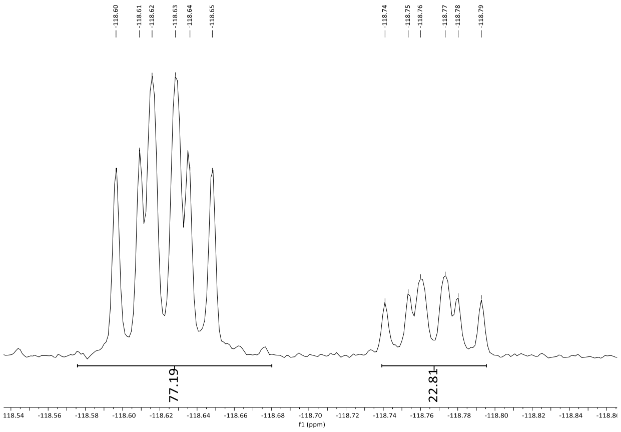

275 K (ice-water)

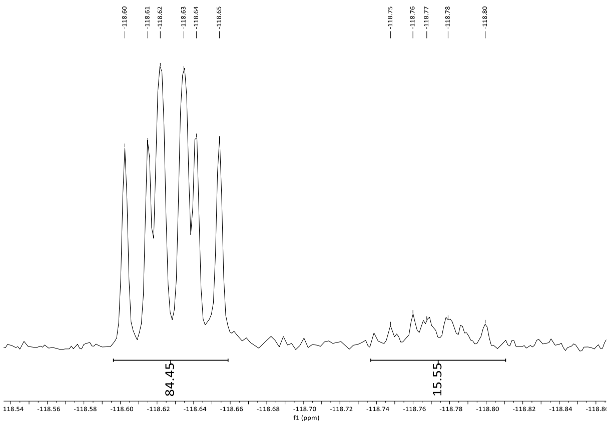

260 K (ice-acetone)

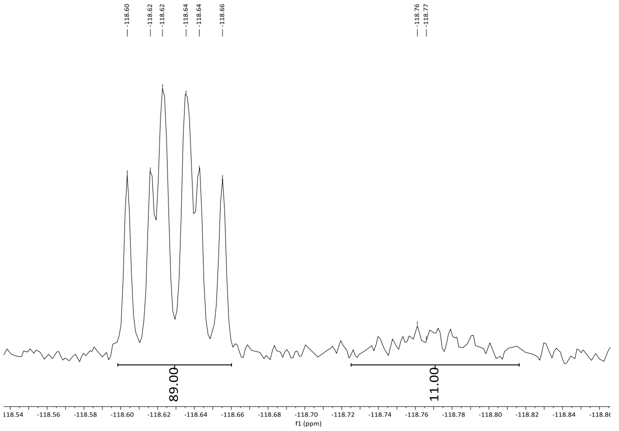

Table S2. Linear to cyclic product distribution as a function of reaction temperature

temperature (K)	ratio(l:c) (batch 1)	temperature (K)	ratio(l:c) (batch 2)
306	2.65	305	2.87
294	3.38	294	3.9
275	5.43	274	5.84
260	8.09	264	7.42
**1/T**	**ln(k/T) (batch 1)**	**1/T**	**ln(k/T) (batch 2)**
0.003266373	11.51129049	0.003277077	11.55741966
0.003399626	11.33529598	0.003399626	11.47839682
0.003634381	11.06698164	0.003647638	11.09772056
0.003843936	10.79943798	0.003785728	10.89836147
**ΔH**	2.425248	**ΔH**	2.671846
**ΔS**	-16.4258	**ΔS**	-15.4183
**ΔG(298K)**	7.3226	**ΔG(298K)**	7.268825

**ΔG$^{\neq}$(298K)**	7.30(4)	**ΔH$^{\neq}$**	2.55(17)	**ΔS$^{\neq}$**	-15.9(7)

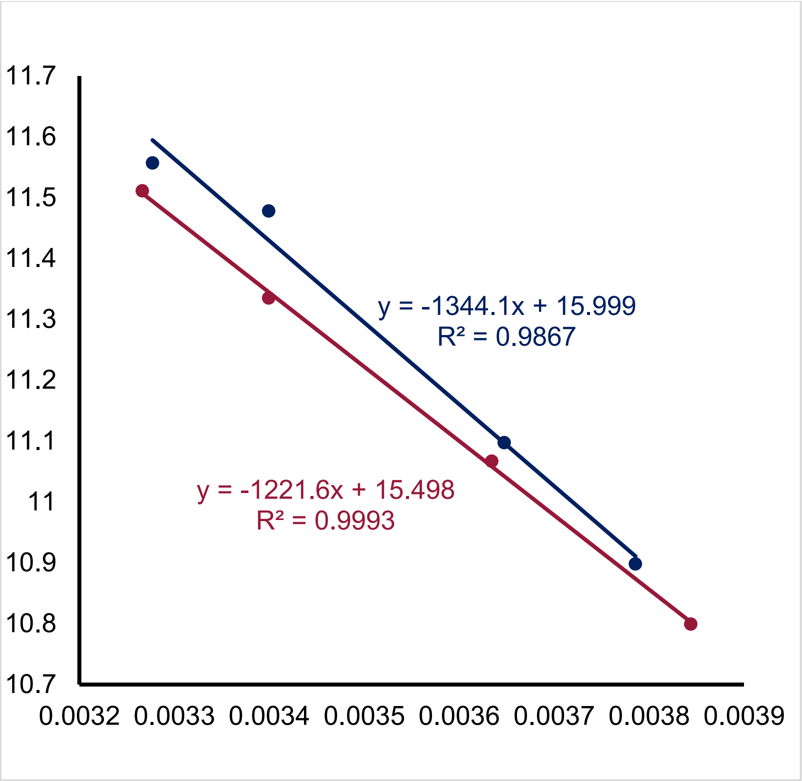

Figure S58. Eyring plot for radical coupling with phenoxy(imine)-nickel(II)-aryl complexes

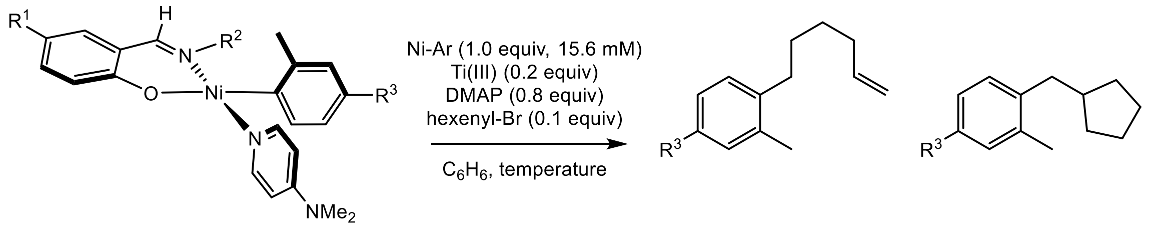

Radical Clock Experiment with Different Ni Complexes: In a N_2-filled glovebox, a 20-mL vial with a stir bar was added a benzene solution of DMAP and Ti(III)-tris-anilide (0.6 mL, 0.033 M (Ti), 0.132 M (DMAP), 0.2 equiv) and a benzene solution of (Me^{F}FI)Ni(2-Me-4-F-C_6H_3)(DMAP) (5 mL, 0.02 M, 0.1 mmol, 1.0 equiv). 0.7 mL benzene was then added to adjust concentration. A solution of hexenyl bromide was then added (0.1 mL, 0.1 M, 0.1 equiv). The reaction mixture was allowed to stir at room temperature for 1 h. Upon completion, the mixture was quenched with HCl (2M, aq.) and extracted with Et_2O. The organic layers were combined and concentrated under reduced pressure. Linear to cyclized product ratio was analyzed by ^{19}F NMR or ^{1}H NMR. The reaction was replicated two times for each complex.

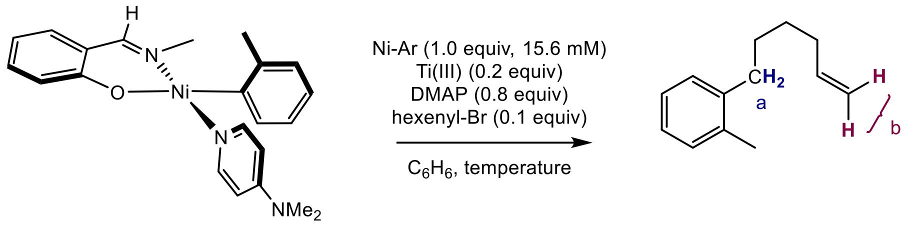

Linear to cyclized ratio was determined by ^{1}H NMR, ratio = $\frac{b}{a-b}$

run 1

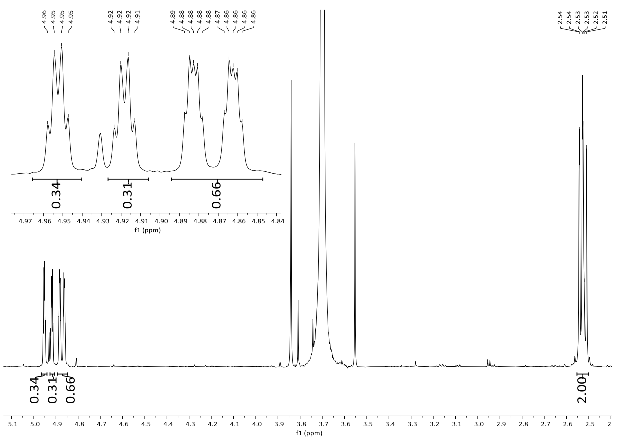

run 2

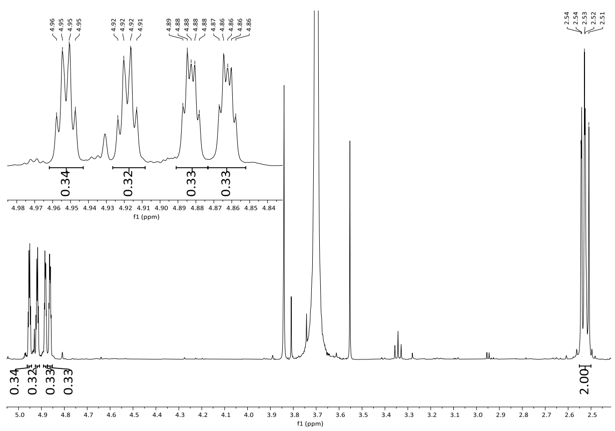

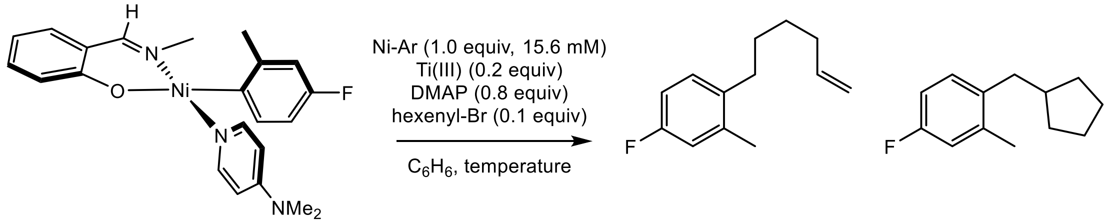

Linear to cyclized ratio was determined by ^{19}F NMR.

run 1

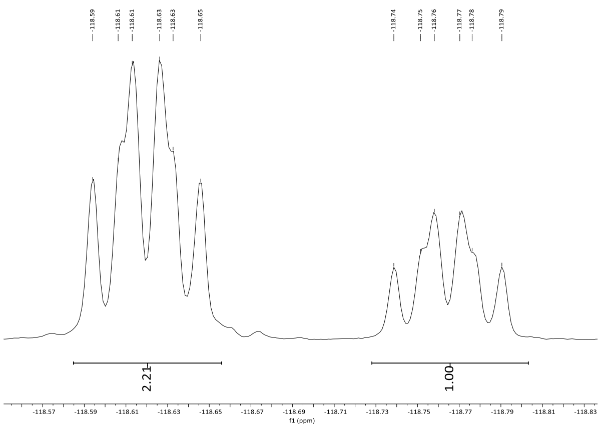

run 2

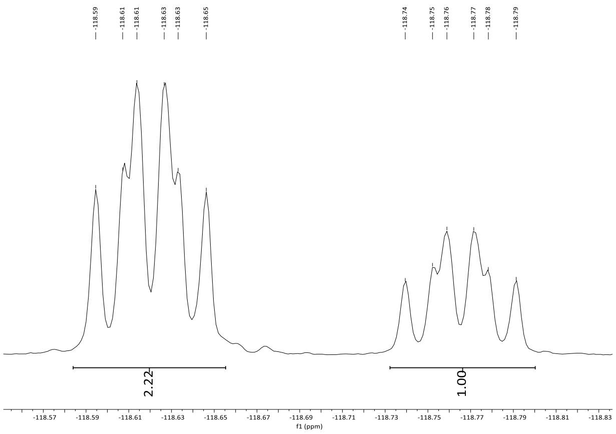

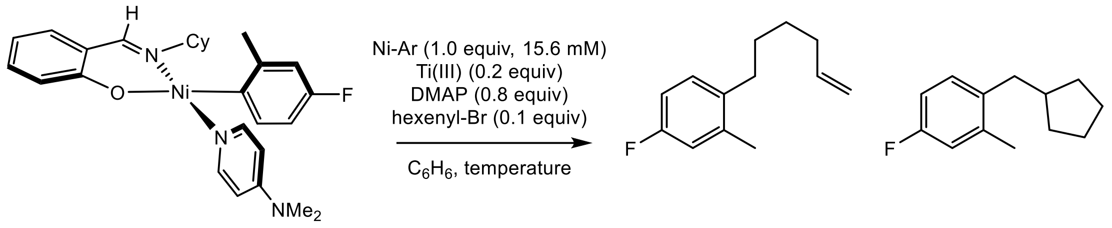

Linear to cyclized ratio was determined by ^{19}F NMR.

run 1

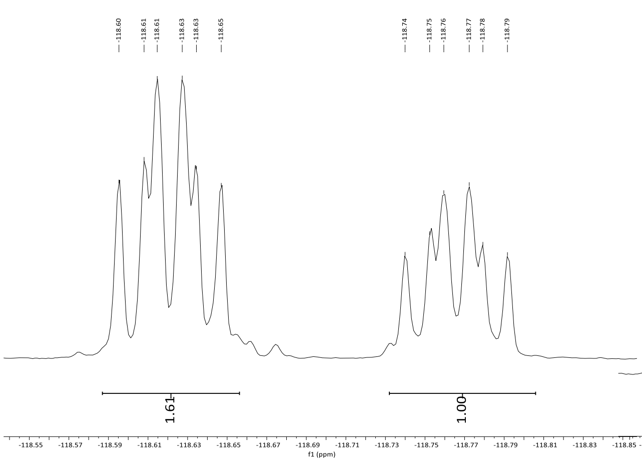

run 2

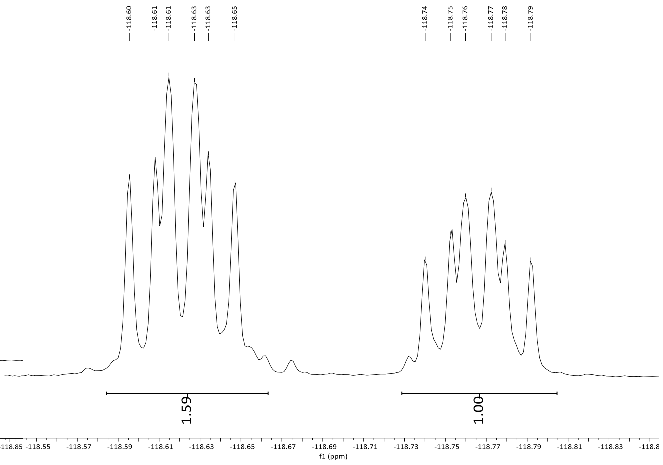

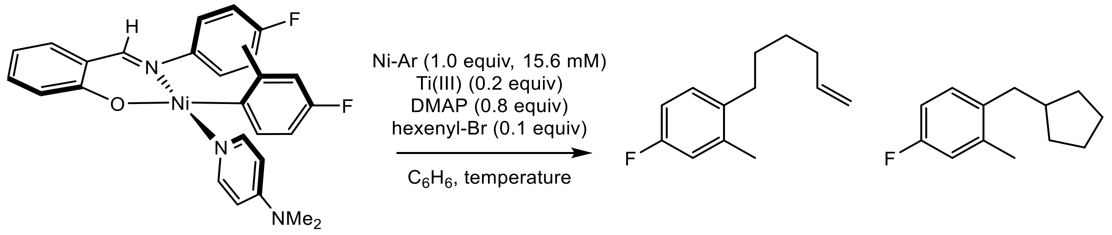

Linear to cyclized ratio was determined by ^{19}F NMR.

run 1

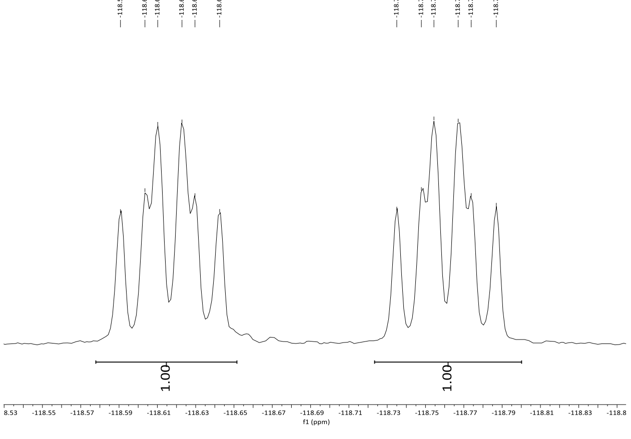

run 2

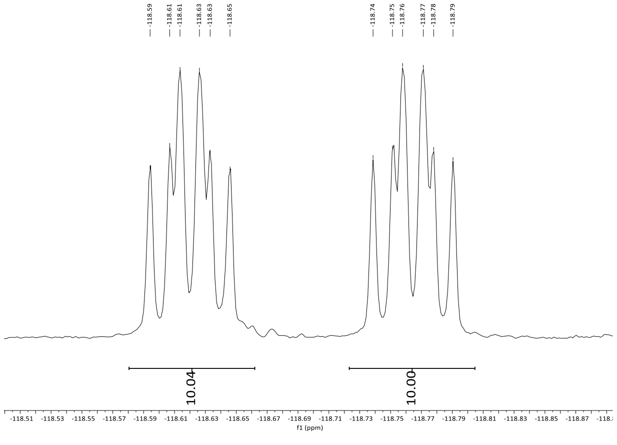

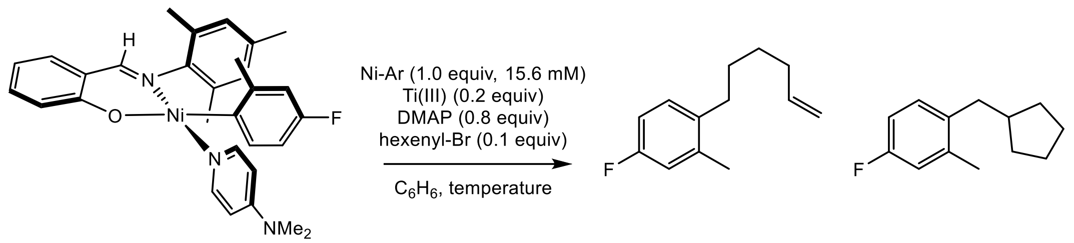

Linear to cyclized ratio was determined by ^{19}F NMR.

run 1 run 2

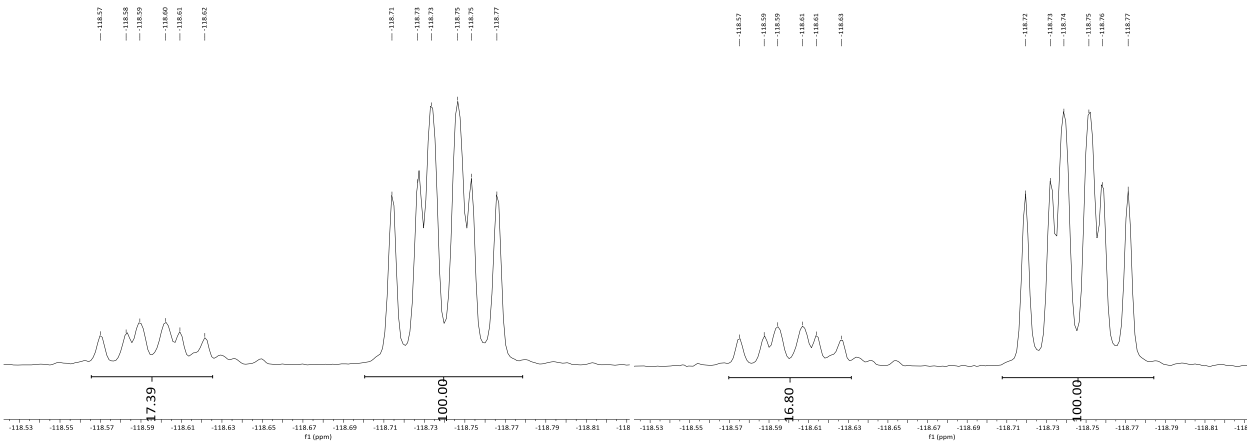

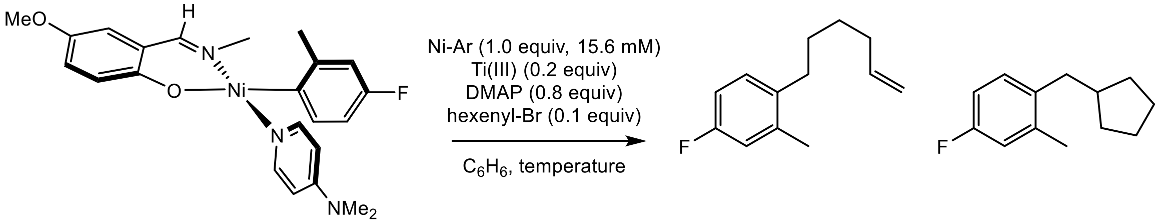

Linear to cyclized ratio was determined by ^{19}F NMR.

run 1 run 2

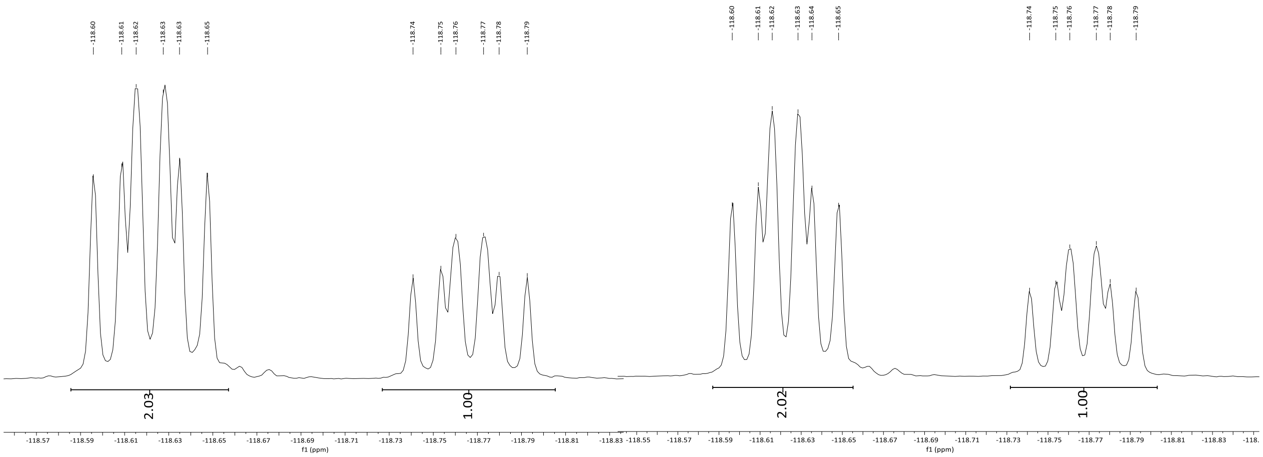

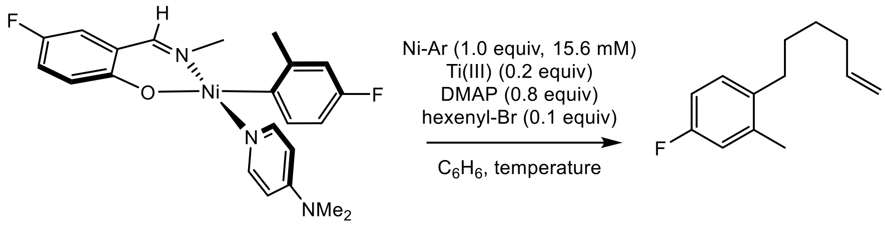

Linear to cyclized ratio was determined by ^{19}F NMR.

run 1

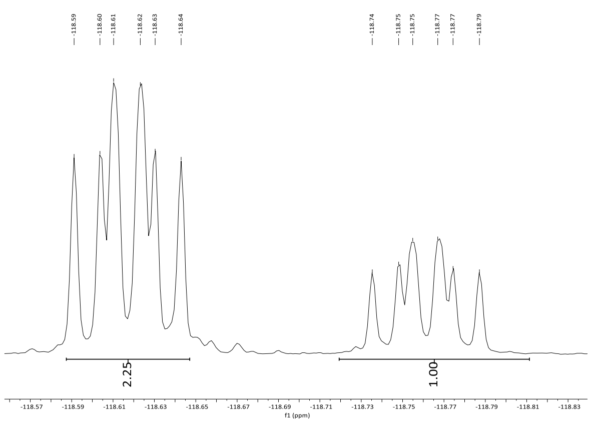

run 2

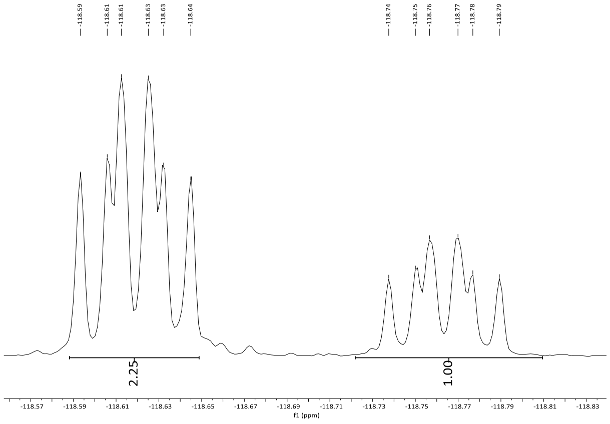

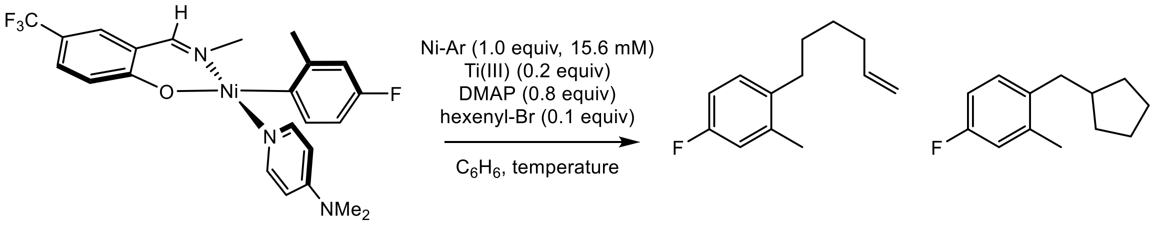

Linear to cyclized ratio was determined by ^{19}F NMR.

run 1

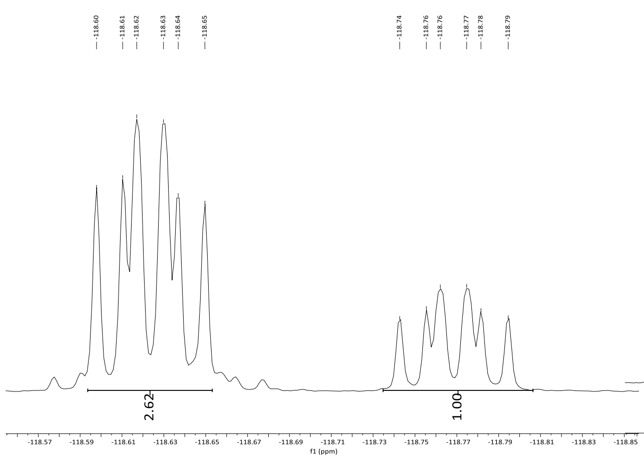

run 2

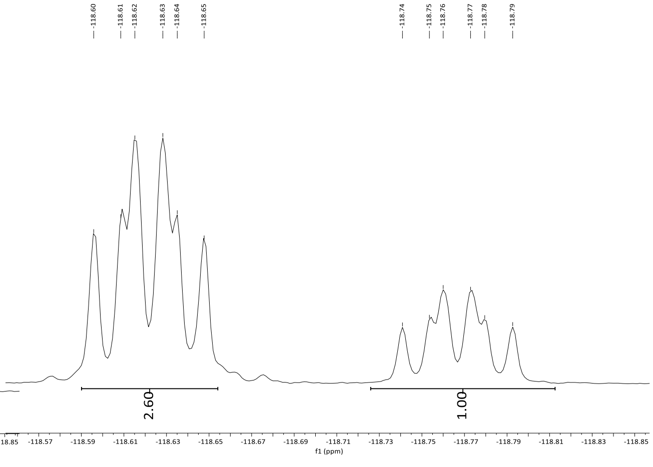

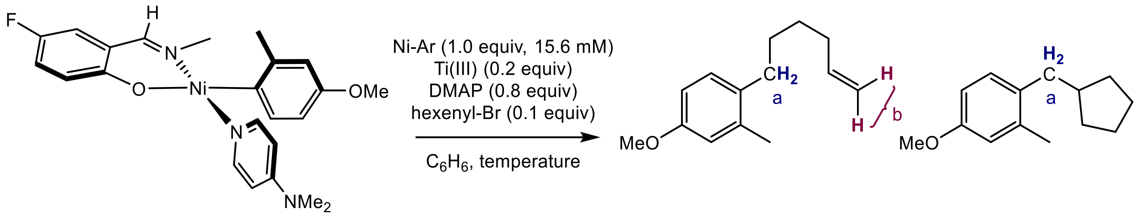

Linear to cyclized ratio was determined by ^{1}H NMR, ratio = $\frac{b}{a-b}$

run 1

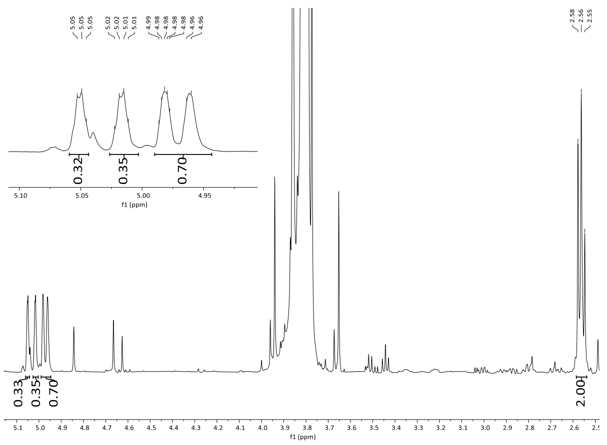

run 2

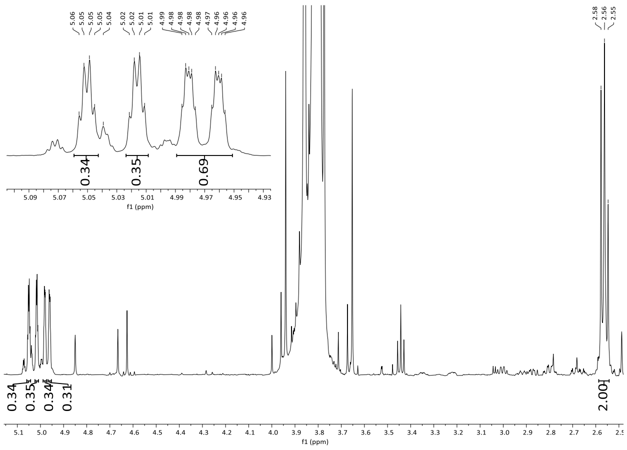

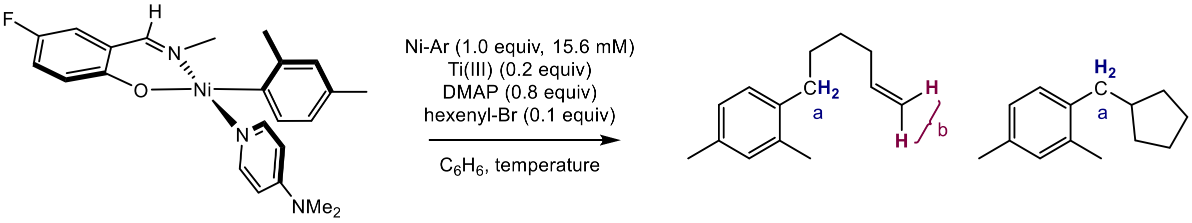

Linear to cyclized ratio was determined by ^{1}H NMR, ratio = $\frac{b}{a-b}$

run 1

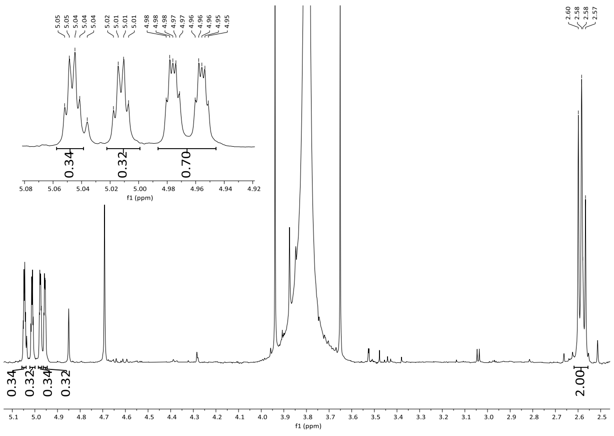

run 2

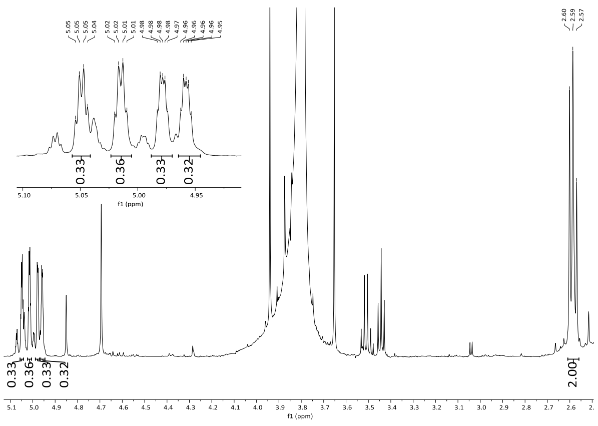

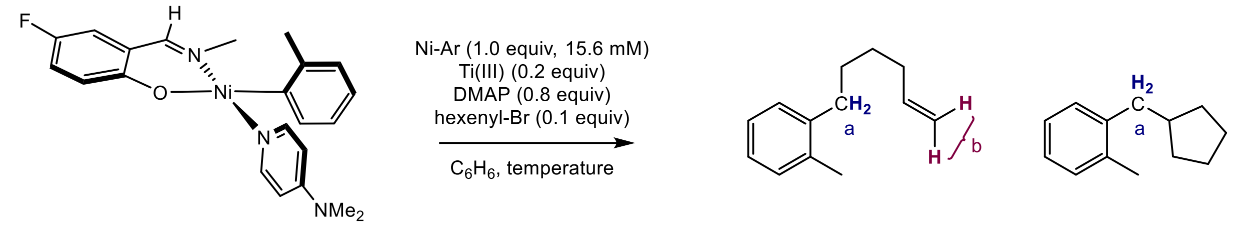

Linear to cyclized ratio was determined by ^{1}H NMR, ratio = $\frac{b}{a-b}$

run 1

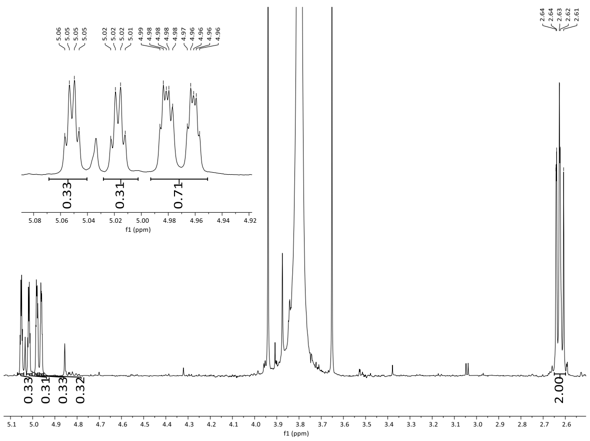

run 2

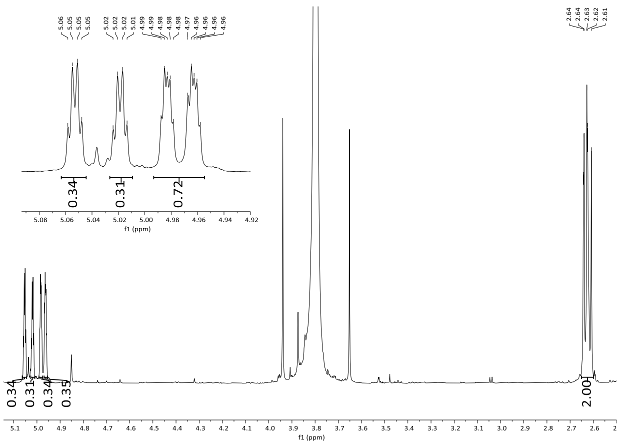

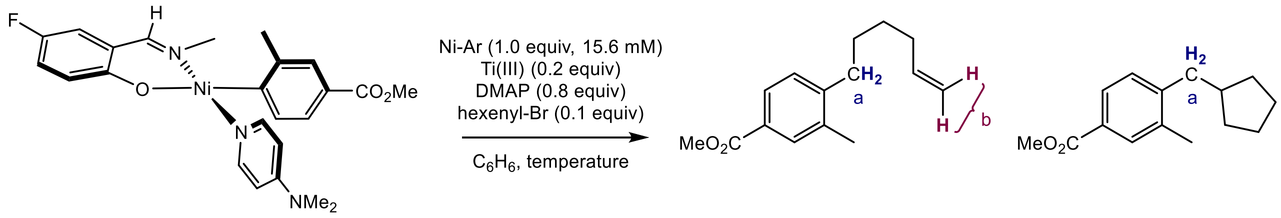

Linear to cyclized ratio was determined by ^{1}H NMR, ratio = $\frac{b}{a-b}$

run 1

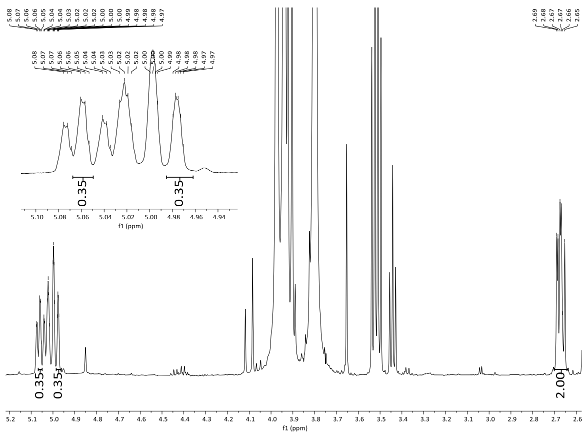

run 2

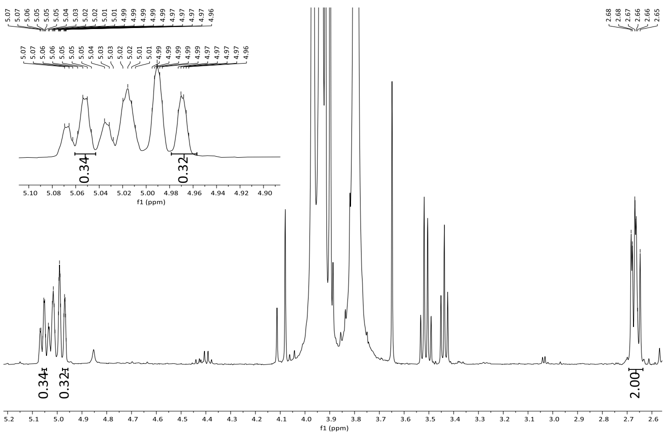

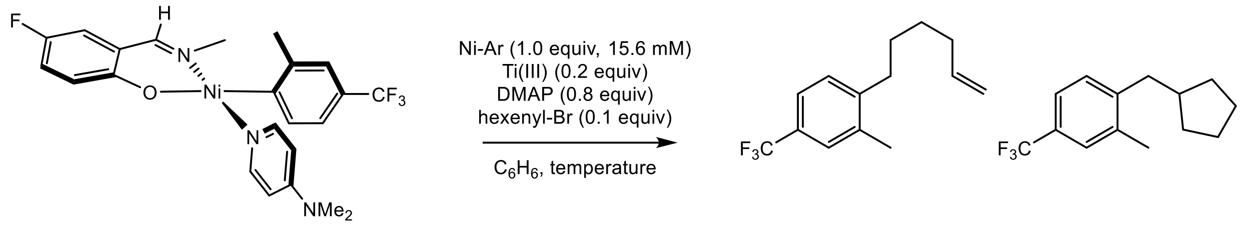

Linear to cyclized ratio was determined by ^{19}F NMR.

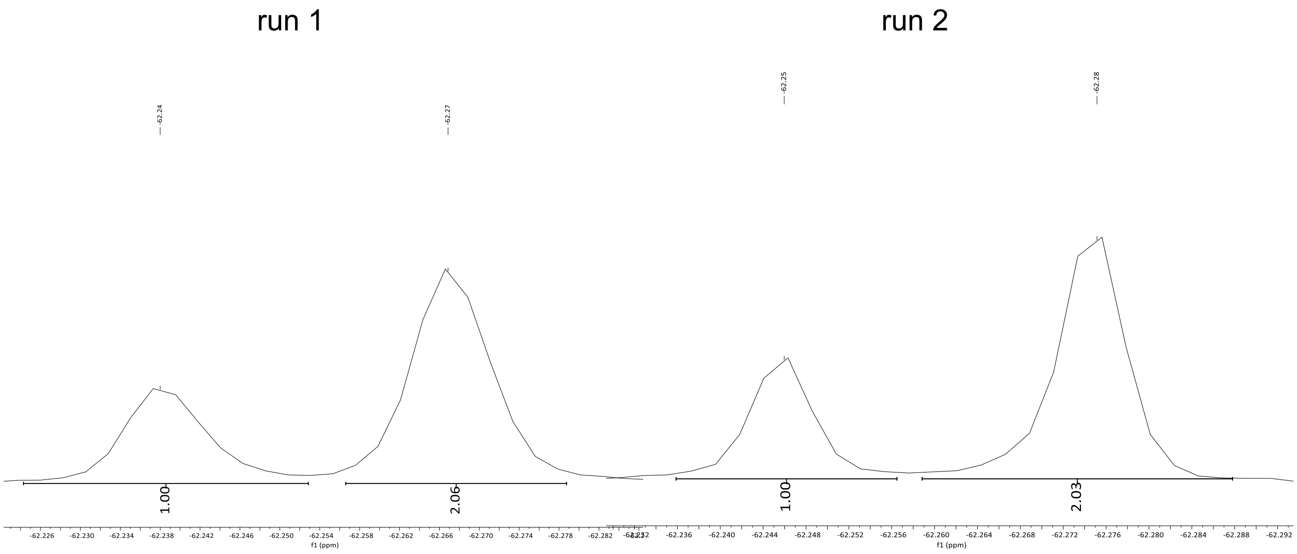

VI Intermolecular Competition Experiments

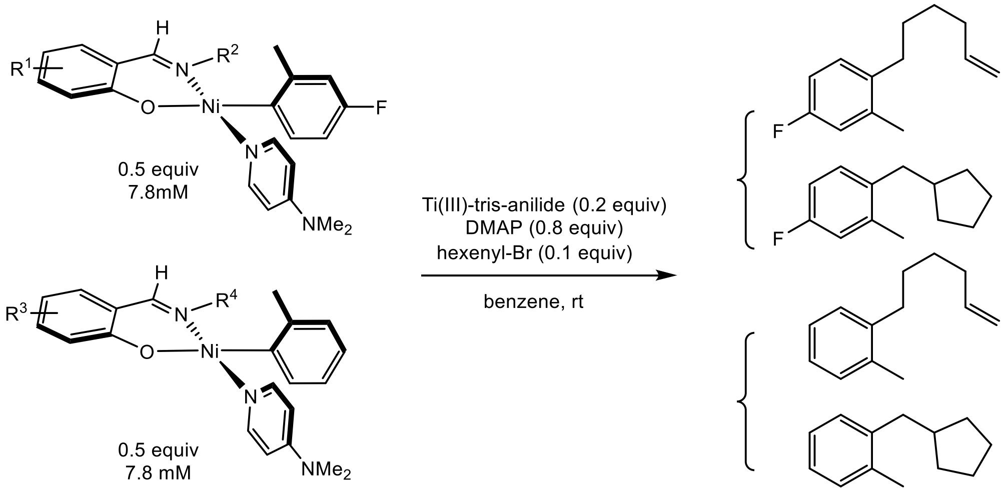

Competition Experiment: In a N_2-filled glovebox, a 20-mL vial with a stir bar was added a benzene solution of DMAP and Ti(III)-tris-anilide (0.6 mL, 0.033 M (Ti), 0.132 M (DMAP), 0.2 equiv) and a benzene solution of two FI-Ni-Ar complexes were added. (2.5 mL, 0.02 M, 0.05 mmol, 0.5 equiv each). 0.7 mL benzene was then added to adjust concentration. A solution of hexenyl bromide was then added (0.1 mL, 0.1 M, 0.1 equiv). The reaction mixture was allowed to stir at room temperature for 1 h. Upon completion, the mixture was quenched with HCl (2M, aq.) and extracted with Et_2O. The organic layers were combined and concentrated under reduced pressure. Linear to cyclized product ratio was analyzed by ^{1}H NMR. The reaction was replicated two times for each set of competition.

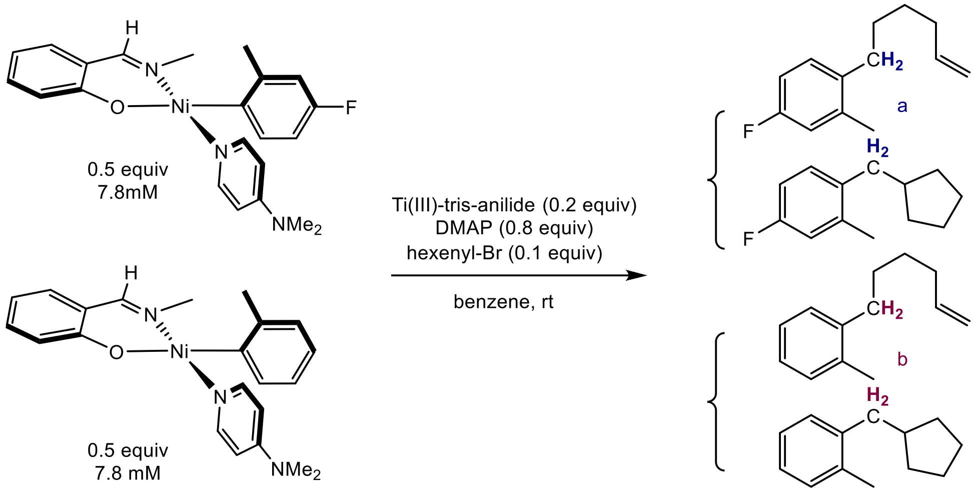

Competition ratio was determined by ^{1}H NMR, ratio = $\frac{a}{b}$

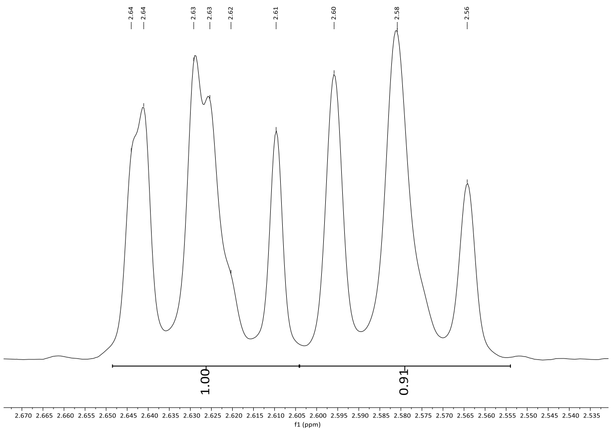

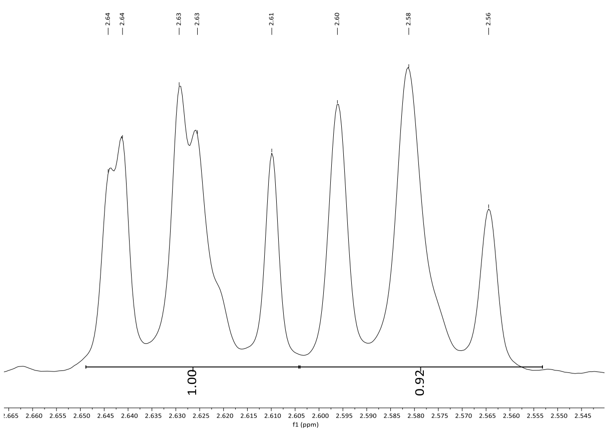

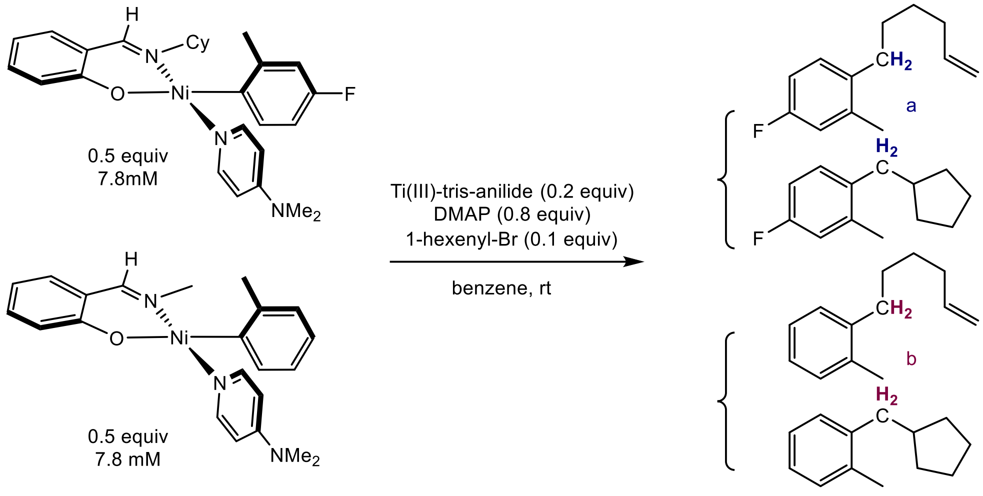

Competition ratio was determined by ^{1}H NMR, ratio = $\frac{a}{b}$

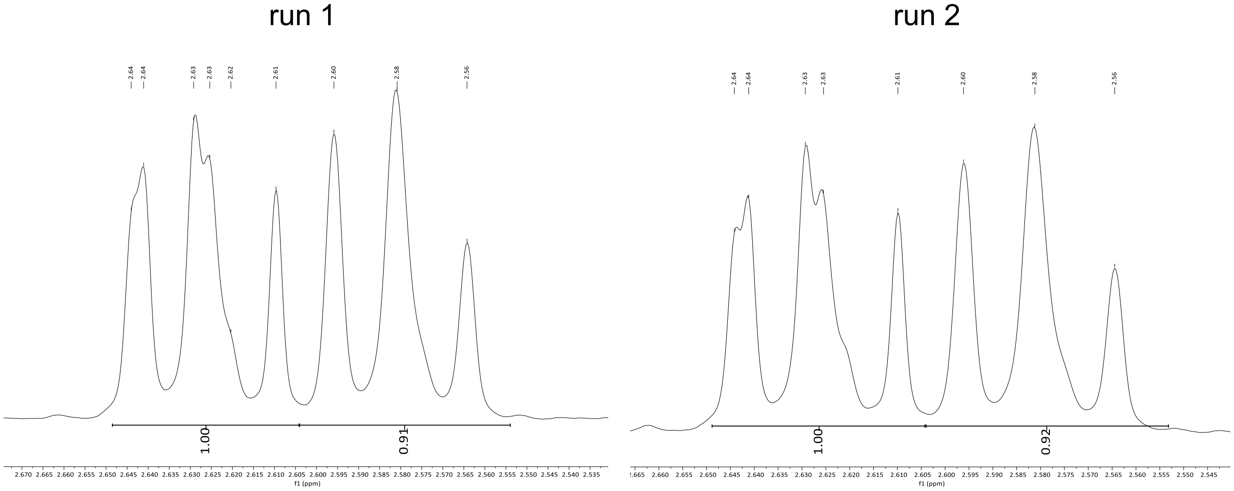

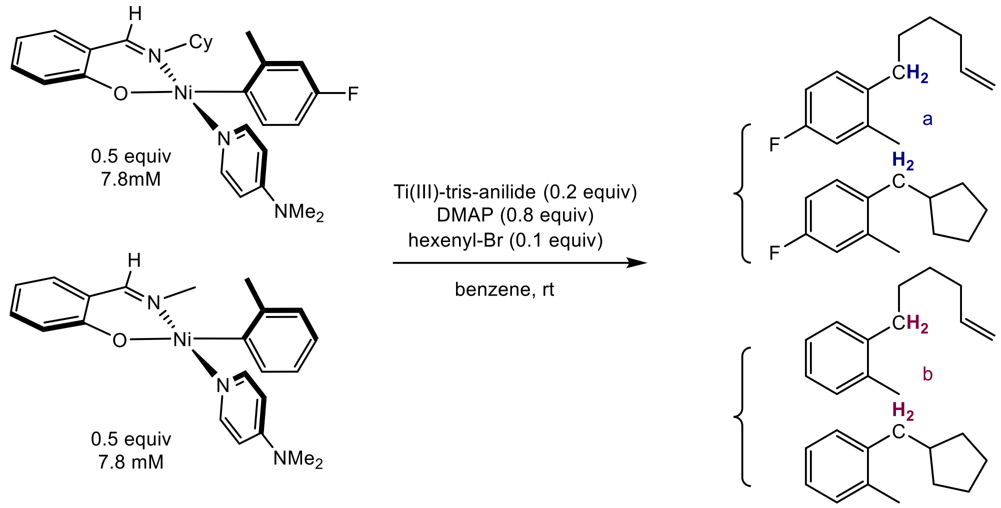

Competition ratio was determined by ^{1}H NMR, ratio = $\frac{a}{b}$

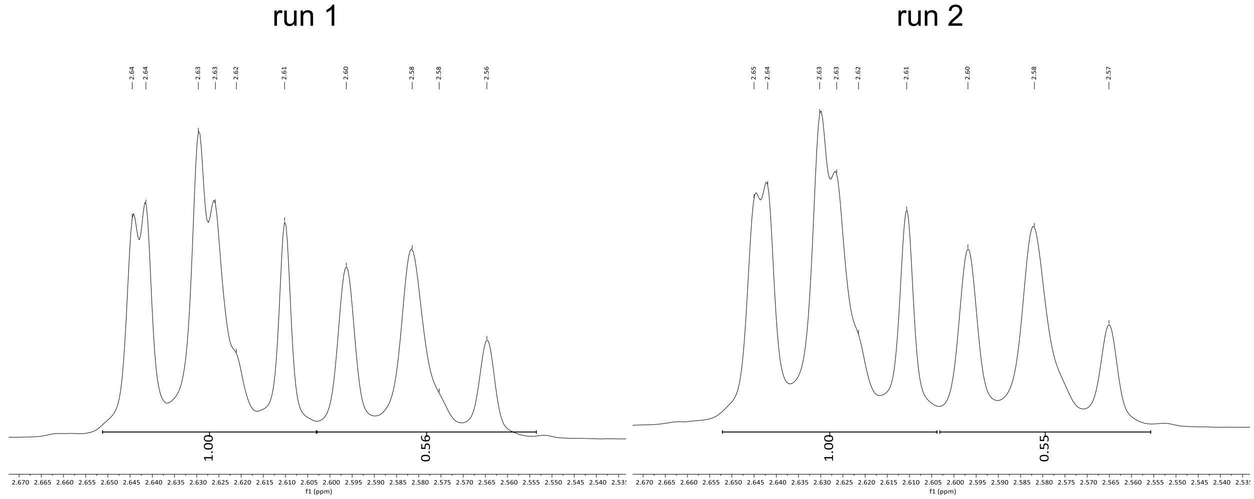

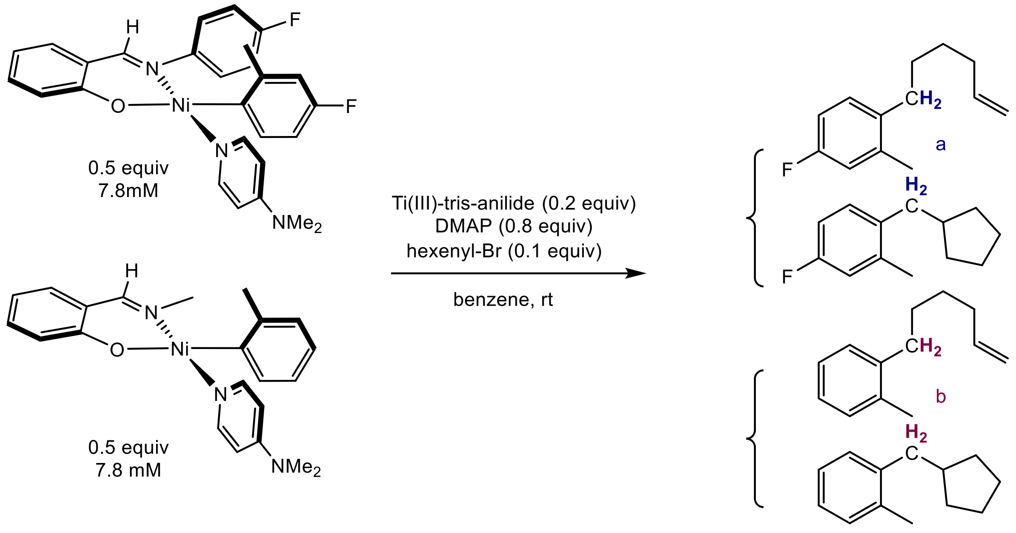

Competition ratio was determined by ^{1}H NMR, ratio = $\frac{a}{b}$

run 1

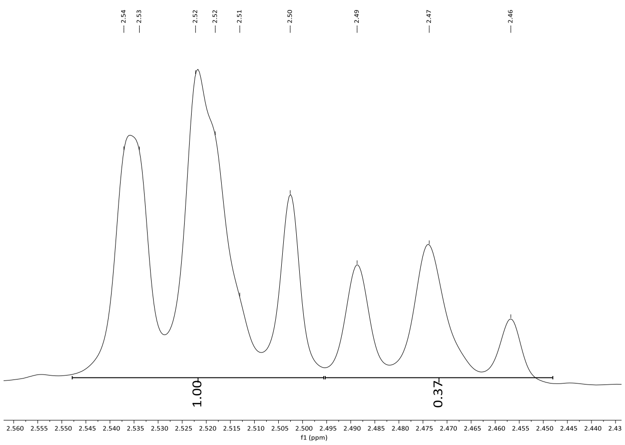

run 2

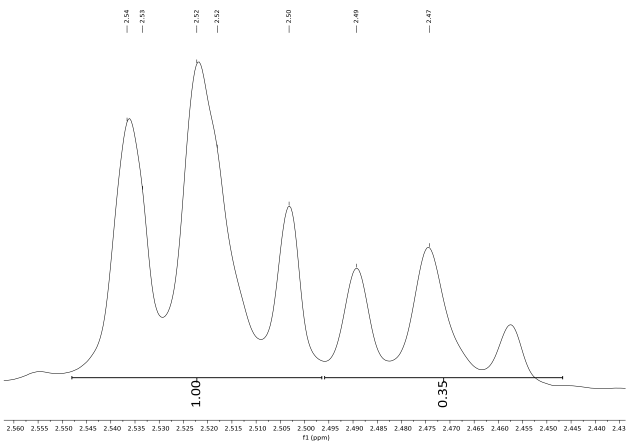

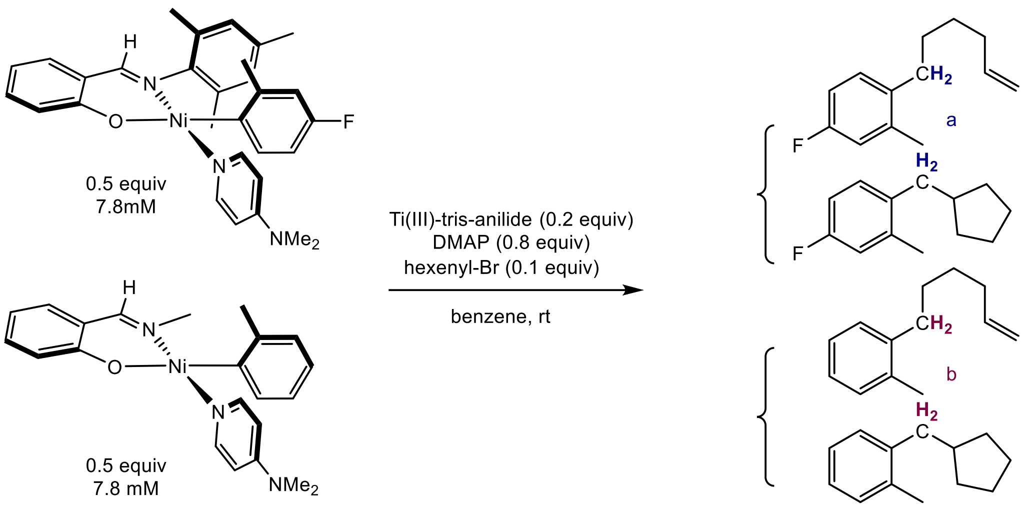

Competition ratio was determined by ^{1}H NMR, ratio = $\frac{a}{b}$

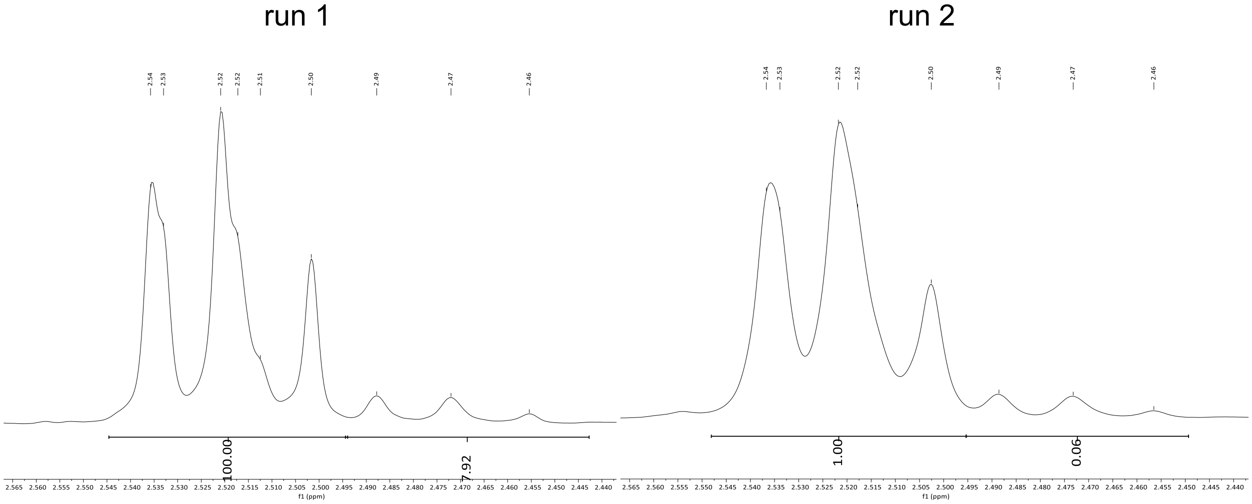

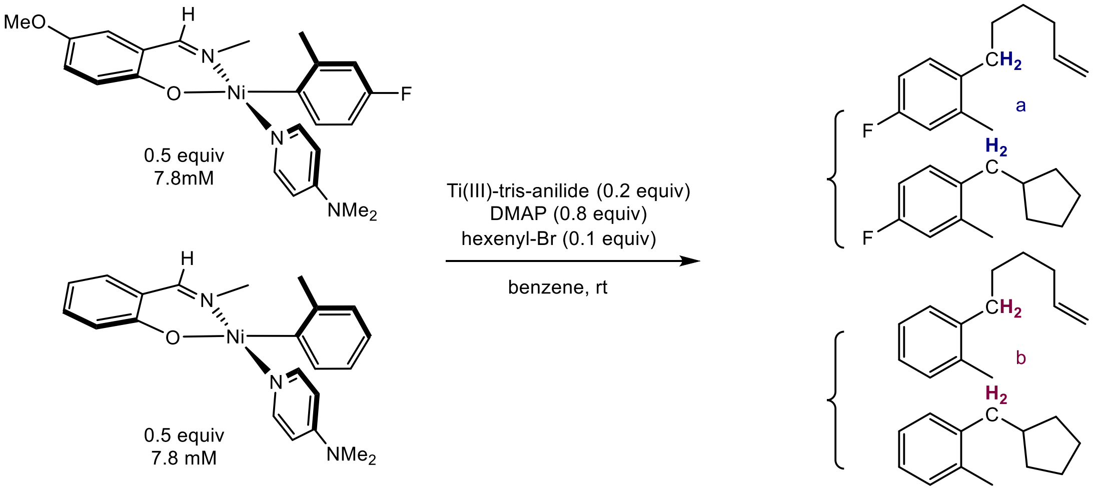

Competition ratio was determined by ^{1}H NMR, ratio = $\frac{a}{b}$

run 1

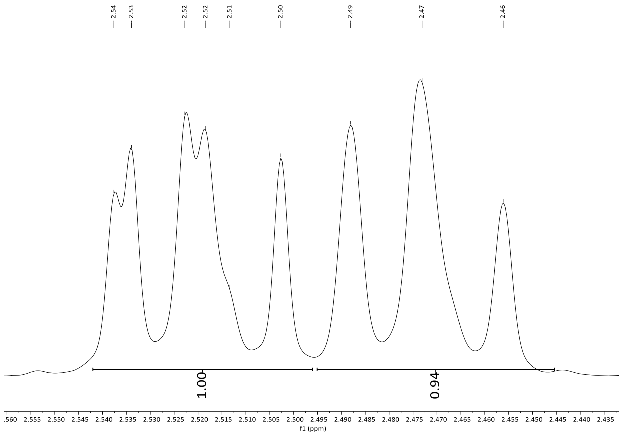

run 2

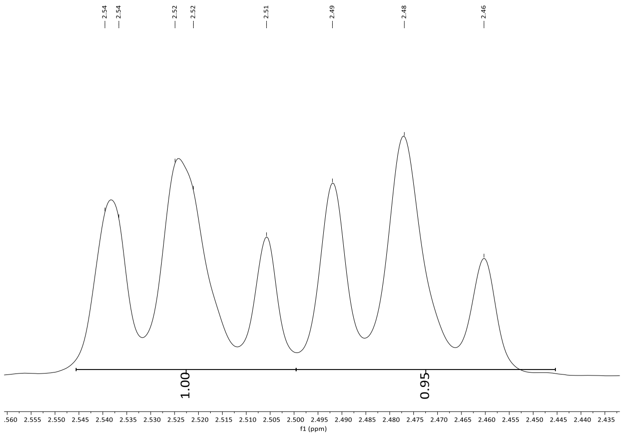

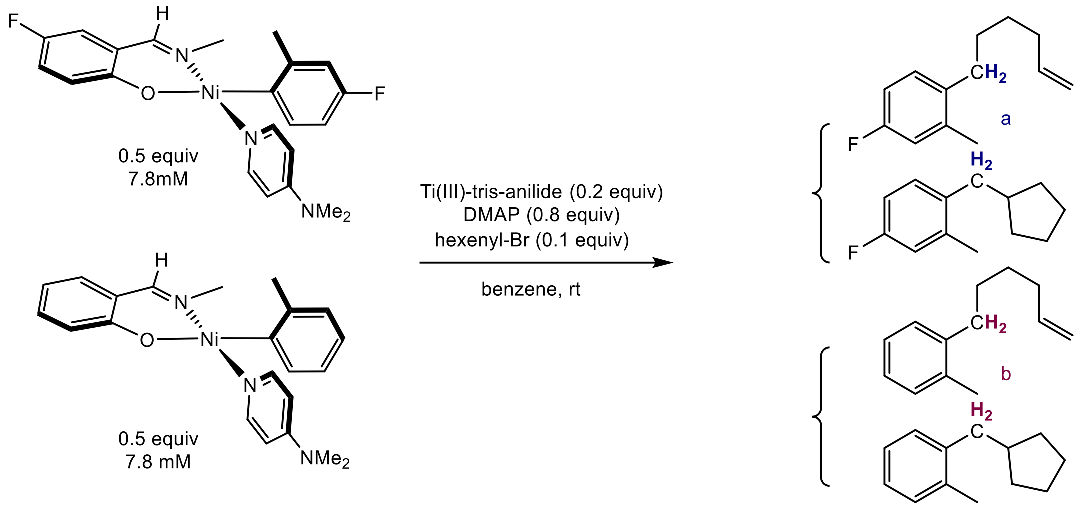

Competition ratio was determined by ^{1}H NMR, ratio = $\frac{a}{b}$

run 1

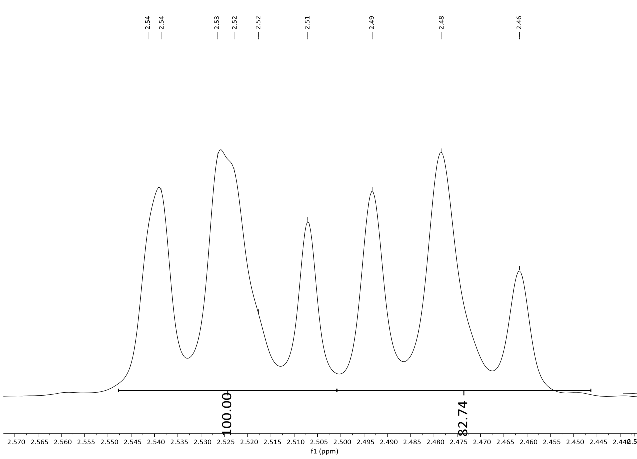

run 2

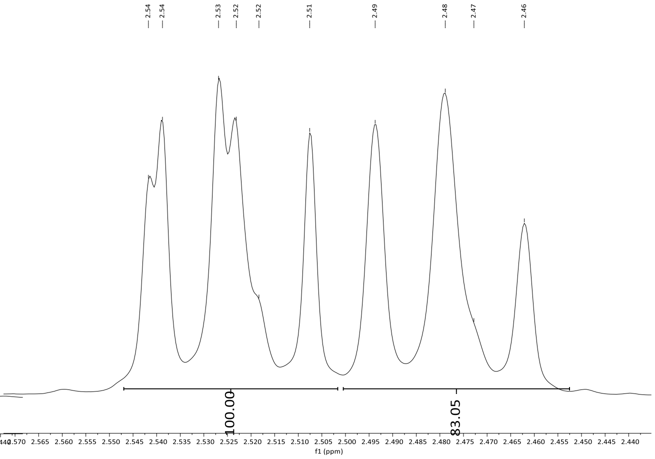

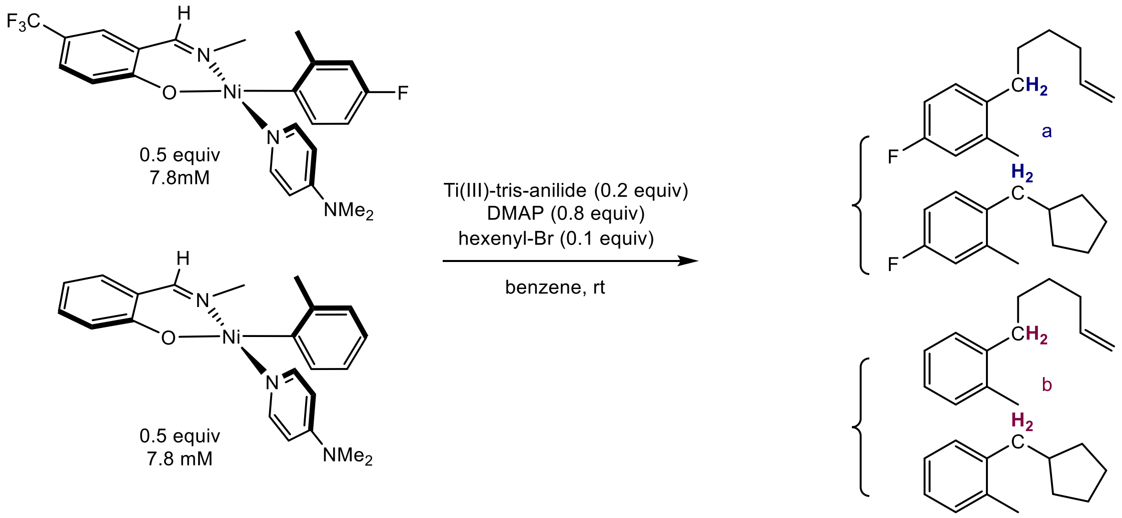

Competition ratio was determined by ^{1}H NMR, ratio = $\frac{a}{b}$

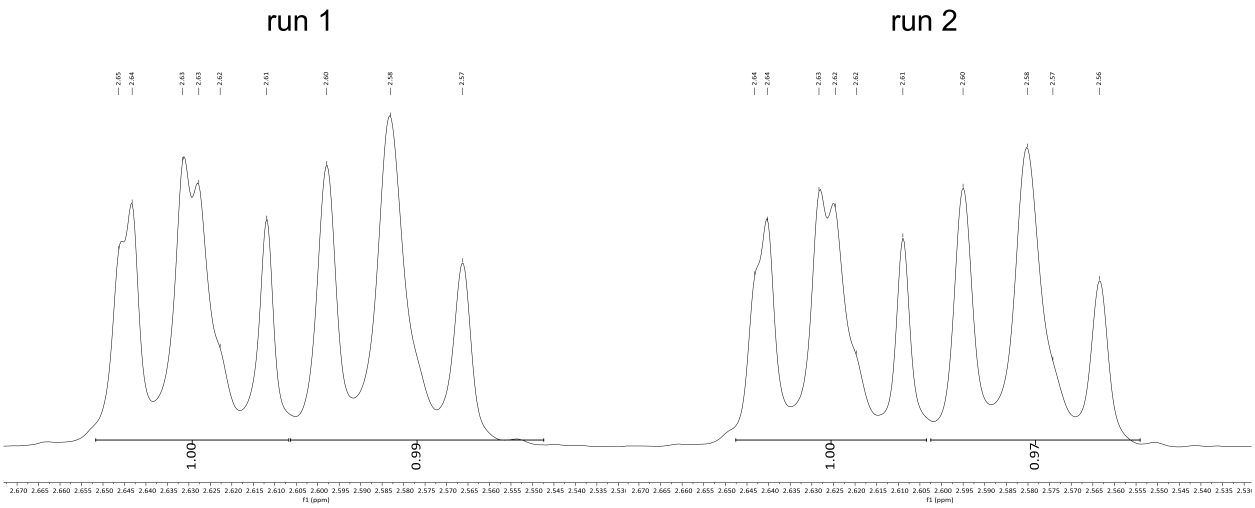

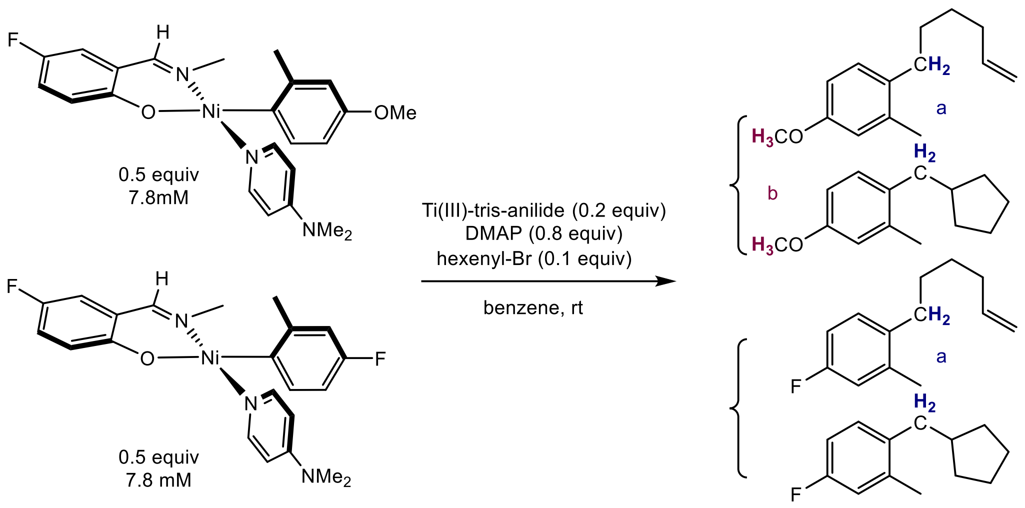

Competition ratio was determined by ^{1}H NMR, ratio = $\frac{b\frac{2}{3}}{a-b\frac{2}{3}}$

run 1

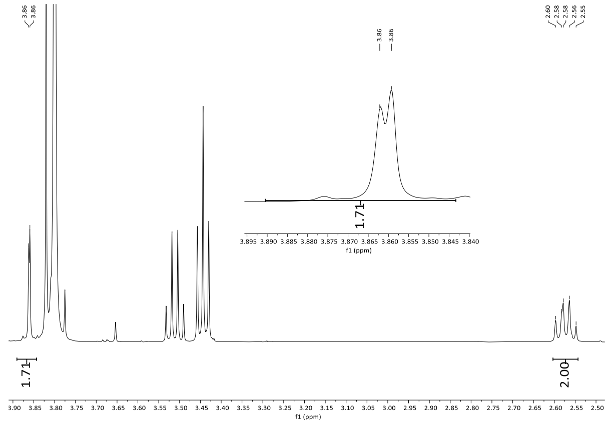

run 2

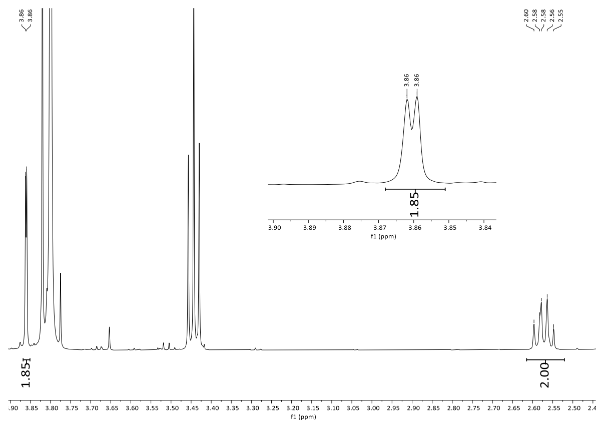

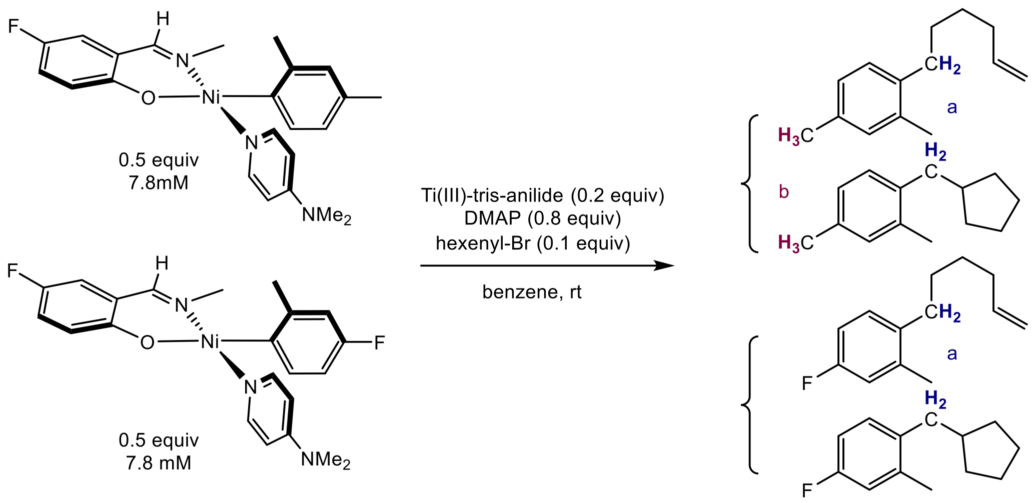

Competition ratio was determined by ^{1}H NMR, ratio = $\frac{b\frac{2}{3}}{a-b\frac{2}{3}}$

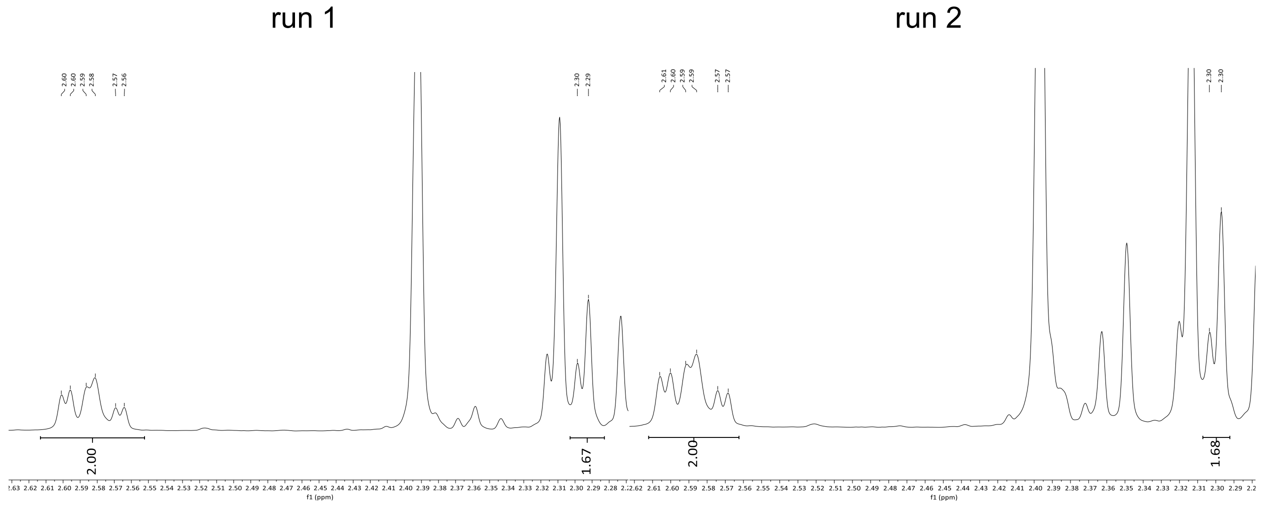

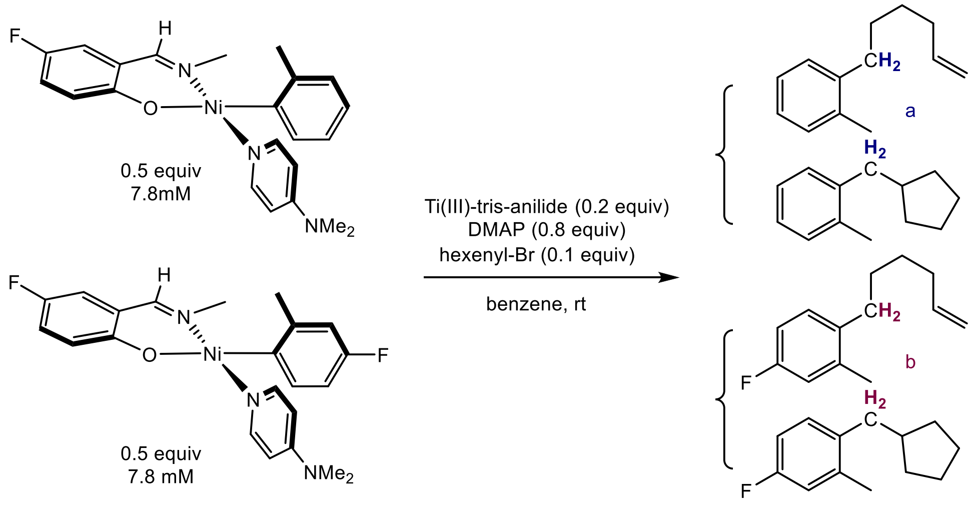

Competition ratio was determined by ^{1}H NMR, ratio = $\frac{a}{b}$

run 1

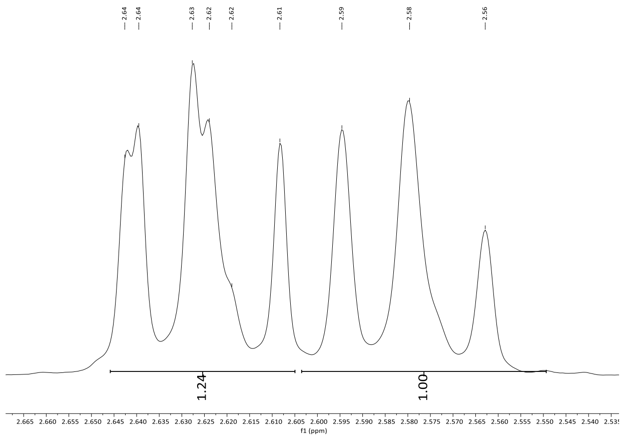

run 2

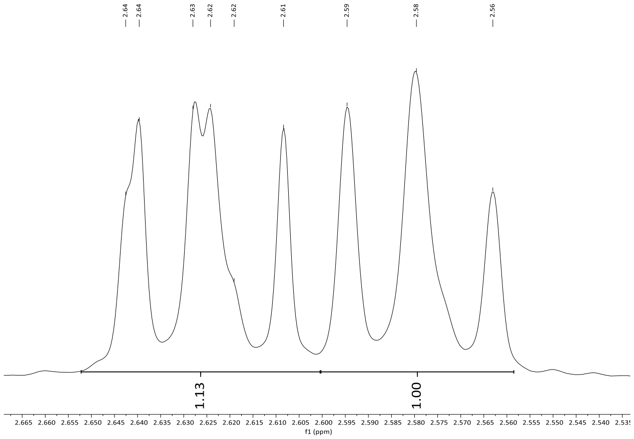

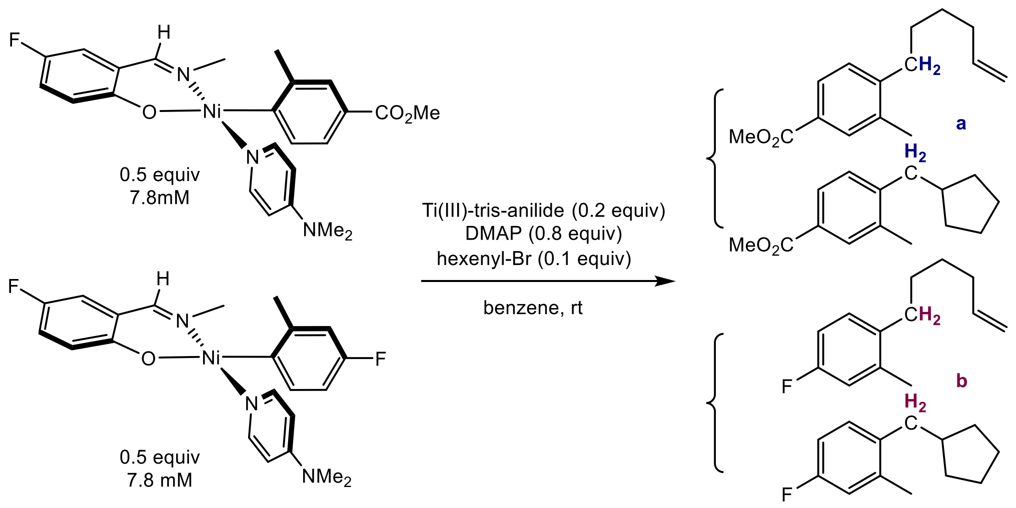

Competition ratio was determined by ^{1}H NMR, ratio = $\frac{a}{b}$

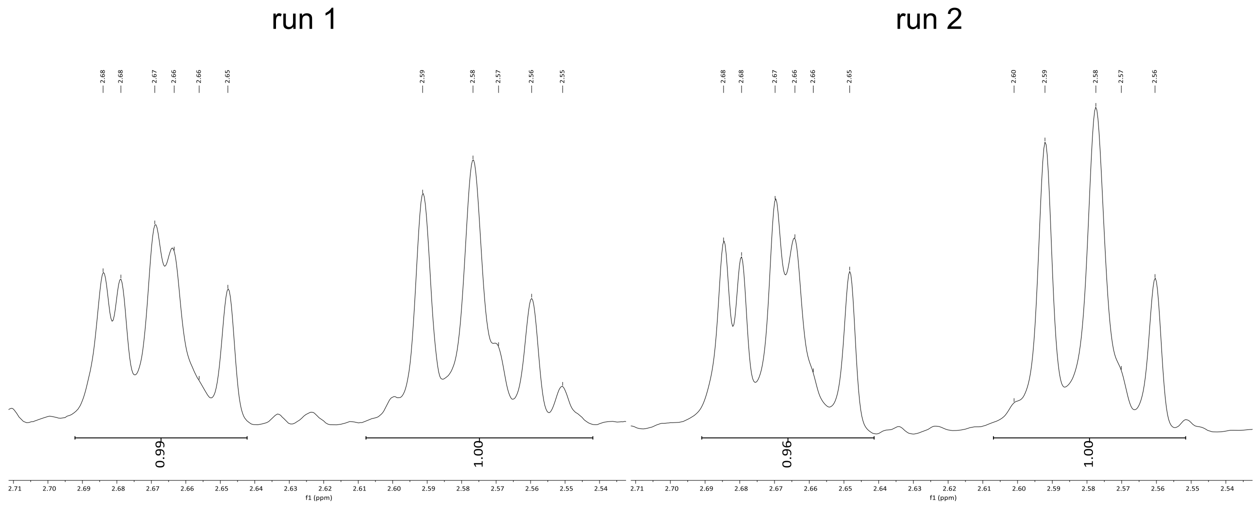

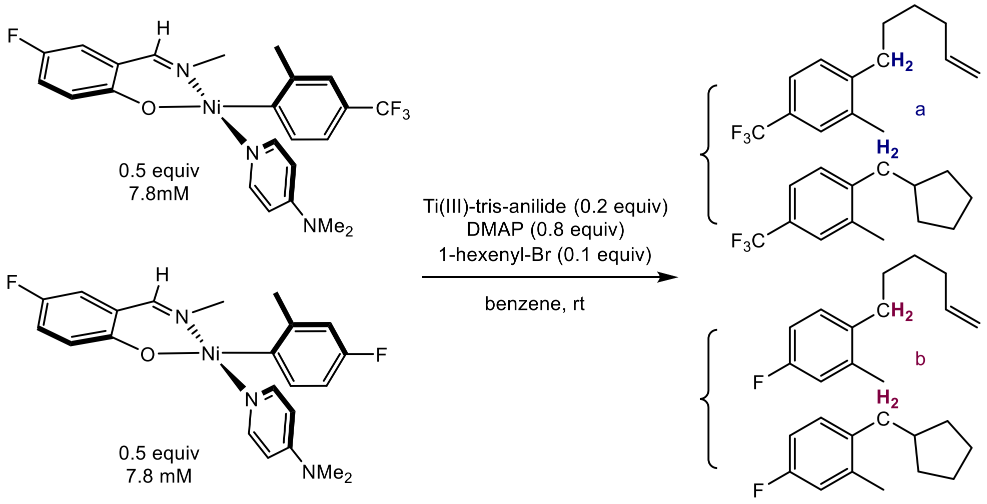

Competition ratio was determined by ^{1}H NMR, ratio = $\frac{a}{b}$

run 1

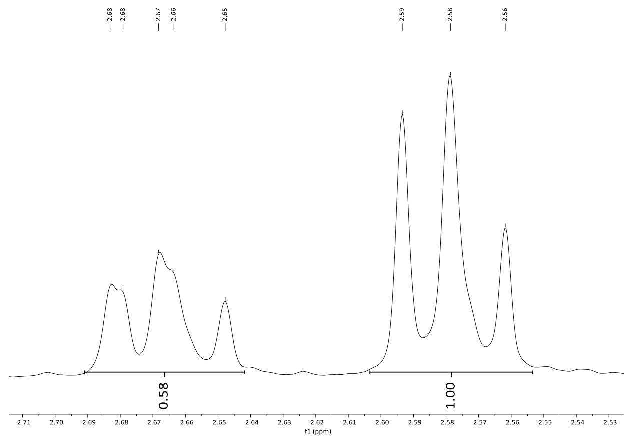

run 2

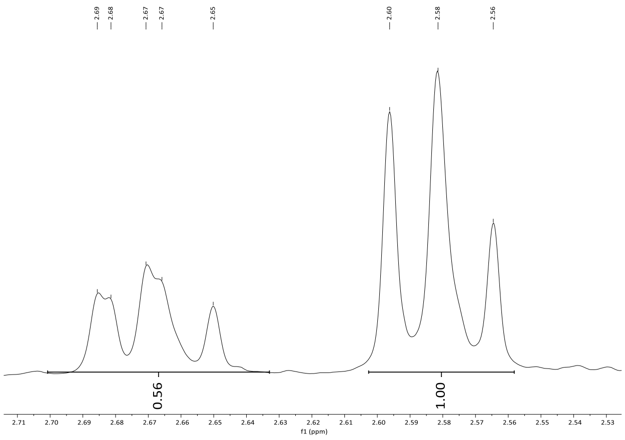

VII Comparison of Radical Clock and Competition Experiments

parallel experiments

$$\frac{\dfrac{\text{l-Alkyl-Ar}^1}{\text{c-Alkyl-Ar}^1}}{\dfrac{\text{l-Alkyl-Ar}^2}{\text{c-Alkyl-Ar}^2}} = \frac{\dfrac{k_1 \bullet [\text{Ni-Ar}^1]}{k_c}}{\dfrac{k_2 \bullet [\text{Ni-Ar}^2]}{k_c}} = \frac{k_1}{k_2}$$

intermolecular competition

$$\frac{\text{Alkyl-Ar}^1}{\text{Alkyl-Ar}^2} = \frac{k_1}{k_2}$$

Table S3. Quotent of rate constant between a pair of nickel complexes calculated with parallel experiments (radical clock) and intermolecular competition under single step kinetic assumptions.

entry	competition	deviation	radical clock	deviation
1	1.37	0.06	0.98	0.04
2	1.27	0.01	0.92	0.07
3	1.19	0.08	0.94	0.07
4	0.98	0.02	0.8	0.07
5	0.61	0.05	0.91	0.02
6	0.83	0.01	1.17	0.03
7	0.95	0.01	1.05	0.01
8	0.98	0.01	1.36	0.01
9	0.92	0.01	1.15	0.01
10	0.07	0.01	0.09	0.01
11	0.36	0.01	0.53	0.02
12	0.56	0.01	0.83	0.01

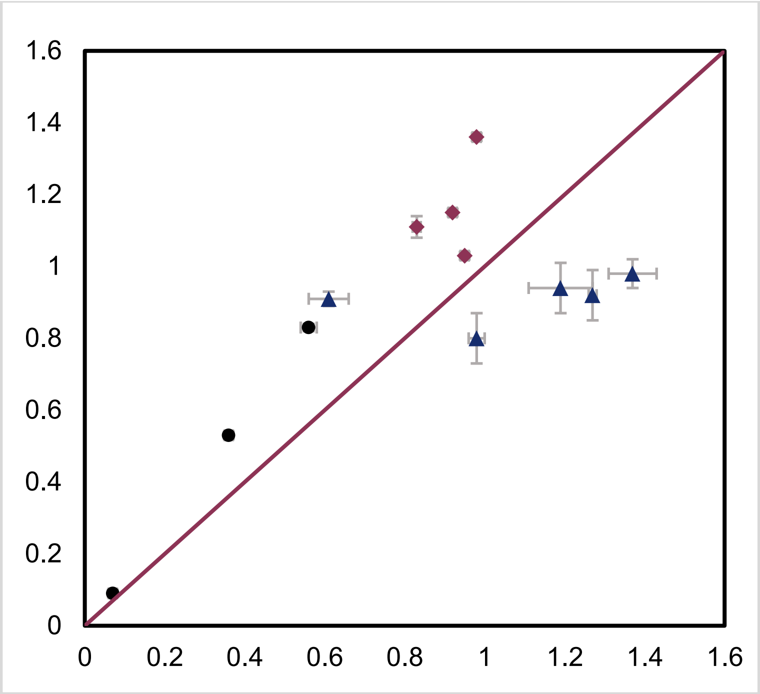

Figure S59. Comparison of parallel experiments (radical clock) or intermolecular competition

VIII $^{12}C/^{13}C$ Kinetic Isotope Effects

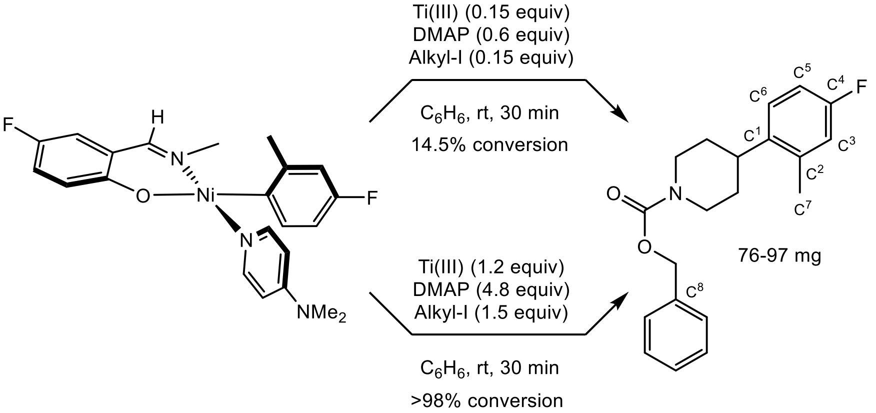

Kinetic Isotope Effect Experiment (low conversion reaction): In a N_2-filled glovebox, a round-bottom flask with a stir bar was added Ti(III)-tris-anilide (173 mg, 0.3 mmol, 0.15 equiv), DMAP (147 mg, 1.2 mmol, 0.6 equiv) and 2 mL benzene. ($Me^{F}FI$)Ni(2-Me-4-F-C_6H_3)(DMAP) (893 mg, 2.02 mmol. 1.0 equiv) was added followed by 38 mL benzene. Benzyl 4-iodopiperidine-1-carboxylate (104 mg, 0.3 mmol, 0.15 equiv) was added, and the reaction mixture was allowed to stir at room temperature for 1 h. The reaction mixture was monitored by ^{19}F NMR (14.5% conversion). Upon completion, the mixture was quenched with HCl (2M, aq.) and extracted with Et_2O. The organic layers were combined and concentrated under reduced pressure, the resulting crude oil was purified by column chromatography to afford the desired product as a colorless oil (76 mg, 0.22 mmol, 11% yield).

Kinetic Isotope Effect Experiment (full conversion reaction): In a N_2-filled glovebox, a round-bottom flask with a stir bar was added Ti(III)-tris-anilide (208 mg, 0.36 mmol, 1.2 equiv), DMAP (176 mg, 1.44 mmol, 4.8 equiv) and 2 mL benzene. ($Me^{F}FI$)Ni(2-Me-4-F-C_6H_3)(DMAP) (133 mg, 0.3 mmol. 1.0 equiv) was added followed by 13 mL benzene. Benzyl 4-iodopiperidine-1-carboxylate (124 mg, 0.36 mmol, 1.2 equiv) was added, and the reaction mixture was allowed to stir at room temperature for 1 h. The reaction mixture was monitored by ^{19}F NMR (>98% conversion). Upon completion, the mixture was quenched with HCl (2M, aq.) and extracted with Et_2O. The organic layers were combined and concentrated under reduced pressure, the resulting

crude oil was purified by column chromatography to afford the desired product as a colorless oil (97 mg, 0.29 mmol, 99% yield).

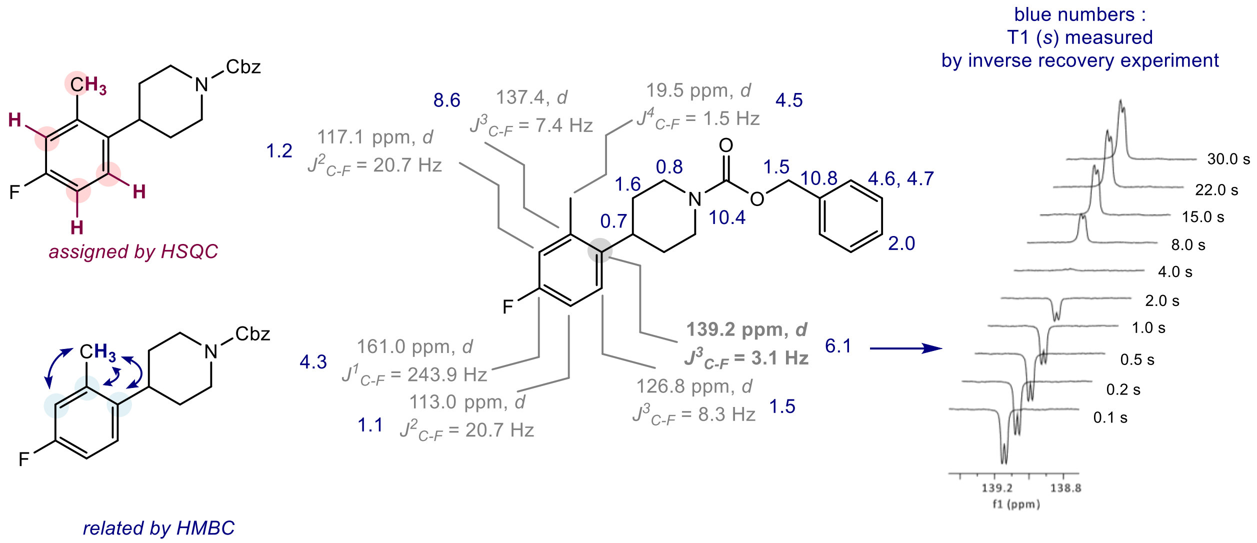

The isolated material was analyzed by quantitative ^{13}C NMR spectroscopy in a 5 mm NMR tube in $CDCl_3$. A total of 64 scans were collected. The T1 with carbon atoms were determined with inversion recovery experiment. It was found that the T1 value of C-8 was the longest (T1 = 10.8 s). To ensure full relaxation in between scans, the interscan delay was set to 120s (D1 > 10T1).

Both NMR spectra were processed with MestReNova in the same way. The auto phase correction and baseline correction was conducted. The signals of interest were integrated to include the C-C coupling satellites. The calculation method for integration was set to sum. Each sample was analyzed with the above mentioned method for 3 times.

The KIE was determined according to the following equation:

$$\frac{k_1}{k_2} = \frac{\ln(1-F)}{\ln(1-\frac{FR_p}{R_0})}$$

With F = conversion, R_0 = integration value of reference and R_p = integration value of respective carbon atom.

$^{12}C/^{13}C$ KIE calculated without reference sample

Kinetic isotope effect determined with C8 as internal standard without reference sample:

Table S4. $^{12}C/^{13}C$ KIE calculated without reference sample

run1

low conv. sample	C1	C2	C3	C4	C5	C6
1.0000 (C8)	0.9948	1.0055	1.0015	1.0021	1.0003	0.9998
ratio with C8	0.9948	1.0055	1.0015	1.0021	1.0003	0.9998
KIE	1.0057	0.9941	0.9984	0.9977	0.9997	1.0002

run2

low conv. sample	C1	C2	C3	C4	C5	C6
1.0000 (C8)	0.9981	0.9950	0.9959	0.9967	1.0051	0.9983
ratio with C8	0.9981	0.9950	0.9959	0.9967	1.0051	0.9983
KIE	1.0021	1.0054	1.0045	1.0036	0.9945	1.0018

run3

low conv. sample	C1	C2	C3	C4	C5	C6
1.0000 (C8)	0.9921	1.0117	0.9906	0.9936	1.0078	1.0014
ratio with C8	0.9921	1.0117	0.9906	0.9936	1.0078	1.0014
KIE	1.0086	0.9875	1.0103	1.0070	0.9916	0.9985

Kinetic isotope effect determined with C8 as internal standard without reference sample:

**KIE**	1.005774863	1.001652	1.007661	1.00309	1.00367	1.003108
deviation	0.005082316	0.015335	0.011661	0.004794	0.017248	0.003805

KIE calculated with reference sample reference sample

Kinetic isotope effect determined using the full conversion sample as internal standard.

C4 was assumed for 1.0000. For full conversion sample, trace amount of proto-dehalogenation impact the integration accuracy of C8, such that C4 was used as integration standard.

Table S5. $^{12}C/^{13}C$ KIE calculated with reference sample reference sample

full conv. sample	C1	C2	C3	C4	C5	C6
run 1	1.0091	0.9978	1.0094	1.0000	1.0039	1.0083
run 2	0.9985	0.9995	1.0067	1.0000	1.0049	0.9989
run 3	0.9967	1.0003	1.0023	1.0000	1.0058	0.9992
average	1.001433333	0.9992	1.006133	1.0000	1.004867	1.002133
deviation	0.006700249	0.001277	0.003584	0	0.00095	0.005343

low conv. sample	C1	C2	C3	C4	C5	C6
run 1	0.9948	1.0055	1.0015	1.0021	1.0003	0.9998
run 2	0.9980	1.0001	1.0002	0.9958	1.0009	0.9976
run 3	0.9921	1.0117	0.9906	0.9936	1.0078	0.10014
average	0.994966667	1.005767	0.9974333	0.9971667	1.0030	0.9996
C4(1.0000)	0.997793749	1.008624	1.000267	1	1.00585	1.00244
deviation	0.002953529	0.005805	0.005953	0.004412	0.004168	0.001908

**KIE**	1.0039488	0.98988430	1.00634855	1	0.998941749	0.999668549
deviation	0.00732234	0.005943	0.006949	0.004412	0.004275	0.005673

$$\Delta = \sqrt[2]{(\Delta_{ref})^2 + (\Delta_{sample})^2}$$

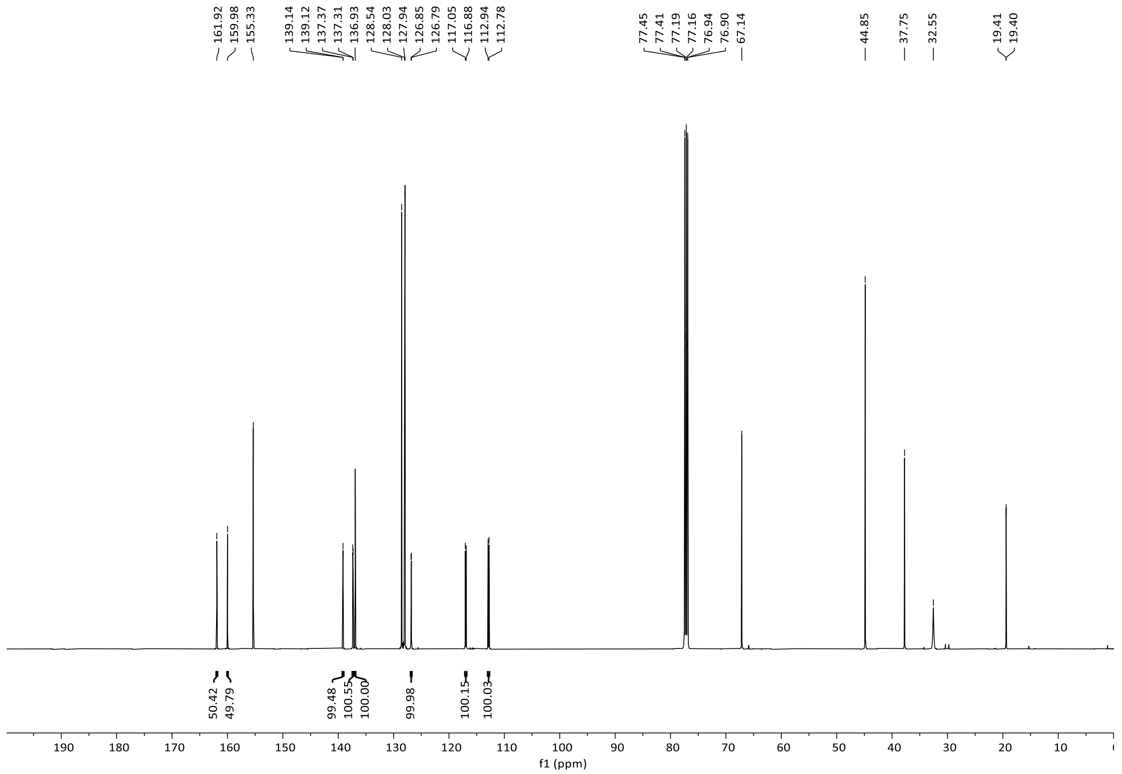

Figure S60. ^{13}C(quant) NMR of sample isolated from low conversion reaction-run1

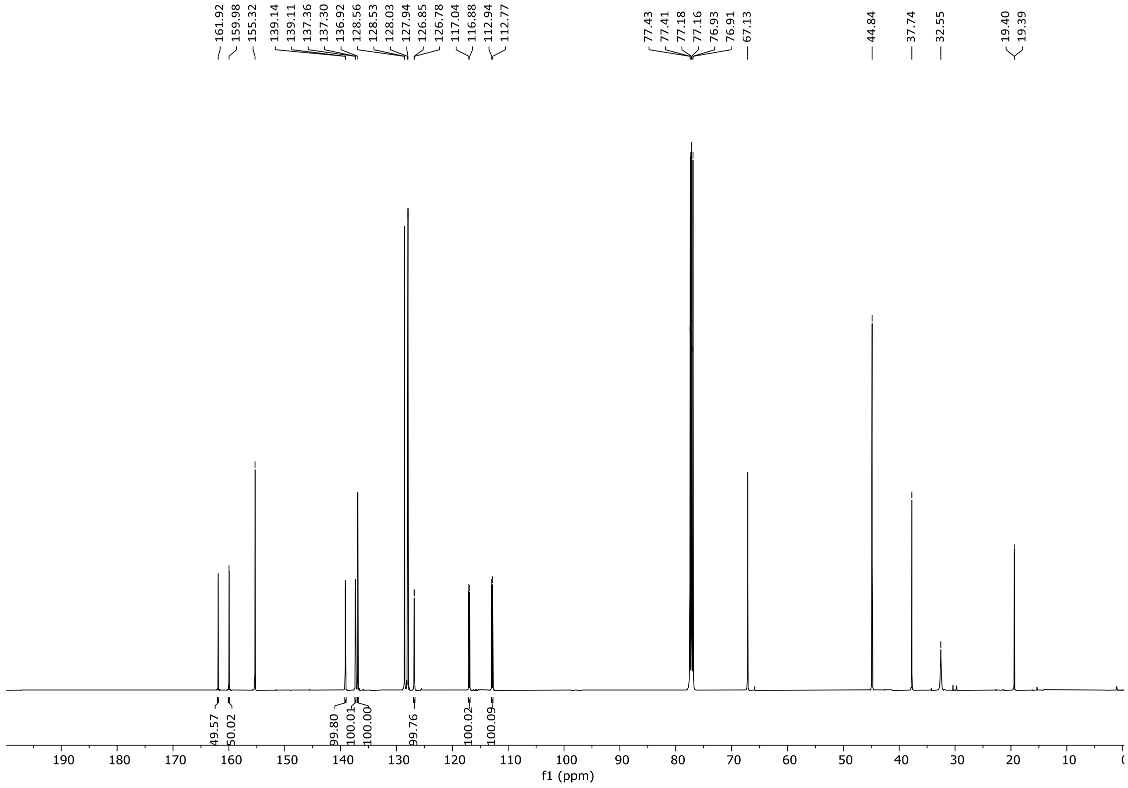

Figure S61. ^{13}C(quant) NMR of sample isolated from low conversion reaction-run2

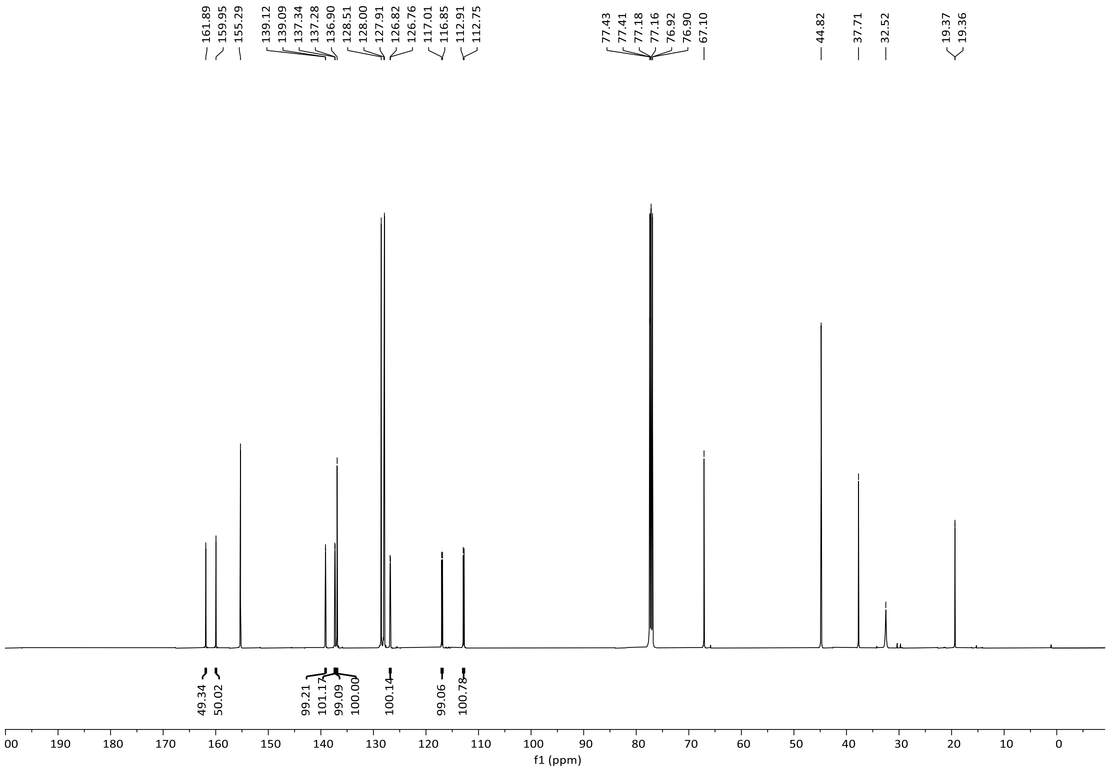

Figure S62. ^{13}C(quant) NMR of sample isolated from low conversion reaction-run3

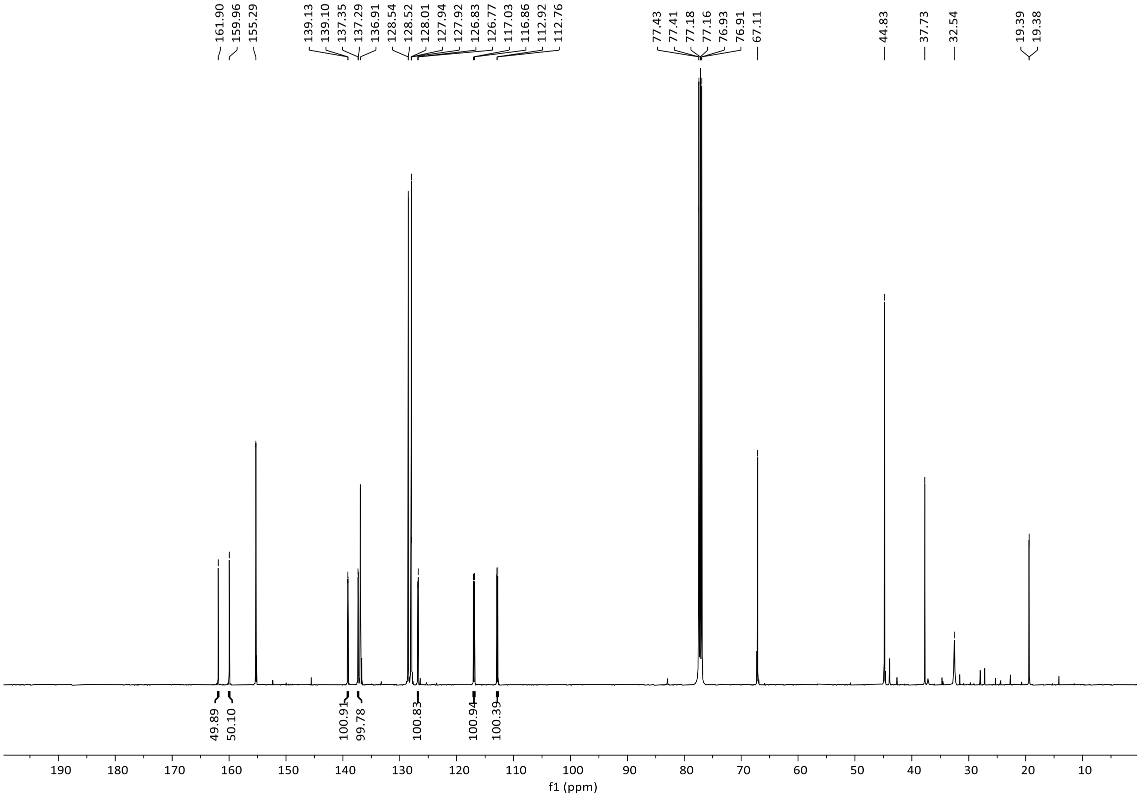

Figure S63. ^{13}C(quant) NMR of sample isolated from full conversion reaction-run1

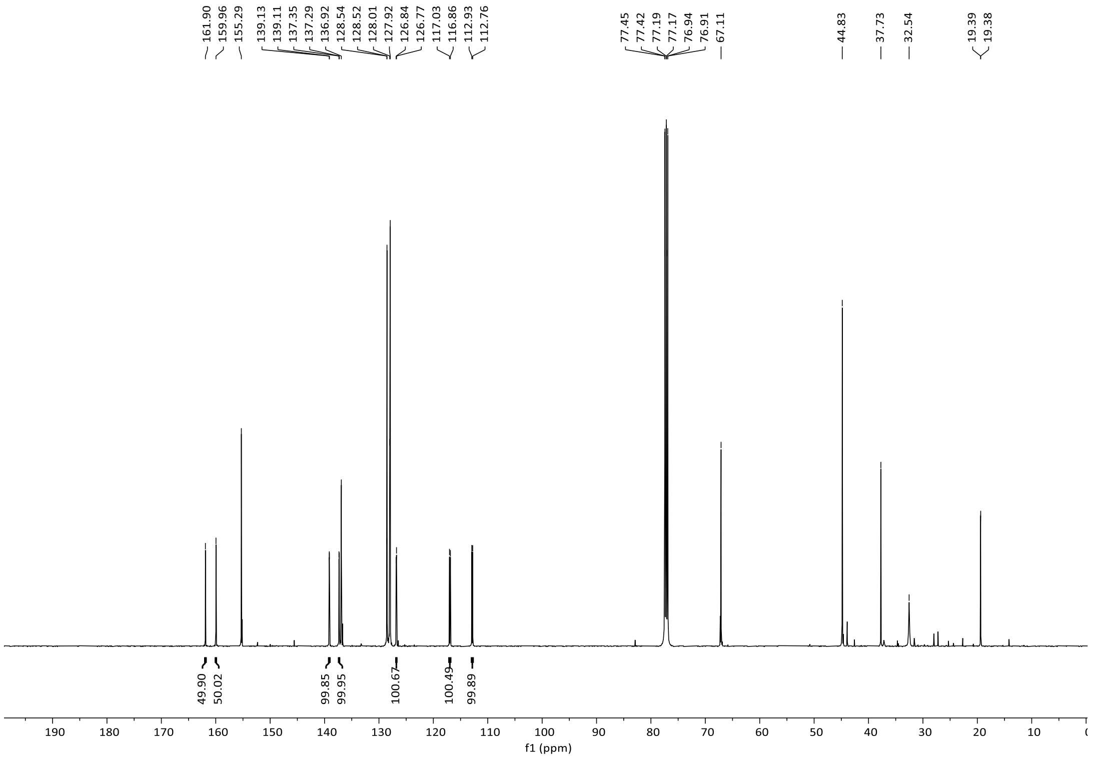

Figure S64. ^{13}C(quant) NMR of sample isolated from full conversion reaction-run2

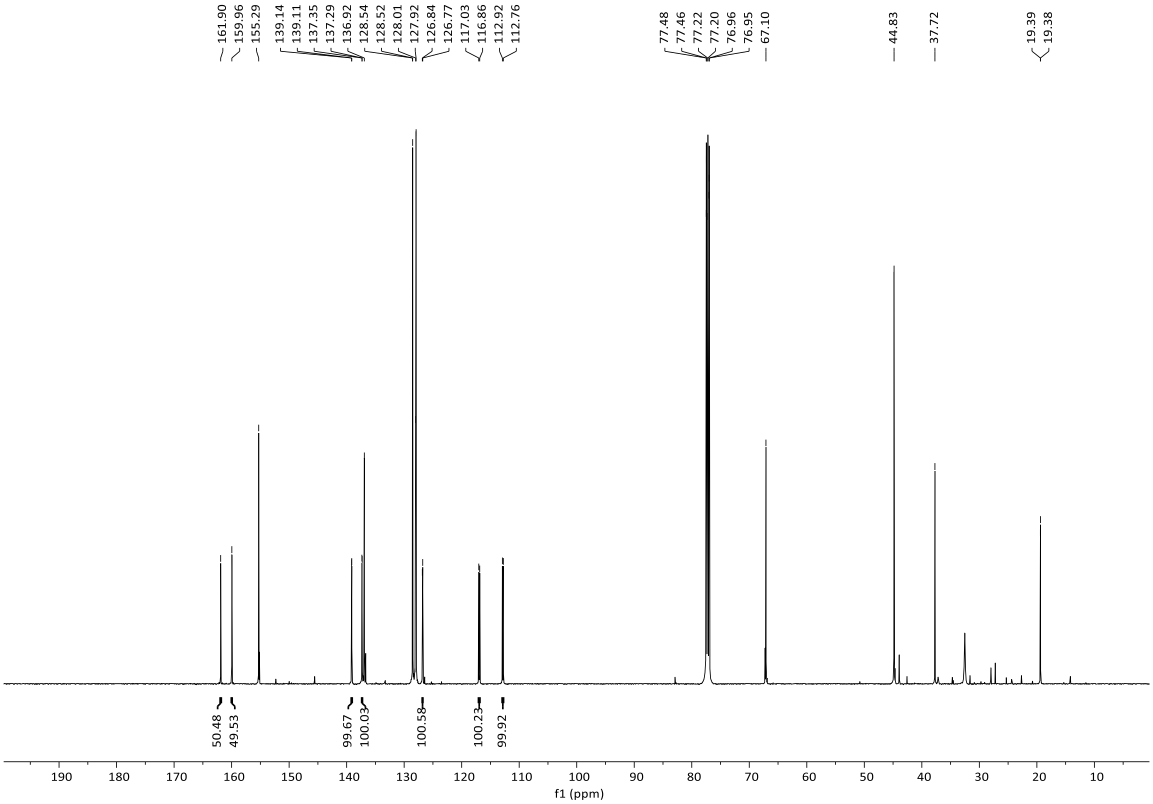

Figure S65. ^{13}C(quant) NMR of sample isolated from full conversion reaction-run3

IV Synthesis and Interception of Ni(III) Complexes

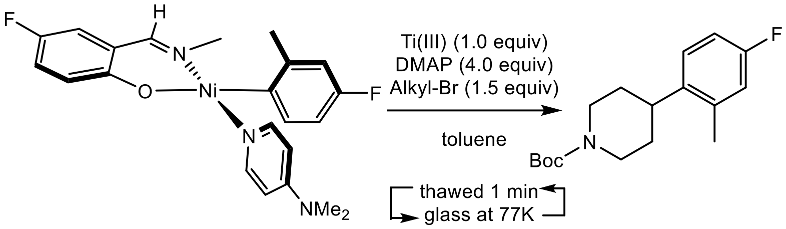

Reaction course monitored by freeze-quenched EPR: In a N_2-filled glovebox, a 20-mL vial was charge with Ti(III)-tris-anilide (6 mg, 0.01 mmol, 1.0 equiv) and 0.5 mL toluene to give a forest green solution. DMAP (5 mg, 0.04 mmol, 4.0 equiv) was added and the solution quickly turned into dark purple. ($Me^{F}FI$)Ni(2-Me-4-F-C_6H_3)(DMAP) (4 mg, 0.01 mmol, 1.0 equiv) was added and the resulting solution was transferred to an EPR tube. The tube top with charge with solids of 4-bromopiperidine-1-carboxylate (~ 10 mg, ~0.4 mmol, ~ 10.0 equiv), capped and brought out of the glovebox horizontally such that the solid did not fall in the reaction solution. The solution was then glassed in liquid nitrogen and brought back to room temperature. The thawing solution was then mixed with alkyl halides, and was then reglassed in liquid nitrogen and analyzed by EPR spectroscopy. The sample was thawed at room temperature for ~1min, reglassed in liquid nitrogen and analyzed be EPR spectroscopy. The process was iterated several times until full decay of EPR signals.

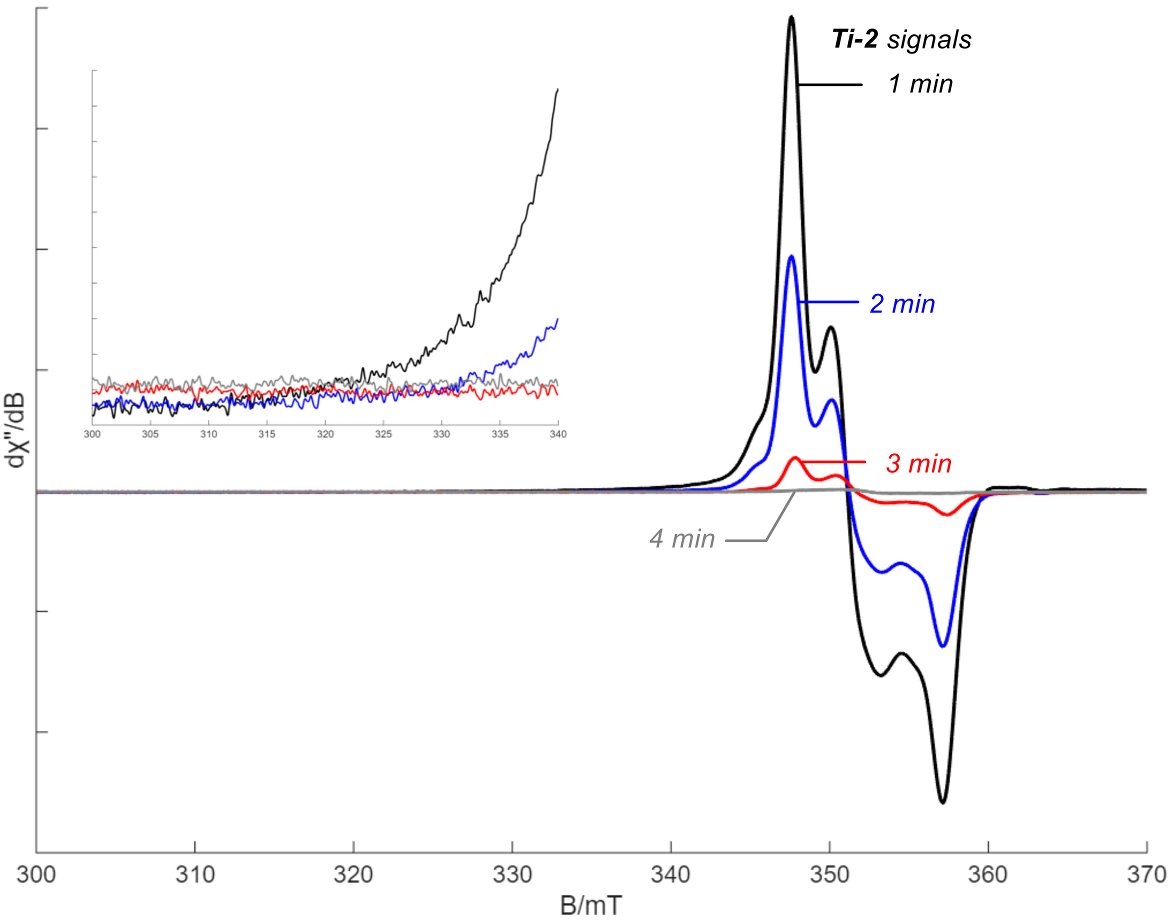

Figure S66. Reaction course monitored by freeze-quenched EPR

EPR spectrum were collected at 77 K in toluene glass. Experimental parameters: microwave frequency = 9.641 GHz, power = 0.3162 mW, and modulation amplitude = 4.000 G.

Simulation parameters:

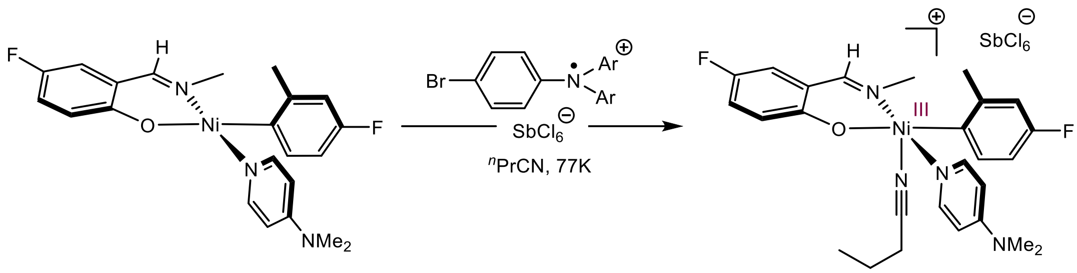

Oxidation with magic blue: In a N_2-filled glovebox, a 20-mL vial was charge with (Me^{F}FI)Ni(2-Me-4-F-C_6H_3)(DMAP) (4 mg, 0.01 mmol, 1.0 equiv) and 0.5 mL butyronitrile. The solution was transferred to an EPR tube. The tube top with charge with solids of tris(4-bromophenyl)ammoniumyl hexachloroantimonate (~ 10 mg, ~0.012 mmol, ~ 1.0 equiv), capped and brought out of the glovebox horizontally such that the solid did not fall in the reaction solution. The solution was then glassed in liquid nitrogen and brought back to room temperature. The thawing solution was then mixed with the oxidant, which quickly turned into a dark red solution, and was then reglassed in liquid nitrogen and analyzed by EPR spectroscopy.

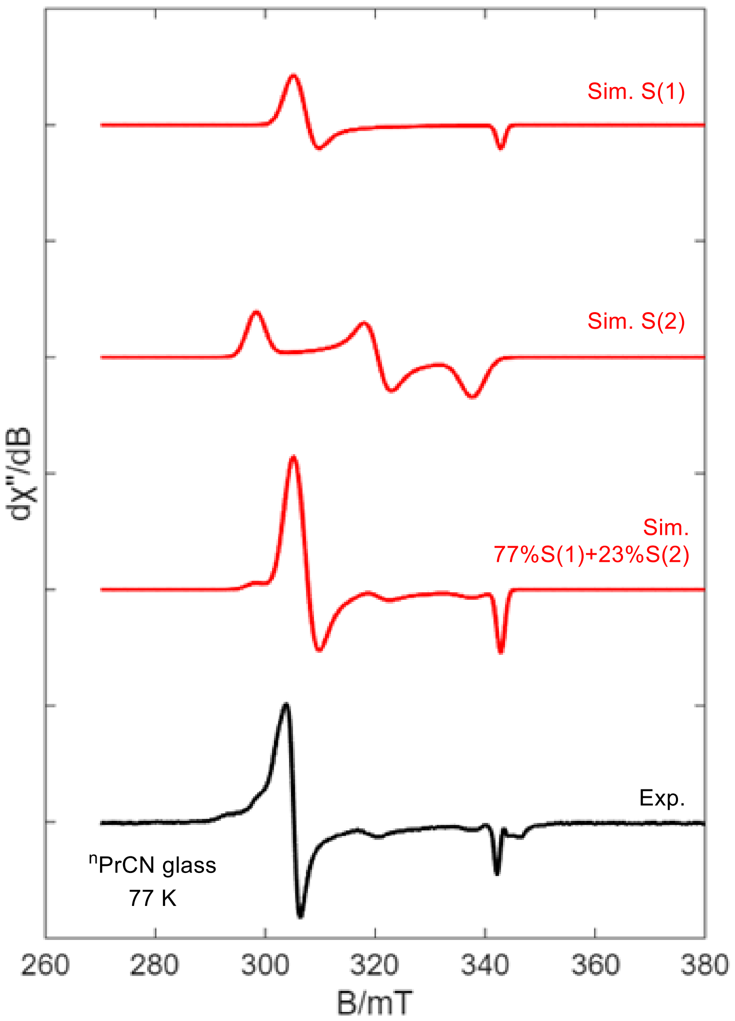

Figure S67. EPR spectrum of oxidation of nickel aryl complex with magic blue.

EPR spectrum were collected at 77 K in butyronitrile glass. Experimental parameters: microwave frequency = 9.644 GHz, power = 0.3162 mW, and modulation amplitude = 4.000 G.

Simulation parameters:

S(1) = 1/2, $g_1(1) = 2.26$, $g_2(1) = 2.24$, $g_3(1) = 2.04$, and $g_{strain}(1) = (0.03, 0.03, 0.01)$;

S(2) = 1/2, $g_1(2) = 2.31$, $g_2(2) = 2.15$, $g_3(2) = 2.04$, and $g_{strain}(2) = (0.03, 0.03, 0.03)$;

S(1) = 77%; S(2) = 23%.

We tentatively assign these signals to two different nickel(III) complexes, including *S*(1): solvated ion pair Ni(III) complex, *S*(2): unsolvated ion pair Ni(III) complex.

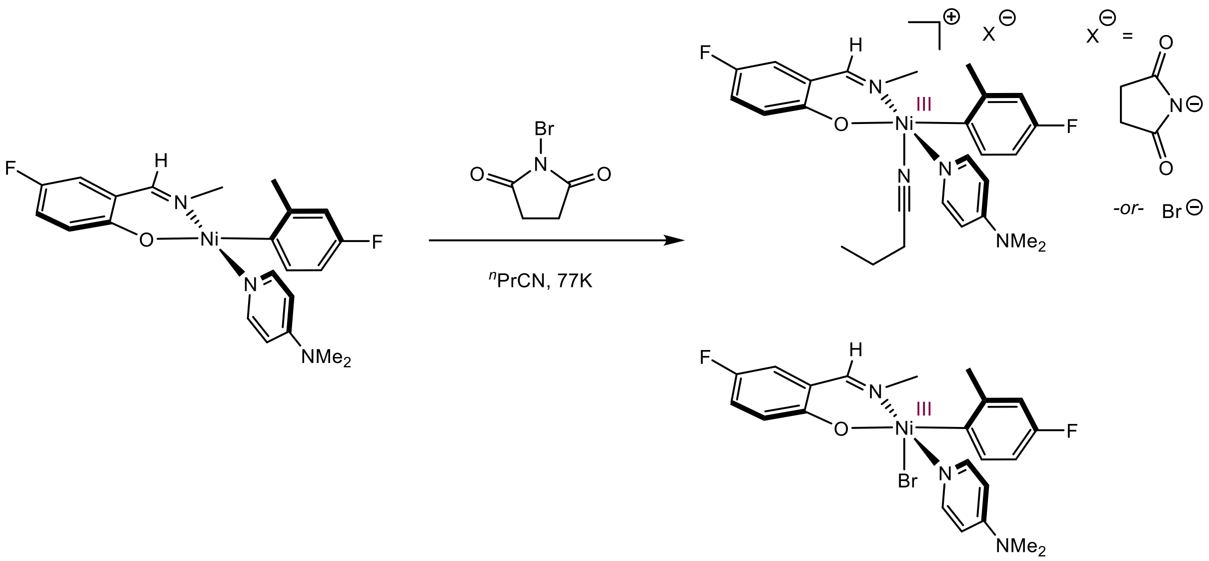

Oxidation with NBS: In a N_2-filled glovebox, a 20-mL vial was charge with (Me^{F}FI)Ni(2-Me-4-F-C_6H_3)(DMAP) (4 mg, 0.01 mmol, 1.0 equiv) and 0.5 mL butyronitrile. The solution was transferred to an EPR tube. The tube top with charge with solids of N-bromosuccinimide (~ 10 mg, ~0.056 mmol, ~ 6 equiv), capped and brought out of the glovebox horizontally such that the solid did not fall in the reaction solution. The solution was then glassed in liquid nitrogen and brought back to room temperature. The thawing solution was then mixed with the oxidant, which quickly turned into a dark red solution, and was then reglassed in liquid nitrogen and analyzed by EPR spectroscopy.

In a separate experiment, the reaction was conducted at 0.1 mmol scale in a 20-mL vial. The reaction was thawed after addition of oxidant for 5 min, the dark red solution faded and turned into an orange-yellow solution. The solution was added 2-F-biphenyl (19 mg) as internal standard, and the reaction mixture was analyzed by ^{19}F NMR. The product was not isolated due to its low boiling point.

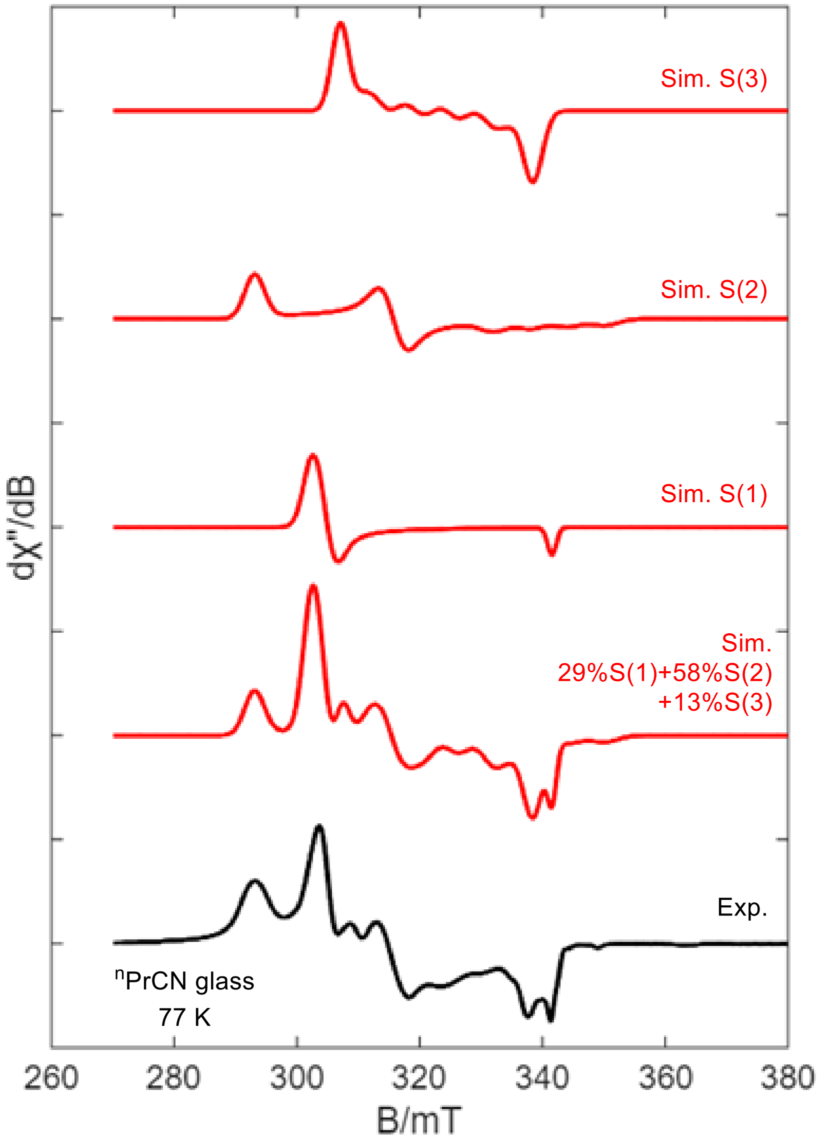

Figure S68. EPR spectrum of oxidation of nickel aryl complex with NBS.

EPR spectrum were collected at 77 K in butyronitrile glass. Experimental parameters: microwave frequency = 9.646 GHz, power = 0.3162 mW, and modulation amplitude = 4.000 G.

Simulation parameters:

S(1) = 1/2, $g_1(1)$ = 2.28, $g_2(1)$ = 2.26, $g_3(1)$ = 2.02, and $g_{strain}(1)$ = (0.03, 0.03, 0.01);

S(2) = 1/2, $g_1(2)$ = 2.35, $g_2(2)$ = 2.18, $g_3(2)$ = 2.02, and $g_{strain}(2)$ = (0.03, 0.03, 0.03);

A(^{79}Br): $A_2(1)$ = 0 MHz, $A_2(2)$ = 0 MHz, $A_2(3)$ = 170 MHz.

S(3) = 1/2, $g_1(3)$ = 2.24, $g_2(3)$ = 2.14, $g_3(3)$ = 2.04, and $g_{strain}(3)$ = (0.03, 0.03, 0.02);

A($Br^{79/81}$): $A_3(1)$ = 0 MHz, $A_3(2)$ = 170 MHz, $A_3(3)$ = 0 MHz.

S(1) = 29%; S(2) = 58%; S(3) =13%.

We tentatively assign these signals to three different nickel(III) complexes, including: *S*(1): solvated ion pair Ni(III) complex, *S*(2): Ni(III)-Br complex isomer 1, *S*(3): Ni(III)-Br complex isomer 2.

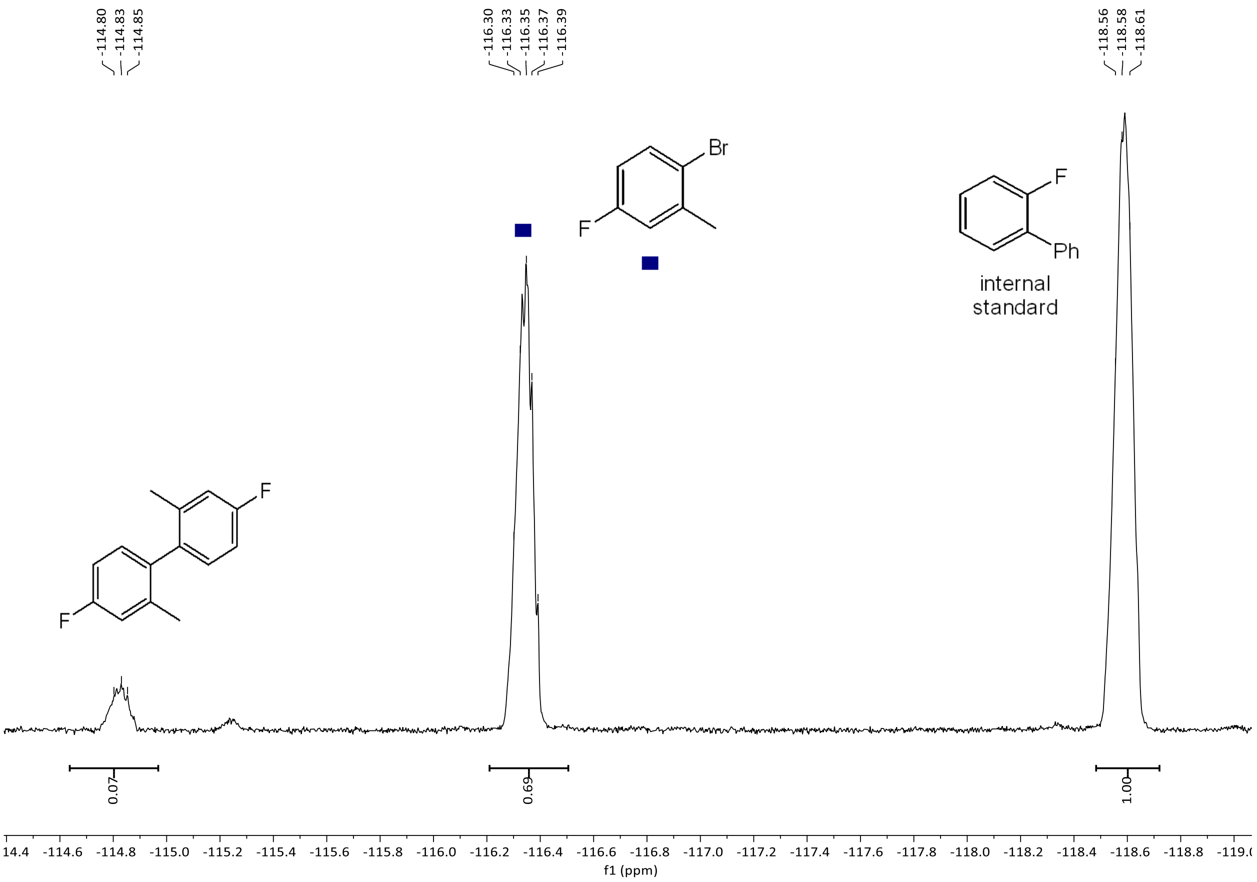

Figure S69. ^{19}F NMR of reaction mixture filtered through a short silica plug.

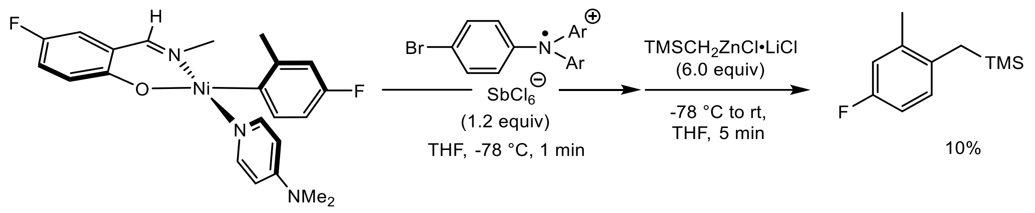

Oxidation with magic blue and intercept with organozinc: In a N_2-filled glovebox, a 20-mL vial was charge with (Me^{F}FI)Ni(2-Me-4-F-C_6H_3)(DMAP) (44 mg, 0.1 mmol, 1.0 equiv) and 1.0 mL THF. The reaction mixture was cooled to -78 ˚C and a solution of tris(4-bromophenyl)ammoniumyl hexachloroantimonate (98 mg, 0.12 mmol, 1.2 equiv) was added. The solution quickly turned to dark red color. After 1 min, a solution of $TMSCH_2ZnCl{\cdot}LiCl$ (prepared by $TMSCH_2Li$ and $ZnCl_2$, 0.6 mmol, 6.0 equiv) was added. The dark red color quickly faded to give an orange-yellow solution. The reaction mixture was allowed to warm to room temperature for 5 min, which was then quenched with HCl (2.0 M, aq.) and extracted with Et_2O. The organic layers were combined and concentrated under reduced pressure, added 5 µL of C_6F_6 and was further analyzed by ^{19}F NMR (10% yield).

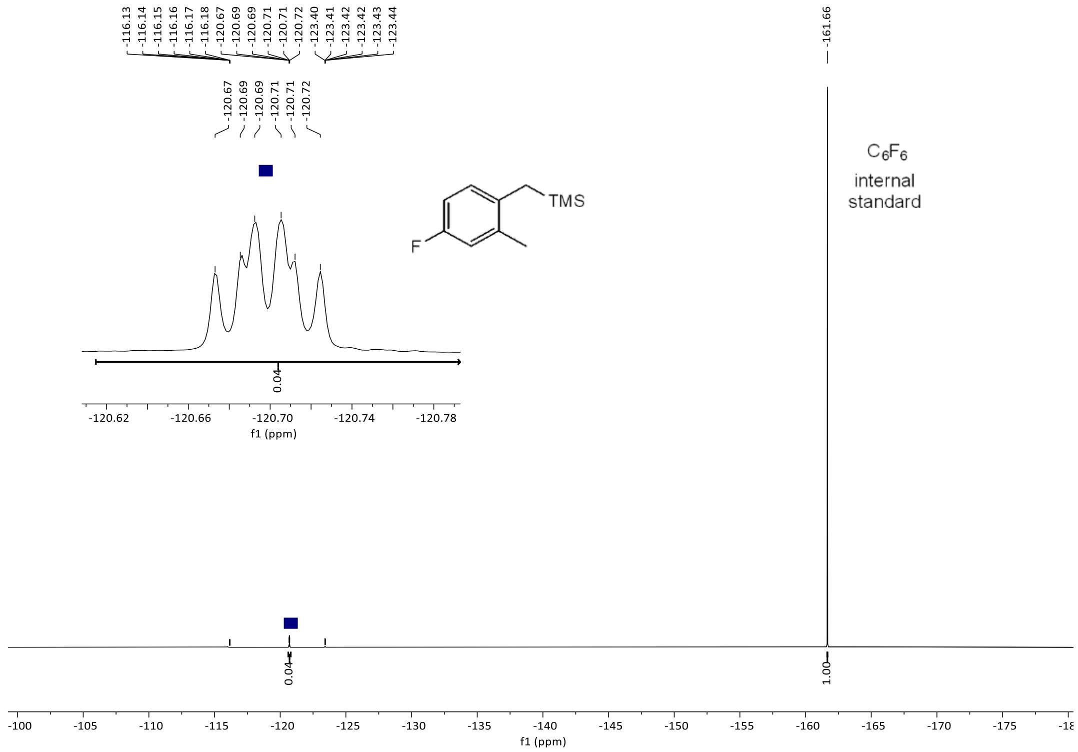

Figure S70. ^{19}F NMR of crude reaction mixture for Ni(III) complex interception.

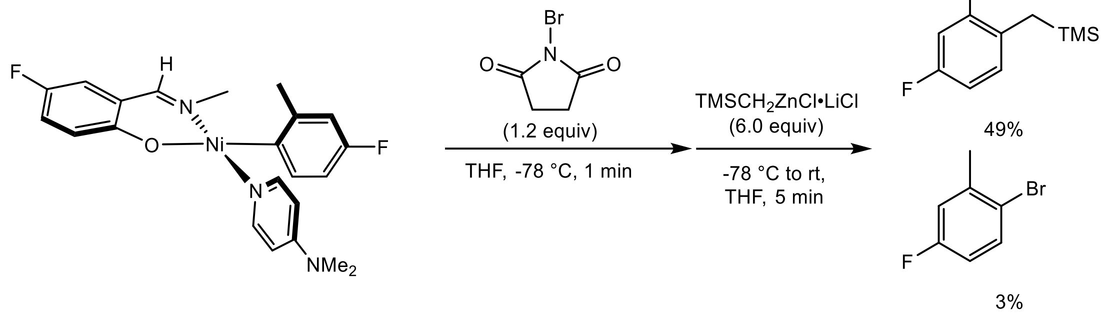

Oxidation with NBS and intercept with organozinc: In a N_2-filled glovebox, a 20-mL vial was charge with (Me^{F}FI)Ni(2-Me-4-F-C_6H_3)(DMAP) (44 mg, 0.1 mmol, 1.0 equiv) and 1.0 mL THF. The reaction mixture was cooled to -78 ˚C and a solution of *N*-bromosuccinimide (21 mg, 0.12 mmol, 1.2 equiv) was added. The solution was quickly turned to dark red color. After 1 min, a solution of $TMSCH_2ZnCl{\cdot}LiCl$ (prepared by $TMSCH_2Li$ and $ZnCl_2$, 0.6 mmol, 6.0 equiv) was added. The dark red color quickly faded to give an orange-yellow solution. The reaction mixture was allowed to warm to room temperature for 5 min, which was then quenched with HCl (2.0 M, aq.) and extracted with Et_2O. The organic layers were combined and concentrated under reduced pressure and was further analyzed by ^{1}H NMR (49% yield, and 3% yield of Ar-Br). The organic product was further purified by column chromatography using pentane as eluent and afford the product as a colorless oil (7 mg, 36% yield). **^{1}H NMR** (500 MHz, $CDCl_3$, 25 ºC): δ 6.88 (dd, *J* = 8.4, 5.9 Hz, 1H), 6.82 (dd, *J* = 9.9, 2.8 Hz, 1H), 6.76 (td, *J* = 8.5, 2.8 Hz, 1H), 2.21 (s, 3H), 2.04 (s, 2H), -0.00 (s, 9H). **^{19}F NMR** (471 MHz, $CDCl_3$, 25 ºC): δ -120.82 (td, *J* = 9.1, 5.9 Hz). **^{13}C NMR** (126 MHz, $CDCl_3$, 25 ºC): δ 160.1 (d, *J* = 240.9 Hz), 136.5 (d, *J* = 7.1 Hz), 134.5 (d, *J* = 3.0 Hz), 129.7 (d, *J* = 7.8 Hz), 116.6 (d, *J* = 20.7 Hz), 112.4 (d, *J* = 20.7 Hz), 22.9, 20.6 (d, *J* = 1.6 Hz), -1.3. **HRMS** *m/z* (EI^+): calcd for $C_{11}H_{17}SiF^+$ (M^+): 196.1078; found: 196.1085.

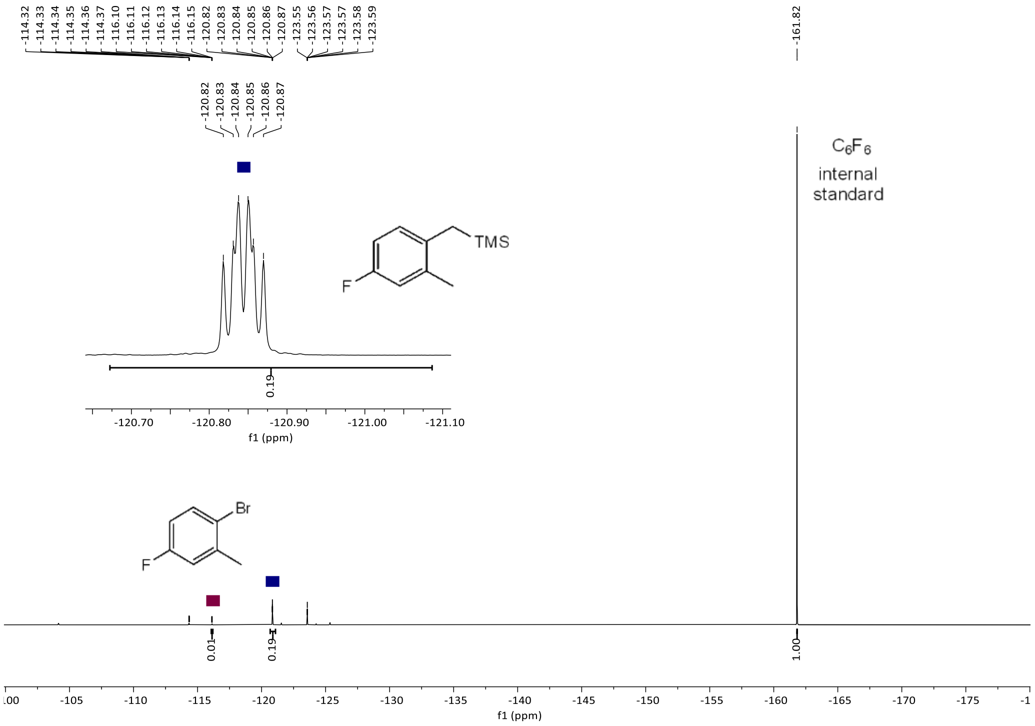

Figure S71. ^{19}F NMR of crude reaction mixture for Ni(III)-Br complex interception.

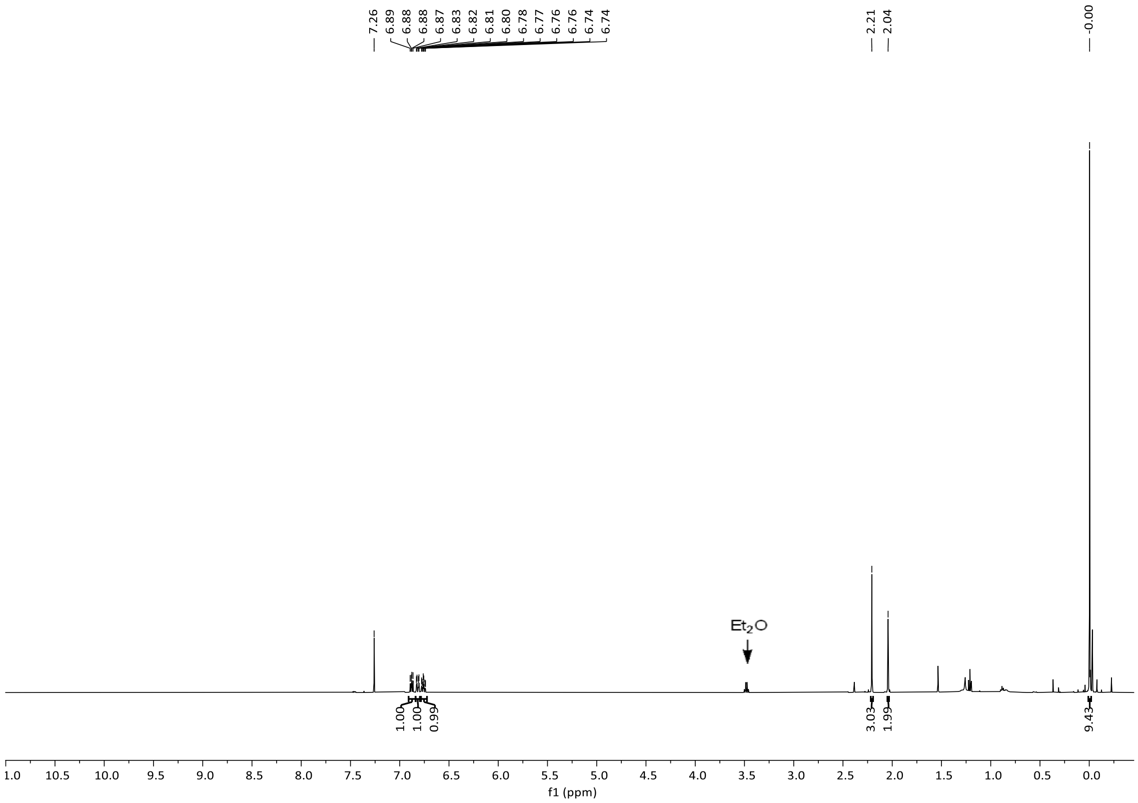

Figure S72. ^{1}H NMR spectrum (500 MHz, $CDCl_3$) of (4-fluoro-2-methylbenzyl)trimethylsilane.

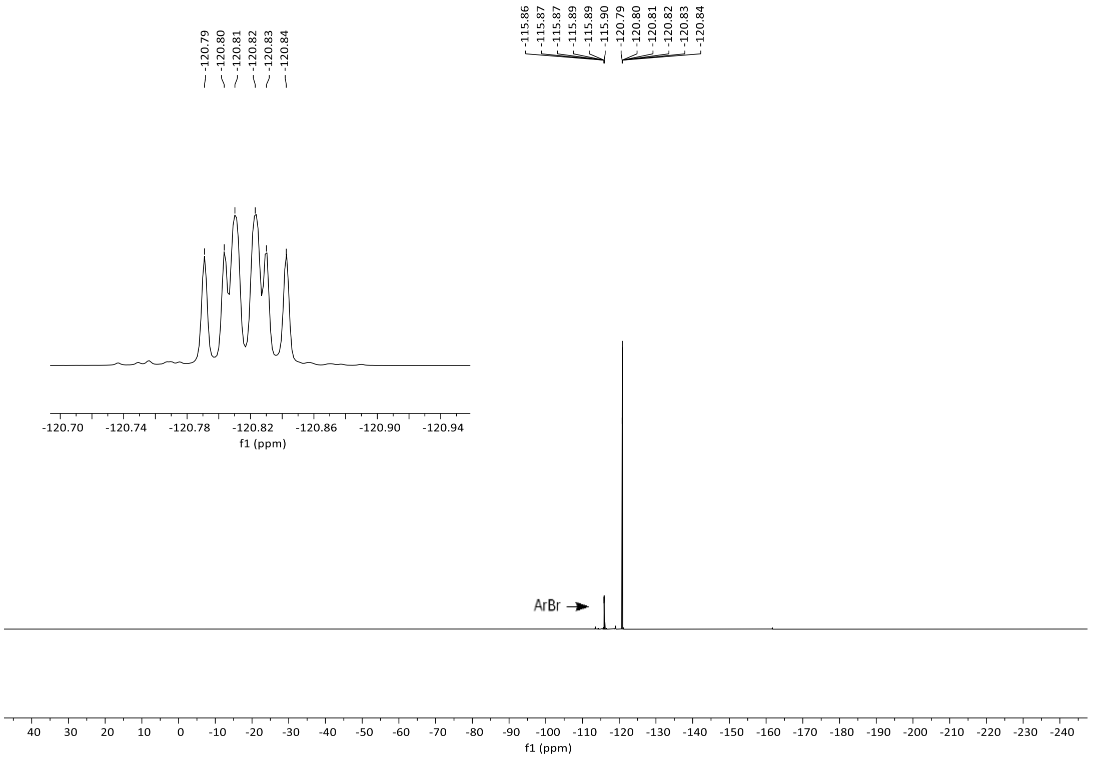

Figure S73. ^{19}F NMR spectrum (471 MHz, $CDCl_3$) of (4-fluoro-2-methylbenzyl)trimethylsilane.

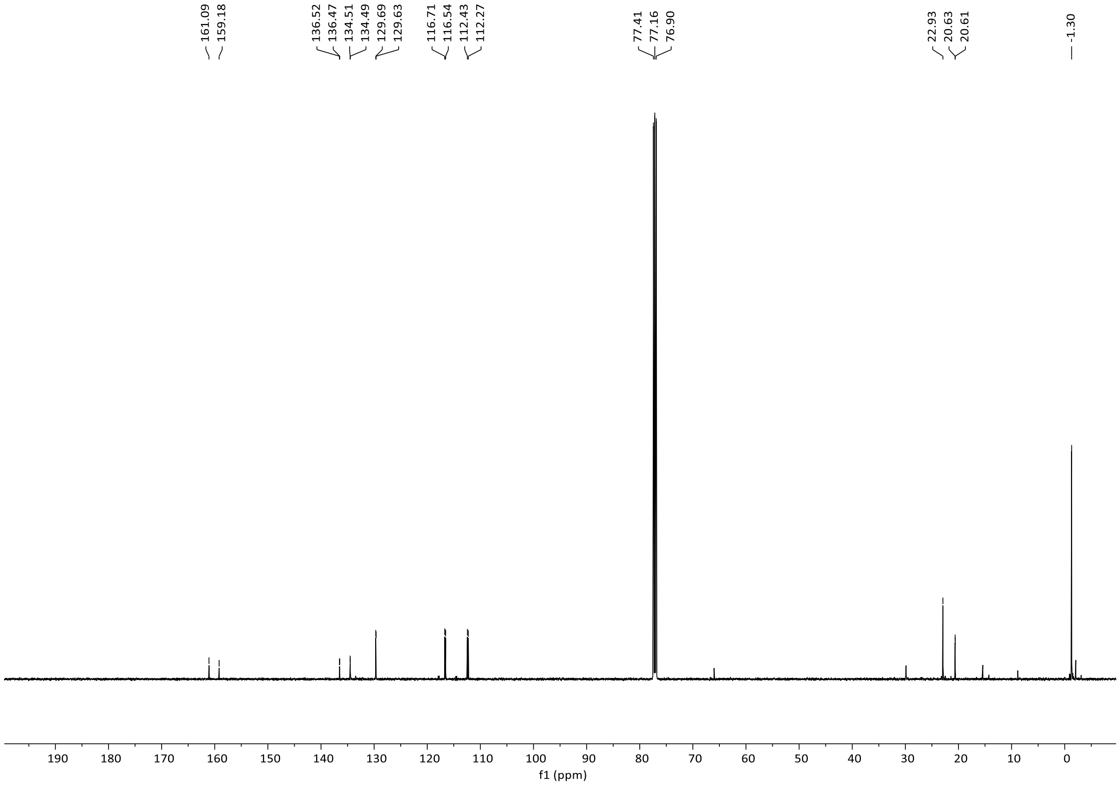

Figure S74. ^{13}C NMR spectrum (126 MHz, $CDCl_3$) of (4-fluoro-2-methylbenzyl)trimethylsilane.

Addition of organozinc to a thawing, dark red solution of Ni(III)-Br led to instant color change to yellow. Rapidly refrozing the sample and retaking EPR suggested the Ni(III) complexes were already fully consumed. This is likely due to the short lifetime of a Ni(III)-aryl-alkyl complex.

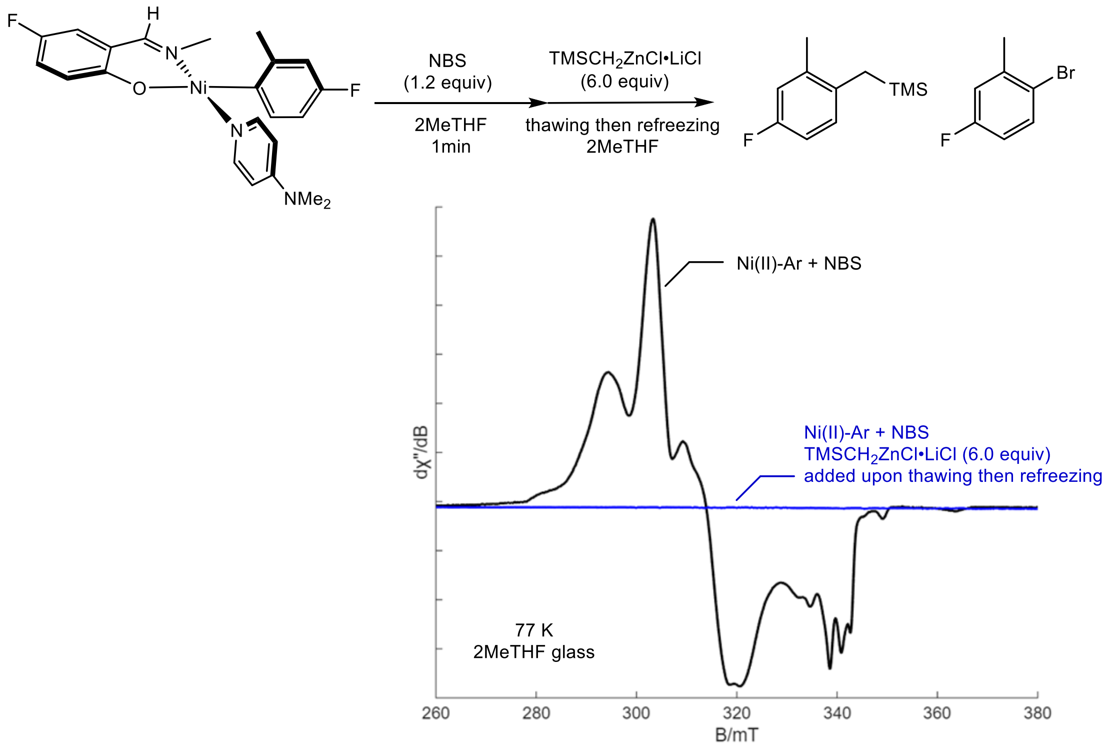

Figure S75. Reaction progress monitored by freeze-quenched EPR

Reaction between Ni(II)-aryl alkyl complex was examined by reaction of Ni-aryl complex and 2.0 equivalence of neosilyl-Zn, neosilyl-Li reagent in THF/C_6D_6. No product formation (<2%) was observed, rendering C(sp^2)-C(sp^3) bond formation from Ni(II)-alkyl-aryl reductive elimination unlikely.

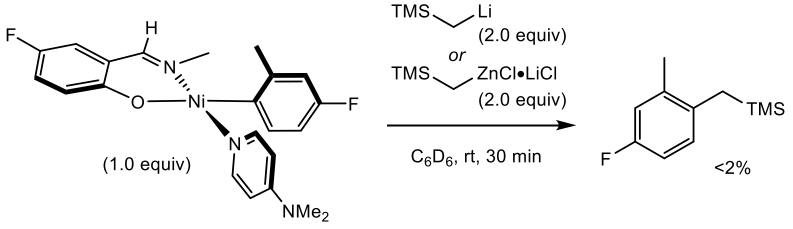

X. Computational Data

Consideration of other functionals

Sevearl functionals (including M06(D3), PBE0(D3), TPSSh(D4)) were used for energy surface calculations. These results provide similar conclusions. TPSSh(D4) was used for all other calculations.

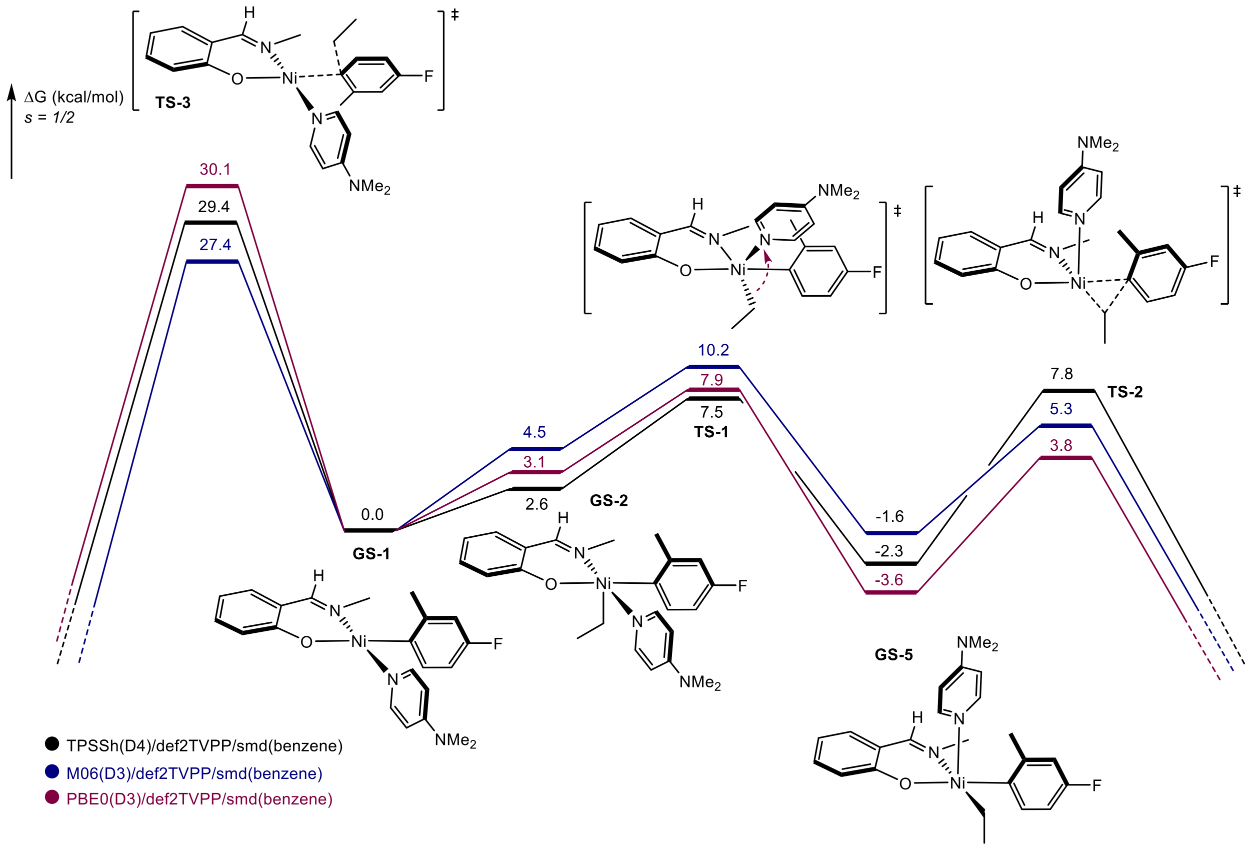

Figure S76. DFT calculation of energy surface with different functional.

Consideration of other isomerization pathways

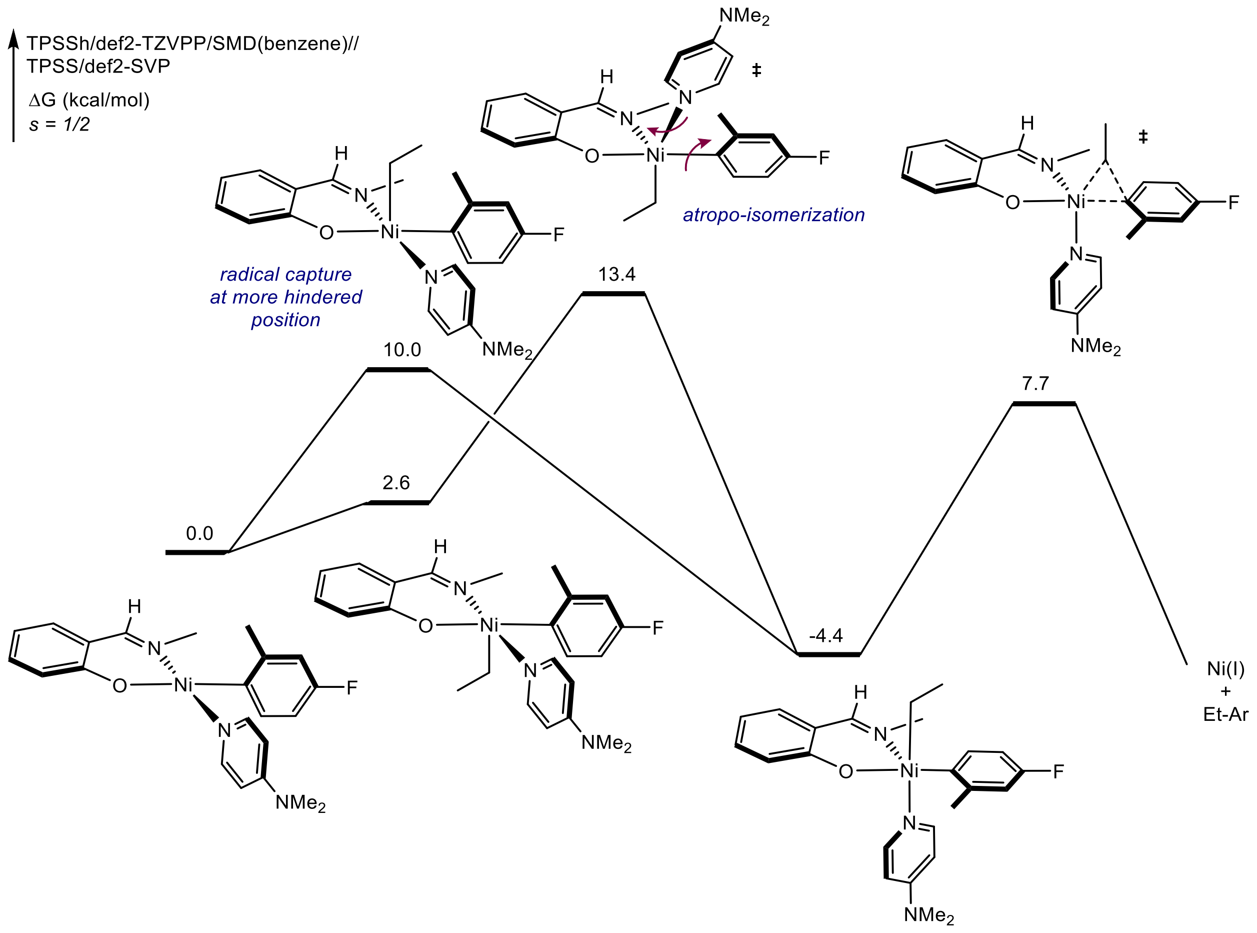

Figure S77. Consideration of other possible isomerization pathways before reductive elimination.

Several other isomerization pathways are also considered, which are higher in energy than the pseudo rotation pathway.

Energy surface with bulky substitution at the ligand imine position

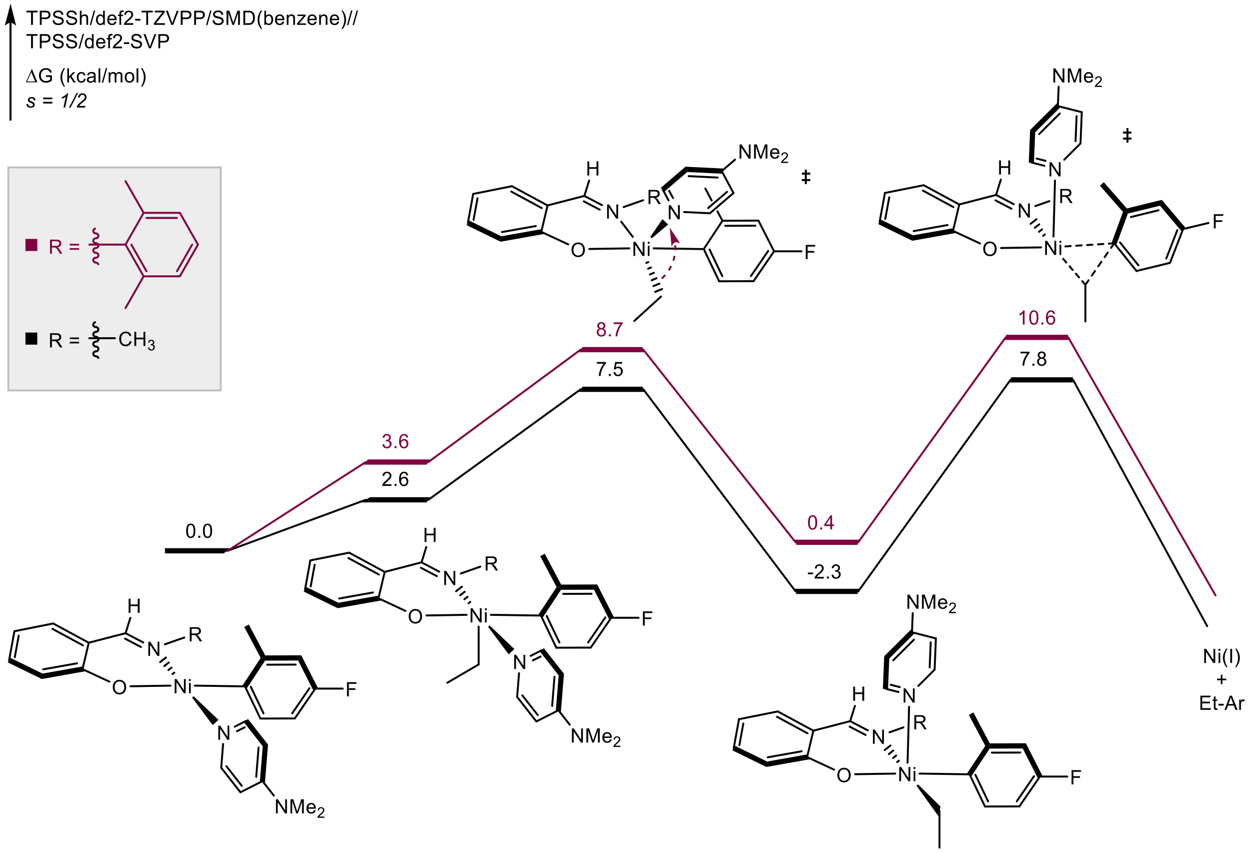

Figure S78. Impact of steric from phenoxy(imine) ligand on energy surface.

Radical capture of FI-Ni(II)-Ar complex with 2,6-dimethylaniline substitution at the imine position was computed. An overall higher-barrier energy surface was observed. In particular, the pseudo rotation barrier was 1.2 kcal higher in energy.

Energy surface with electron rich FI ligands

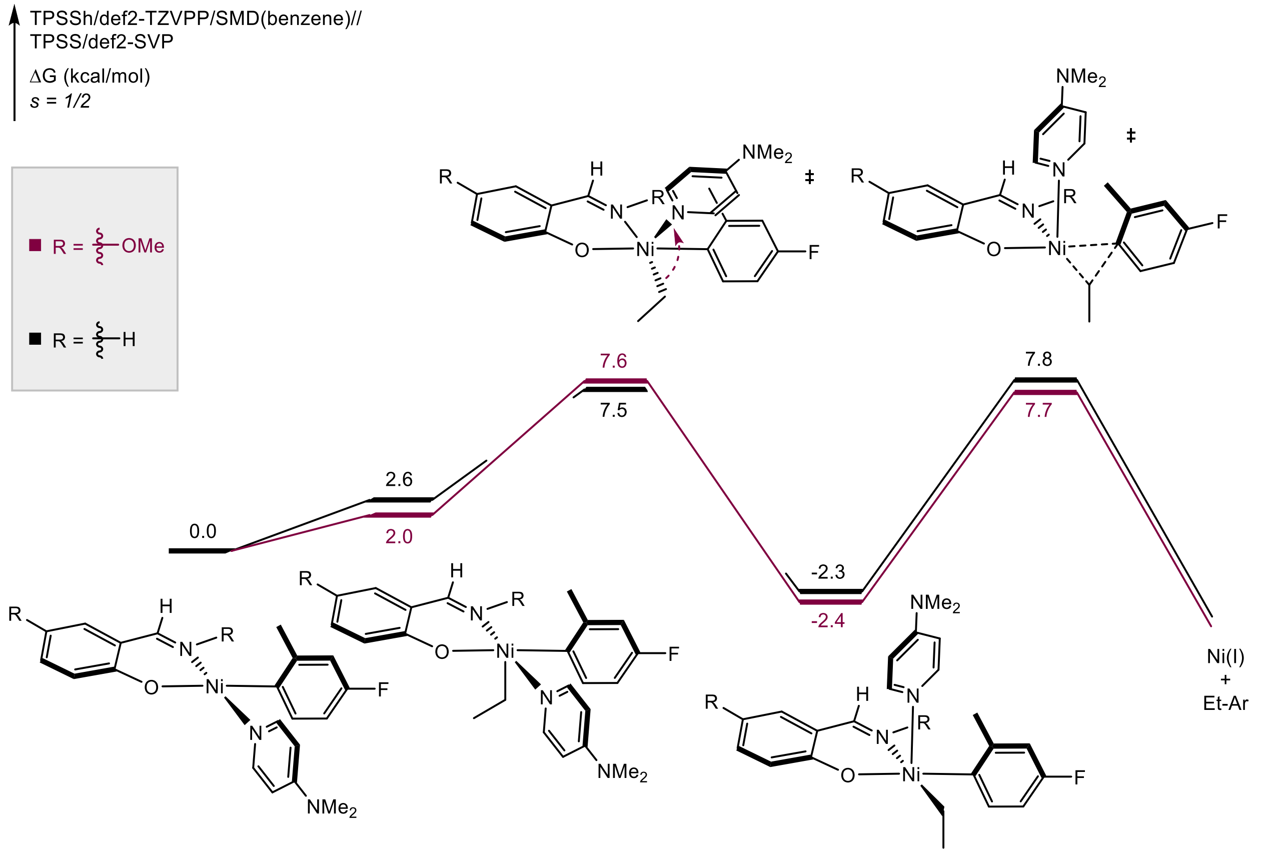

Figure S79. Impact of electronics from phenoxy(imine) ligand on energy surface.

Radical capture of FI-Ni-Ar complex with 4-methoxy substitution at the FI ligand was computed. An overall similar energy barrier was observed. Energy barrier changes are subtle.

Energy surface with electron rich aryl group on nickel

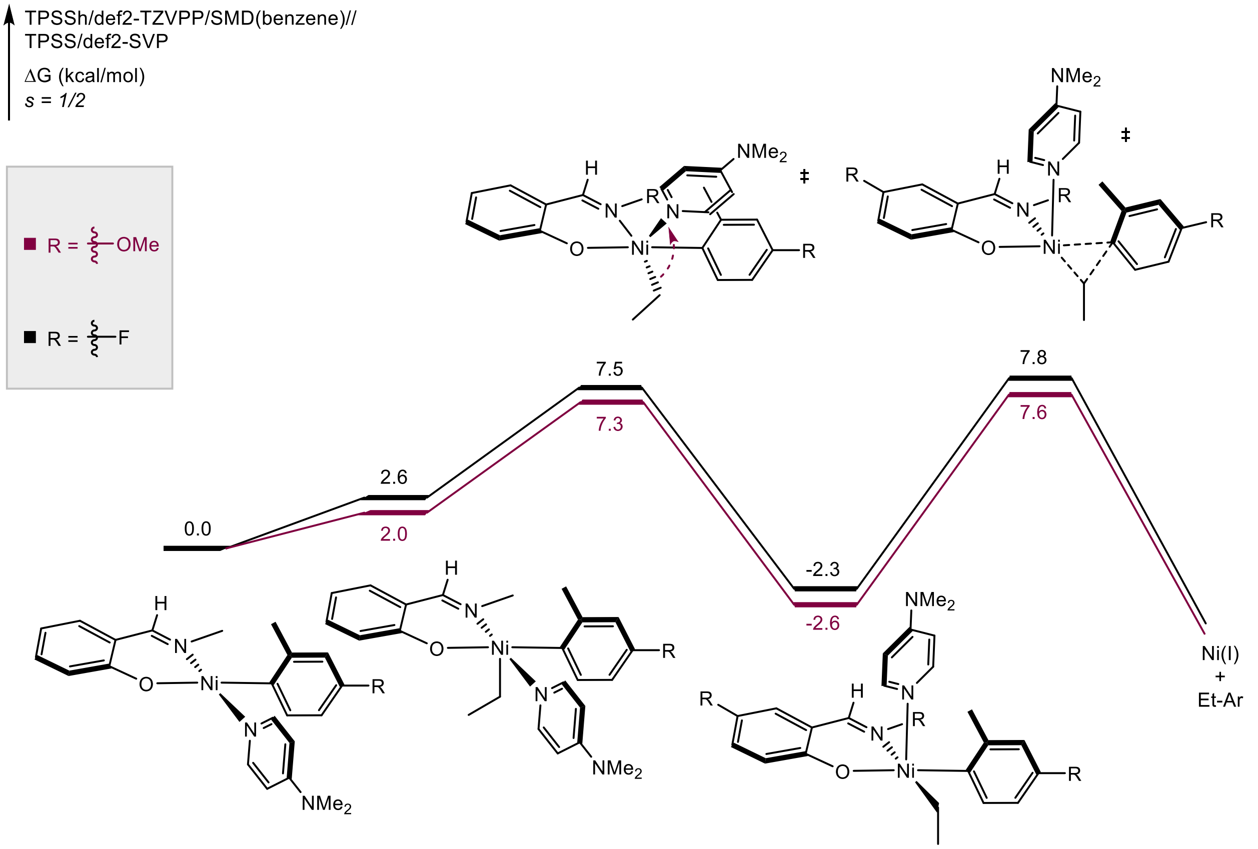

Figure S80. Impact of electronics from aryl group on energy surface.

Radical capture of FI-Ni-Ar complex with 4-methoxy substitution at the Ni-aryl group was computed. An overall lower energy barrier was observed.

Computation of KIE

KIE calculations were conducted with frequency calculations with the adjusted mass. (^{13}C = 13.003354835). For single step reaction, KIE was calculated using the following equation.

$$k_{13C}/k_{12C} = e^{-(\Delta G^{\ddagger}_{13C} - \Delta G^{\ddagger}_{12c})/RT}$$

For multi-step process, the overall product distribution was calculated using COPASI simulation.

KIE of single step reaction

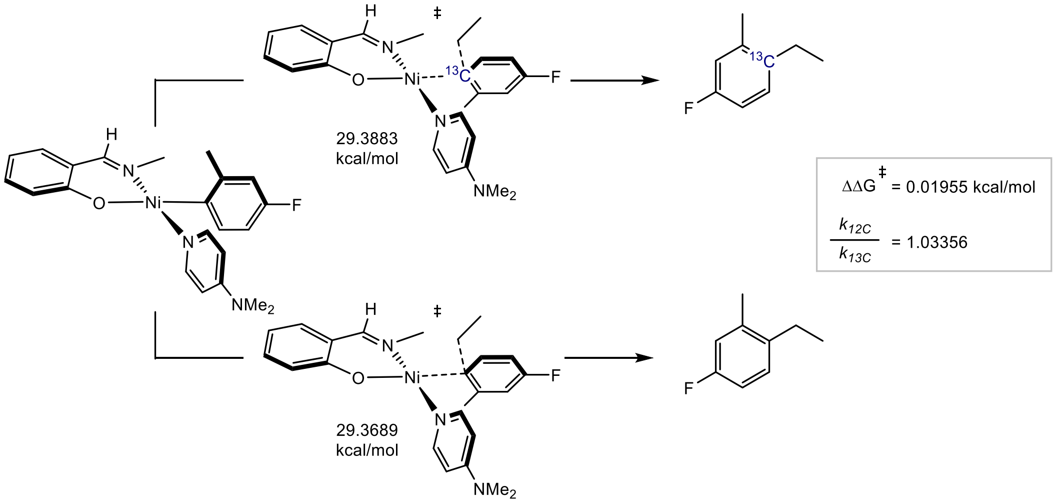

Figure S81. Kinetic isotope effect for a concerted C-C bond formation predicted by DFT calculations.

KIE of multi-step reaction

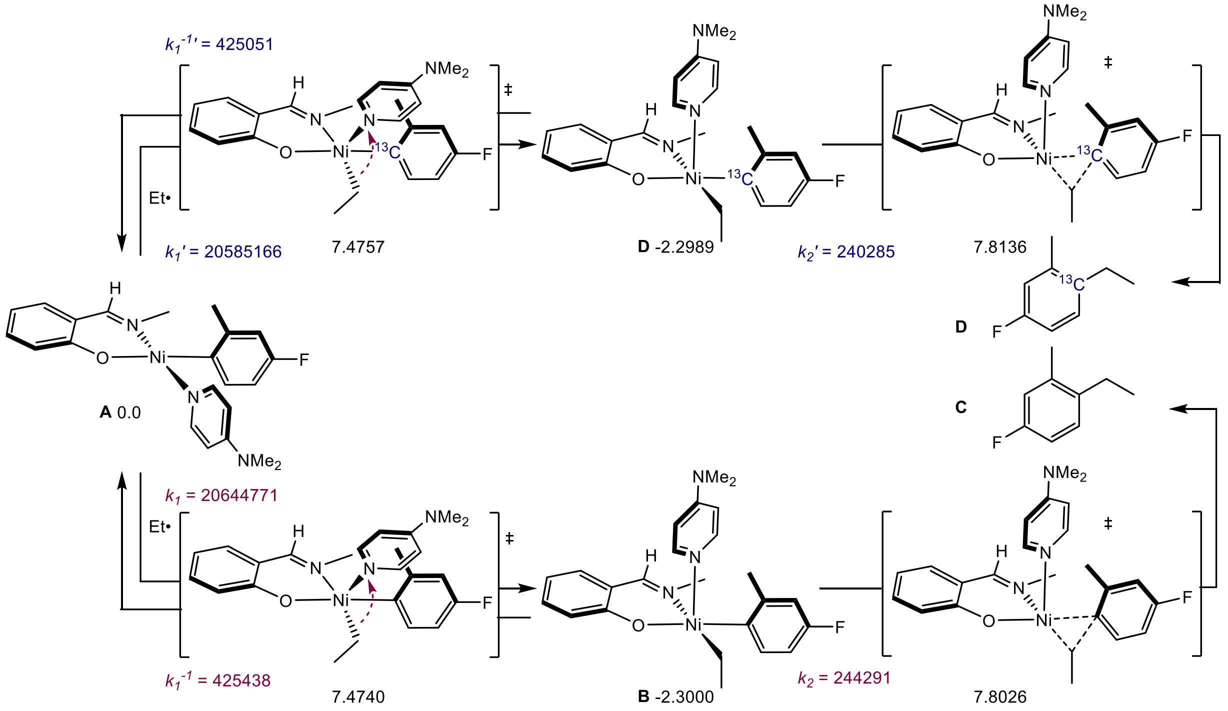

KIE of pseudo rotation

$\Delta\Delta G^{\ddagger}$ = 0.00171 kcal/mol

$\frac{k_{12C}}{k_{13C}}$ = 1.0029

KIE of reductive elimination

$\Delta\Delta G^{\ddagger}$ = 0.01097 kcal/mol

$\frac{k_{12C}}{k_{13C}}$ = 1.0187

Overall product distribution

$\frac{C}{E}$ = 1.0129

COPASI simulated time course in 50 μs

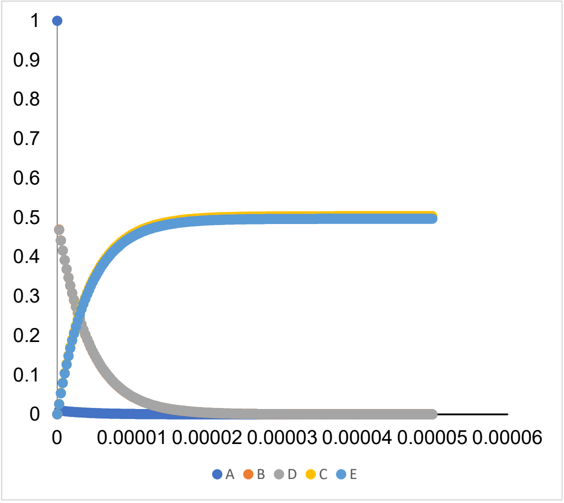

Figure S82. Kinetic isotope effect for multi-step C-C bond formation predicted by DFT calculations and COPASI simulations.

COPASI simulation was done with deterministic (LSODA) method with duration of 5x10^{-5} seconds with 200 intervals.

Coordinates and Energies

Table S6. Summary of computational energies (Hartree)

structure	spin state	Gibbs (geo. opt.)	E (singlet point)	G-E_{el}(geo. opt.)	Final Gibbs
Ethyl radical	1/2	-79.085459	-79.20032824	0.0343533	-79.166
DMAP	0	-381.95145	-382.4463744	0.12722718	-382.319
**GS-1**	0	-2699.4668	-2698.902521	0.35888919	-2698.54
**GS-2**	1/2	-2778.5583	-2778.123903	0.41843715	-2777.71
**GS-3**	1/2	-3160.5085	-3160.583786	0.57149553	-3160.01
**GS-4**	3/2	-2778.5212	-2778.086318	0.41240278	-2777.67
**GS-5**	1/2	-2778.5624	-2778.132108	0.41883626	-2777.71
**GS-6**	1/2	-2778.6036	-2778.176547	0.42036768	-2777.76
**GS-7**	1/2	-2778.6006	-2778.174678	0.41950006	-2777.76
**TS-1**	1/2	-2778.5498	-2778.117396	0.41969993	-2777.7
**TS-2**	1/2	-2778.5456	-2778.114413	0.41724103	-2777.7
**TS-3**	1/2	-2778.5119	-2778.078936	0.41613133	-2777.66

Sample Geometry Optimization Input File

```
!UKS Opt TPSS D3BJ def2-SVP TightSCF UNO NumFreq

%MaxCore 8000

%SCF
MaxIter 5000
end

%pal nprocs 16 end

*xyz 1 2
[coordinates]
*
```

Sample Single Point Calculation Input File

```
!UKS SP TPSSh D4 RIJCOSX UNO UCO DFK-def2/J def2-TZVPP/C TightSCF
!NoAutoStart Normalprint CPCM

%SCF
MaxIter 5000
end

%cpcm
smd true
SMDsolvent “benzene”
End

*xyzfile 0 2 [filename].xyz

%pal nprocs 16 end
%maxcore 2500

%output
Print[P_Basis] 2
Print[P_MOs] 1
end
```

XI. Kinetic Model for Multistep Radical Coupling

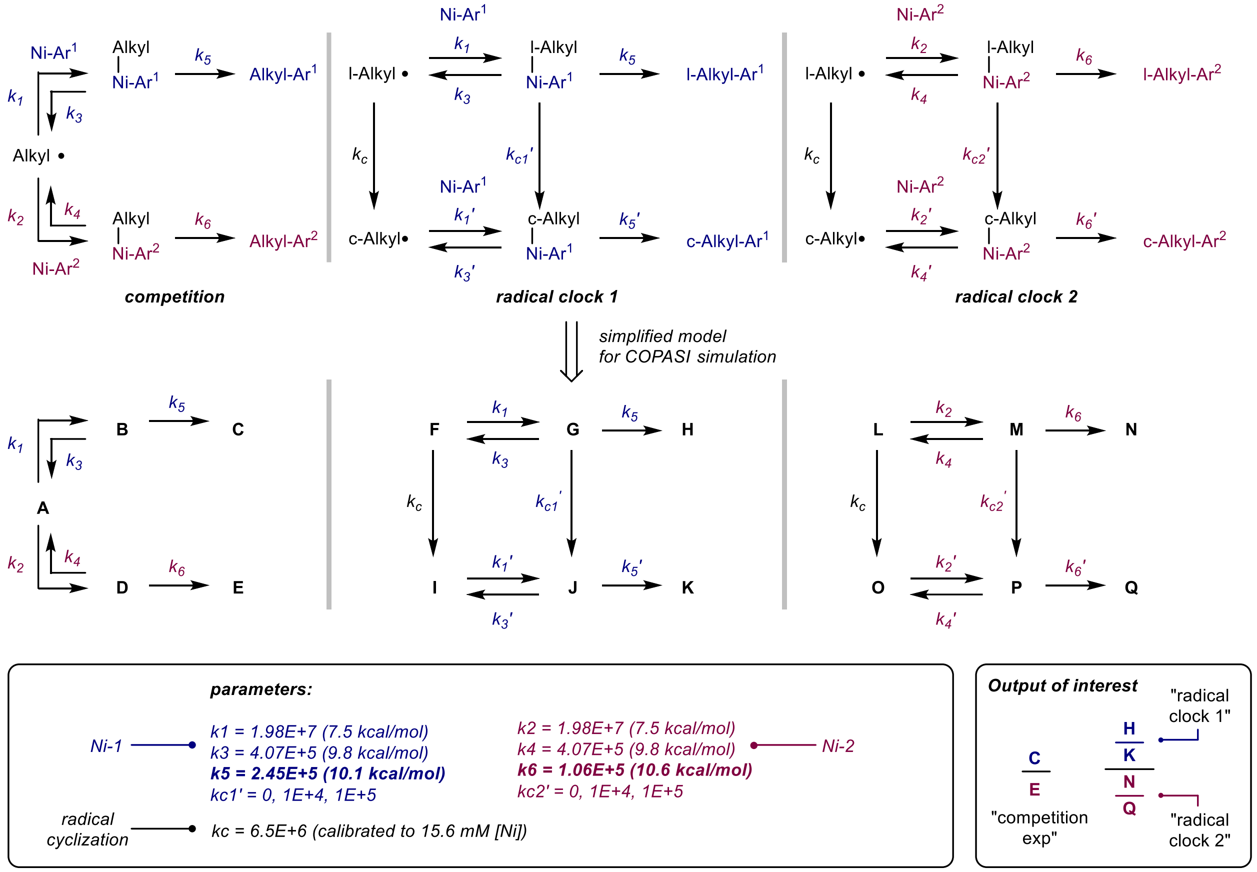

	kc1 = kc2 = 0	kc1 = kc2 = 1E+4	kc1 = kc2 = 1E+5
C / E	1.82	1.82	1.82
(H / K) / (N / Q)	1.80	1.83	1.99

Figure S83. Kinetic model for multi-step C-C bond formation and COPASI simulations.

A kinetic model involving radical capture with Ni(II)-Ar complex at nickel center followed by reductive elimination was proposed. For formation of linear or cyclic product for radical clock 1 and radical clock 2 paradigm, a “in cage” radical cyclization (*kc1’* and *kc2’*) was considered. In this pathway, the linear to cyclic isomerization does not involve full dissociation of alkyl group from Ni(III) intermediate and thus should have a different kinetic behaviour than 5-exo-tric cyclization of a free hexenyl radical.

The model was simplified and processed on COPASI software for demonstration of the complex kinetic picture. **A**, **F** or **G** are considered as generated radicals, which go to full conversion to corresponding products (**C**, **E**, **H**, **K**, **N**, **Q**). Due to pseudo first order kinetics, all reactions are simplified to first order, and the free radical cyclization was normalized with respect to a specific nickel concentration. For simplification. linear and cyclic radical kinetics were considered equal (e.g. $k_1 = k_1'$). Energy parameters similar to DFT suggested barriers were used as input, in which reductive elimination barrier of two nickel complex was set to have a 0.5 kcal/mol difference.

COPASI simulation was computed with full conversion of **A**, **F** and **G** (>99% conv.) and product ratios **C**/**E**, (**H**/**K**)/(**N**/**Q**) were analyzed. Simulated results suggest complex results impacted by multiple parameters, and **C**/**E** and (**H**/**K**)/(**N**/**Q**) are non-equivalent. While real kinetic scenario is even more complex and this simulation cannot represent all the experimentally observed data, these preliminary results suggest that competition reaction product distribution (**C**/**E**) and ratios determined by independent radical clock measurements ((**H**/**K**)/(**N**/**Q**)) are different under a multistep radical coupling paradigm.

XII. Crystallographic Data

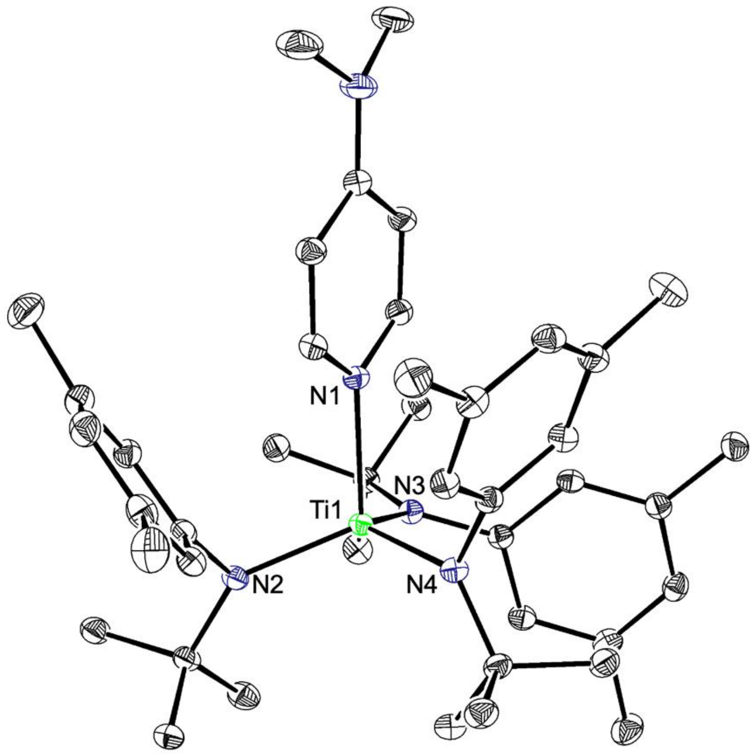

Figure S84. Representation of the solid-state structure of **Ti-2** (CCDC 2520870). Hydrogen atoms omitted for clarity. Ellipsoids presented with 30% probabilities.

Table S7. Crystal data for **Ti-2**.

Crystal data

$C_{43}H_{64}N_5Ti$	$F(000) = 1516$
$M_r = 698.89$	$D_x = 1.118$ Mg m^{-3}
Monoclinic, $P2_1/n$	Cu $K\alpha$ radiation, $\lambda = 1.54184$ Å
$a = 9.7423$ (1) Å	Cell parameters from 32127 reflections
$b = 24.3303$ (3) Å	$\theta = 3.6–75.2°$
$c = 17.6426$ (2) Å	$\mu = 1.99$ mm^{-1}
$\beta = 96.880$ (1)°	$T = 100$ K
$V = 4151.77$ (8) Å^3	Prism, metallic dark brown
$Z = 4$	0.30 × 0.14 × 0.09 mm

Data collection

XtaLAB Synergy, Dualflex, HyPix-Arc 150 diffractometer	8414 independent reflections
Radiation source: micro-focus sealed X-ray tube, PhotonJet (Cu) X-ray Source	7056 reflections with $I > 2\sigma(I)$
Mirror monochromator	R_{int} = 0.051
Detector resolution: 10.0000 pixels mm^{-1}	θ_{max} = 75.8°, θ_{min} = 3.1°
ω scans	h = -11∅12
Absorption correction: multi-scan *CrysAlis PRO* 1.171.43.143a (Rigaku Oxford Diffraction, 2024) Empirical absorption correction using spherical harmonics, implemented in SCALE3 ABSPACK scaling algorithm.	k = -30∅29
T_{min} = 0.722, T_{max} = 1.000	l = -20∅22
79812 measured reflections	

Refinement

Refinement on F^2	Primary atom site location: dual
Least-squares matrix: full	Hydrogen site location: inferred from neighbouring sites
$R[F^2 > 2\sigma(F^2)]$ = 0.042	H-atom parameters constrained
$wR(F^2)$ = 0.122	$w = 1/[\sigma^2(F_o^2) + (0.0686P)^2 + 1.3809P]$ where $P = (F_o^2 + 2F_c^2)/3$
S = 1.05	$(\Delta/\sigma)_{max}$ = 0.001
8414 reflections	$\Delta\rangle_{max}$ = 0.34 e Å^{-3}
459 parameters	$\Delta\rangle_{min}$ = -0.62 e Å^{-3}
0 restraints	

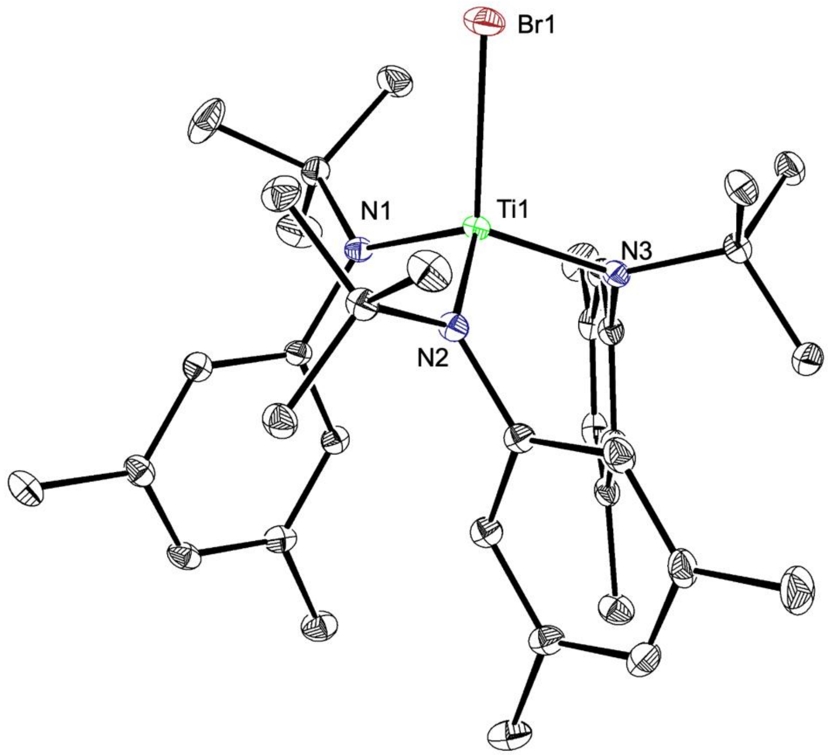

Figure S85. Representation of the solid-state structure of **Ti-6** (CCDC 2520871). Hydrogen atoms omitted for clarity. Ellipsoids presented with 30% probabilities.

Table S8. Crystal data for **Ti-6**.

Crystal data

$C_{36}H_{54}BrN_3Ti$	$F(000) = 1392$
$M_r = 656.63$	$D_x = 1.247$ Mg m^{-3}
Monoclinic, $P2_1/c$	Cu $K\alpha$ radiation, $\lambda = 1.54184$ Å
$a = 18.8456$ (4) Å	Cell parameters from 18020 reflections
$b = 11.1149$ (2) Å	$\theta = 2.5–74.7°$
$c = 17.8913$ (3) Å	$\mu = 3.60$ mm^{-1}
$\beta = 111.069$ (2)°	$T = 100$ K
$V = 3497.10$ (12) Å^3	Prism, clear reddish orange
$Z = 4$	0.37 × 0.20 × 0.18 mm

Data collection

XtaLAB Synergy, Dualflex, HyPix-Arc 150 diffractometer	7075 independent reflections
Radiation source: micro-focus sealed X-ray tube, PhotonJet (Cu) X-ray Source	6015 reflections with $I > 2\sigma(I)$
Mirror monochromator	$R_{int} = 0.057$
Detector resolution: 10.0000 pixels mm^{-1}	$\theta_{max} = 76.0°$, $\theta_{min} = 2.5°$
ω scans	$h = -23\varnothing23$
Absorption correction: multi-scan *CrysAlis PRO* 1.171.43.143a (Rigaku Oxford Diffraction, 2024) Empirical absorption correction using spherical harmonics, implemented in SCALE3 ABSPACK scaling algorithm.	$k = -13\varnothing13$
$T_{min} = 0.812$, $T_{max} = 1.000$	$l = -22\varnothing20$
48733 measured reflections	

Refinement

Refinement on F^2	Primary atom site location: dual
Least-squares matrix: full	Hydrogen site location: inferred from neighbouring sites
$R[F^2 > 2\sigma(F^2)] = 0.043$	H-atom parameters constrained
$wR(F^2) = 0.131$	$w = 1/[\sigma^2(F_o^2) + (0.0811P)^2 + 1.8648P]$ where $P = (F_o^2 + 2F_c^2)/3$
$S = 1.06$	$(\Delta/\sigma)_{max} = 0.001$
7075 reflections	$\Delta\rangle_{max} = 0.76$ e Å^{-3}
385 parameters	$\Delta\rangle_{min} = -0.66$ e Å^{-3}
0 restraints	

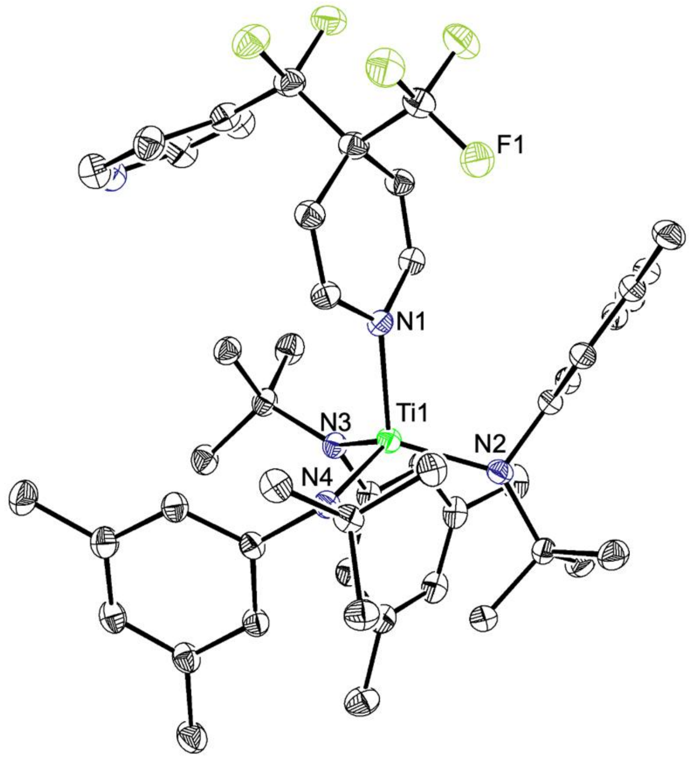

Figure S86. Representation of the solid-state structure of **Ti-5** (CCDC 2520872). Hydrogen atoms omitted for clarity. Ellipsoids presented with 30% probabilities.

Table S9. Crystal data for **Ti-5**.

Crystal data

$C_{48}H_{62}F_5N_5Ti$	$F(000) = 1808$
$M_r = 851.92$	$D_x = 1.303$ Mg m^{-3}
Monoclinic, $P2_1/n$	Cu $K\alpha$ radiation, $\lambda = 1.54184$ Å
$a = 19.5245$ (9) Å	Cell parameters from 32624 reflections
$b = 10.8308$ (4) Å	$\theta = 2.5–75.7°$
$c = 22.3895$ (12) Å	$\mu = 2.17$ mm^{-1}
$\beta = 113.489$ (6)°	$T = 100$ K
$V = 4342.3$ (4) Å^3	Plate, clear light orange
$Z = 4$	0.24 × 0.18 × 0.11 mm

Data collection

XtaLAB Synergy, Dualflex, HyPix-Arc 150 diffractometer	8806 independent reflections
Radiation source: micro-focus sealed X-ray tube, PhotonJet (Cu) X-ray Source	6603 reflections with $I > 2\sigma(I)$
Mirror monochromator	$R_{int} = 0.057$
Detector resolution: 10.0000 pixels mm^{-1}	$\theta_{max} = 76.6°$, $\theta_{min} = 2.6°$
ω scans	$h = -24\varnothing 24$
Absorption correction: multi-scan *CrysAlis PRO* 1.171.43.143a (Rigaku Oxford Diffraction, 2024) Empirical absorption correction using spherical harmonics, implemented in SCALE3 ABSPACK scaling algorithm.	$k = -12\varnothing 13$
$T_{min} = 0.769$, $T_{max} = 1.000$	$l = -27\varnothing 27$
82525 measured reflections	

Refinement

Refinement on F^2	Hydrogen site location: inferred from neighbouring sites
Least-squares matrix: full	H-atom parameters constrained
$R[F^2 > 2\sigma(F^2)] = 0.069$	$w = 1/[\sigma^2(F_o^2) + (0.1436P)^2 + 1.3379P]$ where $P = (F_o^2 + 2F_c^2)/3$
$wR(F^2) = 0.225$	$(\Delta/\sigma)_{max} = 0.003$
$S = 1.12$	$\Delta\rangle_{max} = 0.65$ e Å^{-3}
8806 reflections	$\Delta\rangle_{min} = -0.58$ e Å^{-3}
548 parameters	Extinction correction: *SHELXL2019*/2 (Sheldrick 2019), $Fc^* = kFc[1+0.001xFc^2\lambda^3/\sin(2\theta)]^{-1/4}$
0 restraints	Extinction coefficient: 0.0032 (3)
Primary atom site location: dual	

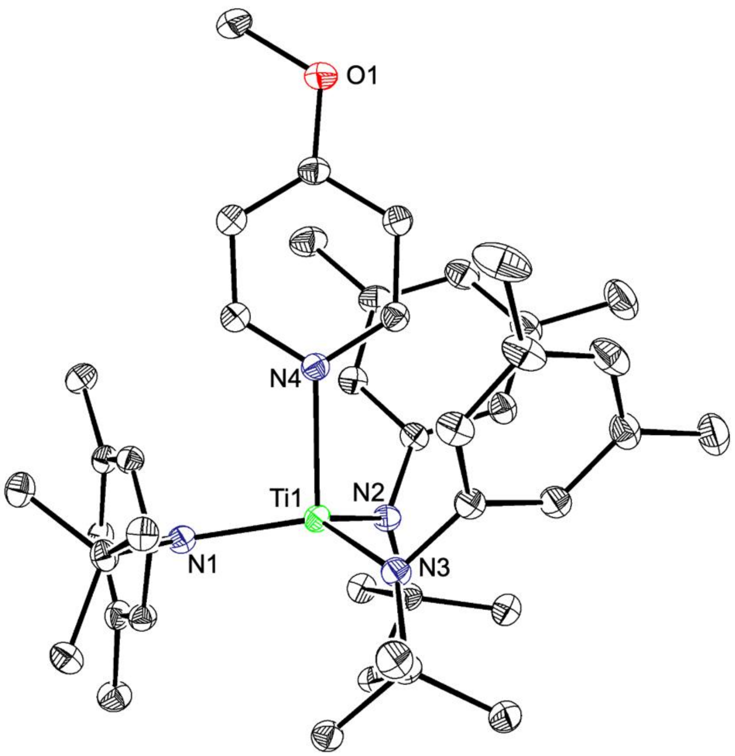

Figure S87. Representation of the solid-state structure of **Ti-3** (CCDC 2520873). Hydrogen atoms omitted for clarity. Ellipsoids presented with 30% probabilities.

Table S10. Crystal data for **Ti-3**.

Crystal data

$C_{42}H_{61}N_4OTi$	$F(000) = 1484$
$M_r = 685.84$	$D_x = 1.139$ Mg m^{-3}
Monoclinic, $P2_1/c$	Cu $K\alpha$ radiation, $\lambda = 1.54184$ Å
$a = 21.6898$ (8) Å	Cell parameters from 34558 reflections
$b = 9.6488$ (3) Å	$\theta = 4.1–75.4°$
$c = 19.4179$ (5) Å	$\mu = 2.07$ mm^{-1}
$\beta = 100.271$ (3)°	$T = 100$ K
$V = 3998.7$ (2) Å^3	Irregular, dull dark brown
$Z = 4$	0.15 × 0.13 × 0.10 mm

Data collection

XtaLAB Synergy, Dualflex, HyPix-Arc 150 diffractometer	8071 independent reflections
Radiation source: micro-focus sealed X-ray tube, PhotonJet (Cu) X-ray Source	6291 reflections with $I > 2\sigma(I)$
Mirror monochromator	R_{int} = 0.074
Detector resolution: 10.0000 pixels mm^{-1}	θ_{max} = 77.9°, θ_{min} = 2.1°
ω scans	h = -27∅26
Absorption correction: multi-scan *CrysAlis PRO* 1.171.43.143a (Rigaku Oxford Diffraction, 2024) Empirical absorption correction using spherical harmonics, implemented in SCALE3 ABSPACK scaling algorithm.	k = -12∅11
T_{min} = 0.801, T_{max} = 1.000	l = -22∅24
75748 measured reflections	

Refinement

Refinement on F^2	Primary atom site location: dual
Least-squares matrix: full	Hydrogen site location: inferred from neighbouring sites
$R[F^2 > 2\sigma(F^2)]$ = 0.063	H-atom parameters constrained
$wR(F^2)$ = 0.193	$w = 1/[\sigma^2(F_o^2) + (0.1131P)^2 + 1.8239P]$ where $P = (F_o^2 + 2F_c^2)/3$
S = 1.10	$(\Delta/\sigma)_{max}$ < 0.001
8071 reflections	$\Delta\rangle_{max}$ = 0.79 e Å^{-3}
449 parameters	$\Delta\rangle_{min}$ = -0.85 e Å^{-3}
0 restraints	

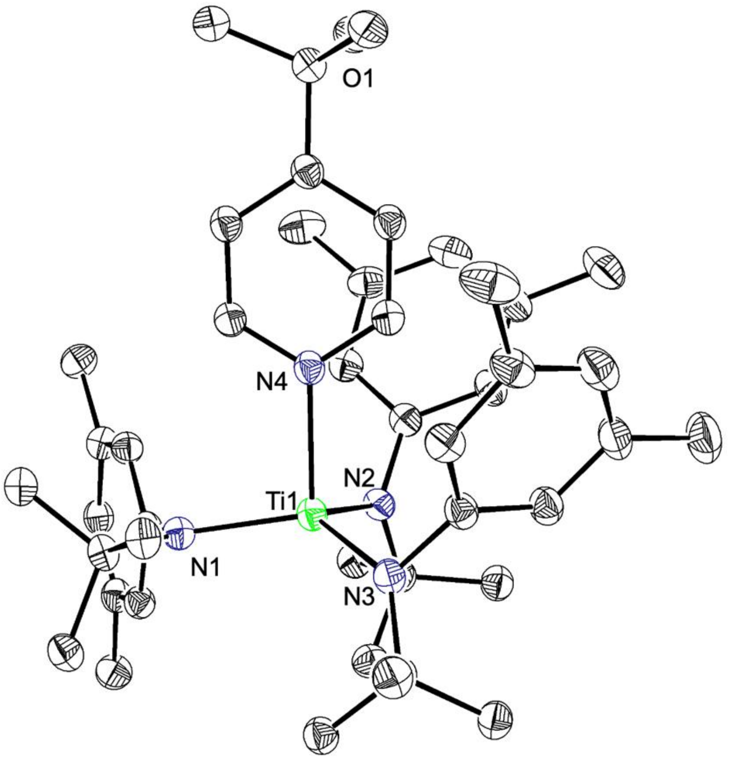

Figure S88. Representation of the solid-state structure of **Ti-4** (CCDC 2520874). Hydrogen atoms omitted for clarity. Ellipsoids presented with 30% probabilities.

Table S11. Crystal data for **Ti-4**.

Crystal data

$C_{45}H_{67}N_4Ti$	$F(000) = 1548$
$M_r = 711.92$	$D_x = 1.127$ Mg m^{-3}
Monoclinic, $P2_1/c$	Cu $K\alpha$ radiation, $\lambda = 1.54184$ Å
$a = 22.7755$ (12) Å	Cell parameters from 29127 reflections
$b = 9.7509$ (4) Å	$\theta = 4.0$–$75.5°$
$c = 19.6593$ (10) Å	$\mu = 1.97$ mm^{-1}
$\beta = 105.976$ (5)°	$T = 100$ K
$V = 4197.3$ (4) Å^3	Block, dull dark black
$Z = 4$	0.18 × 0.14 × 0.10 mm

Data collection

XtaLAB Synergy, Dualflex, HyPix-Arc 150 diffractometer	8486 independent reflections
Radiation source: micro-focus sealed X-ray tube, PhotonJet (Cu) X-ray Source	5917 reflections with $I > 2\sigma(I)$
Mirror monochromator	R_{int} = 0.064
Detector resolution: 10.0000 pixels mm^{-1}	θ_{max} = 76.2°, θ_{min} = 4.0°
ω scans	h = -28∅27
Absorption correction: multi-scan *CrysAlis PRO* 1.171.43.143a (Rigaku Oxford Diffraction, 2024) Empirical absorption correction using spherical harmonics, implemented in SCALE3 ABSPACK scaling algorithm.	k = -12∅11
T_{min} = 0.698, T_{max} = 1.000	l = -24∅24
78905 measured reflections	

Refinement

Refinement on F^2	Hydrogen site location: inferred from neighbouring sites
Least-squares matrix: full	H-atom parameters constrained
$R[F^2 > 2\sigma(F^2)]$ = 0.073	$w = 1/[\sigma^2(F_o^2) + (0.1461P)^2 + 0.8293P]$ where $P = (F_o^2 + 2F_c^2)/3$
$wR(F^2)$ = 0.240	$(\Delta/\sigma)_{max}$ = 0.001
S = 1.12	$\Delta\rangle_{max}$ = 0.93 e Å^{-3}
8486 reflections	$\Delta\rangle_{min}$ = -0.89 e Å^{-3}
470 parameters	Extinction correction: *SHELXL2019*/2 (Sheldrick 2019), $Fc^*=kFc[1+0.001xFc^2\lambda^3/\sin(2\theta)]^{-1/4}$
0 restraints	Extinction coefficient: 0.0029 (3)
Primary atom site location: dual	

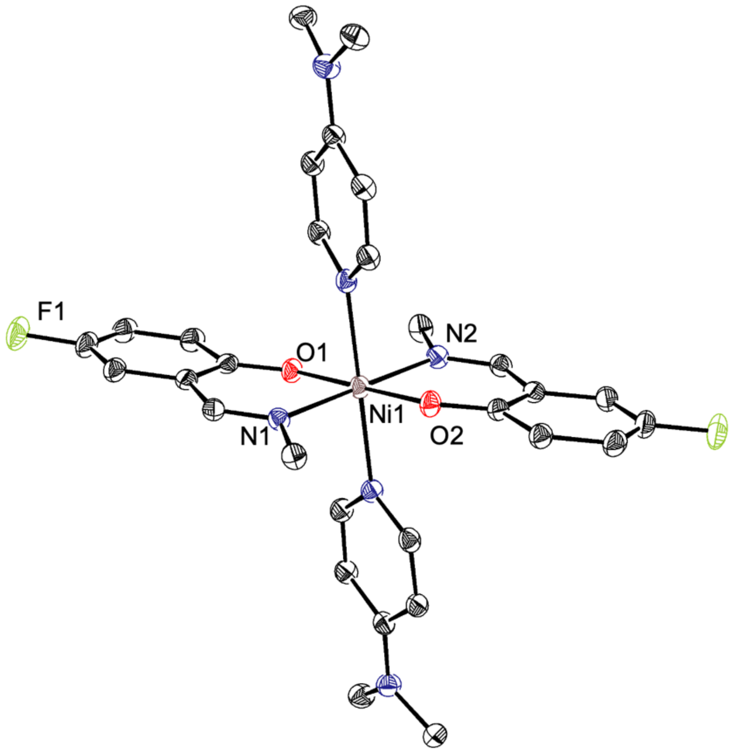

Figure S89. Representation of the solid-state structure of **Ni-2** (CCDC 2520875). Hydrogen atoms omitted for clarity. Ellipsoids presented with 30% probabilities. The unit cell contains two molecules of **Ni-2** and 1.5 molecules of benzene.

Table S12. Crystal data for **Ni-2**.

Crystal data

$4(C_{15}H_{17}FN_3Ni_{0.5}O)\cdot 3(C_3H_3)$	$F(000) = 2796$
$M_r = 1331.84$	$D_x = 1.366$ Mg m^{-3}
Monoclinic, $P2_1/n$	Cu $K\alpha$ radiation, $\lambda = 1.54184$ Å
$a = 16.1321$ (4) Å	Cell parameters from 24450 reflections
$b = 18.8146$ (4) Å	$\theta = 3.1–71.4°$
$c = 22.0829$ (6) Å	$\mu = 1.30$ mm^{-1}
$\beta = 104.940$ (2)°	$T = 100$ K
$V = 6476.0$ (3) Å^3	Irregular, clear light yellow
$Z = 4$	0.21 × 0.06 × 0.04 mm

Data collection

XtaLAB Synergy, Dualflex, HyPix-Arc 150 diffractometer	13340 independent reflections
Radiation source: micro-focus sealed X-ray tube, PhotonJet (Cu) X-ray Source	9874 reflections with $I > 2\sigma(I)$
Mirror monochromator	$R_{int} = 0.074$
Detector resolution: 10.0000 pixels mm^{-1}	$\theta_{max} = 88.1°$, $\theta_{min} = 3.1°$
ω scans	$h = -20\varnothing 19$
Absorption correction: multi-scan *CrysAlis PRO* 1.171.43.143a (Rigaku Oxford Diffraction, 2024) Empirical absorption correction using spherical harmonics, implemented in SCALE3 ABSPACK scaling algorithm.	$k = -23\varnothing 21$
$T_{min} = 0.786$, $T_{max} = 1.000$	$l = -27\varnothing 27$
120150 measured reflections	

Refinement

Refinement on F^2	Primary atom site location: dual
Least-squares matrix: full	Hydrogen site location: inferred from neighbouring sites
$R[F^2 > 2\sigma(F^2)] = 0.060$	H-atom parameters constrained
$wR(F^2) = 0.149$	$w = 1/[\sigma^2(F_o^2) + (0.0613P)^2 + 5.1429P]$ where $P = (F_o^2 + 2F_c^2)/3$
$S = 1.06$	$(\Delta/\sigma)_{max} = 0.001$
13340 reflections	$\Delta\rangle_{max} = 0.90$ e Å^{-3}
835 parameters	$\Delta\rangle_{min} = -0.37$ e Å^{-3}
63 restraints	

XIII. References

(1) Pangborn, A. B.; Giardello, M. A.; Grubbs, R. H.; Rosen, R. K.; Timmers, F. J. Safe and Convenient Procedure for Solvent Purification. *Organometallics* **1996**, *15*, 1518-1520.
(2) (a) Mills, L. R.; Di Mare, F.; Gygi, D.; Lee, H.; Simmons, E. M.; Kim, J.; Wisniewski, S. R.; Chirik, P. J. Phenoxythiazoline (FTz)-Cobalt(II) Precatalysts Enable C(sp2)–C(sp3) Bond-Formation for Key Intermediates in the Synthesis of Toll-like Receptor 7/8 Antagonists. *Angew. Chem. Int. Ed.* **2023**, *62*, e202313848. (b) Mills, L. R.; Simmons, E. M.; Lee, H.; Nester, E.; Kim, J.; Wisniewski, S. R.; Pecoraro, M. V.; Chirik, P. J. (Phenoxyimine)nickel-Catalyzed C(sp2)–C(sp3) Suzuki–Miyaura Cross-Coupling: Evidence for a Recovering Radical Chain Mechanism. *J. Am. Chem. Soc.* **2024**, *146*, 10124-10141.
(3) Peters, J. C.; Johnson, A. R.; Odom, A. L.; Wanandi, P. W.; Davis, W. M.; Cummins, C. C. Assembly of Molybdenum/Titanium μ-Oxo Complexes via Radical Alkoxide C−O Cleavage. *J. Am. Chem. Soc.* **1996**, *118*, 10175-10188.
(4) Stoll, S.; Schweiger, A. EasySpin, a comprehensive software package for spectral simulation and analysis in EPR. *Journal of Magnetic Resonance* **2006**, *178*, 42-55.
(5) Mendiratta, A.; Cummins, C. C. Heterobimetallic Reductive Cross-Coupling of Benzonitrile with Carbon Dioxide, Pyridine, and Benzophenone. *Inorg. Chem.* **2005**, *44*, 7319-7321.
(6) Aitken, R. A.; Hodgson, P. K. G.; Morrison, J. J.; Oyewale, A. O. Flash vacuum pyrolysis over magnesium. Part 1. Pyrolysis of benzylic, other aryl/alkyl and aliphatic halides. *J. Chem. Soc., Perkin Trans. 1* **2002**, 402-415.
(7) Chatgilialoglu, C.; Ingold, K. U.; Scaiano, J. C. Rate constants and Arrhenius parameters for the reactions of primary, secondary, and tertiary alkyl radicals with tri-n-butyltin hydride. *J. Am. Chem. Soc.* **1981**, *103*, 7739-7742.
